# Comparative gene expression profiling between human cultured myotubes and skeletal muscle tissue

**DOI:** 10.1186/1471-2164-11-125

**Published:** 2010-02-22

**Authors:** Frederic Raymond, Sylviane Métairon, Martin Kussmann, Jaume Colomer, Andres Nascimento, Emma Mormeneo, Cèlia García-Martínez, Anna M Gómez-Foix

**Affiliations:** 1 Nestlé Research Center, Vers-Chez-Les-Blanc, CH-1000 Lausanne 26, Switzerland; 2 Unitat de Patologia Neuromuscular, Servei de Neurologia, Hospital Sant Joan de Déu, Barcelona, Spain; 3 CIBER de Diabetes y Enfermedades Metabólicas Asociadas (CIBERDEM), Departament de Bioquímica i Biologia Molecular, IBUB, Facultat de Biologia, Universitat de Barcelona, Diagonal 645, 08028-Barcelona, Spain

## Abstract

**Background:**

A high-sensitivity DNA microarray platform requiring nanograms of RNA input facilitates the application of transcriptome analysis to individual skeletal muscle (SM) tissue samples. Culturing myotubes from SM-biopsies enables investigating transcriptional defects and assaying therapeutic strategies. This study compares the transcriptome of aneurally cultured human SM cells versus that of tissue biopsies.

**Results:**

We used the Illumina expression BeadChips to determine the transcriptomic differences between tissue and cultured SM samples from five individuals. Changes in the expression of several genes were confirmed by QuantiGene Plex assay or reverse transcription real-time PCR. In cultured myotubes compared to the tissue, 1216 genes were regulated: 583 down and 633 up. Gene ontology analysis showed that downregulated genes were mainly associated with cytoplasm, particularly mitochondria, and involved in metabolism and the muscle-system/contraction process. Upregulated genes were predominantly related to cytoplasm, endoplasmic reticulum, and extracellular matrix. The most significantly regulated pathway was mitochondrial dysfunction. Apoptosis genes were also modulated. Among the most downregulated genes detected in this study were genes encoding metabolic proteins AMPD1, PYGM, CPT1B and UCP3, muscle-system proteins TMOD4, MYBPC1, MYOZ1 and XIRP2, the proteolytic CAPN3 and the myogenic regulator MYF6. Coordinated reduced expression of five members of the GIMAP gene family, which form a cluster on chromosome 7, was shown, and the GIMAP4-reduction was validated. Within the most upregulated group were genes encoding senescence/apoptosis-related proteins CDKN1A and KIAA1199 and potential regulatory factors HIF1A, TOP2A and CCDC80.

**Conclusions:**

Cultured muscle cells display reductive metabolic and muscle-system transcriptome adaptations as observed in muscle atrophy and they activate tissue-remodeling and senescence/apoptosis processes.

## Background

Oligonucleotide microarrays can reveal gene expression profiles of SM tissue and provide valuable insight into molecular pathways involved in pathogenesis or abnormally regulated in disease. Various human disorders that affect the SM tissue have been analyzed using microarray technology, including disuse atrophy [1], myositis [2,3], Duchenne muscular dystrophy (DMD) [4-6] and others [7-9].

One of the limitations of applying microarray analyses to such context is the amount of tissue material needed [10]. For SM studies, genome-wide expression profiling has in some cases been applied to pooled samples from multiple patients [4,7]; in others, microarrays with a restricted number of genes were used [2,6,8,9]. The possibility of performing whole transcriptome microarray analysis on small amounts of tissue material facilitates its application to tissue samples from individual subjects.

The culturing of myotubes from SM biopsies opens an alternative to examine transcriptional defects with microarrays and enables therapeutic strategies to be assayed. SM cell cultures can be established by the explant technique [11], which is based on the presence of myogenic progenitors, the satellite cells [12]. Satellite cells are not committed to fiber type lineages [13,14]. When satellite cells are stimulated with growth factors, they generate myoblasts, which can replicate a limited number of times and be induced to fuse and form multinucleated myotubes. The drawback of cell cultures in general is that cells change their phenotype, which may alter the expression of certain genes and compromise the phenotypic expression of the disease. Indeed, aneurally cultured human SM cells remain relatively immature as shown in studies of the protein isoform pattern [14-16] and microarray analysis of the transcriptome [17].

There were no data on transcriptome differences between cultured and tissue human SM cells. We deployed an automated high-sensitivity microarray platform to identify genes differentially expressed between aneurally cultured myotubes derived from human SM biopsies by the explant technique and the SM tissue samples. We provide insight into the phenotype of the cultured human SM, which is a valuable cell model for pathogenesis studies and therapeutic assays.

## Methods

### Skeletal muscle specimens and cultures

Paravertebral muscles biopsies were obtained from five subjects: females of age 12-15 years devoid of neuromuscular disease during surgery for idiopathic scoliosis. Informed consent and approval from the Ethics Committee of the Hospital Sant Joan de Déu (Barcelona) was obtained. Biopsies were embedded into RNAlater for RNA extraction or into cell culture medium. Cultures were prepared through an explant technique [11,17]. Briefly, SM biopsy pieces were dissected under a stereomicroscope; fat, connective tissue and blood were removed; and the pieces were frozen in DMEM medium with 25% fetal bovine serum (FBS) and 6% DMSO. To set up the culture, first the biopsy pieces were spaced embedded in a semi-solid matrix, composed of 5 ml of DMEM/M-199 medium (3:1) with 37.5% FBS and 1.25 ml of human plasma (Sigma-Aldrich, St. Louis, MO, USA), overspread onto tissue culture plates, which were then incubated for 6 to 8 days to permit fibroblast outgrowth. The biopsy pieces were then removed from the matrix with forceps under the stereomicroscope, dissected and embedded in a matrix, composed of 1 ml of human plasma and 2 ml of 1.5% gelatin, spotted and stuck onto new dishes and overlaid with DMEM/M-199 medium (3:1) with 10% FBS, 10 μg/ml insulin, 4 mM glutamine, 25 ng/ml fibroblast growth factor, and 10 ng/ml epidermal growth factor, for 5 to 7 days to permit myoblast proliferation and migration. After myoblast generation, the biopsy pieces were removed, and eventually re-explanted, and myoblast monolayers were dissociated with trypsin and subcultured. Myogenic cells from the same explant were subjected to limited consecutive subcultures to avoid differences resulting from senescence [17]. Myotubes were derived from confluent myoblast cultures; immediately after initiation of myoblast fusion, medium was replaced by DMEM/M-199 medium (3:1) with 10% FBS and 10 μg/ml insulin, to further stimulate differentiation. Myotubes were used 7 days later. Myotube preparations were labeled B19, B22, B24, B25 and B26.

### Immunocytochemistry

Cells grown on coverslips were fixed in 4% paraformaldehyde in PBS for 15 min, then washed in PBS, and incubated for 10 min in PBS containing 50 mM NH_4_Cl, 10 min in PBS containing 20 mM glycine, 10 min in PBS containing 0.1% Triton X-100 and 30 min in PBS containing 10% FBS. Subsequently coverslips were incubated for 1 h at room temperature with a rabbit anti-desmin antibody (1:100; AB907 Millipore, Billerica, MA, USA). Primary antibody was detected with an Alexa Fluor-488 goat anti-rabbit antibody (1:500; Molecular Probes, Eugene, OR, USA). Both primary and secondary antibodies were diluted in blocking solution. Nuclei were stained with Hoescht (1 μg/ml) during secondary antibody incubation. After staining, samples were mounted on Mowiol mounting medium and analyzed with a Leica TCS SP2 confocal microscope. Images were processed using Photoshop CS software (Adobe Corp, San Jose, CA, USA). From 200 to 500 nuclei in each of the 5 myotube cultures were analyzed with ImageJ (Rasband WS, ImageJ, National Institutes of Health, Bethesda, MD, USA, http://rsb.info.nih.gov/ij/ and the mean ± SD values of the percentage of nuclei located in desmin-labeled myotubes were calculated.

### Gene expression analysis

Ten samples, 5 from cultured SM cells, and 5 from SM biopsies were analyzed by DNA microarrays. Total RNA was extracted from SM biopsies with RNeasy fibrous tissue kit and homogenized using a TissueLyser, and from cultured myotubes with RNeasy kit (Qiagen, Valencia, CA, USA), with the same DNase treatment than in the RNeasy fibrous tissue kit, and homogenized with a Polytron. RNA samples were quantified with the RiboGreen RNA Quantification Kit (Molecular Probes), and monitored with the Agilent 2100 Bioanalyzer, to check high-quality RNA (RNA integrity = 8). We used HumanRef-8 v2.0 Expression BeadChips (Illumina, San Diego, CA, USA), which comprise probes to interrogate 22200 transcripts, based on the curated content of the NCBI Reference Sequence database, release 17.

An aliquot of 150 ng of total RNA was used to produce double-stranded cDNA, followed by transcription *in vitro*, and by cRNA labeling with biotin using the TotalPrep RNA Amplification kit (Applied Biosystems/Ambion, Austin, TX, USA) recommended by Illumina. This method is based on the protocol developed in J. Eberwine's laboratory [18]. The procedure consists of reverse transcription with an oligo(dT) primer bearing a T7 promoter using a reverse transcriptase engineered to produce high yields of first strand cDNA. The reverse transcriptase catalyzes the synthesis of full-length cDNA which then undergoes second strand synthesis and clean-up to become a template for *in vitro *transcription (IVT) with T7 RNA Polymerase. The IVT, along with biotin UTP, is used to generate hundreds to thousands of biotinylated, antisense RNA copies of each mRNA. These procedures were performed on an automated system primarily developed for the preparation of samples for Affymetrix arrays [19], which we adapted for the Illumina procedure. It is composed by a Microlab Star liquid handling system (Hamilton, Bonaduz, Switzerland) for reagent and sample pipetting and mixing, coupled with a thermocycler TRobot (Biometra, Goettingen, Germany) for incubations, and a microtiter plate reader SpectraMax M2 (Molecular Devices, Sunnyvale, CA, USA) for nucleic acids quantification with RiboGreen assay. The 10 samples analyzed in the present experiment were processed in a single robotic session. The labeled-cRNAs thus produced were measured with the Agilent 2100 Bioanalyzer to control for length. All cRNA sizes were close to 1200 nt and therefore eligible for hybridization onto the microarrays. Then, 750 ng of labeled-cRNAs was added to the hybridization mix, which contained control oligonucleotides in hybridization buffer. Then, 15 μl of each hybridization mix was dispensed on the BeadArrays. After hybridization (16 h, 58°C), the arrays were washed to remove non-hybridized material and were stained with streptavidin-Cy3, which bound to biotin. Scanning was performed using the BeadArray Reader, which provided intensity values for all transcripts. Bead redundancy allowed the calculation of a detection p-value which served at declaring the transcripts "significantly detected" or not. Signal intensities were extracted and summarized in the BeadStudio software. Data were expressed as absolute intensities, to which we applied a background correction. Background was calculated for each array by the average signal of the negative control probes. All microarray experiments were run at the same time. The Illumina HumanRef-8 BeadChip is a glass slide comprising 8 identical microarrays. We therefore used 2 slides for this experiment. To minimize possible batch effect that can occur between the two glass slides, we randomized the sample distribution in the following order: in one slide A (B19 *in vitro*), B (B26 *in vitro*), C (B25 *in vivo*), D (B24 *in vitro*), E (B24 *in vivo*), F (B22 *in vivo*), G (B26 *in vivo*) and H (B25 *in vitro*) and in the other slide A (B19 *in vivo*) and B (B22 *in vitro*). "*In vitro" *stands for cultured myotubes and "*in vivo" *means SM tissue biopsies. As shown in a recent study [20], randomization on Illumina slides greatly improves the results reducing the number of possible false positive occurrences that would lead to a misinterpretation of the data. Microarray data files have been deposited in GEO Omnibus [http://www.ncbi.nlm.nih.gov/geo/ GEO accession number GSE17503].

QuantiGene Plex 2.0 assay (Panomics/Affymetrix, Fremont, CA, USA) was used for confirmation of the microarray analysis. The advantage of this assay is that no RNA amplification is needed, unlike microarrays or quantitative PCR. A comparison to other gene expression platforms in the frame of the MAQC project demonstrated the sensitivity and accuracy of QuantiGene techniques [21]. This technology uses three sets of pooled oligonucleotides that function to capture, provide binding sites for signal amplification molecules, and stabilize the mRNA of interest. The capture extender (CE) pool contains oligonucleotides that are complementary to the mRNA of interest and to capture probes (CPs) covalently linked to carboxylated fluorescent-encoded micro spheres (Luminex, Austin, TX, USA). The label extender (LE) pool has oligonucleotides that contain sequences complementary to the mRNA of interest and to the amplifier molecules which carry a streptavidin phycoerythrin (SAPE) conjugate. The blocker (BL) pool contains oligonucleotides that are complementary to the mRNA where neither CE nor LE bind and it protects the mRNA by maintaining an intact RNA-DNA hybrid. The micro spheres (or beads) thus prepared are analyzed with a Luminex reader, which utilizes two lasers, the first for bead identification, measuring the fluorescent signal specific to each bead type, and the second for mRNA quantification, determining the fluorescence level of SAPE and therefore the expression level of the transcript. The assay was performed according to the manufacturer's instructions, accessible at: http://panomics.com/downloads/UM13075_RevB_QGP2_080430.pdf. Briefly, the RNAs extracted from tissue and cell samples were deposited on a 96-well plate along with the QuantiGene hybridization solution (containing RNase-free water, lysis mixture, blocking reagent, proteinase K, capture beads and probe set). The hybridization plate was then sealed and placed in a shaking incubator (Vortemp 56 Shaker/Incubator, Labnet International, Inc., Woodbridge, NJ, USA) at 54°C and 600 rpm for overnight hybridization (18-22 h). After centrifugation to remove condensation, the hybridization mixture was transferred to a pre-wetted filter plate, where the mixture was filtered and washed three times in wash buffer. All washing steps were performed with a MultiScreen Vacuum Manifold (Millipore) at low pressure, and after the final wash, the filter plate bottom was blotted to remove any remaining liquid to prevent leakage. The samples were then incubated successively in three working solutions: pre-amplifier and amplifier, biotinylated probes and SAPE. Two washes were performed between each incubation to remove unbound reagents. The plate was covered with aluminum foil during SAPE incubation to prevent photo bleaching. For Luminex measurement, after a calibration of the Luminex, the plate was placed in the instrument. Prior to the measurement, each bead type was identified in the Luminex software in order to specify to which transcripts the beads corresponded. At the start of the measurement, the content of each well was aspirated and pushed towards the lasers. As occurs in a flow cytometer, the beads passed successively in front of the two lasers. Beads were identified, and fluorescent signals were measured. The final outcome was a raw data file containing signal intensity and background level for each gene in each sample. Data were analyzed using the transferrin receptor (TFRC) gene as the reference gene, since its expression was similar within the two examined groups, cultured myotubes and SM biopsies.

For reverse transcription (RT) and real-time PCR, an aliquot of 0.5 μg of total RNA was retro-transcribed (RT) with TaqMan reverse transcription reagents from Applied Biosystems (Foster City, CA, USA) using random hexamers and in a 25 μl total volume reaction. Real-time PCR was performed on 1 μl of the RT reaction mixture with the TaqMan universal PCR master mix and TaqMan Gene Expression Assays using the ABI PRISM 7700 sequence detection system. Probes for PYGM (Hs00194493_m1), UCP3 (Hs00243297_m1), THY1 (Hs00264235_s1) and B2M (Hs99999907_m1) were from Applied Biosystems. The threshold cycle (Ct) values of the reference probe beta-2 microglobulin (B2M) were subtracted from each target probe Ct values (ΔCt), the ΔCt values of the SM biopsies were subtracted from the ΔCt values of myotube cultures. Data were expressed as mean values of 2^-ΔΔCT ^for upregulated genes and 1/2^-ΔΔCT ^for downregulated ones and the significance of differences was estimated by a paired t-test.

### Statistical analysis and filtering

After the scanning of the microarrays, BeadStudio generated a dendrogram to control for the sample clustering. Subsequent steps of quality control (QC), statistical analysis and filtering of microarray data were carried out with GeneSpring GX software v10.0.2 (Agilent Technologies, Santa Clara, CA, USA). Raw data with background subtracted were loaded from Illumina BeadStudio to GeneSpring GX. This step was followed by quantile normalization and log2 transformation. The samples were then grouped into two conditions which we called "*in vitro*" and "*in vivo*", corresponding respectively to the SM cultures and the tissue biopsies. GeneSpring GX was used as the first step for QC of data on all the samples with two unsupervised methods, Pearson correlation and Principal Component Analysis (PCA). The PCA vector space transformation was used to reduce multidimensional data sets to lower dimensions for analysis to assess the behavior of the samples and to identify or confirm possible outliers. Within the quality control step, Genespring GX enables to compute the 3 first principal components. The percentage of total variance that each of these 3 principal components capture is shown in the legend of Additional File 1.

To assess which genes were differentially expressed between the two conditions examined, we performed a corrected paired t-test on all probes. Because of the small number of biological replicates, we used the Benjamini-Hochberg false discovery rate (FDR) method controlling for false positives, in order to select genes with the lowest FDR. This method is less stringent than other corrections such as the Bonferroni, and provides a good balance between discovery of statistically significant genes and limitation of false positive occurrences. A corrected p-value cutoff of 0.01 was used to select the regulated genes with the lowest FDR. A fold change value was also computed by GeneSpring GX to assess the level and the direction of the gene regulation. Fold change was calculated as the absolute ratio of normalized intensities between the mean values of the samples in the two conditions, cultured myotubes and SM tissue biopsy, grouped.

Data from QuantiGene Plex were also loaded into GeneSpring GX software. Originally GeneSpring GX was designed for gene expression microarray experiments and uses the main commercially available arrays (Affymetrix, Illumina, Agilent and CodeLink). However, it is possible to configure a "custom" array. The QuantiGene Plex assay was therefore configured in GeneSpring GX. After normalization to the TFRC control gene, data were log2 transformed, and significance of differential expression was assessed by a paired t-test.

### Bioinformatic analysis

Functional annotation and classification analysis was also carried out with GeneSpring GX program, running a Gene Ontology (GO) analysis on the gene set selected with the statistical analysis described above. GeneSpring GX computed a p-value to quantify the significance in the GO analysis. This p-value is the probability that a random subset of x genes drawn from the total set of n genes will have y or more entities containing a given GO term. This probability is described by a standard hypergeometric distribution. GeneSpring GX uses the hypergeometric formula from first principles to compute this probability. Since a large number of hypotheses will be tested, some form of correction is required. GeneSpring GX addresses this issue using the Benjamini-Yekutieli correction, which takes into account the dependency among the GO terms. In the GO analysis done in GeneSpring GX, a low p-value therefore implies that the given GO term is enriched. The Gene Ontology data in GX 10.0.2 is compiled from version 1.2 of OBO (Open Biomedical Ontologies).

Data were interpreted using Ingenuity Pathways Analysis (IPA) (Ingenuity Systems, Redwood City, CA, USA) http://www.ingenuity.com. The list of significantly regulated genes selected by the microarray analysis described above was loaded in IPA with the following criteria: Reference set: HumanRef-8 v2.0; Direct and Indirect relationships included; filtered by species (human), and in a second round, by tissue (skeletal muscle). Then IPA computed the data to generate significant networks of genes that are associated with particular biological functions, diseases, and molecular processes. It showed also the canonical pathways stimulated by the experiment. IPA used the right-tailed Fisher's exact test to extract significant pathways. IPA canonical pathways are well-characterized metabolic and cell-signaling pathways coming from articles, reviews, books, and KEGG Ligand.

Gene cluster analysis was performed with the REEF (REgionally Enriched Features in genomes) program, which scans the genome using a sliding window approach, and calculates the statistical significance of each window using the hypergeometric distribution and the false discovery rate [22].

## Results

### Genes differentially expressed in cultured *versus *skeletal muscle tissue

Microarray analyses were performed on the 5 SM cultures and the 5 SM biopsies. We confirmed that myotubes were the prevalent cell type in each of the SM cultures by immunostaining for desmin, a muscle-specific intermediate filament protein [23] (Additional file 1, Figure S1). Most of the Hoescht-stained nuclei were located in desmin-labeled myotubes: B19 (78.3 ± 3.12%), B22 (61.4 ± 6.60%), B24 (61.8 ± 8.36%), B25 (83.6 ± 14.50%) and B26 (79.7 ± 5.05%). The microarray analysis showed that in the cultured myotubes compared to the tissue biopsies 1260 transcripts, which correspond to 1216 nuclear genes, were differentially expressed (selection criteria: absolute fold-change value > 2 with a corrected p-value of p < 0.01). Of these, 583 were downregulated and 633 were upregulated. The complete list of regulated genes is shown in Additional file 2, Table S1. On the basis of the correlation analysis performed, Additional file 3, Figure S2 shows that the samples were clearly separated based on their condition cultured myotubes or SM biopsy. The dendrogram also illustrates that the paired samples were differently distributed in the two conditions. PCA and correlation matrix procedures show that the two experimental conditions were well separated and there were no particular outliers in the experiment. Moreover, they highlight that the variability was higher within the SM culture group (*in vitro*). Pearson coefficients are also provided; the average Pearson coefficient was 0.97 for the SM biopsies and 0.93 for the SM cultures. PCA, correlation matrix and dendrogram also confirmed that there was no significant batch effect between the two Illumina slides in our experiment. In an attempt to restrict differentially expressed genes to those expressed in SM, microarray data were filtered according to the IPA knowledge-base, so that genes expressed in SM were selected. SM-specificity is shown in Additional file 2, Table S1. IPA knowledge about mRNA expression in tissues is based on two sources: the GNF body atlas [24], and literature findings. Among the downregulated genes, 384 passed this filter along with 318 of the upregulated genes. The filtering served as a means for identifying genes that are expressed in other cell types, present in the SM tissue, within the most regulated genes. For instance, hemoglobin HBB and HBA2 genes, which are mainly expressed in erythroid cells [25], from the most downregulated group and the THY1 gene, which is mainly expressed in human fibroblasts, neurons and endothelial cells [26], from the most upregulated group. Remarkably, the ratios between the normalized signal of the THY1 and desmin gene markers in the SM cultures were in a narrow range: B19 (1.94), B22 (1.12), B24 (1.42), B25 (1.28) and B26 (1.44), again indicating that fibroblast presence did not vary widely within SM cultures. However, the THY1/desmin ratios were from 1.3- to 3.2-fold higher in the SM cultures than in the SM biopsies.

The complete set of differentially expressed genes were classified, separately for downregulated and upregulated genes, according to GO (Additional file 4, Table S2A). With respect to the cellular component, genes that were downregulated in cultured myotubes were mainly associated with cytoplasm and enriched in mitochondria. The over-represented biological processes were metabolism, with the largest group of regulated genes, and muscle-system/contraction. With respect to metabolism, genes were mainly involved in the generation of precursor metabolites and energy, cellular respiration and oxidative phosphorylation; quinone cofactor metabolism was also affected. The most augmented molecular function was oxidoreductase activity. Among the genes that were upregulated in cultured myotubes, the enriched cellular components were cytoplasm (with the largest group associated with endoplasmic reticulum (ER)) and the extracellular matrix. The enriched molecular function here was oligosaccharyl transferase activity. The augmented biological process was modification of an amino acid residue in a protein: N-linked posttranslational glycosylation at asparagine residues. GO annotations of the SM-expressed subset of downregulated genes (Additional file 4, Table S2B) did not differ greatly from those of the whole gene set, except for the occurrence of the following over-represented GO annotations: cytoskeletal protein binding (molecular function); fatty acid beta-oxidation and organic acid metabolism-related terms (metabolic processes); and muscle development (biological process). There was no GO annotation enrichment within the upregulated SM-expressed subset for molecular function and biological process categories (Additional file 4, Table S2B).

The most regulated SM-expressed genes on the basis of the fold-change are listed in Table 1. This selection was performed on the whole set of 1260 regulated transcripts (data not shown) before the replicated genes were removed. In Additional file 5, Table S3, the standard deviation for each of these genes, based on the normalized signals, is shown. A closer examination of the downregulated group reveals several muscle-system genes encoding proteins of myofilaments MYL3; associated/regulatory proteins TMOD4, MYBPC1, MYOZ3, MYOZ1, NRAP and XIRP2; the regulator of the muscle sarcomere CAPN3 [27]; and calcium-related proteins CASQ1 and S100A1. Genes encoding metabolic proteins are included in this group: LPL, CPT1B, PYGM and AMPD1. Potential regulatory factors are also present: members of the ankyrin repeat- and SOCS (suppressor of cytokine signaling) box-containing protein (ASB) family, ASB10 and ASB12; members of the muscle ankyrin repeat protein (MARP) family, ANKRD2 and ANKRD23; and the transcription factor MYF6. The ion channels CLIC5 and KCNA7, the transporters AQP4 and MB, and the neurite outgrowth inhibitor RTN4 are included. Other genes such as those encoding NIPSNAP3B, HHATL and MYOC have no defined function in SM.

**Table 1:** Top regulated genes filtered by expression in skeletal muscle

Gene ID	Gene Name	Description	FC
**DOWNREGULATED**			
4151	MB	myoglobin	-2541
761	CA3	carbonic anhydrase III, muscle specific	-2391
29765	TMOD4	tropomodulin 4 (muscle)	-1564
4634	MYL3	myosin, light chain 3, alkali; ventricular, skeletal, slow	-1529
844	CASQ1	calsequestrin 1 (fast-twitch, skeletal muscle)	-1496
270	AMPD1	adenosine monophosphate deaminase 1 (isoform M)	-1320
4604	MYBPC1	myosin binding protein C, slow type	-1128
136371	ASB10	ankyrin repeat and SOCS box-containing 10	-869.2
91977	MYOZ3	myozenin 3	-841.6
4023	LPL	lipoprotein lipase	-608.5
58529	MYOZ1	myozenin 1	-546.7
53405	CLIC5	chloride intracellular channel 5	-504.7
6271	S100A1	S100 calcium binding protein A1	-366.4
57467	HHATL	hedgehog acyltransferase-like	-364.5
57142	RTN4	reticulon 4	-338.4
6123	RPL3L	ribosomal protein L3-like	-317.8
200539	ANKRD23	ankyrin repeat domain 23	-298.1
4892	NRAP	nebulin-related anchoring protein	-294.9
123722	FSD2	fibronectin type III and SPRY domain containing 2	-293.3
760	CA2	carbonic anhydrase II	-290.2
5837	PYGM	phosphorylase, glycogen; muscle (McArdle syndrome, glycogen storage disease type V)	-277.4
84448	ABLIM2	actin binding LIM protein family, member 2	-276.9
4618	MYF6	myogenic factor 6 (herculin)	-265.7
142689	ASB12	ankyrin repeat and SOCS box-containing 12	-228.6
339456	TMEM52	transmembrane protein 52	-217.2
129446	XIRP2	xin actin-binding repeat containing 2	-216.1
26287	ANKRD2	ankyrin repeat domain 2 (stretch responsive muscle)	-200.5
712	C1QA	complement component 1, q subcomponent, A chain	-197.9
10840	ALDH1L1	aldehyde dehydrogenase 1 family, member L1	-196.1
9452	ITM2A	integral membrane protein 2A	-178.5
5104	SERPINA5	serpin peptidase inhibitor, clade A (alpha-1 antiproteinase, antitrypsin), member 5	-175.8
825	CAPN3	calpain 3, (p94)	-164.2
4653	MYOC	myocilin, trabecular meshwork inducible glucocorticoid response	-162.9
55335	NIPSNAP3B	nipsnap homolog 3B (C. elegans)	-161.9
361	AQP4	aquaporin 4	-160.4
4129	MAOB	monoamine oxidase B	-158.9
1375	CPT1B	carnitine palmitoyltransferase 1B (muscle)	-151.4
3743	KCNA7	potassium voltage-gated channel, shaker-related subfamily, member 7	-137.9

**UPREGULATED**			
57214	KIAA1199	KIAA1199	+1279
5806	PTX3	pentraxin-related gene, rapidly induced by IL-1 beta	+491.7
1026	CDKN1A	cyclin-dependent kinase inhibitor 1A (p21, Cip1)	+192.8
114801	TMEM200A	transmembrane protein 200A	+184.9
2335	FN1	fibronectin 1	+82.93/43.39^D^
23362	PSD3	pleckstrin and Sec7 domain containing 3	+82.63
124565	SLC38A10	solute carrier family 38, member 10	+68.68
7077	TIMP2	TIMP metallopeptidase inhibitor 2	+68.09
7153	TOP2A	topoisomerase (DNA) II alpha 170kDa	+43.58
8985	PLOD3	procollagen-lysine, 2-oxoglutarate 5-dioxygenase 3	+43.02
6505	SLC1A1	solute carrier family 1 (neuronal/epithelial high affinity glutamate transporter, system Xag), member 1	+40.97
9663	LPIN2	lipin 2	+40.92
54480	CSGlcAT	chondroitin sulfate glucuronyltransferase	+39.96
338773	TMEM119	transmembrane protein 119	+38.12
5352	PLOD2	procollagen-lysine, 2-oxoglutarate 5-dioxygenase 2	+36.07/25.77^D^
493869	GPX8	glutathione peroxidase 8	+35.9
3091	HIF1A	hypoxia-inducible factor 1, alpha subunit (basic helix-loop-helix transcription factor)	+34.89
115908	CTHRC1	collagen triple helix repeat containing 1	+33.74
8572	PDLIM4	PDZ and LIM domain 4	+31.9
151887	CCDC80	coiled-coil domain containing 80	+31.02/17.06^D^
4323	MMP14	matrix metallopeptidase 14 (membrane-inserted)	+29.75
23114	NFASC	neurofascin homolog (chicken)	+26.53
55165	CEP55	centrosomal protein 55kDa	+25.73
5270	SERPINE2	serpin peptidase inhibitor, clade E (nexin, plasminogen activator inhibitor type 1), member 2	+24.59
54431	DNAJC10	DnaJ (Hsp40) homolog, subfamily C, member 10	+24.52
6695	SPOCK1	sparc/osteonectin, cwcv and kazal-like domains proteoglycan (testican) 1	+24.35
2629	GBA	glucosidase, beta; acid (includes glucosylceramidase)	+24.33
7421	VDR	vitamin D (1,25- dihydroxyvitamin D3) receptor	+23.83
4643	MYO1E	myosin IE	+23.46
166929	SGMS2	sphingomyelin synthase 2	+22.63
7058	THBS2	thrombospondin 2	+22.59

The list includes the 10% most differentially expressed genes in cultured myotubes compared to SM tissue. The set of the 1260 regulated gene transcripts, including replicated genes, was selected on the basis of their expression in SM, according to the IPA knowledge-base and the fold-change and was ordered by the magnitude of the fold change. For each gene, the fold change in gene expression was calculated between mean values in cultured myotubes versus SM biopsy. Duplicated genes on the microarray are marked (^D^) and the two values are shown. The significance of differences was estimated by a Benjamini-Hochberg corrected paired t-test (p < 0.01). The first column lists the ID for Entrez Gene, the second the gene name, the third the description of the gene and the fourth the fold change (FC) with a negative symbol for downregulated genes and a positive symbol for upregulated genes.

Among the most upregulated genes detected in this study (Table 1) are well-known regulatory factors such as CDKN1A/p21, TOP2A and HIF1A. Several genes are related to the extracellular matrix, such as those encoding: the Ca(2+)-binding proteoglycan SPOCK1; isoforms of lysyl hydroxylase (PLOD3, PLOD2) [28], an enzyme that catalyzes the formation of hydroxylysine in collagens; CSGlcA-T, which is involved in the synthesis of chondroitin sulphate as a glucuronyltransferase [29]; the metalloproteinase MMP14; TIMP2, an inhibitor of the metalloproteinase type IV collagenolytic activity [30] and the protease inhibitor SERPINE2 [31]. The NFASC gene, which promotes axon subcellular targeting and synapse formation [32], was another highly induced gene, suggesting stimulation of neuronal signaling. Two genes are related to stress: the gene encoding the putative glutathione peroxidase GPX8, an enzyme involved in protection against oxidative stress [33], and the DNAJC10/ERDJ5 gene. The PTX3 gene is related to inflammation [34]. Other genes are related to sphingomyelin metabolism such as those encoding: GBA, a lysosomal enzyme that catalyzes glucosylceramide breakdown [35], and SGMS2, an enzyme that produces sphingomyelin [36]. Finally, a group of genes encodes proteins that either have an undefined (i.e. TMEM200A), or an unknown function in the SM tissue (KIAA1199, PSD3, CCDC80/URB/SSG1/DRO1 and LPIN2).

We assessed the changed expression for 17 genes that were selected as follows. Within the 10% most regulated genes, including both downregulated and upregulated groups, we selected genes that were involved in various regulated biological functions (metabolism, muscle-system/contraction and apoptosis), the GIMAP4 family member, the transcription factors MYF6 and HIF1A and other potential regulators such as TOP2A and CCDC80, and THY1. We used the QuantiGene Plex assay to corroborate most of the genes. This assay does not require any RNA amplification, unlike microarrays or quantitative PCR, and is both sensitive and accurate [21]. However, 3 of these genes were validated by the customary, readily accessible RT and real-time PCR technique [17]. Differential expression was confirmed for all of the selected genes (Table 2). Differences in downregulated genes that were assessed with the QuantiGene Plex assay were equivalent to or greater (less than 6-fold) than those assessed with the microarray, as were the differences assessed with RT and real-time PCR (12- or 20-fold greater). In the latter case, increased expression of the B2M control gene in SM cultures compared to SM tissue (Additional file 2, Table S1), which was confirmed by RT real-time PCR, may have contributed to increasing the differences. In contrast, differences in upregulated genes measured with the QuantiGene Plex assay were smaller than those measured with the microarray (1.7- to 7-fold). However, with THY1, the difference assessed by RT and real-time PCR was similar.

**Table 2:** Genes validated for differential expression

Gene ID	Gene Name	Description	FCM	FCV
29765	TMOD4	tropomodulin 4 (muscle)	-1564	-3065*
270	AMPD1	adenosine monophosphate deaminase 1 (isoform M)	-1320	-2799*
4604	MYBPC1	myosin binding protein C, slow type	-1128	-1574*
58529	MYOZ1	myozenin 1	-546.7	-3449*
5837	PYGM	phosphorylase, glycogen; muscle	-277.4	-3444**
4618	MYF6	myogenic factor 6 (herculin)	-265.7	-258.8*
129446	XIRP2	xin actin-binding repeat containing 2	-216.1	-498.5*
825	CAPN3	calpain 3, (p94)	-164.2	-497.9*
1375	CPT1B	carnitine palmitoyltransferase 1B (muscle)	-151.4	-109.5*
7352	UCP3	uncoupling protein 3 (mitochondrial, proton carrier)	-106.5	-2086**
55303	GIMAP4	GTPase, IMAP family member 4	-71.28	-146.3*
7070	THY1	Thy-1 cell surface antigen	+16.67	+18.15*
151887	CCDC80	coiled-coil domain containing 80	+31.02/17.06^D^	+7.04*
3091	HIF1A	hypoxia-inducible factor 1, alpha subunit	+34.89	+19.62*
7153	TOP2A	topoisomerase (DNA) II alpha 170kDa	+43.58	+5.83*
1026	CDKN1A	cyclin-dependent kinase inhibitor 1A (p21, Cip1)	+192.8	+31.67*
57214	KIAA1199	KIAA1199	+1279	+370.6*

List of genes with changed expression as validated by RT and real-time PCR (PYGM, UCP3, THY1) or QuantiGene Plex (the remaining genes). For each targeted gene, the expression was determined relative to the B2M (in RT and real-time PCR) or TFRC (in QuantiGene Plex) genes. Similar data were obtained with QuantiGene Plex when the B2M gene was used as a control (data not shown). For each gene, the fold change in gene expression was calculated between mean values in cultured myotubes versus SM biopsy. The significance of differences was estimated by a paired t-test: *p < 0.01 and **p < 0.005. The first, second and third columns are as in Table 1, the fourth lists the fold change in the microarray (FCM) as in Table 1 and the fifth the fold change in the validation techniques (FCV), with a negative symbol for downregulated genes and a positive symbol for upregulated genes. The Pearson correlation coefficient between FCM and FCV values is 0.684 (including the two copies of the CCDC80 gene).

### Genome mapping

**Figure 1:**
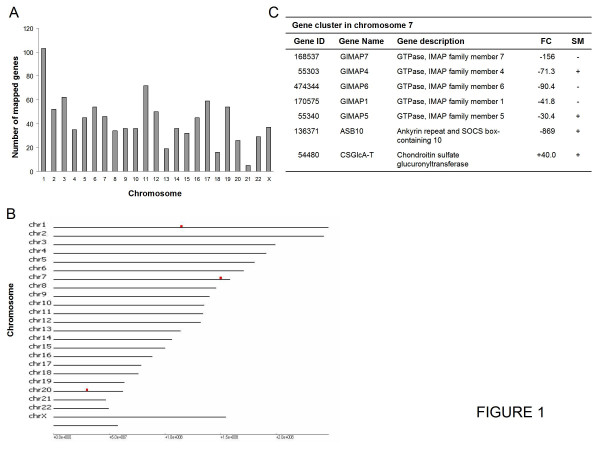
**Chromosome distribution and clusters of enriched regulated genes**. We used the GeneSpring GX program to obtain the chromosome and genomic position of the 1216 differentially expressed genes. Of these, 983 had chromosome annotations. Data were analyzed with the REEF program, with a window width of 1 M, a shift of 200 k, a Q-value of 0.05 and a minimum number of transcripts in clusters of 2. We used the REEF Reference file as reference features. (A) The number of regulated genes is plotted against each annotated chromosome. (B) The image shows the list of chromosomes in the analyzed samples and a representation of the position of clusters of enriched features as red squares. The last line that corresponds to the Y chromosome is not listed. (C) Selected genes in the cluster on chromosome 7 position 149600000 - 151000000 (p-value ≤ 2.117e-004). The first column lists the ID for Entrez Gene, the second column the gene name, the third column the gene description, the fourth column the fold change (FC) with a negative symbol for downregulated genes and a positive symbol for upregulated genes, and the fifth column indicates whether these genes are expressed in SM according to IPA (positive symbol). The cluster on chromosome 1, position 114200000 - 115800000 (p-value ≤ 1.612e-004) was composed of OLFML3, DENND2C, AMPD1, NRAS and CSDE1. The cluster on chromosome 20, position 29400000 - 30600000 (p-value ≤ 9.859e-005) included HM13, COX4I2, MYLK2, C20orf160 and POFUT1.

Since members of the same gene family or genes that are coordinately expressed might form clusters on chromosomes, we tested the possibility that coregulated genes showed spatial clustering. We applied the GeneSpring GX program to the entire set of 1216 differentially expressed genes to identify the chromosome and genomic position. Of these genes, 983 had chromosome annotations that were distributed throughout all chromosomes (Figure 1A), although the highest number was on chromosome 1. We applied the REEF program to the whole set of 983 differentially expressed chromosome-annotated genes to search for local clustering. Three gene clusters were recognized using the REEF program (Figure 1B). The cluster on chromosome 7 (Figure 1C) contained the largest number of genes and included five members of the GTPase of the immunity-associated protein (GIMAP) gene family, plus ASB10 and CSGlcA-T. Except the CSGlcA-T gene, all genes in the cluster are highly downregulated. The genes encoding GIMAP4, GIMAP5, ASB10 and CSGlcA-T are expressed in SM (Additional file 2, Table S1). No gene families were detected in clusters on chromosomes 1 and 20.

### Bioinformatic analysis and interpretation of microarray expression data

**Figure 2:**
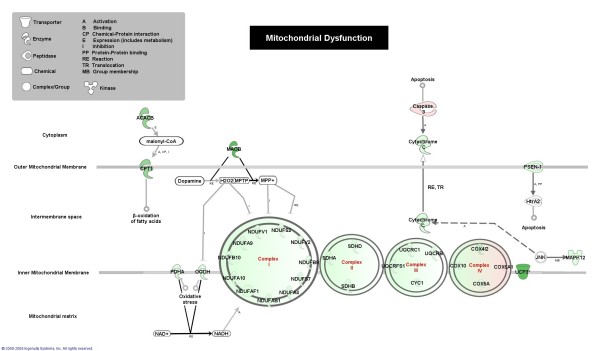
**Mitochondrial dysfunction**. Graphical representation of the differentially expressed genes in cultured myotubes compared to SM tissue and their molecular relationships. Genes are represented as nodes. Node color indicates the direction of change of gene expression. Downregulated genes are highlighted in green and upregulated genes in red. Color intensity corresponds to the magnitude of change. Nodes are displayed using various shapes that represent the functional class of the gene product. The lines in between genes represent known interactions. The pathway image was created using IPA software.

To reveal regulated metabolic and signaling pathways the complete set of differentially expressed genes was analyzed by IPA. According to IPA, 10 signaling and 13 metabolic canonical pathways are regulated in myotubes versus SM tissue (Table 3). The most regulated metabolic pathways are related to mitochondria: the citrate cycle, oxidative phosphorylation and ubiquinone biosynthesis. Consequently, mitochondrial dysfunction is the most regulated signaling pathway. Network analysis of this pathway illustrates known interactions between the identified genes and overall pathway downregulation (Figure 2). Included genes are those encoding: components of the oxidative phosphorylation system (most of which are in complex I, some are components of complexes II, III and IV and cytochrome *c *CYCS); UCP3, an inner mitochondrial membrane transporter that dissipates the proton gradient [37]; CPT1B, which is associated with the outer mitochondrial membrane and facilitates the mitochondrial import of long chain fatty acids, and ACACB, which converts acetylCoA into malonylCoA to inhibit CPT1B [38]; MAOB, an enzyme involved in the degradation of biogenic amines [39]; the pyruvate dehydrogenase E1-alpha subunit (PDHA); and alpha-ketoglutarate dehydrogenase (KGDH). All these genes are expressed in SM except the NDUFAB1, NDUFA4, COX10 and UQCRB genes according to the IPA knowledge-base. Additional regulated metabolic pathways involve glucose, amino acid and monocarboxylic acid metabolism. Results were similar when the subset of SM-expressed genes was analyzed. However, the pathways One carbon pool by folate and Biosynthesis of steroids were not enriched, whereas the pathways Fatty acid metabolism, Phenylalanine metabolism and Glyoxylate, dicarboxylate metabolism were enriched with -log(p-values) of 2.38, 2.13 and 1.81, respectively.

**Table 3:** Significantly regulated canonical pathways

	Score	Ratio	Number of Focus Genes
**Signaling Pathways**			
Mitochondrial Dysfunction	7.88	17.60%	30
Integrin Signaling	3.52	14.50%	28
Hepatic Fibrosis/Hepatic Stellate Cell Activation	2.38	14.10%	19
Caveolar-mediated Endocytosis	2.02	14.80%	12
Regulation of Actin-based Motility by Rho	1.77	13.00%	12
Actin Cytoskeleton Signaling	1.46	10.40%	23
Apoptosis Signaling	1.35	12.10%	11
Circadian Rhythm Signaling	1.31	15.60%	5
p53 Signaling	1.29	12.60%	11
PTEN Signaling	1.29	11.70%	11

**Metabolic Pathways**			
Citrate Cycle	6.27	20.30%	12
Oxidative Phosphorylation	4.61	15.90%	25
Ubiquinone Biosynthesis	3.66	12.50%	13
Glycolysis/Gluconeogenesis	3.39	12.10%	17
Pyruvate Metabolism	3.15	9.66%	14
Tyrosine Metabolism	3.01	6.45%	12
Butanoate Metabolism	2.61	8.53%	11
One Carbon Pool by Folate	2.51	15.80%	6
Glycosaminoglycan Degradation	1.85	9.84%	6
Biosynthesis of Steroids	1.78	4.72%	6
Alanine and Aspartate Metabolism	1.67	9.30%	8
Propanoate Metabolism	1.54	7.14%	9
Lysine Degradation	1.34	6.25%	9

Canonical pathways that have significant associations with the whole set of regulated transcripts were assessed using IPA software. The scores are shown as -log(p-value) in order to show positive numbers. The p-value was calculated by IPA using Fisher's Exact Test. A threshold value of 1.3 was selected (p-value < 0.05). The first column lists the signaling or metabolic pathways; the second the score value; the third the ratio, which represents the proportion of genes from the dataset over the total number of genes in the pathway; and the fourth the number of focus genes.

**Figure 3:**
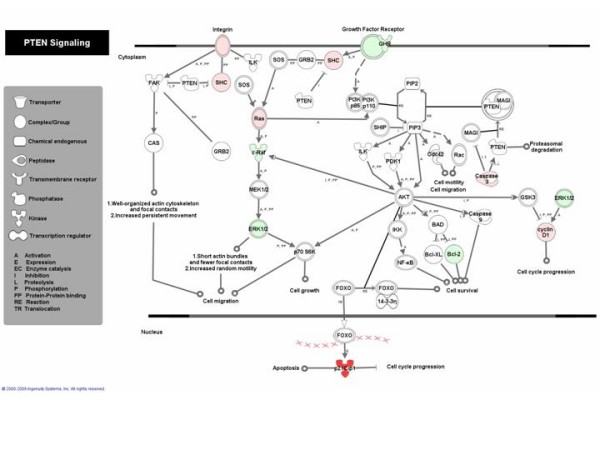
**PTEN signaling pathway**. Graphical representation of the differentially expressed genes in cultured myotubes compared to SM tissue and their molecular relationships. Genes are represented as nodes. Node color indicates the direction of change of gene expression. Downregulated genes are highlighted in green and upregulated genes in red. Color intensity corresponds to the magnitude of change. Nodes are displayed using various shapes that represent the functional class of the gene product. The lines in between genes represent known interactions. The pathway image was created using IPA software.

Other regulated signaling pathways in the complete set of the differentially expressed genes relate to cell interaction with the extracellular matrix (integrin signaling, hepatic fibrosis), cell communication with its environment (caveolar-mediated endocytosis) and actin-cytoskeleton signaling. In addition, circadian rhythm signaling was altered, with five genes all downregulated. Finally, apoptosis and the signaling of phosphatase and tensin homolog (PTEN) (Figure 3) were regulated. PTEN dephosphorylates the signaling lipid phosphatidylinositol (3,4,5)-trisphosphate and affects cellular processes related to cell proliferation, apoptosis and muscle contractility [40]. All signaling pathways except the p53-pathway were significantly and similarly regulated when the subset of SM-expressed genes was analyzed (data not shown).

## Discussion

Using the high-sensitivity Illumina microarray platform, we identified changes in the human SM cell transcriptome that were induced by tissue biopsy culture based on the explant technique. More than one thousand genes displayed differential expression between cultured myotubes and SM tissue. A similar number of genes were upregulated and downregulated: 65% and 50% of the genes in each group, respectively, were expressed in SM according to the IPA knowledge-base.

Ontology analysis of downregulated genes in SM cultures revealed the following. In terms of their associated cellular components, there was an over-representation of genes encoding proteins located in mitochondria. In terms of their associated cell process, the genes that were enriched were involved in energy metabolism and the muscle-system. In line with this, the genes that had the most reduced expression included those encoding key regulators of glycogenolysis (PYGM), fatty acid oxidation (CPT1B) and preservation of ATP formation (AMPD1). Various genes involved in glycolysis, energy consumption (UCP3) and oxidative phosphorylation (complexes I, II, III and IV) were also repressed. IPA pathway analysis consistently revealed that the majority of regulated metabolic genes are involved in mitochondrial pathways of energy production and the most regulated signaling pathway is mitochondrial dysfunction. The highly downregulated genes also included those that encode proteins associated with myofilaments, calcium-binding proteins and CAPN3, a muscle-specific calcium-activated nonlysosomal cysteine protease that contributes to sarcomere homeostasis [27]. Downregulated genes are slow-type isoforms such as MYL3, MYBPC1 and MYOZ1 and isoforms that are predominantly expressed in fast-type muscle fibers such as TMOD4, CASQ1 and MYOZ3. Noteworthy, the lumbar paravertebral muscles in humans are described as containing fiber types, type I (slow) and type II (fast), in similar proportions [41,42]. The downregulation found in the metabolic and muscle-system profile in SM cultures is consistent with findings in several myopathies. For instance, energy metabolism genes were the major group of genes downregulated in human muscle by disuse atrophy [1], DMD [6,7], α-sarcoglycan deficiency [7] and X-linked myotubular myopathy (XLMTM) [9]. Decreased expression of genes encoding muscle-system/contraction associated or regulatory proteins was observed in DMD [6] and XLMTM patients [9]. In muscles of immobilized legs in patients [1], major categories of downregulated genes were involved in sarcomere structure and protein turnover (including the CAPN3 gene). According to our data, the full phenotype of several muscle pathologies may not be expressed in cultured muscle as the genes are markedly downregulated. This includes PYGM-deficiency (McArdle disease) [43]; lack of AMPD1, which causes metabolic myopathy in humans [44]; and loss-of-function mutations in the CAPN3 gene that have been associated with limb-girdle muscular dystrophy type 2A (LGMD2A) [27].

We identified potential regulators of SM function among the most downregulated genes. Myf6/MRF4 is a member of the family of myogenic regulatory factors that includes MyoD, Myf5 and myogenin [45]. MRF4 expression is sufficient for conversion of fibroblasts to myogenic lineage [46]. However, MRF4 did not stimulate myogenic differentiation in C2C12 cells, but cell proliferation, in contrast to MyoD [47]. In addition, it was inefficient at trans-activating contractile protein genes, unlike MyoD and myogenin [46]. Various genes encoding proteins that contain the ankyrin repeat structural motif were strongly downregulated, namely, the ASB family members ASB10 and ASB12. ASBs are implicated in various biological processes. For example, ASB15 promotes muscle cell growth and stimulates protein synthesis [48], while ASB6 regulates components of the insulin signaling pathway in adipocytes [49]. Two members of the MARP family are also included: ANKRD2 and ANKRD23. ANKRD2/Arpp appears to be involved in sensing stress signals and linking these to muscle gene regulation [50], and ANKRD23/DARP is upregulated in insulin-resistant animals [51]. Abrogation of the three MARP family members CARP, ANKRD2 and DARP in mice indicated a role in passive mechanical behavior and in the regenerative response in muscle [52].

In myotubes versus SM tissue, we found coordinated reduced expression of five members of the GIMAP gene family GIMAP1, GIMAP4, GIMAP5, GIMAP6 and GIMAP7, which form a cluster on chromosome 7 and participate in cell survival/death. Seven GIMAP genes are functional: *GIMAP1*, *GIMAP2*, *GIMAP4*, *GIMAP5*, *GIMAP6*, *GIMAP7 *and *GIMAP8 *[53]. The GIMAPS are also called immune-associated nucleotide-binding proteins and are considered cell survival regulators, with a crucial role in lymphocytes: GIMAP5 is a key genetic factor for lymphopenia in spontaneous BioBreeding rat insulin-dependent diabetes and GIMAP4 has been shown to accelerate programmed cell death in T-cells [54]. In humans, the GIMAP family genes are expressed in several tissues [54], although the highest expression of most of these genes is in immune tissues. However, to the best of our knowledge, this is the first description of coordinate regulation of the expression of the genes in this family, which indicates the participation of common mechanisms of transcriptional regulation: shared control regions or the same control elements in each gene.

In contrast, most of the upregulated genes in culture were located in the ER and extracellular matrix on the basis of the gene ontology analysis. Induced genes included those involved in tissue remodeling, such as genes encoding TIMP2, PLOD2 and PLOD3, CSGlcAT, SERPINE2 and MMP14, all of which were among the 10% most regulated. In addition, induced genes included those involved in the formation of the extracellular matrix, such as those encoding collagens, laminins, fibronectin, integrins and proteoglycans. This increase may be partially due to the enrichment of SM cultures in fibroblasts compared to the tissue. This is indicated by the increased expression, albeit less than that of the 10% most regulated, of the THY1 gene, which is expressed in fibroblasts, neurons and endothelial cells. Myotubes may also contribute to this increase in expression. In this regard, IPA analysis revealed alteration of signaling pathways related to cell interaction with the extracellular matrix/environment in either the whole set or the subset of SM-filtered differentially expressed genes. Remarkably, the proteoglycan SPOCK1/SPARC/OSTEONECTIN gene was highly induced. Upregulation of genes of the extracellular matrix are common findings in muscle from DMD [5-7], LGMD2A [8] and XLMTM patients [9], where it is considered to reflect dystrophic changes. In some cases, the SPARC gene is also included [5-7,9]. In our SM cultures, the strong induction of DNAJC10/ERDJ5 gene expression suggests ER stress. DNAJC10/ERDJ5 is abundant in secretory cells and induced during ER stress [55]. It resides in ER, where secreted proteins are translocated and encounter the folding machinery; it has disulfide reductase activity and is involved in the retro-translocation of misfolded proteins into cytosol for degradation [56].

One of the most induced genes in culture is that encoding CDKN1A/p21, an inhibitor of cell cycle progression, involved in the p53-dependent cellular senescence, possibly mediating apoptosis execution [57]. Another highly induced gene encodes KIAA1199, a protein of undefined function that was identified as an induced transcript in replicative senescent-induced renal carcinoma cells by transfer of human chromosome 3 [58], and as a candidate for hearing loss [59]. These data suggest that cultured muscle cells have activated a senescence process that was not established in the muscle tissue. Accordingly, the IPA analysis detected apoptosis as a regulated signaling pathway.

Among the most induced genes in SM cultures were potential transcriptional regulators. These included the gene encoding TOP2A, a DNA topoisomerase that is associated with the RNA polymerase II holoenzyme and is a required component of chromatin-dependent coactivation [60], and the HIF1A/MOP1 gene, which encodes the oxygen-sensitive alpha subunit of the transcription factor hypoxia inducible factor-1 (HIF-1) that forms a heterodimer with the beta subunit [61]. During hypoxia the HIF1A protein is stabilized and activated. The HIF1A gene is expressed constitutively in some cell types, in others it is upregulated by hypoxia [62] or obesity [63], although downregulation of mRNA levels by hypoxia has also been reported in human fat cells [64]. With respect to hypoxia, we found that the *MB *gene encoding the oxygen-binding myoglobin is one of the most repressed in cultured SM cells. Among the targets of HIF-1 [61], several genes showed increased expression in the SM cultures, such as the gene encoding the protein related to vascular tone, ADM; to angiogenesis, VEGFC; to glucose metabolism, ENO1; and the homolog of the pro-apoptotic BNIP3 protein BNIP3L/NIX gene, which suggests increased activity of the HIF-1 transcription factor. However, other HIF-1 gene targets involved in glucose metabolism [61] were not upregulated. The overexpression of HIF1A factor is related to local invasion and metastatic spread of tumor cells [61]. Thus, in SM cultures it may contribute to the activation of the tissue remodeling process. Moreover, the HIF-1 factor has been related [65] to resistance to anoikis, a programmed cell death induced by the loss of or inappropriate cell adhesion, in transformed epithelial cells and it might also have this role in the SM cultures.

Two genes with potential involvement but no clear role in metabolic control also showed increased expression in culture. One is the gene encoding CCDC80/URB/SSG1/DRO1, which was identified as an upregulated transcript in the adipose tissue of bombesin receptor subtype-3 (BRS-3)-deficient mice displaying mild late-onset obesity [66] and sensitizes cells to anoikis and apoptosis [67]. The other is the gene encoding LPIN2, which is a member of the lipin protein family that possesses phosphatidate phosphatase activity converting phosphatidate to diacylglycerol. The function of the latter has not yet been defined and mutations of the gene have been found in patients with Majeed syndrome, an auto-inflammatory disorder [68].

In summary, our data show a reduction in the metabolic and muscle-system transcriptome in the SM cultures that may be due to inappropriate stimuli, including the lack of innervation, the limited extracellular matrix molecules in SM cultures or other less-defined stimuli such as circadian rhythm. The fact that the tissue remodeling transcriptome is increased and signaling pathways related to cell interaction with the extracellular matrix/environment are regulated indicates that a lack of appropriate matrix stimuli may be involved, at least partly. The hypoxia response pathway, which appears to be activated in SM cultures, may to some extent trigger the tissue remodeling process. Finally, the atrophic phenotype of cultured myotubes is associated with the induction of genes related to apoptosis or anoikis and activation of the apoptosis signaling pathway.

## Conclusions

We show that a high-sensitivity microarray platform enables analysis of SM tissue samples from individual donors and derived cultures at whole transcriptome level. Microarrays deliver the differential transcriptome. The main findings are that SM cultures show reductive metabolic and muscle-system transcriptome adaptations as observed in muscle atrophy. The metabolic reduction strongly affects key genes of the catabolism of glucose and lipids and moderately and extensively affects genes involved in mitochondrial energy production. In contrast, SM cultures show augmented tissue remodeling transcriptome and induction of genes involved in the apoptosis or anoikis process. Finally, the hypoxia-response pathway appears to participate in this adaptation. Biological pathway analysis by IPA provides insights into processes driven by the culturing of SM such as mitochondrial dysfunction and pathways related to cell interaction with the extracellular matrix and apoptosis. This study contributes to the definition of the phenotype of the SM primary culture, which is a valuable cell model that has key implications for the study of muscle pathogenesis and therapeutic assays.

## Authors' contributions

FR contributed to statistical analysis, validation of gene expression, pathway analysis and manuscript drafting. SM contributed to microarray sample processing and validation of gene expression analysis. MK contributed to study design and manuscript drafting. JC and AN contributed to conception of the study and sample recruitment. EM contributed to sample processing and validation of gene expression analysis. CGM contributed to conception and design of the study and SM cultures. AMGF contributed to conception and design of the study, data analysis and manuscript drafting. All authors have read and approved the final manuscript.

## Supplementary Material

Additional file 1 **Figure S1. Immunostaining of muscle cultures with the muscle-specific marker desmin**. Cultured cells were immunostained with desmin antibody. Then, to highlight nuclei, cells were stained with Hoescht. Representative immunofluorescence micrographs of SM cultures (A) B19, (B) B22, (C) B24, (D) B25 and (E) B26 are shown. Bar represents 50 μm. Most of the Hoescht-stained nuclei are observed in desmin-labeled myotubes. Click here for file

Additional file 2 **Table S1. Complete list of differentially expressed genes after skeletal muscle culture**. List of the complete set of genes with differential expression in cultured myotubes compared to SM tissue. Replicated genes, which occur more than once in the microarray, were filtered and the first one appearing in the list was arbitrarily chosen. For each gene, the fold change in gene expression was calculated as the ratio between mean values in cultured myotubes versus SM biopsy. The significance of differences was estimated by a Benjamini-Hochberg corrected paired t-test (p < 0.01). The first column lists the ID for Entrez Gene, the second column the gene name, the third column the description of the gene, the fourth column the quality of expression (1) or not (0) in skeletal muscle tissue (M), according to the IPA knowledge-base, and the fifth column the fold change (FC) with a negative symbol for downregulated genes and no symbol for upregulated genes. Click here for file

Additional file 3 **Figure S2**. **Cluster and correlation analysis for microarray data**. A) Principal component analysis plot: X = principal component 1, Y = principal component 2 and Z = principal component 3. The percentage of total variance that each principal component captures is 95.9% for component 1, 2.15% for component 2 and 1.90% for component 3. B) Absolute correlation dendrogram. C) Pearson correlation matrix of all samples based on whole gene expression profiles. D) Pearson correlation coefficients between all samples. A value of 1 would mean a perfect correlation. In panels A and C, the samples from the cultured myotube group (*in vitro*) are represented in red and the samples from the SM tissue biopsies (*in vivo*) are represented in blue. Click here for file

Additional file 4 **Table S2. Most significantly regulated gene ontologies**. List of gene ontologies annotated from (A) the whole set or (B) the subset filtered by expression in SM, on the basis of the IPA knowledge-base, of downregulated and upregulated transcripts in cultured myotubes compared to the SM tissue. We used the GO database http://www.geneontology.org with the GeneSpring GX software. GeneSpring GX calculated enrichment scores for GO terms based on the list of regulated genes, and used enrichment scores and Benjamini-Yekutieli (False Discovery Rate) corrected p-values to filter the set of genes. GO terms that are enriched with a p-value cut-off of 0.1 are shown. Less specific nodes in the GO hierarchy that contained the same annotated genes as the stated most-specific nodes are not shown. The first column lists the GO symbol, the second the GO term, the third the corrected p-value and the fourth the count in the selection. Click here for file

Additional file 5 **Table S3. Microarray data variability of the 10% most skeletal muscle-filtered differentially expressed transcripts**. Log2 normalized intensities for each significantly regulated transcript (p < 0.01 and fold change > 2) in each sample. The standard deviation for each condition, *in vitro *and *in vivo*, is shown. A normalized intensity value of 0 means that the raw signal was below the background and an arbitrary value of 1 was assigned to enable the log2 transformation (log2(1) = 0). Click here for file

## References

[B1] Chen YW Gregory CM Scarborough MT Shi R Walter GA Vandenborne K Transcriptional pathways associated with skeletal muscle disuse atrophy in humans Physiol Genomics 2007 31 510 520 10.1152/physiolgenomics.00115.2006 17804603

[B2] Zhou X Dimachkie MM Xiong M Tan FK Arnett FC cDNA microarrays reveal distinct gene expression clusters in idiopathic inflammatory myopathies Med Sci Monit 2004 10 BR191 BR197 15232492

[B3] Greenberg SA A gene expression approach to study perturbed pathways in myositis Curr Opin Rheumatol 2007 19 536 541 10.1097/BOR.0b013e3282efe261 17917532

[B4] Bakay M Zhao P Chen J Hoffman EP A web-accessible complete transcriptome of normal human and DMD muscle Neuromuscul Disord 2002 12 Suppl 1 S125 S141 10.1016/S0960-8966(02)00093-7 12206807

[B5] Haslett JN Sanoudou D Kho AT Bennett RR Greenberg SA Kohane IS Beggs AH Kunkel LM Gene expression comparison of biopsies from Duchenne muscular dystrophy (DMD) and normal skeletal muscle Proc Natl Acad Sci USA 2002 99 15000 15005 10.1073/pnas.192571199 12415109 PMC137534

[B6] Noguchi S Tsukahara T Fujita M Kurokawa R Tachikawa M Toda T Tsujimoto A Arahata K Nishino I cDNA microarray analysis of individual Duchenne muscular dystrophy patients Hum Mol Genet 2003 12 595 600 10.1093/hmg/12.6.595 12620965

[B7] Chen YW Zhao P Borup R Hoffman EP Expression profiling in the muscular dystrophies: identification of novel aspects of molecular pathophysiology J Cell Biol 2000 151 1321 1336 10.1083/jcb.151.6.1321 11121445 PMC2190600

[B8] Keira Y Noguchi S Kurokawa R Fujita M Minami N Hayashi YK Kato T Nishino I Characterization of lobulated fibers in limb girdle muscular dystrophy type 2A by gene expression profiling Neurosci Res 2007 57 513 521 10.1016/j.neures.2006.12.010 17258832

[B9] Noguchi S Fujita M Murayama K Kurokawa R Nishino I Gene expression analyses in X-linked myotubular myopathy Neurology 2005 65 732 737 10.1212/01.wnl.0000174625.67484.4d 16157907

[B10] Nygaard V Hovig E Options available for profiling small samples: a review of sample amplification technology when combined with microarray profiling Nucleic Acids Res 2006 34 996 1014 10.1093/nar/gkj499 16473852 PMC1363777

[B11] Askanas V Gallez-Hawkins G Synergistic influence of polypeptide growth factors on cultured human muscle Arch Neurol 1985 42 749 752 3896205 10.1001/archneur.1985.04210090013004

[B12] Zammit PS Partridge TA Yablonka-Reuveni Z The skeletal muscle satellite cell: the stem cell that came in from the cold J Histochem Cytochem 2006 54 1177 1191 10.1369/jhc.6R6995.2006 16899758

[B13] Bonavaud S Agbulut O Nizard R D'honneur G Mouly V Butler-Browne G A discrepancy resolved: human satellite cells are not preprogrammed to fast and slow lineages Neuromuscul Disord 2001 11 747 752 10.1016/S0960-8966(01)00222-X 11595517

[B14] Mouly V Edom F Barbet JP Butler-Browne GS Plasticity of human satellite cells Neuromuscul Disord 1993 3 371 377 10.1016/0960-8966(93)90080-4 8186678

[B15] lannaccone ST Nagy B Samaha FJ Partial biochemical maturation of aneurally cultured human skeletal muscle Neurology 1982 32 846 851 7201579 10.1212/wnl.32.8.846

[B16] Martinuzzi A Askanas V Kobayashi T Engel WK Di Mauro S Expression of muscle-gene-specific isozymes of phosphorylase and creatine kinase in innervated cultured human muscle J Cell Biol 1986 103 1423 1429 10.1083/jcb.103.4.1423 3771644 PMC2114337

[B17] Ferrer-Martínez A Montell E Montori-Grau M García-Martínez C Gómez-Foix AM Roberts MA Mansourian R Macé K Long-term cultured human myotubes decrease contractile gene expression and regulate apoptosis-related genes Gene 2006 384 145 153 10.1016/j.gene.2006.07.042 17052863

[B18] Van Gelder RN von Zastrow ME Yool A Dement WC Barchas JD Eberwine JH Amplified RNA synthesized from limited quantities of heterogeneous cDNA Proc Natl Acad Sci USA 1990 87 1663 1667 10.1073/pnas.87.5.1663 1689846 PMC53542

[B19] Raymond F Metairon S Borner R Hofmann M Kussmann M Automated target preparation for microarray-based gene expression analysis Anal Chem 2006 78 6299 6305 10.1021/ac060097t 16970301

[B20] Verdugo RA Deschepper CF Muñoz G Pomp D Churchill GA Importance of randomization in microarray experimental designs with Illumina platforms Nucleic Acids Res 2009 37 5610 5618 10.1093/nar/gkp573 19617374 PMC2761262

[B21] Canales RD Luo Y Willey JC Austermiller B Barbacioru CC Boysen C Hunkapiller K Jensen RV Knight CR Lee KY Ma Y Maqsodi B Papallo A Peters EH Poulter K Ruppel PL Samaha RR Shi L Yang W Zhang L Goodsaid FM Evaluation of DNA microarray results with quantitative gene expression platforms Nature Biotechnology 2006 24 1115 1122 10.1038/nbt1236 16964225

[B22] Coppe A Danieli GA Bortoluzzi S REEF: searching REgionally Enriched Features in genomes BMC Bioinformatics 2006 7 453 10.1186/1471-2105-7-453 17042935 PMC1624853

[B23] Paulin D Li Z Desmin: a major intermediate filament protein essential for the structural integrity and function of muscle Exp Cell Res 2004 301 1 7 10.1016/j.yexcr.2004.08.004 15501438

[B24] Su AI Wiltshire T Batalov S Lapp H Ching KA Block D Zhang J Soden R Hayakawa M Kreiman G Cooke MP Walker JR Hogenesch JB A gene atlas of the mouse and human protein-encoding transcriptomes Proc Natl Acad Sci USA 2004 101 6062 6067 10.1073/pnas.0400782101 15075390 PMC395923

[B25] Hardison R Hemoglobins from bacteria to man: evolution of different patterns of gene expression J Exp Biol 1998 201 1099 1117 9510523 10.1242/jeb.201.8.1099

[B26] Rege TA Hagood JS Thy-1 as a regulator of cell-cell and cell-matrix interactions in axon regeneration, apoptosis, adhesion, migration, cancer, and fibrosis FASEB J 2006 20 1045 1054 10.1096/fj.05-5460rev 16770003

[B27] Zatz M Starling A Calpains and disease N Engl J Med 2005 352 2413 2423 10.1056/NEJMra043361 15944426

[B28] Myllylä R Wang C Heikkinen J Juffer A Lampela O Risteli M Ruotsalainen H Salo A Sipilä L Expanding the lysyl hydroxylase toolbox: new insights into the localization and activities of lysyl hydroxylase 3 (LH3) J Cell Physiol 2007 212 323 329 10.1002/jcp.21036 17516569

[B29] Gotoh M Yada T Sato T Akashima T Iwasaki H Mochizuki H Inaba N Togayachi A Kudo T Watanabe H Kimata K Narimatsu H Molecular cloning and characterization of a novel chondroitin sulfate glucuronyltransferase that transfers glucuronic acid to N-acetylgalactosamine J Biol Chem 2002 277 38179 38188 10.1074/jbc.M202601200 12145278

[B30] Stetler-Stevenson WG Krutzsch HC Liotta LA Tissue inhibitor of metalloproteinase (TIMP-2). A new member of the metalloproteinase inhibitor family J Biol Chem 1989 264 17374 17378 2793861

[B31] Carter RE Cerosaletti KM Burkin DJ Fournier RE Jones C Greenberg BD Citron BA Festoff BW The gene for the serpin thrombin inhibitor (PI7), protease nexin I, is located on human chromosome 2q33-q35 and on syntenic regions in the mouse and sheep genomes Genomics 1995 27 196 199 10.1006/geno.1995.1025 7665170

[B32] Ango F di Cristo G Higashiyama H Bennett V Wu P Huang ZJ Ankyrin-based subcellular gradient of neurofascin, an immunoglobulin family protein, directs GABAergic innervation at Purkinje axon initial segment Cell 2004 119 257 272 10.1016/j.cell.2004.10.004 15479642

[B33] Margis R Dunand C Teixeira FK Margis-Pinheiro M Glutathione peroxidase family - an evolutionary overview FEBS J 2008 275 3959 3970 10.1111/j.1742-4658.2008.06542.x 18616466

[B34] Bottazzi B Garlanda C Cotena A Moalli F Jaillon S Deban L Mantovani A The long pentraxin PTX3 as a prototypic humoral pattern recognition receptor: interplay with cellular innate immunity Immunol Rev 2009 227 9 18 10.1111/j.1600-065X.2008.00719.x 19120471

[B35] Beutler E Gaucher disease: new molecular approaches to diagnosis and treatment Science 1992 256 794 799 10.1126/science.1589760 1589760

[B36] Huitema K Dikkenberg J van den Brouwers JFHM Holthuis JCM Identification of a family of animal sphingomyelin synthases EMBO J 2004 23 33 44 10.1038/sj.emboj.7600034 14685263 PMC1271672

[B37] Dalgaard LT Pedersen O Uncoupling proteins: functional characteristics and role in the pathogenesis of obesity and Type II diabetes Diabetologia 2001 44 946 965 10.1007/s001250100596 11484071

[B38] Ruderman NB Saha AK Vavvas D Witters LA Malonyl-CoA, fuel sensing, and insulin resistance Am J Physiol 1999 276 E1 E18 9886945 10.1152/ajpendo.1999.276.1.E1

[B39] Lenders JW Eisenhofer G Abeling NG Berger W Murphy DL Konings CH Wagemakers LM Kopin IJ Karoum F van Gennip AH Brunner HG Specific genetic deficiencies of the A and B isoenzymes of monoamine oxidase are characterized by distinct neurochemical and clinical phenotypes J Clin Invest 1996 97 1010 1019 10.1172/JCI118492 8613523 PMC507147

[B40] Keniry M Parsons R The role of PTEN signaling perturbations in cancer and in targeted therapy Oncogene 2008 27 5477 5485 10.1038/onc.2008.248 18794882

[B41] Sirca A Kostevc V The fibre type composition of thoracic and lumbar paravertebral muscles in man J Anat 1985 141 131 137 2934358 PMC1166395

[B42] Thorstensson A Carlson H Fibre types in human lumbar back muscles Acta Physiol Scand 1987 131 195 202 10.1111/j.1748-1716.1987.tb08226.x 2960128

[B43] Lucia A Nogales-Gadea G Pérez M Martín MA Andreu AL Arenas J McArdle disease: what do neurologists need to know? Nat Clin Pract Neurol 2008 4 568 577 10.1038/ncpneuro0913 18833216

[B44] Gross M Clinical heterogeneity and molecular mechanisms in inborn muscle AMP deaminase deficiency J Inherit Metab Dis 1997 20 186 192 10.1023/A:1005352605421 9211191

[B45] Pownall ME Gustafsson MK Emerson CP Jr Myogenic regulatory factors and the specification of muscle progenitors in vertebrate embryos Annu Rev Cell Dev Biol 2002 18 747 783 10.1146/annurev.cellbio.18.012502.105758 12142270

[B46] Yutzey KE Rhodes SJ Konieczny SF Differential trans activation associated with the muscle regulatory factors MyoD1, myogenin, and MRF4 Mol Cell Biol 1990 10 3934 3944 1695319 10.1128/mcb.10.8.3934-3944.1990 PMC360904

[B47] Jin X Kim JG Oh MJ Oh HY Sohn YW Pian X Yin JL Beck S Lee N Son J Kim H Yan C Wang JH Choi YJ Whang KY Opposite roles of MRF4 and MyoD in cell proliferation and myogenic differentiation Biochem Biophys Res Commun 2007 364 476 482 10.1016/j.bbrc.2007.10.042 17959144

[B48] McDaneld TG Hannon K Moody DE Ankyrin repeat and SOCS box protein 15 regulates protein synthesis in skeletal muscle Am J Physiol Regul Integr Comp Physiol 2006 290 R1672 R1682 16424087 10.1152/ajpregu.00239.2005

[B49] Wilcox A Katsanakis KD Bheda F Pillay TS Asb6, an adipocyte-specific ankyrin and SOCS box protein, interacts with APS to enable recruitment of elongins B and C to the insulin receptor signaling complex J Biol Chem 2004 279 38881 38888 10.1074/jbc.M406101200 15231829

[B50] Kojic S Medeot E Guccione E Krmac H Zara I Martinelli V Valle G Faulkner G The Ankrd2 protein, a link between the sarcomere and the nucleus in skeletal muscle J Mol Biol 2004 339 313 325 10.1016/j.jmb.2004.03.071 15136035

[B51] Ikeda K Emoto N Matsuo M Yokoyama M Molecular identification and characterization of a novel nuclear protein whose expression is up-regulated in insulin-resistant animals J Biol Chem 2003 278 3514 3520 10.1074/jbc.M204563200 12456686

[B52] Barash IA Bang ML Mathew L Greaser ML Chen J Lieber RL Structural and regulatory roles of muscle ankyrin repeat protein family in skeletal muscle Am J Physiol Cell Physiol 2007 293 C218 C227 10.1152/ajpcell.00055.2007 17392382

[B53] Krücken J Schroetel RM Müller IU Saïdani N Marinovski P Benten WP Stamm O Wunderlich F Comparative analysis of the human gimap gene cluster encoding a novel GTPase family Gene 2004 341 291 304 10.1016/j.gene.2004.07.005 15474311

[B54] Nitta T Takahama Y The lymphocyte guard-IANs: regulation of lymphocyte survival by IAN/GIMAP family proteins Trends Immunol 2007 28 58 65 10.1016/j.it.2006.12.002 17196432

[B55] Cunnea PM Miranda-Vizuete A Bertoli G Simmen T Damdimopoulos AE Hermann S Leinonen S Huikko MP Gustafsson JA Sitia R Spyrou G ERdj5, an endoplasmic reticulum (ER)-resident protein containing DnaJ and thioredoxin domains, is expressed in secretory cells or following ER stress J Biol Chem 2003 278 1059 1066 10.1074/jbc.M206995200 12411443

[B56] Ushioda R Hoseki J Araki K Jansen G Thomas DY Nagata K ERdj5 is required as a disulfide reductase for degradation of misfolded proteins in the ER Science 2008 321 569 572 10.1126/science.1159293 18653895

[B57] Zhang H Molecular signaling and genetic pathways of senescence: Its role in tumorigenesis and aging J Cell Physiol 2007 210 567 574 10.1002/jcp.20919 17133363

[B58] Michishita E Garcés G Barrett JC Horikawa I Upregulation of the KIAA1199 gene is associated with cellular mortality Cancer Lett 2006 239 71 77 10.1016/j.canlet.2005.07.028 16157444

[B59] Abe S Usami S Nakamura Y Mutations in the gene encoding KIAA1199 protein, an inner-ear protein expressed in Deiters' cells and the fibrocytes, as the cause of non-syndromic hearing loss J Hum Genet 2003 48 564 570 10.1007/s10038-003-0079-2 14577002

[B60] Mondal N Parvin JD DNA topoisomerase II-alpha is required for RNA polymerase II transcription on chromatin templates Nature 2001 413 435 438 10.1038/35096590 11574892

[B61] Ke Q Costa M Hypoxia-inducible factor-1 (HIF-1) Mol Pharmacol 2006 70 1469 1480 10.1124/mol.106.027029 16887934

[B62] Wiener CM Booth G Semenza GL In vivo expression of mRNAs encoding hypoxia-inducible factor 1 Biochem Biophys Res Commun 1996 225 485 488 10.1006/bbrc.1996.1199 8753788

[B63] Cancello R Henegar C Viguerie N Taleb S Poitou C Rouault C Coupaye M Pelloux V Hugol D Bouillot JL Bouloumié A Barbatelli G Cinti S Svensson PA Barsh GS Zucker JD Basdevant A Langin D Clément K Reduction of macrophage infiltration and chemoattractant gene expression changes in white adipose tissue of morbidly obese subjects after surgery-induced weight loss Diabetes 2005 54 2277 2286 10.2337/diabetes.54.8.2277 16046292

[B64] Wang B Wood IS Trayhurn P Dysregulation of the expression and secretion of inflammation-related adipokines by hypoxia in human adipocytes Pflügers Archiv Eur J Physiol 2007 455 479 492 10.1007/s00424-007-0301-8 PMC2040175 17609976

[B65] Rohwer N Welzel M Daskalow K Pfander D Wiedenmann B Detjen K Cramer T Hypoxia-inducible factor 1alpha mediates anoikis resistance via suppression of alpha5 integrin Cancer Res 2008 68 10113 10120 10.1158/0008-5472.CAN-08-1839 19074877

[B66] Aoki K Sun YJ Aoki S Wada K Wada E Cloning, expression, and mapping of a gene that is upregulated in adipose tissue of mice deficient in bombesin receptor subtype-3 Biochem Biophys Res Commun 2002 290 1282 1288 10.1006/bbrc.2002.6337 11812002

[B67] Bommer GT Jäger C Dürr EM Baehs S Eichhorst ST Brabletz T Hu G Fröhlich T Arnold G Kress DC Göke B Fearon ER Kolligs FT DRO1, a gene down-regulated by oncogenes, mediates growth inhibition in colon and pancreatic cancer cells J Biol Chem 2005 280 7962 7975 10.1074/jbc.M412593200 15563452

[B68] Al-Mosawi ZS Al-Saad KK Ijadi-Maghsoodi R El-Shanti HI Ferguson PJ A splice site mutation confirms the role of LPIN2 in Majeed syndrome Arthritis Rheum 2007 56 960 964 10.1002/art.22431 17330256

