# Supplementary figures and images for: Comparative gene expression profiling between human cultured myotubes and skeletal muscle tissue
Source: BMC Genomics. 2010 Feb 22;11:125. doi: 10.1186/1471-2164-11-125 (PMC2838843; doi:10.1186/1471-2164-11-125)

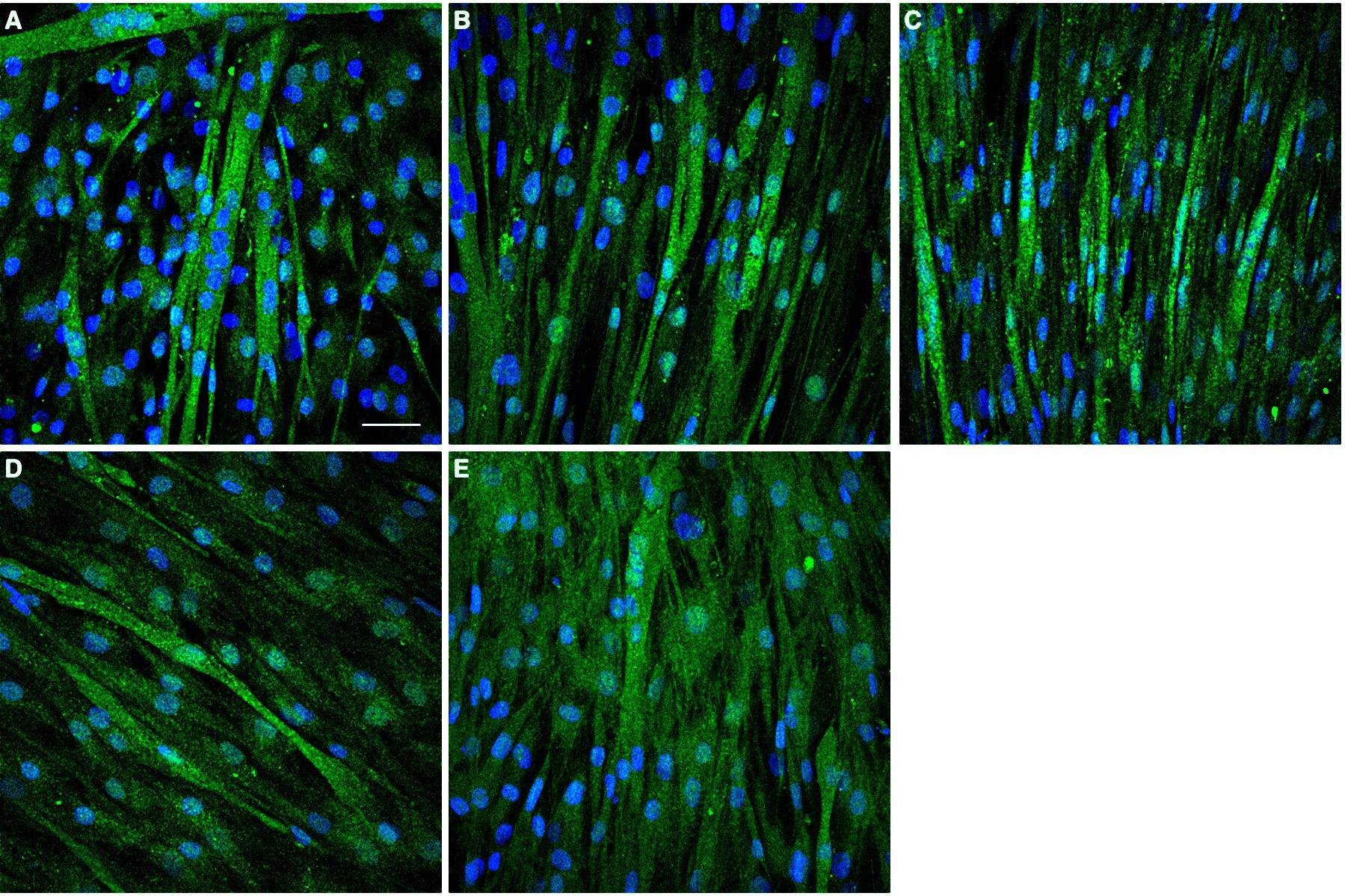

Supplement: Additional file 1 — Figure S1. Immunostaining of muscle cultures with the muscle-specific marker desmin. Cultured cells were immunostained with desmin antibody. Then, to highlight nuclei, cells were stained with Hoescht. Representative immunofluorescence micrographs of SM cultures (A) B19, (B) B22, (C) B24, (D) B25 and (E) B26 are shown. Bar represents 50 μm. Most of the Hoescht-stained nuclei are observed in desmin-labeled myotubes. [file 1471-2164-11-125-S1.JPEG]

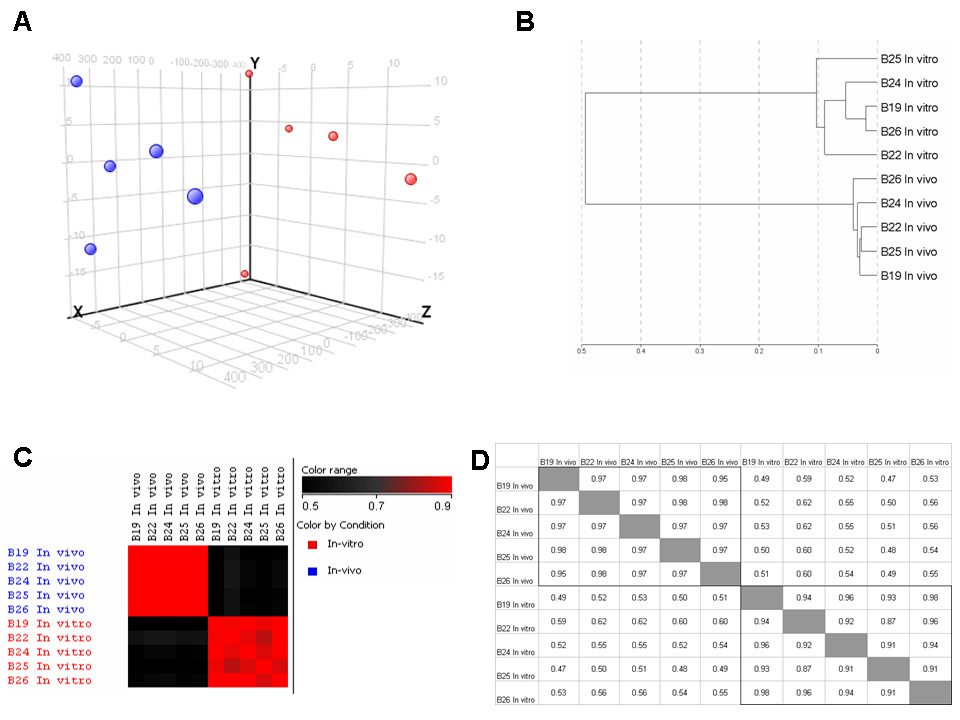

Supplement: Additional file 3 — Figure S2. Cluster and correlation analysis for microarray data. A) Principal component analysis plot: X = principal component 1, Y = principal component 2 and Z = principal component 3. The percentage of total variance that each principal component captures is 95.9% for component 1, 2.15% for component 2 and 1.90% for component 3. B) Absolute correlation dendrogram. C) Pearson correlation matrix of all samples based on whole gene expression profiles. D) Pearson correlation coefficients between all samples. A value of 1 would mean a perfect correlation. In panels A and C, the samples from the cultured myotube group (in vitro) are represented in red and the samples from the SM tissue biopsies (in vivo) are represented in blue. [file 1471-2164-11-125-S3.JPEG]
