# Supplementary material for: Comparative gene expression profiling between human cultured myotubes and skeletal muscle tissue
Source: BMC Genomics. 2010 Feb 22;11:125. doi: 10.1186/1471-2164-11-125 (PMC2838843; doi:10.1186/1471-2164-11-125)
Supplement: Additional file 2 — Table S1. Complete list of differentially expressed genes after skeletal muscle culture. List of the complete set of genes with differential expression in cultured myotubes compared to SM tissue. Replicated genes, which occur more than once in the microarray, were filtered and the first one appearing in the list was arbitrarily chosen. For each gene, the fold change in gene expression was calculated as the ratio between mean values in cultured myotubes versus SM biopsy. The significance of differences was estimated by a Benjamini-Hochberg corrected paired t-test (p < 0.01). The first column lists the ID for Entrez Gene, the second column the gene name, the third column the description of the gene, the fourth column the quality of expression (1) or not (0) in skeletal muscle tissue (M), according to the IPA knowledge-base, and the fifth column the fold change (FC) with a negative symbol for downregulated genes and no symbol for upregulated genes. [file 1471-2164-11-125-S2.DOC]

| **Gene ID** | **Gene Name** | **Description** | | **M** | **FC** |
| --- | --- | --- | --- | --- | --- |
| 3043  4151  761  128826  29765  4634  844  270  4604  136371  91977  3040  4023  6542  58529  53405  253017  91734  6271  342527  57467  57142  6123  153201  200539  4892  123722  760  492307  5837  84448  4618  142689  339456  129446  26287  712  10840  166348  117247  9452  5104  825  4653  55335  361  4129  168537  118461  1375  2532  349236  51285  3743  8490  713  187  81786  5588  122622  7704  57110  257240  3158  1271  286006  57644  7352  22918  762  1149  202333  11156  9413  27231  2167  338328  1003  7069  5207  53346  124  9619  197257  474344  83483  2875  4004  2819  6359  84417  3177  419  161247  2104  4629  55303  9666  3767  137735  11170  6035  92162  6330  126  140706  1511  23224  64231  53353  4222  51705  9547  84701  199  1436  5350  491  133688  7306  10991  56997  157310  170575  167838  284723  79041  1602  51085  284403  3741  388531  3373  2719  10611  56704  6258  65268  55930  84812  5348  349565  90139  139221  83595  64757  123591  123624  85366  53342  55340  23034  8452  29931  25780  113622  2701  51807  388698  3249  92241  54855  2350  50486  6324  152273  26112  5187  57462  171425  137872  400169  51751  32  3952  79365  84215  58528  22849  26051  116441  488  2275  64793  622  8975  333926  7957  163732  147495  7942  9149  59285  81563  23037  132228  6920  7644  84696  84532  10261  11251  3613  2205  91807  1942  4005  596  29763  9535  2099  93058  375775  6327  5213  6300  286  2982  2327  5569  117248  23171  148345  8292  26468  2593  9806  6529  286336  6640  55827  2690  55527  28992  2289  4137  952  34  85457  120425  2872  54538  10579  55208  367  79944  481  58480  91689  64359  23492  N/A  54777  2039  6470  23368  516  50  338  51076  441549  2631  4967  64328  85464  128977  3937  27244  9882  84706  10875  8987  23461  84814  2273  5523  6249  25849  254552  114907  444  348980  23382  4302  1408  5175  2806  8605  9332  6691  29116  10749  2948  25822  374291  6376  317649  1891  3033  26074  340719  11138  157567  64174  10610  54438  56203  2287  5116  7832  63891  89801  154  84988  28971  5264  11059  84263  206338  51805  9322  55268  9465  55031  2101  N/A  11019  202018  51676  9670  593  7326  10229  8402  9148  5212  1737  55902  285193  6495  11309  38  6389  3045  84331  5500  3936  9927  128346  10140  9958  4501  1352  84881  27124  2180  10299  6535  1632  57017  8803  1845  151230  79699  54205  7384  6256  55843  9457  151473  3021  23095  10422  4286  114876  8925  89853  4720  217  7812  353116  7629  9879  2946  513  29780  10939  83887  64771  7381  23543  5886  4715  138716  80142  130502  5160  51478  23408  10131  6487  51069  9267  58526  8050  10904  27245  8864  8065  5532  91647  221545  9513  85476  7075  3632  64208  8214  4729  687  51004  83479  7360  54963  7259  7871  51277  90135  166  6777  55968  49855  97  1431  1537  54927  9553  84461  79724  827  124808  57158  4704  6392  7419  80344  23731  4716  23399  9862  7678  27109  64978  115201  5504  2314  55605  4717  3298  79135  8226  51455  79133  10241  5141  4705  163259  51295  112869  4723  26273  22982  119559  8997  9572  9377  84418  51074  84991  1730  369  7507  55662  4191  5894  23401  137682  9617  84193  79643  6390  9817  5576  25813  55684  8531  129868  88523  51117  28957  55847  26589  55168  89882  91369  64710  23248  3766  116138  26154  51340  79671  220929  64965  64100  7386  100036519  51204  26073  26060  3895  84306  28955  8648  2271  8805  10560  54826  10284  25948  4209  10989  51103  118472  10113  8785  64960  23199  5663  4802  3420  3903  51  3191  253959  4697  830  222484  51081  55074  55833  51247  55353  64288  5683  5018  5245  5108  51002  89910  65008  79731  4706  59343  5860  51503  5310  27183  54948  4154  23353  9491  57533  55066  7411  91661  83460  7067  10455  55831  64951  10240  51138  219931  10300  10055  6731  261726  51504  27236  10807  1186  5437  103910  57038  10126  440589  7511  136319  6206  11234  56926  2967  5936  9950  113201  10810  1495  114294  10134  8099  80025  55898  5531  26009  79833  64430  84216  51177  152007  113612  126792  148022  103  9403  57136  10552  51131  6451  51228  10016  140710  7329  9409  79685  54499  6789  8733  79042  56889  51272  1175  567  8896  23187  27095  347734  6223  133619  10606  10897  25829  51122  6175  1603  26151  353  6890  2782  6301  23509  22872  8826  9948  86  93487  55505  284119  257364  1069  28988  81614  2794  91283  84817  10269  65981  4833  219285  5898  81542  55154  122769  8992  7769  88455  57631  22820  706  5621  8624  1312  3275  2017  3984  4201  25  81555  348262  23301  56983  1203  92703  125144  56986  8546  7041  6184  9204  1019  10801  6873  2734  10195  650152  284131  4758  10548  64778  57414  377  2799  4637  65264  84918  975  26472  27044  4240  50814  8772  64766  132299  79673  1635  292  293  2615  7132  3916  57555  9519  11183  8655  56952  10613  60  1627  10226  10175  29902  1211  171568  10581  23471  3688  7325  51065  8560  114882  9019  6836  64747  55577  10630  4597  3566  26503  10169  1915  2132  727761  8565  85012  8683  25996  64834  7289  5361  219541  1650  56829  5589  10567  10227  23324  2764  201266  57542  84282  145389  79053  26271  51309  80184  4000  245812  81537  26020  1337  23753  84798  10765  90488  10330  81037  6472  3915  80127  29957  4218  10410  4925  3927  977  91012  10092  1265  23641  836  558  51715  29940  51150  8910  9500  2633  3156  85360  483  6745  6122  389  665  1072  731049  55603  4267  9094  54918  23214  7184  3685  89796  2961  55920  3703  59342  1973  79759  5034  10159  254359  51000  55323  4282  5238  29927  8460  26511  57616  10577  55355  3074  57228  822  9181  51524  967  4055  6237  3912  26090  7050  9213  27166  715  53340  8682  5834  23275  6748  27436  3309  7205  6809  139886  378  285362  255758  55013  50854  6464  148867  11167  11135  1281  29922  26022  1292  10525  6147  2534  2896  8829  9823  10457  8511  25941  391  54732  10346  57106  7791  10280  7873  84720  124044  9168  6574  11078  55832  8836  4830  837  63910  2023  30851  4281  9276  23293  4893  2121  47  441531  64924  201895  3428  9134  9961  55106  79143  28978  6624  5954  25796  6498  800  475  118429  1293  26123  6618  11031  10733  85236  813  8651  55757  4650  30008  11014  7112  2246  147179  9601  55742  5476  3920  9022  1611  9780  27018  6185  10970  55170  9638  6876  81567  85363  9942  79001  3706  83982  54879  84790  1728  11216  27346  93109  84068  7107  26272  9949  10549  1278  85365  23643  1021  3710  56944  649  2512  3693  1601  1291  2146  83871  4192  2131  9118  8767  133  3161  11164  3614  11072  375444  3678  6491  6536  2297  7086  9540  10981  6307  83442  58505  11332  10130  64838  5479  79586  10026  128434  302  84513  133308  1893  54839  11147  131566  4666  64855  51291  8714  4131  150223  30850  285590  127700  411  1803  5002  64393  84264  4644  51596  10478  22891  6282  27286  84930  79600  55300  56005  79581  10954  121457  1289  5654  25870  26227  60681  80329  25999  79019  337876  4016  25809  1462  11010  57124  54885  23541  2539  25797  8372  6591  8795  1039  122616  9015  57333  79924  22936  25923  595  5328  126969  6541  871  2191  64175  7993  7070  11015  5611  7045  151887  9697  55612  25758  200894  8317  55357  8974  55276  92799  80067  6772  9363  9319  1009  55361  8382  91373  8532  1029  7424  51303  6499  81502  7058  166929  51148  4643  7421  214  2629  6695  219902  54431  5270  57168  23423  55165  5352  6335  1277  5271  84695  60559  23114  80003  11057  25902  26575  771  55653  26263  4323  400406  7076  494514  8572  4861  115908  79695  3091  493869  80210  623  338773  54480  2588  9663  6505  10643  8985  2335  7153  26002  85016  79070  284217  4237  7162  9023  5754  11082  5329  10687  55190  7077  124565  90853  249  24147  221336  7298  23362  5831  51200  290  84141  7857  114801  1026  5999  11341  10381  8614  5806  4015  57214 | HBB  MB  CA3  C20orf166  TMOD4  MYL3  CASQ1  AMPD1  MYBPC1  ASB10  MYOZ3  HBA2  LPL  SLC7A2  MYOZ1  CLIC5  SRD5A2L2  IDI2  S100A1  SMTNL2  HHATL  RTN4  RPL3L  SLC36A2  ANKRD23  NRAP  FSD2  CA2  C8orf22  PYGM  ABLIM2  MYF6  ASB12  TMEM52  XIRP2  ANKRD2  C1QA  ALDH1L1  KLHDC6  SLC16A10  ITM2A  SERPINA5  CAPN3  MYOC  NIPSNAP3B  AQP4  MAOB  GIMAP7  C10orf71  CPT1B  DARC  C9orf164  RASL12  KCNA7  RGS5  C1QB  AGTRL1  TRIM7  PRKCQ  ADSSL1  ZBTB16  HRASLS  KLHL34  HMGCS2  CNTFR  C7orf53  MYH7B  UCP3  CD93  CA4  CIDEA  CMYA5  PTP4A3  C9orf61  ITGB1BP3  FABP4  LOC338328  CDH5  THRSP  PFKFB1  TM6SF1  ADH1A  ABCG1  LDHD  GIMAP6  PLVAP  GPT  LMO1  GPD1  CCL15  C2orf40  SLC29A2  ART3  LOC161247  ESRRG  MYH11  GIMAP4  DZIP3  KCNJ11  ABRA  FAM107A  RNASE1  TMEM88  SCN4B  ADH1C  C20orf160  CTSG  SYNE2  MS4A6A  LRP1B  MEOX1  EMCN  CXCL14  COX4I2  AIF1  CSF1R  PLN  ATP2B2  UGT3A1  TYRP1  SLC38A3  CABC1  PEBP4  GIMAP1  TXLNB  SLC25A34  TMEM38A  DACH1  MLXIPL  WDR62  KCNA5  RGS9BP  HYAL1  GPC3  PDLIM5  JPH1  RXRG  WNK2  MYO5C  PLCD4  FXYD1  NMNAT3  TSPAN18  MUM1L1  SOX7  MOSC1  C15orf27  FLJ32310  MYLK2  IL17D  GIMAP5  SAMD4A  CUL3  LOH3CR2A  RASGRP3  ADPRHL1  GJA4  TUBA8  FLG2  HPN  RCSD1  FAM46C  FOLR2  G0S2  SCN1B  FGD5  CCDC69  PER1  KIAA1161  CLYBL  ADHFE1  DKFZp451A211  HIGD1B  ACACB  LEP  BHLHB3  ZNF541  RRAGD  CPEB3  PPP1R16B  TM4SF18  ATP2A2  FHL3  CCDC21  BDH1  USP13  PPM1J  EPM2A  CITED4  APCDD1  TFEB  DYRK1B  CACNG6  C1orf21  PDZD2  C3orf45  TCEA3  ZNF91  ABHD1  ACSS1  IGSF6  GPR44  IMPA2  FCER1A  MLCK  EFNA1  LMO2  BCL2  PACSIN3  GMFG  ESR1  COQ10A  PNPLA7  SCN2B  PFKM  MAPK12  ANK1  GUCY1A3  FMO2  PKIA  GALNTL2  GPD1L  C1orf127  COLQ  LHX6  GAMT  SPOCK2  SLC6A1  FAM78A  SNTA1  IQWD1  GHR  FEM1A  MACROD1  FKBP5  MAPT  CD38  ACADM  KIAA1737  AMICA1  MKNK2  ROBO4  TACC2  DCUN1D2  AR  L2HGDH  ATP1B1  RHOU  C22orf32  NXN  CBX7  LOC205251  C10orf92  EPB49  SHMT1  PPP1R13B  ATP5G1  ACO2  APOB  CUTC  ARMETL1  GBAS  OGDH  XPO4  SSH2  LOC128977  LCP2  SESN1  TBC1D4  GPT2  FGL2  STBD1  ABCA5  PPAPDC3  FHL1  PPP2R3A  CLIP1  DKFZP564O0823  NUDT8  FBXO32  ASPH  HCN1  KIAA0828  MLLT6  CRY2  PECAM1  GOT2  PLA2G4C  CD163  SPINK2  MYLIP  KIF1C  GSTM4  DNAJB5  NDUFS7  CX3CL1  EIF4E3  ECH1  HADH  C20orf26  NANOS1  TBC1D8  ANKRD46  DPEP2  ST6GALNAC2  GFOD1  LMOD3  FKBP3  PCNT  BTG2  RNF123  PPP1R3F  ADRB2  PPP1R16A  C11orf67  PHYH  WWP1  HSDL2  FLJ90650  COQ3  TRIP10  ECHDC2  AKAP7  USP47  ESRRA  HSD17B7P2  LIAS  TAPT1  ASB2  IPO13  BCKDHA  UBE2G1  COQ7  SLC25A11  NEURL  VIT  DLAT  ACSS2  DUSP28  SIX1  SLCO2B1  ACAT1  SDHA  HBD  C16orf14  PPP1CB  LCP1  MFN2  C1orf162  TOB1  USP15  MT1X  COX10  RPUSD4  PIB5PA  ACSL1  MARCH6  SLC6A8  DCI  COQ9  SUCLA2  DUSP3  KLHL23  ZYG11B  CYCS  UQCRC1  RXRA  ARHGAP15  FHL5  SLC16A14  H3F3B  KIF1B  UBAC1  MITF  OSBPL1A  HERC1  FAM125B  NDUFS2  ALDH2  CSDE1  FLJ39378  ZNF76  DDX46  GSTM2  ATP5D  PARVB  AFG3L2  TTLL2  C6orf106  UQCRB  RBM9  RAD23A  NDUFB9  C9orf23  PTGES2  TTC32  PDHA1  HSD17B7  SIRT5  TRAP1  ST3GAL3  MRPL2  PSCD1  MID1IP1  PDHX  BLCAP  AHDC1  PER2  CUL5  PPP3CB  ATPAF2  C6orf136  FXR2  GFM1  TIE1  INPP5A  POPDC3  DGCR6  NDUFV2  KLF9  COQ6  DDX59  UGP2  UCKL1  TSPYL1  SLMAP  RBJ  BTBD6  AES  STAT5B  NSFL1C  ZNF291  ACYP1  CS  CYC1  CHCHD3  MRPL33  KIAA1787  ZNF768  CAPN6  CCDC43  JPH2  NDUFA9  SDHD  VDAC3  WDR23  C9orf5  NDUFB10  DULLARD  THRAP4  ZNF124  ATP5S  MRPL38  ATG4A  PPP1R2  FLII  KIF21A  NDUFC1  HSF2  APOO  HDHD1A  REV1  C20orf7  CALCOCO2  PDE4A  NDUFA10  DENND2C  ECSIT  CCDC101  NDUFV1  FBXO3  DIP2C  SFXN4  KALRN  NR1D1  COX5A  C5orf32  APIP  RBM17  DIAPH2  ARAF  XPA  HIF1AN  MDH2  RAF1  FRAT2  C8orf38  MTRF1  SETD3  CHMP6  SDHB  KEAP1  PRKAR2A  SAMM50  C9orf86  CSDA  TRIM43  LOC88523  COQ4  MRPS28  ZCD1  MRPL46  MRPS18A  TPD52L3  ANKRD40  NUCKS1  KIAA0460  KCNJ10  KLHDC3  ABCA12  CRNKL1  NLRX1  ZNF438  MRPS9  ELSPBP1  UQCRFS1  FOXD4L2  CCDC44  POLDIP2  APPL1  KTN1  PDCD2L  DEXI  NCOA1  FH  TRIM24  SLC19A2  GIN1  SAP18  KBTBD2  MEF2D  IMMT  NDUFAF1  ZNF511  PREB  MATN4  MRPS15  KIAA0182  PSEN1  NFYC  IDH3B  LAIR1  ACOX1  HNRPL  GARNL1  NDUFA4  CAPZA2  LNX2  MRPS7  OXR1  UBAP2  PAIP2  LAPTM4B  ZNF323  PSMA2  OXA1L  PHB  PCM1  TPRKB  UBE3B  MRPL1  NARS2  NDUFAB1  SENP2  QDPR  CWC15  PKD1  VPS4A  MRPL16  MBNL1  UNC84A  PSMF1  TBC1D14  PDPR  VBP1  LOC91661  TMEM93  THRA  PECI  TMEM111  MRPS24  MRPS31  COPS4  TPCN2  KATNB1  SAE1  SRP72  TIPRL  HSPC152  ARFIP1  SDCCAG3  CLCN7  POLR2H  MRLC2  RARS2  DNAL4  LOC440589  XPNPEP1  MTPN  RPS12  HPS5  NCLN  GTF2H3  RBM4  GOLGA5  CASC4  WASF3  CTNNA1  LACTB  BCAP31  CDK2AP1  PANK2  UNC45A  PPP4C  ZZZ3  GEMIN6  C14orf135  TMEM117  PLEKHO1  C9orf19  CYP2U1  B3GALT6  TICAM1  ADAR  SEP15  C20orf3  ARPC1A  PHF11  SH3BGRL  GLTP  PDCD6  C20orf117  UBE2I  PEX16  SAP30L  TMCO1  STK4  GPAA1  TSEN34  TM9SF3  BET1L  AP2S1  B2M  BUD31  PHLDB1  TRAPPC3  SLC35B2  RPS19  PRRC1  PAICS  YIF1A  TMEM184B  COMMD2  RPLP0  DAD1  NAT9  APRT  TAP1  GNB1  SARS  POFUT1  SEC31A  IQGAP1  WDR1  ACTL6A  C14orf32  NOLA3  PTRF  SH3PX3  CETN2  DBNL  NIPA2  GNL1  C9orf30  TXNL5  ZMPSTE24  CAPRIN2  NME4  SAMD9L  RALA  TXNDC1  MSTO1  PPIL5  ATP6V0E1  ZNF226  ANKRD13A  LRCH2  COPG  TSPO  PRNP  DSCR2  COMT  PRMT2  CTTN  LIMK1  MEA1  ABL1  YIPF5  LOC348262  EHBP1  KTELC1  CLN5  TMEM183A  C17orf45  DTWD1  AP3B1  TGFB1I1  RPN1  ZMYM6  CDK4  SEP9  TAF2  GLG1  ALG3  LOC650152  FLJ35220  NEU1  TM9SF1  FNDC3B  RHBDD2  ARF3  GNS  MYL6  UBE2Z  LRP11  CD81  PPP1R14B  SND1  MFGE8  NSDHL  FADD  S100PBP  OCIAD2  ZNF329  DCTD  SLC25A5  SLC25A6  LRRC32  TNFRSF1A  LAMP1  NLGN2  TBPL1  MAP4K5  DNCL1  PRTFDC1  ERLIN1  ACTB  DBN1  M6PRBP1  CNIH  C12orf24  CLTA  POLR3H  IFITM2  TRAM1  ITGB1  UBE2E2  RPS27L  DEGS1  OSBPL8  MPZL1  SURF4  MFSD1  NAGK  PDPN  MVD  IL4R  SLC17A5  SERF2  EEF1A1  EXT2  LOC727761  YARS  TCEAL3  SFRS9  REXO2  ELOVL1  TULP3  PLXNA1  MED19  DDOST  ZC3HAV1  PRKCSH  RABAC1  TETRAN  MAN2B2  GMFB  SLC39A11  KLHDC5  RNF135  SLC38A6  ALG8  FBXO5  ARMCX1  CEP290  LMNA  MGC40499  SGPP1  LRP10  COX6A1  SDF2L1  C19orf48  JARID1B  C12orf23  TMEM4  CLPTM1L  SHMT2  LAMC1  C14orf45  SLC25A24  RAB8A  IFITM3  NUCB2  LASP1  CD151  LASS5  ARPC5  CNN2  LDOC1  CASP3  AXL  RAB23  DSE  SDF4  SGCE  MAGED1  GBP1  HMGCR  SYDE1  ATP1B3  SSR1  RPL3  RHOC  BNIP3L  CFL1  LOC731049  FAM46A  CD99  UNC119  CMTM6  XPO6  HSP90B1  ITGAV  NAV1  GTF2E2  RCC2  STT3A  SCPEP1  EIF4A1  ZNF668  P4HB  ATP6AP2  ZDHHC24  SLC35B3  LARP6  MIF  PGM3  SEC61A1  TPST1  CHIC2  TSHZ3  NPC2  DKFZp762E1312  HEXB  LOC57228  CAPG  ARHGEF2  TMEM138  CD63  LTBR  RRAS  LAMB1  ABHD12  TGIF1  XPR1  PRELID1  C1R  SPA17  PEA15  PYGB  POFUT2  SSR4  EML4  HSPA5  TRIP6  STX3  SPIN4  ARF4  SUMF1  MGC33212  CCDC109B  C6orf48  SHC1  SLC30A7  FSTL1  CDC42EP1  COL3A1  NME7  TMEM98  COL6A2  HYOU1  RPL23A  FYN  GRN  NRP1  ARMCX2  GPNMB  MMP23A  C18orf10  RHOG  TMED9  TRIM22  NAT14  ZYX  OPRS1  ARMET  PIGO  SPATA2L  TMSB10  SLC20A1  TRIOBP  CAND1  GGH  NME1  CASP4  C20orf59  ENO1  TAX1BP3  MID1  COPB2  SMG6  NRAS  EVC  ACLY  PGAM4  SLC30A5  C4orf34  IFI16  CCNE2  MVP  SLFN12  LENG4  TMEM14A  FSCN1  RCN1  PGLS  SKIL  CALD1  ATOX1  ANTXR2  COL6A3  C10orf61  SNAPC2  RAB31  PLK4  HIST1H2BK  CALU  SOCS1  UGCGL2  MYO9B  EFEMP2  KDELR2  TMPO  FGF1  WIPF2  PDIA4  PARVA  CTSA  LAMP2  CLIC3  DAP  FAM38A  NGFRAP1  RPN2  CKAP4  PRMT6  FEZ1  TAGLN  TXNDC5  TRIM5  XYLB  VKORC1  ITPKA  FAM14A  ST7L  TUBA1C  NQO1  AKAP10  TMEM97  TMEM44  SLC10A7  GPR137B  FBXO4  AMMECR1  PRDX4  COL1A2  ALG2  LY96  CDK6  ITPR3  OLFML3  BMP1  FTL  ITGB5  DAB2  COL6A1  EZH2  RAB34  MDK  EXT1  INA  RIPK2  ADM  HMMR  NUDT5  IMPDH1  DUSP14  C5orf34  ITGA5  STIL  SLC6A9  FOXD1  TKT  TP53I3  RAB32  SC4MOL  SH3BGRL3  DC2  ACOT7  PDIA6  FNDC4  PPIB  CHPF  PIGK  C20orf102  ANXA2  PPAPDC1B  NHEDC2  ECM1  LRRC49  HHLA3  DCBLD2  NACA  FAM129B  GMIP  ABCC3  MAP1B  LOC150223  CDR2L  SH3PXD2B  C1orf102  ARSB  DPP4  SLC22A18  ZMAT3  HAGHL  MYO5A  CUTA  SLC25A17  ZNF365  S100A11  SRPX2  MASTL  TECT1  PI4K2B  C19orf10  GPR172A  PDIA5  IKIP  COL5A1  HTRA1  SUMF2  PHGDH  FKBP10  ULBP1  CLIP3  CENPM  CHSY-2  LOXL1  TTLL1  VCAN  GLIPR1  CD248  TBC1D8B  SEC14L2  G6PD  QPCT  HYAL3  SNAI2  TNFRSF10B  CDR2  C14orf79  TAF1A  RCN3  ADM2  ELL2  DKFZP564J0863  CCND1  PLAU  SLC44A3  SLC7A1  SERPINH1  FAP  LEPRE1  UBXD6  THY1  KDELR3  DNAJC3  TGFBI  CCDC80  TRAM2  C20orf42  C11orf41  ARL13B  CDC7  TBC1D2  P4HA2  PGM2  SHKBP1  C2orf37  STAT1  RAB33A  TRIP13  CDH11  PI4K2A  NME5  UAP1L1  CPZ  CDKN2A  VEGFC  FKBP11  SKIV2L  HM13  THBS2  SGMS2  CEECAM1  MYO1E  VDR  ALCAM  GBA  SPOCK1  TMEM136  DNAJC10  SERPINE2  ASPHD2  TMED3  CEP55  PLOD2  SCN9A  COL1A1  SERPINB8  LOXL3  SPCS3  NFASC  PCNXL2  ABHD2  MTHFD1L  RGS17  CA12  BCAS4  FBXO22  MMP14  LOC400406  TIMP1  C18orf56  PDLIM4  NPAS1  CTHRC1  GALNT12  HIF1A  LOC493869  ARMC9  BDKRB1  TMEM119  CSGlcA-T  GALNS  LPIN2  SLC1A1  IGF2BP3  PLOD3  FN1  TOP2A  MOXD1  C11orf70  KDELC1  LAMA1  MFAP2  TPBG  CH25H  PTK7  ESM1  PLAUR  PNMA2  NUDT11  TIMP2  MGC15523  SPOCD1  ALPL  FJX1  C6orf65  TYMS  PSD3  PYCR1  CPA4  ANPEP  TMEM166  SCG2  KIAA1913  CDKN1A  RGS4  SCRG1  TUBB3  STC2  PTX3  LOX  KIAA1199 | | hemoglobin, beta  myoglobin  carbonic anhydrase III, muscle specific  chromosome 20 open reading frame 166  tropomodulin 4 (muscle)  myosin, light chain 3, alkali; ventricular, skeletal, slow  calsequestrin 1 (fast-twitch, skeletal muscle)  adenosine monophosphate deaminase 1 (isoform M)  myosin binding protein C, slow type  ankyrin repeat and SOCS box-containing 10  myozenin 3  hemoglobin, alpha 2  lipoprotein lipase  solute carrier family 7 (cationic amino acid transporter, y+ system), member 2  myozenin 1  chloride intracellular channel 5  steroid 5 alpha-reductase 2-like 2  isopentenyl-diphosphate delta isomerase 2  S100 calcium binding protein A1  smoothelin-like 2  hedgehog acyltransferase-like  reticulon 4  ribosomal protein L3-like  solute carrier family 36 (proton/amino acid symporter), member 2  ankyrin repeat domain 23  nebulin-related anchoring protein  fibronectin type III and SPRY domain containing 2  carbonic anhydrase II  chromosome 8 open reading frame 22  phosphorylase, glycogen; muscle (McArdle syndrome, glycogen storage disease type V)  actin binding LIM protein family, member 2  myogenic factor 6 (herculin)  ankyrin repeat and SOCS box-containing 12  transmembrane protein 52  xin actin-binding repeat containing 2  ankyrin repeat domain 2 (stretch responsive muscle)  complement component 1, q subcomponent, A chain  aldehyde dehydrogenase 1 family, member L1  kelch domain containing 6  solute carrier family 16, member 10 (aromatic amino acid transporter)  integral membrane protein 2A  serpin peptidase inhibitor, clade A (alpha-1 antiproteinase, antitrypsin), member 5  calpain 3, (p94)  myocilin, trabecular meshwork inducible glucocorticoid response  nipsnap homolog 3B (C. elegans)  aquaporin 4  monoamine oxidase B  GTPase, IMAP family member 7  chromosome 10 open reading frame 71  carnitine palmitoyltransferase 1B (muscle)  Duffy blood group, chemokine receptor  chromosome 9 open reading frame 164  RAS-like, family 12  potassium voltage-gated channel, shaker-related subfamily, member 7  regulator of G-protein signaling 5  complement component 1, q subcomponent, B chain  apelin receptor  tripartite motif-containing 7  protein kinase C, theta  adenylosuccinate synthase like 1  zinc finger and BTB domain containing 16  HRAS-like suppressor  kelch-like 34 (Drosophila)  3-hydroxy-3-methylglutaryl-Coenzyme A synthase 2 (mitochondrial)  ciliary neurotrophic factor receptor  chromosome 7 open reading frame 53  myosin, heavy chain 7B, cardiac muscle, beta  uncoupling protein 3 (mitochondrial, proton carrier)  CD93 molecule  carbonic anhydrase IV  cell death-inducing DFFA-like effector a  cardiomyopathy associated 5  protein tyrosine phosphatase type IVA, member 3  chromosome 9 open reading frame 61  integrin beta 1 binding protein 3  fatty acid binding protein 4, adipocyte  glycosylphosphatidylinositol anchored high density lipoprotein binding protein 1  cadherin 5, type 2, VE-cadherin (vascular epithelium)  thyroid hormone responsive (SPOT14 homolog, rat)  6-phosphofructo-2-kinase/fructose-2,6-biphosphatase 1  transmembrane 6 superfamily member 1  alcohol dehydrogenase 1A (class I), alpha polypeptide  ATP-binding cassette, sub-family G (WHITE), member 1  lactate dehydrogenase D  GTPase, IMAP family member 6  plasmalemma vesicle associated protein  glutamic-pyruvate transaminase (alanine aminotransferase)  LIM domain only 1 (rhombotin 1)  glycerol-3-phosphate dehydrogenase 1 (soluble)  chemokine (C-C motif) ligand 15  chromosome 2 open reading frame 40  solute carrier family 29 (nucleoside transporters), member 2  ADP-ribosyltransferase 3  fat-inducing transcript 1  estrogen-related receptor gamma  myosin, heavy chain 11, smooth muscle  GTPase, IMAP family member 4  DAZ interacting protein 3, zinc finger  potassium inwardly-rectifying channel, subfamily J, member 11  actin-binding Rho activating protein  family with sequence similarity 107, member A  ribonuclease, RNase A family, 1 (pancreatic)  transmembrane protein 88  sodium channel, voltage-gated, type IV, beta  alcohol dehydrogenase 1C (class I), gamma polypeptide  chromosome 20 open reading frame 160  cathepsin G  spectrin repeat containing, nuclear envelope 2  membrane-spanning 4-domains, subfamily A, member 6A  low density lipoprotein-related protein 1B (deleted in tumors)  mesenchyme homeobox 1  endomucin  chemokine (C-X-C motif) ligand 14  cytochrome c oxidase subunit IV isoform 2 (lung)  allograft inflammatory factor 1  colony stimulating factor 1 receptor, formerly McDonough feline sarcoma viral (v-fms) oncogene homolog  phospholamban  ATPase, Ca++ transporting, plasma membrane 2  UDP glycosyltransferase 3 family, polypeptide A1  tyrosinase-related protein 1  solute carrier family 38, member 3  chaperone, ABC1 activity of bc1 complex homolog (S. pombe)  phosphatidylethanolamine-binding protein 4  GTPase, IMAP family member 1  taxilin beta  solute carrier family 25, member 34  transmembrane protein 38A  dachshund homolog 1 (Drosophila)  MLX interacting protein-like  WD repeat domain 62  potassium voltage-gated channel, shaker-related subfamily, member 5  regulator of G protein signaling 9 binding protein  hyaluronoglucosaminidase 1  glypican 3  PDZ and LIM domain 5  junctophilin 1  retinoid X receptor, gamma  WNK lysine deficient protein kinase 2  myosin VC  phospholipase C, delta 4  FXYD domain containing ion transport regulator 1 (phospholemman)  nicotinamide nucleotide adenylyltransferase 3  tetraspanin 18  melanoma associated antigen (mutated) 1-like 1  SRY (sex determining region Y)-box 7  MOCO sulphurase C-terminal domain containing 1  chromosome 15 open reading frame 27  ATP/GTP binding protein-like 1  myosin light chain kinase 2  interleukin 17D  GTPase, IMAP family member 5  sterile alpha motif domain containing 4A  cullin 3  loss of heterozygosity, 3, chromosomal region 2, gene A  RAS guanyl releasing protein 3 (calcium and DAG-regulated)  ADP-ribosylhydrolase like 1  gap junction protein, alpha 4, 37kDa  tubulin, alpha 8  filaggrin family member 2  hepsin (transmembrane protease, serine 1)  RCSD domain containing 1  family with sequence similarity 46, member C  folate receptor 2 (fetal)  G0/G1switch 2  sodium channel, voltage-gated, type I, beta  FYVE, RhoGEF and PH domain containing 5  coiled-coil domain containing 69  period homolog 1 (Drosophila)  KIAA1161  citrate lyase beta like  alcohol dehydrogenase, iron containing, 1  DKFZp451A211 protein  HIG1 domain family, member 1B  acetyl-Coenzyme A carboxylase beta  leptin  basic helix-loop-helix domain containing, class B, 3  zinc finger protein 541  Ras-related GTP binding D  cytoplasmic polyadenylation element binding protein 3  protein phosphatase 1, regulatory (inhibitor) subunit 16B  transmembrane 4 L six family member 18  ATPase, Ca++ transporting, cardiac muscle, slow twitch 2  four and a half LIM domains 3  coiled-coil domain containing 21  3-hydroxybutyrate dehydrogenase, type 1  ubiquitin specific peptidase 13 (isopeptidase T-3)  protein phosphatase 1J (PP2C domain containing)  epilepsy, progressive myoclonus type 2A, Lafora disease (laforin)  Cbp/p300-interacting transactivator, with Glu/Asp-rich carboxy-terminal domain, 4  adenomatosis polyposis coli down-regulated 1  transcription factor EB  dual-specificity tyrosine-(Y)-phosphorylation regulated kinase 1B  calcium channel, voltage-dependent, gamma subunit 6  chromosome 1 open reading frame 21  PDZ domain containing 2  chromosome 3 open reading frame 45  transcription elongation factor A (SII), 3  zinc finger protein 91  abhydrolase domain containing 1  acyl-CoA synthetase short-chain family member 1  immunoglobulin superfamily, member 6  G protein-coupled receptor 44  inositol(myo)-1(or 4)-monophosphatase 2  Fc fragment of IgE, high affinity I, receptor for; alpha polypeptide  myosin light chain kinase 3  ephrin-A1  LIM domain only 2 (rhombotin-like 1)  B-cell CLL/lymphoma 2  protein kinase C and casein kinase substrate in neurons 3  glia maturation factor, gamma  estrogen receptor 1  coenzyme Q10 homolog A (S. cerevisiae)  patatin-like phospholipase domain containing 7  sodium channel, voltage-gated, type II, beta  phosphofructokinase, muscle  mitogen-activated protein kinase 12  ankyrin 1, erythrocytic  guanylate cyclase 1, soluble, alpha 3  flavin containing monooxygenase 2 (non-functional)  protein kinase (cAMP-dependent, catalytic) inhibitor alpha  UDP-N-acetyl-alpha-D-galactosamine:polypeptide N-acetylgalactosaminyltransferase  -like 2  glycerol-3-phosphate dehydrogenase 1-like  chromosome 1 open reading frame 127  collagen-like tail subunit (single strand of homotrimer) of asymmetric acetylcholinesterase  LIM homeobox 6  guanidinoacetate N-methyltransferase  sparc/osteonectin, cwcv and kazal-like domains proteoglycan (testican) 2  solute carrier family 6 (neurotransmitter transporter, GABA), member 1  family with sequence similarity 78, member A  syntrophin, alpha 1 (dystrophin-associated protein A1, 59kDa, acidic component)  IQ motif and WD repeats 1  growth hormone receptor  fem-1 homolog a (C. elegans)  MACRO domain containing 1  FK506 binding protein 5  microtubule-associated protein tau  CD38 molecule  acyl-Coenzyme A dehydrogenase, C-4 to C-12 straight chain  KIAA1737  adhesion molecule, interacts with CXADR antigen 1  MAP kinase interacting serine/threonine kinase 2  roundabout homolog 4, magic roundabout (Drosophila)  transforming, acidic coiled-coil containing protein 2  DCN1, defective in cullin neddylation 1, domain containing 2 (S. cerevisiae)  androgen receptor (dihydrotestosterone receptor; testicular feminization; spinal and bulbar muscular atrophy; Kennedy disease)  L-2-hydroxyglutarate dehydrogenase  ATPase, Na+/K+ transporting, beta 1 polypeptide  ras homolog gene family, member U  chromosome 22 open reading frame 32  nucleoredoxin  chromobox homolog 7  N/A  chromosome 10 open reading frame 92  erythrocyte membrane protein band 4.9 (dematin)  serine hydroxymethyltransferase 1 (soluble)  protein phosphatase 1, regulatory (inhibitor) subunit 13B  ATP synthase, H+ transporting, mitochondrial F0 complex, subunit C1 (subunit 9)  aconitase 2, mitochondrial  apolipoprotein B (including Ag(x) antigen)  cutC copper transporter homolog (E. coli)  arginine-rich, mutated in early stage tumors-like 1  glioblastoma amplified sequence  oxoglutarate (alpha-ketoglutarate) dehydrogenase (lipoamide)  exportin 4  slingshot homolog 2 (Drosophila)  chromosome 22 open reading frame 39  lymphocyte cytosolic protein 2 (SH2 domain containing leukocyte protein of 76kDa)  sestrin 1  TBC1 domain family, member 4  glutamic pyruvate transaminase (alanine aminotransferase) 2  fibrinogen-like 2  starch binding domain 1  ATP-binding cassette, sub-family A (ABC1), member 5  phosphatidic acid phosphatase type 2 domain containing 3  four and a half LIM domains 1  protein phosphatase 2 (formerly 2A), regulatory subunit B'', alpha  CAP-GLY domain containing linker protein 1  DKFZP564O0823 protein  nudix (nucleoside diphosphate linked moiety X)-type motif 8  F-box protein 32  aspartate beta-hydroxylase  hyperpolarization activated cyclic nucleotide-gated potassium channel 1  S-adenosylhomocysteine hydrolase-like 2  myeloid/lymphoid or mixed-lineage leukemia (trithorax homolog, Drosophila); translocated to, 6  cryptochrome 2 (photolyase-like)  platelet/endothelial cell adhesion molecule (CD31 antigen)  glutamic-oxaloacetic transaminase 2, mitochondrial (aspartate aminotransferase 2)  phospholipase A2, group IVC (cytosolic, calcium-independent)  CD163 molecule  serine peptidase inhibitor, Kazal type 2 (acrosin-trypsin inhibitor)  myosin regulatory light chain interacting protein  kinesin family member 1C  glutathione S-transferase M4  DnaJ (Hsp40) homolog, subfamily B, member 5  NADH dehydrogenase (ubiquinone) Fe-S protein 7, 20kDa (NADH-coenzyme Q reductase)  chemokine (C-X3-C motif) ligand 1  eukaryotic translation initiation factor 4E family member 3  enoyl Coenzyme A hydratase 1, peroxisomal  hydroxyacyl-Coenzyme A dehydrogenase  chromosome 20 open reading frame 26  nanos homolog 1 (Drosophila)  TBC1 domain family, member 8 (with GRAM domain)  ankyrin repeat domain 46  dipeptidase 2  ST6 (alpha-N-acetyl-neuraminyl-2,3-beta-galactosyl-1,3)-N-acetylgalactosaminide alpha-2,6-sialyltransferase 2  glucose-fructose oxidoreductase domain containing 1  leiomodin 3 (fetal)  FK506 binding protein 3, 25kDa  pericentrin  BTG family, member 2  ring finger protein 123  protein phosphatase 1, regulatory (inhibitor) subunit 3F  adrenergic, beta-2-, receptor, surface  protein phosphatase 1, regulatory (inhibitor) subunit 16A  chromosome 11 open reading frame 67  phytanoyl-CoA 2-hydroxylase  WW domain containing E3 ubiquitin protein ligase 1  hydroxysteroid dehydrogenase like 2  laeverin  coenzyme Q3 homolog, methyltransferase (S. cerevisiae)  thyroid hormone receptor interactor 10  enoyl Coenzyme A hydratase domain containing 2  A kinase (PRKA) anchor protein 7  ubiquitin specific peptidase 47  estrogen-related receptor alpha  N/A  lipoic acid synthetase  transmembrane anterior posterior transformation 1  ankyrin repeat and SOCS box-containing 2  importin 13  branched chain keto acid dehydrogenase E1, alpha polypeptide  ubiquitin-conjugating enzyme E2G 1 (UBC7 homolog, yeast)  coenzyme Q7 homolog, ubiquinone (yeast)  solute carrier family 25 (mitochondrial carrier; oxoglutarate carrier), member 11  neuralized homolog (Drosophila)  vitrin  dihydrolipoamide S-acetyltransferase  acyl-CoA synthetase short-chain family member 2  dual specificity phosphatase 28  SIX homeobox 1  solute carrier organic anion transporter family, member 2B1  acetyl-Coenzyme A acetyltransferase 1 (acetoacetyl Coenzyme A thiolase)  succinate dehydrogenase complex, subunit A, flavoprotein (Fp)  hemoglobin, delta  chromosome 16 open reading frame 14  protein phosphatase 1, catalytic subunit, beta isoform  lymphocyte cytosolic protein 1 (L-plastin)  mitofusin 2  chromosome 1 open reading frame 162  transducer of ERBB2, 1  ubiquitin specific peptidase 15  metallothionein 1X  COX10 homolog, cytochrome c oxidase assembly protein, heme A: farnesyltransferase (yeast)  RNA pseudouridylate synthase domain containing 4  phosphatidylinositol (4,5) bisphosphate 5-phosphatase, A  acyl-CoA synthetase long-chain family member 1  membrane-associated ring finger (C3HC4) 6  solute carrier family 6 (neurotransmitter transporter, creatine), member 8  dodecenoyl-Coenzyme A delta isomerase (3,2 trans-enoyl-Coenzyme A isomerase)  coenzyme Q9 homolog (S. cerevisiae)  succinate-CoA ligase, ADP-forming, beta subunit  dual specificity phosphatase 3  kelch-like 23 (Drosophila)  zyg-11 homolog B (C. elegans)  cytochrome c, somatic  ubiquinol-cytochrome c reductase core protein I  retinoid X receptor, alpha  Rho GTPase activating protein 15  four and a half LIM domains 5  solute carrier family 16, member 14 (monocarboxylic acid transporter 14)  H3 histone, family 3B (H3.3B)  kinesin family member 1B  UBA domain containing 1  microphthalmia-associated transcription factor  oxysterol binding protein-like 1A  hect (homologous to the E6-AP (UBE3A) carboxyl terminus) domain and RCC1 (CHC1)-like domain (RLD) 1  family with sequence similarity 125, member B  NADH dehydrogenase (ubiquinone) Fe-S protein 2, 49kDa (NADH-coenzyme Q reductase)  aldehyde dehydrogenase 2 family (mitochondrial)  cold shock domain containing E1, RNA-binding  Rab interacting lysosomal protein-like 1  zinc finger protein 76 (expressed in testis)  DEAD (Asp-Glu-Ala-Asp) box polypeptide 46  glutathione S-transferase M2 (muscle)  ATP synthase, H+ transporting, mitochondrial F1 complex, delta subunit  parvin, beta  AFG3 ATPase family gene 3-like 2 (yeast)  tubulin tyrosine ligase-like family, member 2  chromosome 6 open reading frame 106  ubiquinol-cytochrome c reductase binding protein  RNA binding motif protein 9  RAD23 homolog A (S. cerevisiae)  NADH dehydrogenase (ubiquinone) 1 beta subcomplex, 9, 22kDa  chromosome 9 open reading frame 23  prostaglandin E synthase 2  tetratricopeptide repeat domain 32  pyruvate dehydrogenase (lipoamide) alpha 1  hydroxysteroid (17-beta) dehydrogenase 7  sirtuin (silent mating type information regulation 2 homolog) 5 (S. cerevisiae)  TNF receptor-associated protein 1  ST3 beta-galactoside alpha-2,3-sialyltransferase 3  mitochondrial ribosomal protein L2  pleckstrin homology, Sec7 and coiled-coil domains 1(cytohesin 1)  MID1 interacting protein 1 (gastrulation specific G12 homolog (zebrafish))  pyruvate dehydrogenase complex, component X  bladder cancer associated protein  AT hook, DNA binding motif, containing 1  period homolog 2 (Drosophila)  cullin 5  protein phosphatase 3 (formerly 2B), catalytic subunit, beta isoform  ATP synthase mitochondrial F1 complex assembly factor 2  chromosome 6 open reading frame 136  fragile X mental retardation, autosomal homolog 2  G elongation factor, mitochondrial 1  tyrosine kinase with immunoglobulin-like and EGF-like domains 1  inositol polyphosphate-5-phosphatase, 40kDa  popeye domain containing 3  DiGeorge syndrome critical region gene 6  NADH dehydrogenase (ubiquinone) flavoprotein 2, 24kDa  Kruppel-like factor 9  coenzyme Q6 homolog, monooxygenase (S. cerevisiae)  DEAD (Asp-Glu-Ala-Asp) box polypeptide 59  UDP-glucose pyrophosphorylase 2  uridine-cytidine kinase 1-like 1  TSPY-like 1  sarcolemma associated protein  rab and DnaJ domain containing  BTB (POZ) domain containing 6  amino-terminal enhancer of split  signal transducer and activator of transcription 5B  NSFL1 (p97) cofactor (p47)  S phase cyclin A-associated protein in the ER  acylphosphatase 1, erythrocyte (common) type  citrate synthase  cytochrome c-1  coiled-coil-helix-coiled-coil-helix domain containing 3  mitochondrial ribosomal protein L33  KIAA1787 protein  zinc finger protein 768  calpain 6  coiled-coil domain containing 43  junctophilin 2  NADH dehydrogenase (ubiquinone) 1 alpha subcomplex, 9, 39kDa  succinate dehydrogenase complex, subunit D, integral membrane protein  voltage-dependent anion channel 3  WD repeat domain 23  chromosome 9 open reading frame 5  NADH dehydrogenase (ubiquinone) 1 beta subcomplex, 10, 22kDa  dullard homolog (Xenopus laevis)  mediator complex subunit 24  zinc finger protein 124  ATP synthase, H+ transporting, mitochondrial F0 complex, subunit s (factor B)  mitochondrial ribosomal protein L38  ATG4 autophagy related 4 homolog A (S. cerevisiae)  protein phosphatase 1, regulatory (inhibitor) subunit 2  flightless I homolog (Drosophila)  kinesin family member 21A  NADH dehydrogenase (ubiquinone) 1, subcomplex unknown, 1, 6kDa  heat shock transcription factor 2  apolipoprotein O  haloacid dehalogenase-like hydrolase domain containing 1A  REV1 homolog (S. cerevisiae)  chromosome 20 open reading frame 7  calcium binding and coiled-coil domain 2  phosphodiesterase 4A, cAMP-specific (phosphodiesterase E2 dunce homolog, Drosophila)  NADH dehydrogenase (ubiquinone) 1 alpha subcomplex, 10, 42kDa  DENN/MADD domain containing 2C  ECSIT homolog (Drosophila)  coiled-coil domain containing 101  NADH dehydrogenase (ubiquinone) flavoprotein 1, 51kDa  F-box protein 3  DIP2 disco-interacting protein 2 homolog C (Drosophila)  sideroflexin 4  kalirin, RhoGEF kinase  nuclear receptor subfamily 1, group D, member 1  cytochrome c oxidase subunit Va  chromosome 5 open reading frame 32  APAF1 interacting protein  RNA binding motif protein 17  diaphanous homolog 2 (Drosophila)  v-raf murine sarcoma 3611 viral oncogene homolog  xeroderma pigmentosum, complementation group A  hypoxia-inducible factor 1, alpha subunit inhibitor  malate dehydrogenase 2, NAD (mitochondrial)  v-raf-1 murine leukemia viral oncogene homolog 1  frequently rearranged in advanced T-cell lymphomas 2  chromosome 8 open reading frame 38  mitochondrial translational release factor 1  SET domain containing 3  chromatin modifying protein 6  succinate dehydrogenase complex, subunit B, iron sulfur (Ip)  kelch-like ECH-associated protein 1  protein kinase, cAMP-dependent, regulatory, type II, alpha  sorting and assembly machinery component 50 homolog (S. cerevisiae)  chromosome 9 open reading frame 86  cold shock domain protein A  tripartite motif-containing 43  NEDD4 binding protein 2-like 2  coenzyme Q4 homolog (S. cerevisiae)  mitochondrial ribosomal protein S28  CDGSH iron sulfur domain 1  mitochondrial ribosomal protein L46  mitochondrial ribosomal protein S18A  tumor protein D52-like 3  ankyrin repeat domain 40  nuclear casein kinase and cyclin-dependent kinase substrate 1  KIAA0460  potassium inwardly-rectifying channel, subfamily J, member 10  kelch domain containing 3  ATP-binding cassette, sub-family A (ABC1), member 12  crooked neck pre-mRNA splicing factor-like 1 (Drosophila)  NLR family member X1  zinc finger protein 438  mitochondrial ribosomal protein S9  epididymal sperm binding protein 1  ubiquinol-cytochrome c reductase, Rieske iron-sulfur polypeptide 1  forkhead box D4-like 2  coiled-coil domain containing 44  polymerase (DNA-directed), delta interacting protein 2  adaptor protein, phosphotyrosine interaction, PH domain and leucine zipper containing 1  kinectin 1 (kinesin receptor)  programmed cell death 2-like  dexamethasone-induced transcript  nuclear receptor coactivator 1  fumarate hydratase  tripartite motif-containing 24  solute carrier family 19 (thiamine transporter), member 2  gypsy retrotransposon integrase 1  Sin3A-associated protein, 18kDa  kelch repeat and BTB (POZ) domain containing 2  myocyte enhancer factor 2D  inner membrane protein, mitochondrial (mitofilin)  NADH dehydrogenase (ubiquinone) 1 alpha subcomplex, assembly factor 1  zinc finger protein 511  prolactin regulatory element binding  matrilin 4  mitochondrial ribosomal protein S15  KIAA0182  presenilin 1 (Alzheimer disease 3)  nuclear transcription factor Y, gamma  isocitrate dehydrogenase 3 (NAD+) beta  leukocyte-associated immunoglobulin-like receptor 1  acyl-Coenzyme A oxidase 1, palmitoyl  heterogeneous nuclear ribonucleoprotein L  GTPase activating Rap/RanGAP domain-like 1  NADH dehydrogenase (ubiquinone) 1 alpha subcomplex, 4, 9kDa  capping protein (actin filament) muscle Z-line, alpha 2  ligand of numb-protein X 2  mitochondrial ribosomal protein S7  oxidation resistance 1  ubiquitin associated protein 2  poly(A) binding protein interacting protein 2  lysosomal associated protein transmembrane 4 beta  zinc finger protein 323  proteasome (prosome, macropain) subunit, alpha type, 2  oxidase (cytochrome c) assembly 1-like  prohibitin  pericentriolar material 1  TP53RK binding protein  ubiquitin protein ligase E3B  mitochondrial ribosomal protein L1  asparaginyl-tRNA synthetase 2, mitochondrial (putative)  NADH dehydrogenase (ubiquinone) 1, alpha/beta subcomplex, 1, 8kDa  SUMO1/sentrin/SMT3 specific peptidase 2  quinoid dihydropteridine reductase  CWC15 homolog (S. cerevisiae)  polycystic kidney disease 1 (autosomal dominant)  vacuolar protein sorting 4 homolog A (S. cerevisiae)  mitochondrial ribosomal protein L16  muscleblind-like (Drosophila)  unc-84 homolog A (C. elegans)  proteasome (prosome, macropain) inhibitor subunit 1 (PI31)  TBC1 domain family, member 14  pyruvate dehydrogenase phosphatase regulatory subunit  von Hippel-Lindau binding protein 1  zinc finger protein 765  transmembrane protein 93  thyroid hormone receptor, alpha (erythroblastic leukemia viral (v-erb-a) oncogene homolog, avian)  peroxisomal D3,D2-enoyl-CoA isomerase  transmembrane protein 111  mitochondrial ribosomal protein S24  mitochondrial ribosomal protein S31  COP9 constitutive photomorphogenic homolog subunit 4 (Arabidopsis)  two pore segment channel 2  katanin p80 (WD repeat containing) subunit B 1  SUMO1 activating enzyme subunit 1  signal recognition particle 72kDa  TIP41, TOR signaling pathway regulator-like (S. cerevisiae)  hypothetical protein HSPC152  ADP-ribosylation factor interacting protein 1 (arfaptin 1)  serologically defined colon cancer antigen 3  chloride channel 7  polymerase (RNA) II (DNA directed) polypeptide H  myosin regulatory light chain MRLC2  arginyl-tRNA synthetase 2, mitochondrial  dynein, axonemal, light chain 4  hypothetical LOC440589  X-prolyl aminopeptidase (aminopeptidase P) 1, soluble  myotrophin  ribosomal protein S12  Hermansky-Pudlak syndrome 5  nicalin homolog (zebrafish)  general transcription factor IIH, polypeptide 3, 34kDa  RNA binding motif protein 4  golgi autoantigen, golgin subfamily a, 5  cancer susceptibility candidate 4  WAS protein family, member 3  catenin (cadherin-associated protein), alpha 1, 102kDa  lactamase, beta  B-cell receptor-associated protein 31  CDK2-associated protein 1  pantothenate kinase 2 (Hallervorden-Spatz syndrome)  unc-45 homolog A (C. elegans)  protein phosphatase 4 (formerly X), catalytic subunit  zinc finger, ZZ-type containing 3  gem (nuclear organelle) associated protein 6  chromosome 14 open reading frame 135  transmembrane protein 117  pleckstrin homology domain containing, family O member 1  chromosome 9 open reading frame 19  cytochrome P450, family 2, subfamily U, polypeptide 1  UDP-Gal:betaGal beta 1,3-galactosyltransferase polypeptide 6  toll-like receptor adaptor molecule 1  adenosine deaminase, RNA-specific  15 kDa selenoprotein  chromosome 20 open reading frame 3  actin related protein 2/3 complex, subunit 1A, 41kDa  PHD finger protein 11  SH3 domain binding glutamic acid-rich protein like  glycolipid transfer protein  programmed cell death 6  chromosome 20 open reading frame 117  ubiquitin-conjugating enzyme E2I (UBC9 homolog, yeast)  peroxisomal biogenesis factor 16  SAP30-like  transmembrane and coiled-coil domains 1  serine/threonine kinase 4  glycosylphosphatidylinositol anchor attachment protein 1 homolog (yeast)  tRNA splicing endonuclease 34 homolog (S. cerevisiae)  transmembrane 9 superfamily member 3  blocked early in transport 1 homolog (S. cerevisiae)-like  adaptor-related protein complex 2, sigma 1 subunit  beta-2-microglobulin  BUD31 homolog (S. cerevisiae)  pleckstrin homology-like domain, family B, member 1  trafficking protein particle complex 3  solute carrier family 35, member B2  ribosomal protein S19  proline-rich coiled-coil 1  phosphoribosylaminoimidazole carboxylase, phosphoribosylaminoimidazole succinocarboxamide synthetase  Yip1 interacting factor homolog A (S. cerevisiae)  transmembrane protein 184B  COMM domain containing 2  ribosomal protein, large, P0  defender against cell death 1  N-acetyltransferase 9  adenine phosphoribosyltransferase  transporter 1, ATP-binding cassette, sub-family B (MDR/TAP)  guanine nucleotide binding protein (G protein), beta polypeptide 1  seryl-tRNA synthetase  protein O-fucosyltransferase 1  SEC31 homolog A (S. cerevisiae)  IQ motif containing GTPase activating protein 1  WD repeat domain 1  actin-like 6A  mitogen-activated protein kinase 1 interacting protein 1-like  nucleolar protein family A, member 3 (H/ACA small nucleolar RNPs)  polymerase I and transcript release factor  sorting nexin 33  centrin, EF-hand protein, 2  drebrin-like  non imprinted in Prader-Willi/Angelman syndrome 2  guanine nucleotide binding protein-like 1  chromosome 9 open reading frame 30  thioredoxin domain containing 17  zinc metallopeptidase (STE24 homolog, S. cerevisiae)  caprin family member 2  non-metastatic cells 4, protein expressed in  sterile alpha motif domain containing 9-like  v-ral simian leukemia viral oncogene homolog A (ras related)  thioredoxin domain containing 1  misato homolog 1 (Drosophila)  peptidylprolyl isomerase (cyclophilin)-like 5  ATPase, H+ transporting, lysosomal 9kDa, V0 subunit e1  zinc finger protein 226  ankyrin repeat domain 13A  leucine-rich repeats and calponin homology (CH) domain containing 2  coatomer protein complex, subunit gamma  translocator protein (18kDa)  prion protein (p27-30) (Creutzfeldt-Jakob disease, Gerstmann-Strausler-Scheinker syndrome, fatal familial insomnia)  proteasome (prosome, macropain) assembly chaperone 1  catechol-O-methyltransferase  protein arginine methyltransferase 2  cortactin  LIM domain kinase 1  male-enhanced antigen 1  c-abl oncogene 1, receptor tyrosine kinase  Yip1 domain family, member 5  hypothetical protein LOC348262  EH domain binding protein 1  KTEL (Lys-Tyr-Glu-Leu) containing 1  ceroid-lipofuscinosis, neuronal 5  transmembrane protein 183A  chromosome 17 open reading frame 45  DTW domain containing 1  adaptor-related protein complex 3, beta 1 subunit  transforming growth factor beta 1 induced transcript 1  ribophorin I  zinc finger, MYM-type 6  cyclin-dependent kinase 4  septin 9  TAF2 RNA polymerase II, TATA box binding protein (TBP)-associated factor, 150kDa  golgi apparatus protein 1  asparagine-linked glycosylation 3 homolog (S. cerevisiae, alpha-1,3-mannosyltransferase)  N/A  hypothetical protein FLJ35220  sialidase 1 (lysosomal sialidase)  transmembrane 9 superfamily member 1  fibronectin type III domain containing 3B  rhomboid domain containing 2  ADP-ribosylation factor 3  glucosamine (N-acetyl)-6-sulfatase (Sanfilippo disease IIID)  myosin, light chain 6, alkali, smooth muscle and non-muscle  ubiquitin-conjugating enzyme E2Z  low density lipoprotein receptor-related protein 11  CD81 molecule  protein phosphatase 1, regulatory (inhibitor) subunit 14B  staphylococcal nuclease and tudor domain containing 1  milk fat globule-EGF factor 8 protein  NAD(P) dependent steroid dehydrogenase-like  Fas (TNFRSF6)-associated via death domain  S100P binding protein  OCIA domain containing 2  zinc finger protein 329  dCMP deaminase  solute carrier family 25 (mitochondrial carrier; adenine nucleotide translocator), member 5  solute carrier family 25 (mitochondrial carrier; adenine nucleotide translocator), member 6  leucine rich repeat containing 32  tumor necrosis factor receptor superfamily, member 1A  lysosomal-associated membrane protein 1  neuroligin 2  TBP-like 1  mitogen-activated protein kinase kinase kinase kinase 5  dynein, light chain, LC8-type 1  phosphoribosyl transferase domain containing 1  ER lipid raft associated 1  actin, beta  drebrin 1  mannose-6-phosphate receptor binding protein 1  cornichon homolog (Drosophila)  chromosome 12 open reading frame 24  clathrin, light chain (Lca)  polymerase (RNA) III (DNA directed) polypeptide H (22.9kD)  interferon induced transmembrane protein 2 (1-8D)  translocation associated membrane protein 1  integrin, beta 1 (fibronectin receptor, beta polypeptide, antigen CD29 includes MDF2, MSK12)  ubiquitin-conjugating enzyme E2E 2 (UBC4/5 homolog, yeast)  ribosomal protein S27-like  degenerative spermatocyte homolog 1, lipid desaturase (Drosophila)  oxysterol binding protein-like 8  myelin protein zero-like 1  surfeit 4  major facilitator superfamily domain containing 1  N-acetylglucosamine kinase  podoplanin  mevalonate (diphospho) decarboxylase  interleukin 4 receptor  solute carrier family 17 (anion/sugar transporter), member 5  small EDRK-rich factor 2  eukaryotic translation elongation factor 1 alpha 1  exostoses (multiple) 2  similar to Deoxythymidylate kinase (thymidylate kinase)  tyrosyl-tRNA synthetase  transcription elongation factor A (SII)-like 3  splicing factor, arginine/serine-rich 9  REX2, RNA exonuclease 2 homolog (S. cerevisiae)  elongation of very long chain fatty acids (FEN1/Elo2, SUR4/Elo3, yeast)-like 1  tubby like protein 3  plexin A1  mediator complex subunit 19  dolichyl-diphosphooligosaccharide-protein glycosyltransferase  zinc finger CCCH-type, antiviral 1  protein kinase C substrate 80K-H  Rab acceptor 1 (prenylated)  major facilitator superfamily domain containing 10  mannosidase, alpha, class 2B, member 2  glia maturation factor, beta  solute carrier family 39 (metal ion transporter), member 11  kelch domain containing 5  ring finger protein 135  solute carrier family 38, member 6  asparagine-linked glycosylation 8 homolog (S. cerevisiae, alpha-1,3-glucosyltransferase)  F-box protein 5  armadillo repeat containing, X-linked 1  centrosomal protein 290kDa  lamin A/C  canopy 4 homolog (zebrafish)  sphingosine-1-phosphate phosphatase 1  low density lipoprotein receptor-related protein 10  cytochrome c oxidase subunit VIa polypeptide 1  stromal cell-derived factor 2-like 1  chromosome 19 open reading frame 48  jumonji, AT rich interactive domain 1B  chromosome 12 open reading frame 23  canopy 2 homolog (zebrafish)  CLPTM1-like  serine hydroxymethyltransferase 2 (mitochondrial)  laminin, gamma 1 (formerly LAMB2)  chromosome 14 open reading frame 45  solute carrier family 25 (mitochondrial carrier; phosphate carrier), member 24  RAB8A, member RAS oncogene family  interferon induced transmembrane protein 3 (1-8U)  nucleobindin 2  LIM and SH3 protein 1  CD151 molecule (Raph blood group)  LAG1 homolog, ceramide synthase 5  actin related protein 2/3 complex, subunit 5, 16kDa  calponin 2  leucine zipper, down-regulated in cancer 1  caspase 3, apoptosis-related cysteine peptidase  AXL receptor tyrosine kinase  RAB23, member RAS oncogene family  dermatan sulfate epimerase  stromal cell derived factor 4  sarcoglycan, epsilon  melanoma antigen family D, 1  guanylate binding protein 1, interferon-inducible, 67kDa  3-hydroxy-3-methylglutaryl-Coenzyme A reductase  synapse defective 1, Rho GTPase, homolog 1 (C. elegans)  ATPase, Na+/K+ transporting, beta 3 polypeptide  signal sequence receptor, alpha (translocon-associated protein alpha)  ribosomal protein L3  ras homolog gene family, member C  BCL2/adenovirus E1B 19kDa interacting protein 3-like  cofilin 1 (non-muscle)  similar to Ubiquitin-conjugating enzyme E2S (Ubiquitin-conjugating enzyme E2-24 kDa) (Ubiquitin-protein ligase) (Ubiquitin carrier protein) (E2-EPF5)  family with sequence similarity 46, member A  CD99 molecule  unc-119 homolog (C. elegans)  CKLF-like MARVEL transmembrane domain containing 6  exportin 6  heat shock protein 90kDa beta (Grp94), member 1  integrin, alpha V (vitronectin receptor, alpha polypeptide, antigen CD51)  neuron navigator 1  general transcription factor IIE, polypeptide 2, beta 34kDa  regulator of chromosome condensation 2  STT3, subunit of the oligosaccharyltransferase complex, homolog A (S. cerevisiae)  serine carboxypeptidase 1  eukaryotic translation initiation factor 4A, isoform 1  zinc finger protein 668  procollagen-proline, 2-oxoglutarate 4-dioxygenase (proline 4-hydroxylase), beta polypeptide  ATPase, H+ transporting, lysosomal accessory protein 2  zinc finger, DHHC-type containing 24  solute carrier family 35, member B3  La ribonucleoprotein domain family, member 6  macrophage migration inhibitory factor (glycosylation-inhibiting factor)  phosphoglucomutase 3  Sec61 alpha 1 subunit (S. cerevisiae)  tyrosylprotein sulfotransferase 1  cysteine-rich hydrophobic domain 2  teashirt zinc finger homeobox 3  Niemann-Pick disease, type C2  Holliday junction recognition protein  hexosaminidase B (beta polypeptide)  small trans-membrane and glycosylated protein  capping protein (actin filament), gelsolin-like  rho/rac guanine nucleotide exchange factor (GEF) 2  transmembrane protein 138  CD63 molecule  lymphotoxin beta receptor (TNFR superfamily, member 3)  related RAS viral (r-ras) oncogene homolog  laminin, beta 1  abhydrolase domain containing 12  TGFB-induced factor homeobox 1  xenotropic and polytropic retrovirus receptor  PRELI domain containing 1  complement component 1, r subcomponent  sperm autoantigenic protein 17  phosphoprotein enriched in astrocytes 15  phosphorylase, glycogen; brain  protein O-fucosyltransferase 2  signal sequence receptor, delta (translocon-associated protein delta)  echinoderm microtubule associated protein like 4  heat shock 70kDa protein 5 (glucose-regulated protein, 78kDa)  thyroid hormone receptor interactor 6  syntaxin 3  spindlin family, member 4  ADP-ribosylation factor 4  sulfatase modifying factor 1  Tctex1 domain containing 2  coiled-coil domain containing 109B  chromosome 6 open reading frame 48  SHC (Src homology 2 domain containing) transforming protein 1  solute carrier family 30 (zinc transporter), member 7  follistatin-like 1  CDC42 effector protein (Rho GTPase binding) 1  collagen, type III, alpha 1  non-metastatic cells 7, protein expressed in (nucleoside-diphosphate kinase)  transmembrane protein 98  collagen, type VI, alpha 2  hypoxia up-regulated 1  ribosomal protein L23a  FYN oncogene related to SRC, FGR, YES  granulin  neuropilin 1  armadillo repeat containing, X-linked 2  glycoprotein (transmembrane) nmb  matrix metallopeptidase 23A (pseudogene)  chromosome 18 open reading frame 10  ras homolog gene family, member G (rho G)  transmembrane emp24 protein transport domain containing 9  tripartite motif-containing 22  N-acetyltransferase 14  zyxin  opioid receptor, sigma 1  arginine-rich, mutated in early stage tumors  phosphatidylinositol glycan anchor biosynthesis, class O  spermatogenesis associated 2-like  thymosin beta 10  solute carrier family 20 (phosphate transporter), member 1  TRIO and F-actin binding protein  cullin-associated and neddylation-dissociated 1  gamma-glutamyl hydrolase (conjugase, folylpolygammaglutamyl hydrolase)  non-metastatic cells 1, protein (NM23A) expressed in  caspase 4, apoptosis-related cysteine peptidase  chromosome 20 open reading frame 59  enolase 1, (alpha)  Tax1 (human T-cell leukemia virus type I) binding protein 3  midline 1 (Opitz/BBB syndrome)  coatomer protein complex, subunit beta 2 (beta prime)  Smg-6 homolog, nonsense mediated mRNA decay factor (C. elegans)  neuroblastoma RAS viral (v-ras) oncogene homolog  Ellis van Creveld syndrome  ATP citrate lyase  phosphoglycerate mutase family member 4  solute carrier family 30 (zinc transporter), member 5  chromosome 4 open reading frame 34  interferon, gamma-inducible protein 16  cyclin E2  major vault protein  schlafen family member 12  membrane bound O-acyltransferase domain containing 7  transmembrane protein 14A  fascin homolog 1, actin-bundling protein (Strongylocentrotus purpuratus)  reticulocalbin 1, EF-hand calcium binding domain  6-phosphogluconolactonase  SKI-like oncogene  caldesmon 1  ATX1 antioxidant protein 1 homolog (yeast)  anthrax toxin receptor 2  collagen, type VI, alpha 3  tectonic family member 3  small nuclear RNA activating complex, polypeptide 2, 45kDa  RAB31, member RAS oncogene family  polo-like kinase 4 (Drosophila)  histone cluster 1, H2bk  calumenin  suppressor of cytokine signaling 1  UDP-glucose ceramide glucosyltransferase-like 2  myosin IXB  EGF-containing fibulin-like extracellular matrix protein 2  KDEL (Lys-Asp-Glu-Leu) endoplasmic reticulum protein retention receptor 2  thymopoietin  fibroblast growth factor 1 (acidic)  WAS/WASL interacting protein family, member 2  protein disulfide isomerase family A, member 4  parvin, alpha  cathepsin A  lysosomal-associated membrane protein 2  chloride intracellular channel 3  death-associated protein  family with sequence similarity 38, member A  nerve growth factor receptor (TNFRSF16) associated protein 1  ribophorin II  cytoskeleton-associated protein 4  protein arginine methyltransferase 6  fasciculation and elongation protein zeta 1 (zygin I)  transgelin  thioredoxin domain containing 5  tripartite motif-containing 5  xylulokinase homolog (H. influenzae)  vitamin K epoxide reductase complex, subunit 1  inositol 1,4,5-trisphosphate 3-kinase A  family with sequence similarity 14, member A  suppression of tumorigenicity 7 like  tubulin, alpha 1c  NAD(P)H dehydrogenase, quinone 1  A kinase (PRKA) anchor protein 10  transmembrane protein 97  transmembrane protein 44  solute carrier family 10 (sodium/bile acid cotransporter family), member 7  G protein-coupled receptor 137B  F-box protein 4  Alport syndrome, mental retardation, midface hypoplasia and elliptocytosis chromosomal region, gene 1  peroxiredoxin 4  collagen, type I, alpha 2  asparagine-linked glycosylation 2 homolog (S. cerevisiae, alpha-1,3-mannosyltransferase)  lymphocyte antigen 96  cyclin-dependent kinase 6  inositol 1,4,5-triphosphate receptor, type 3  olfactomedin-like 3  bone morphogenetic protein 1  ferritin, light polypeptide  integrin, beta 5  disabled homolog 2, mitogen-responsive phosphoprotein (Drosophila)  collagen, type VI, alpha 1  enhancer of zeste homolog 2 (Drosophila)  RAB34, member RAS oncogene family  midkine (neurite growth-promoting factor 2)  exostoses (multiple) 1  internexin neuronal intermediate filament protein, alpha  receptor-interacting serine-threonine kinase 2  adrenomedullin  hyaluronan-mediated motility receptor (RHAMM)  nudix (nucleoside diphosphate linked moiety X)-type motif 5  IMP (inosine monophosphate) dehydrogenase 1  dual specificity phosphatase 14  chromosome 5 open reading frame 34  integrin, alpha 5 (fibronectin receptor, alpha polypeptide)  SCL/TAL1 interrupting locus  solute carrier family 6 (neurotransmitter transporter, glycine), member 9  forkhead box D1  transketolase (Wernicke-Korsakoff syndrome)  tumor protein p53 inducible protein 3  RAB32, member RAS oncogene family  sterol-C4-methyl oxidase-like  SH3 domain binding glutamic acid-rich protein like 3  DC2 protein  acyl-CoA thioesterase 7  protein disulfide isomerase family A, member 6  fibronectin type III domain containing 4  peptidylprolyl isomerase B (cyclophilin B)  chondroitin polymerizing factor  phosphatidylinositol glycan anchor biosynthesis, class K  V-set and transmembrane domain containing 2 like  annexin A2  phosphatidic acid phosphatase type 2 domain containing 1B  Na+/H+ exchanger domain containing 2  extracellular matrix protein 1  leucine rich repeat containing 49  HERV-H LTR-associating 3  discoidin, CUB and LCCL domain containing 2  nascent polypeptide-associated complex alpha subunit  family with sequence similarity 129, member B  GEM interacting protein  ATP-binding cassette, sub-family C (CFTR/MRP), member 3  microtubule-associated protein 1B  YdjC homolog (bacterial)  cerebellar degeneration-related protein 2-like  SH3 and PX domains 2B  chromosome 1 open reading frame 102  arylsulfatase B  dipeptidyl-peptidase 4 (CD26, adenosine deaminase complexing protein 2)  solute carrier family 22, member 18  zinc finger, matrin type 3  hydroxyacylglutathione hydrolase-like  myosin VA (heavy chain 12, myoxin)  cutA divalent cation tolerance homolog (E. coli)  solute carrier family 25 (mitochondrial carrier; peroxisomal membrane protein, 34kDa), member 17  zinc finger protein 365  S100 calcium binding protein A11  sushi-repeat-containing protein, X-linked 2  microtubule associated serine/threonine kinase-like  tectonic family member 1  phosphatidylinositol 4-kinase type 2 beta  chromosome 19 open reading frame 10  G protein-coupled receptor 172A  protein disulfide isomerase family A, member 5  IKK interacting protein  collagen, type V, alpha 1  HtrA serine peptidase 1  sulfatase modifying factor 2  phosphoglycerate dehydrogenase  FK506 binding protein 10, 65 kDa  UL16 binding protein 1  CAP-GLY domain containing linker protein 3  centromere protein M  chondroitin sulfate synthase 3  lysyl oxidase-like 1  tubulin tyrosine ligase-like family, member 1  versican  GLI pathogenesis-related 1 (glioma)  CD248 molecule, endosialin  TBC1 domain family, member 8B (with GRAM domain)  SEC14-like 2 (S. cerevisiae)  glucose-6-phosphate dehydrogenase  glutaminyl-peptide cyclotransferase (glutaminyl cyclase)  hyaluronoglucosaminidase 3  snail homolog 2 (Drosophila)  tumor necrosis factor receptor superfamily, member 10b  cerebellar degeneration-related protein 2, 62kDa  chromosome 14 open reading frame 79  TATA box binding protein (TBP)-associated factor, RNA polymerase I, A, 48kDa  reticulocalbin 3, EF-hand calcium binding domain  adrenomedullin 2  elongation factor, RNA polymerase II, 2  atlastin 3  cyclin D1  plasminogen activator, urokinase  solute carrier family 44, member 3  solute carrier family 7 (cationic amino acid transporter, y+ system), member 1  serpin peptidase inhibitor, clade H (heat shock protein 47), member 1, (collagen binding protein 1)  fibroblast activation protein, alpha  leucine proline-enriched proteoglycan (leprecan) 1  UBX domain containing 6  Thy-1 cell surface antigen  KDEL (Lys-Asp-Glu-Leu) endoplasmic reticulum protein retention receptor 3  DnaJ (Hsp40) homolog, subfamily C, member 3  transforming growth factor, beta-induced, 68kDa  coiled-coil domain containing 80  translocation associated membrane protein 2  fermitin family homolog 1 (Drosophila)  chromosome 11 open reading frame 41  ADP-ribosylation factor-like 13B  cell division cycle 7 homolog (S. cerevisiae)  TBC1 domain family, member 2  procollagen-proline, 2-oxoglutarate 4-dioxygenase (proline 4-hydroxylase), alpha polypeptide II  phosphoglucomutase 2  SH3KBP1 binding protein 1  chromosome 2 open reading frame 37  signal transducer and activator of transcription 1, 91kDa  RAB33A, member RAS oncogene family  thyroid hormone receptor interactor 13  cadherin 11, type 2, OB-cadherin (osteoblast)  phosphatidylinositol 4-kinase type 2 alpha  non-metastatic cells 5, protein expressed in (nucleoside-diphosphate kinase)  UDP-N-acteylglucosamine pyrophosphorylase 1-like 1  carboxypeptidase Z  cyclin-dependent kinase inhibitor 2A (melanoma, p16, inhibits CDK4)  vascular endothelial growth factor C  FK506 binding protein 11, 19 kDa  superkiller viralicidic activity 2-like (S. cerevisiae)  histocompatibility (minor) 13  thrombospondin 2  sphingomyelin synthase 2  cerebral endothelial cell adhesion molecule  myosin IE  vitamin D (1,25- dihydroxyvitamin D3) receptor  activated leukocyte cell adhesion molecule  glucosidase, beta; acid (includes glucosylceramidase)  sparc/osteonectin, cwcv and kazal-like domains proteoglycan (testican) 1  transmembrane protein 136  DnaJ (Hsp40) homolog, subfamily C, member 10  serpin peptidase inhibitor, clade E (nexin, plasminogen activator inhibitor type 1), member 2  aspartate beta-hydroxylase domain containing 2  transmembrane emp24 protein transport domain containing 3  centrosomal protein 55kDa  procollagen-lysine, 2-oxoglutarate 5-dioxygenase 2  sodium channel, voltage-gated, type IX, alpha subunit  collagen, type I, alpha 1  serpin peptidase inhibitor, clade B (ovalbumin), member 8  lysyl oxidase-like 3  signal peptidase complex subunit 3 homolog (S. cerevisiae)  neurofascin homolog (chicken)  pecanex-like 2 (Drosophila)  abhydrolase domain containing 2  methylenetetrahydrofolate dehydrogenase (NADP+ dependent) 1-like  regulator of G-protein signaling 17  carbonic anhydrase XII  breast carcinoma amplified sequence 4  F-box protein 22  matrix metallopeptidase 14 (membrane-inserted)  N/A  TIMP metallopeptidase inhibitor 1  chromosome 18 open reading frame 56  PDZ and LIM domain 4  neuronal PAS domain protein 1  collagen triple helix repeat containing 1  UDP-N-acetyl-alpha-D-galactosamine:polypeptide N-acetylgalactosaminyltransferase 12 (GalNAc-T12)  hypoxia-inducible factor 1, alpha subunit (basic helix-loop-helix transcription factor)  glutathione peroxidase 8  armadillo repeat containing 9  bradykinin receptor B1  transmembrane protein 119  chondroitin sulfate glucuronyltransferase  galactosamine (N-acetyl)-6-sulfate sulfatase (Morquio syndrome, mucopolysaccharidosis type IVA)  lipin 2  solute carrier family 1 (neuronal/epithelial high affinity glutamate transporter, system Xag), member 1  insulin-like growth factor 2 mRNA binding protein 3  procollagen-lysine, 2-oxoglutarate 5-dioxygenase 3  fibronectin 1  topoisomerase (DNA) II alpha 170kDa  monooxygenase, DBH-like 1  chromosome 11 open reading frame 70  KDEL (Lys-Asp-Glu-Leu) containing 1  laminin, alpha 1  microfibrillar-associated protein 2  trophoblast glycoprotein  cholesterol 25-hydroxylase  PTK7 protein tyrosine kinase 7  endothelial cell-specific molecule 1  plasminogen activator, urokinase receptor  paraneoplastic antigen MA2  nudix (nucleoside diphosphate linked moiety X)-type motif 11  TIMP metallopeptidase inhibitor 2  solute carrier family 38, member 10  SPOC domain containing 1  alkaline phosphatase, liver/bone/kidney  four jointed box 1 (Drosophila)  chromosome 6 open reading frame 65  thymidylate synthetase  pleckstrin and Sec7 domain containing 3  pyrroline-5-carboxylate reductase 1  carboxypeptidase A4  alanyl (membrane) aminopeptidase (aminopeptidase N, aminopeptidase M, microsomal aminopeptidase, CD13, p150)  family with sequence similarity 176, member A  secretogranin II (chromogranin C)  transmembrane protein 200A  cyclin-dependent kinase inhibitor 1A (p21, Cip1)  regulator of G-protein signaling 4  scrapie responsive protein 1  tubulin, beta 3  stanniocalcin 2  pentraxin-related gene, rapidly induced by IL-1 beta  lysyl oxidase  KIAA1199 | 0  1  1  0  1  1  1  1  1  1  1  0  1  0  1  1  0  0  1  0  1  1  1  0  1  1  1  1  0  1  1  1  1  1  1  1  1  1  0  0  1  1  1  1  1  1  1  0  0  1  0  0  0  1  1  0  1  1  1  1  1  1  0  0  1  0  0  1  1  0  1  0  1  0  1  1  1  1  0  1  0  0  1  0  0  1  0  1  1  1  1  0  1  1  1  1  1  0  0  1  0  0  0  0  1  1  1  1  1  1  0  0  1  1  1  1  0  0  0  1  1  1  1  0  1  0  1  1  1  0  1  0  1  0  1  1  1  1  0  0  1  0  0  1  0  1  0  0  1  0  1  1  1  0  0  0  0  1  0  0  1  0  0  0  1  1  0  1  1  1  0  0  0  1  1  1  0  1  1  0  0  1  1  1  0  1  1  1  1  1  1  1  1  1  1  0  1  0  0  1  0  1  1  0  1  0  0  0  1  1  1  1  1  1  1  1  1  0  1  1  0  0  0  0  0  1  0  0  1  1  0  1  1  1  1  1  1  1  1  0  1  1  1  0  1  1  1  1  1  0  1  0  0  1  0  0  1  1  0  1  1  1  1  0  1  0  0  1  0  1  1  1  0  1  1  0  1  1  1  1  1  1  1  1  1  1  1  1  1  0  1  1  0  1  1  0  0  1  1  1  1  1  1  1  0  0  1  1  0  1  1  0  1  1  1  1  1  1  1  0  1  0  1  1  1  0  1  1  1  1  1  1  1  1  1  1  1  0  1  1  0  0  1  0  1  1  1  1  0  1  1  0  0  1  0  1  1  1  1  1  1  0  0  1  1  1  1  1  0  0  1  1  1  1  1  1  1  1  1  1  1  0  0  0  1  0  0  0  1  0  1  0  1  0  1  1  1  0  1  1  0  1  1  1  0  1  0  0  1  1  1  1  1  0  0  1  1  1  1  0  0  0  1  1  1  1  1  0  1  1  0  1  1  1  1  1  0  0  0  0  1  1  1  1  1  1  0  1  1  1  0  0  1  1  0  1  1  0  1  1  0  1  1  1  1  1  0  1  0  1  0  1  0  0  1  1  1  1  0  1  1  0  1  1  0  0  1  1  1  0  1  1  1  0  1  1  0  0  0  1  1  1  1  0  1  1  1  1  0  1  0  0  0  1  0  1  0  0  1  1  1  1  0  1  1  1  1  0  0  1  1  1  1  0  0  1  1  0  1  1  1  0  1  0  1  0  1  1  0  1  1  1  1  0  1  0  1  1  0  1  0  1  0  1  0  1  1  1  1  1  1  0  0  1  1  0  1  1  1  1  1  0  1  0  1  0  1  0  1  1  1  1  0  0  0  0  0  1  1  0  1  0  0  1  1  0  0  1  1  1  1  0  1  1  0  0  0  0  1  1  1  0  1  1  1  1  1  0  1  1  1  0  1  1  0  0  1  1  0  1  0  1  1  0  1  1  1  0  1  1  0  1  0  0  1  0  1  1  1  1  1  1  1  1  1  0  1  1  0  1  1  1  1  0  1  1  0  0  1  1  1  0  0  1  0  1  0  0  1  1  0  0  0  1  0  1  1  0  0  1  0  1  1  0  1  1  0  0  1  0  1  0  1  0  0  0  1  1  1  0  1  1  0  0  0  1  1  0  1  1  1  1  0  0  1  0  0  0  1  1  0  1  1  1  0  1  1  0  0  1  0  1  0  1  1  1  1  1  1  1  1  1  1  0  1  0  1  0  1  1  0  0  1  0  1  1  1  1  0  0  0  0  1  1  0  1  1  1  0  1  0  0  0  0  0  1  0  1  1  1  0  0  0  0  1  1  0  1  0  1  0  1  0  1  1  0  0  1  0  1  1  0  1  0  0  1  0  0  1  1  1  1  1  1  0  0  1  0  1  1  1  1  1  1  0  0  0  0  0  1  1  1  0  0  0  1  0  1  1  1  0  1  0  1  1  1  0  0  1  0  1  1  0  0  1  0  1  0  1  1  1  1  0  1  1  0  0  1  1  0  0  0  1  0  1  0  1  0  0  0  0  0  0  1  1  0  1  0  0  0  1  0  1  1  0  1  0  0  1  0  1  0  1  0  1  1  0  0  0  1  1  1  0  1  0  1  1  1  1  0  0  1  0  0  0  0  1  1  1  1  1  1  1  1  1  0  1  1  0  0  1  1  1  1  0  1  1  0  1  1  1  0  0  0  0  0  0  1  1  0  0  0  0  1  0  0  1  1  1  0  0  1  0  0  1  1  0  1  0  0  0  0  0  0  1  1  1  1  0  1  0  1  0  0  1  0  0  1  0  0  0  1  1  0  1  1  1  1  1  0  0  1  1  1  0  1  1  0  1  0  0  1  1  0  0  1  0  1  0  1  0  1  0  0  0  1  1  1  1  0  0  0  0  1  1  1  0  0  0  1  1  0  1  0  0  0  0  1  1  0  1  0  1  1  0  0  0  0  1  1  1  0  0  0  0  1  0  1  0  0  1  1  0  1  0  0  0  0  1  1  1  0  0  1  0  0  1  0  0  0  0  1  1  0  0  0  0  0  0  1  0  0  0  1  1  0  1  1  0  1  1  0  1  1  0  0  1  1  0  0  0  0  0  1  0  0  0  0  0  0  0  1  0  0  0  1  0  1  0  1  1  0  0  1  1  0  1  1  0  1  1  1  0  0  0  0  0  0  0  0  0  0  0  0  1  1  0  0  0  0  0  1  0  0  0  0  0  1  1  0  0  0  0  1  0  1 | -5330  -2541  -2391  -1668  -1564  -1529  -1496  -1320  -1128  -869.2  -841.6  -711.9  -608.6  -547.8  -546.7  -504.7  -449.1  -421.9  -366.4  -364.5  -364.5  -338.4  -317.8  -310.3  -298.1  -294.9  -293.3  -290.2  -278.5  -277.4  -276.9  -265.7  -228.6  -217.2  -216.1  -200.5  -197.9  -196.1  -190.9  -190.4  -178.5  -175.8  -164.2  -162.9  -161.9  -160.4  -158.9  -155.6  -152.6  -151.4  -148.3  -146.9  -143.9  -137.9  -129.9  -128.1  -121.4  -118.1  -117.5  -117.1  -116.8  -116.7  -115.3  -115.2  -114.1  -113.5  -111.2  -106.5  -105.4  -104.7  -103.8  -103.7  -102.9  -101.3  -98.45  -97.42  -97.31  -97.24  -96.36  -95.53  -93.75  -92.67  -91.69  -90.85  -90.39  -87.94  -84.92  -84.48  -81.19  -81.18  -80.02  -80.01  -75.82  -75.81  -72.98  -71.94  -71.28  -70.66  -68.80  -68.33  -67.79  -65.11  -63.95  -63.94  -61.25  -59.68  -59.53  -58.47  -58.14  -57.43  -55.33  -54.94  -54.24  -51.63  -50.78  -50.15  -49.85  -48.33  -47.98  -47.42  -46.69  -43.77  -42.28  -41.79  -39.06  -38.61  -38.42  -37.95  -37.54  -37.30  -36.77  -36.56  -36.14  -35.71  -35.54  -35.49  -35.38  -34.43  -33.88  -33.41  -33.39  -33.09  -32.02  -31.97  -31.69  -31.65  -30.84  -30.68  -30.62  -30.50  -30.45  -30.39  -30.15  -29.73  -29.66  -29.52  -29.29  -29.10  -29.00  -28.86  -28.40  -28.23  -28.06  -27.86  -27.51  -27.23  -26.71  -26.71  -26.27  -25.32  -25.19  -23.99  -23.87  -23.56  -23.24  -23.10  -22.92  -22.45  -22.31  -22.29  -21.76  -21.39  -20.50  -20.49  -20.21  -20.17  -19.98  -19.92  -19.32  -18.90  -18.86  -18.47  -18.32  -18.20  -18.16  -17.98  -17.78  -17.56  -17.53  -17.43  -17.42  -17.40  -16.80  -16.75  -16.73  -16.72  -16.57  -16.45  -16.41  -15.99  -15.79  -15.52  -15.44  -15.26  -15.12  -15.03  -14.93  -14.93  -14.09  -14.05  -14.01  -13.88  -13.82  -13.81  -13.70  -13.57  -13.48  -13.37  -13.20  -12.98  -12.91  -12.56  -12.51  -12.48  -12.33  -12.26  -12.09  -12.06  -11.68  -11.54  -11.44  -11.35  -11.12  -11.09  -11.08  -11.06  -11.02  -11.00  -10.84  -10.75  -10.71  -10.65  -10.55  -10.54  -10.41  -10.35  -10.33  -10.19  -10.05  -9.94  -9.89  -9.82  -9.72  -9.67  -9.62  -9.62  -9.57  -9.40  -9.36  -9.36  -9.32  -8.96  -8.93  -8.71  -8.69  -8.66  -8.50  -8.49  -8.43  -8.38  -8.30  -8.26  -8.21  -8.17  -8.14  -8.06  -8.04  -7.95  -7.88  -7.83  -7.71  -7.59  -7.51  -7.47  -7.39  -7.34  -7.26  -7.24  -7.18  -7.16  -7.14  -7.11  -7.07  -6.92  -6.91  -6.91  -6.80  -6.80  -6.69  -6.54  -6.53  -6.50  -6.48  -6.43  -6.40  -6.36  -6.32  -6.32  -6.30  -6.29  -6.23  -6.22  -6.20  -6.16  -6.13  -6.08  -6.06  -6.05  -5.96  -5.95  -5.95  -5.94  -5.89  -5.89  -5.87  -5.84  -5.76  -5.72  -5.64  -5.59  -5.58  -5.52  -5.51  -5.50  -5.47  -5.47  -5.42  -5.38  -5.36  -5.34  -5.33  -5.32  -5.30  -5.29  -5.27  -5.17  -5.09  -5.07  -5.06  -5.05  -5.02  -4.93  -4.90  -4.89  -4.89  -4.86  -4.86  -4.84  -4.78  -4.78  -4.77  -4.75  -4.75  -4.71  -4.66  -4.63  -4.58  -4.55  -4.53  -4.52  -4.51  -4.50  -4.49  -4.48  -4.47  -4.47  -4.43  -4.38  -4.36  -4.34  -4.33  -4.32  -4.30  -4.27  -4.26  -4.21  -4.16  -4.15  -4.12  -4.08  -4.08  -4.05  -4.04  -4.02  -4.01  -4.00  -3.95  -3.94  -3.94  -3.90  -3.75  -3.75  -3.75  -3.74  -3.70  -3.70  -3.67  -3.67  -3.66  -3.65  -3.65  -3.61  -3.58  -3.58  -3.57  -3.53  -3.53  -3.52  -3.51  -3.48  -3.47  -3.47  -3.42  -3.41  -3.40  -3.39  -3.39  -3.39  -3.37  -3.36  -3.35  -3.34  -3.32  -3.32  -3.31  -3.31  -3.30  -3.28  -3.27  -3.27  -3.26  -3.26  -3.25  -3.25  -3.23  -3.23  -3.22  -3.20  -3.18  -3.16  -3.13  -3.13  -3.13  -3.11  -3.11  -3.11  -3.11  -3.11  -3.10  -3.10  -3.09  -3.09  -3.09  -3.08  -3.07  -3.05  -3.05  -3.05  -3.04  -3.04  -3.04  -3.03  -3.02  -3.02  -3.01  -3.00  -2.98  -2.98  -2.98  -2.98  -2.98  -2.97  -2.95  -2.93  -2.93  -2.93  -2.92  -2.92  -2.91  -2.90  -2.89  -2.86  -2.83  -2.83  -2.80  -2.80  -2.80  -2.78  -2.75  -2.74  -2.74  -2.73  -2.72  -2.71  -2.70  -2,70  -2.69  -2.66  -2.64  -2.64  -2.64  -2.63  -2.62  -2.62  -2.61  -2.60  -2.59  -2.57  -2.55  -2.55  -2.54  -2.54  -2.54  -2.54  -2.54  -2.53  -2.52  -2.51  -2.51  -2.47  -2.47  -2.47  -2.47  -2.45  -2.43  -2.43  -2.41  -2.37  -2.37  -2.36  -2.36  -2.35  -2.35  -2.35  -2.35  -2.33  -2.33  -2.30  -2.28  -2.28  -2.27  -2.26  -2.26  -2.22  -2.20  -2.19  -2.18  -2.18  -2.17  -2.17  -2.16  -2.16  -2.16  -2.14  -2.14  -2.14  -2.14  -2.12  -2.11  -2.08  -2.05  -2.05  -2.04  2.02  2.02  2.02  2.04  2.05  2.06  2.06  2.07  2.07  2.09  2.12  2.12  2.12  2.14  2.17  2.17  2.18  2.18  2.18  2.19  2.19  2.19  2.20  2.20  2.21  2.23  2.24  2.25  2.26  2.27  2.28  2.28  2.29  2.31  2.33  2.33  2.33  2.34  2.34  2.35  2.36  2.37  2.37  2.37  2.38  2.39  2.39  2.40  2.40  2.40  2.41  2.42  2.42  2.44  2.44  2.44  2.44  2.45  2.45  2.45  2.45  2.46  2.46  2.47  2.49  2.50  2.50  2.51  2.53  2.55  2.55  2.56  2.56  2.57  2.57  2.57  2.60  2.61  2.63  2.63  2.64  2.65  2.66  2.69  2.69  2.70  2.70  2.72  2.72  2.75  2.75  2.78  2.80  2.80  2.81  2.83  2.83  2.83  2.84  2.85  2.85  2.85  2.85  2.86  2.86  2.87  2.87  2.89  2.89  2.91  2.92  2.92  2.93  2.93  2.95  2.95  2.95  2.95  2.96  2.98  3.00  3.01  3.01  3.02  3.03  3.03  3.04  3.05  3.05  3.06  3.06  3.07  3.08  3.08  3.09  3.11  3.14  3.14  3.14  3.15  3.15  3.16  3.16  3.16  3.17  3.17  3.18  3.19  3.20  3.21  3.21  3.22  3.23  3.23  3.25  3.25  3.25  3.27  3.28  3.28  3.29  3.29  3.30  3.31  3.31  3.32  3.34  3.37  3.37  3.37  3.38  3.39  3.40  3.41  3.43  3.44  3.46  3.47  3.48  3.49  3.50  3.50  3.52  3.54  3.55  3.56  3.56  3.59  3.59  3.60  3.64  3.65  3.67  3.67  3.67  3.68  3.68  3.69  3.70  3.73  3.73  3.74  3.75  3.77  3.77  3.78  3.79  3.79  3.81  3.81  3.82  3.82  3.82  3.84  3.85  3.85  3.86  3.87  3.87  3.95  3.96  3.97  4.00  4.00  4.00  4.01  4.01  4.05  4.07  4.07  4.08  4.08  4.09  4.11  4.12  4.12  4.12  4.13  4.14  4.15  4.16  4.18  4.18  4.18  4.19  4.21  4.21  4.22  4.23  4.23  4.24  4.24  4.26  4.27  4.29  4.30  4.31  4.31  4.32  4.38  4.40  4.40  4.42  4.43  4.44  4.44  4.46  4.48  4.48  4.49  4.49  4.49  4.50  4.50  4.53  4.54  4.55  4.56  4.63  4.64  4.68  4.68  4.69  4.69  4.70  4.70  4.73  4.73  4.74  4.75  4.77  4.78  4.79  4.79  4.81  4.83  4.83  4.86  4.89  4.90  4.91  4.95  4.96  4.98  4.98  5.00  5.02  5.03  5.04  5.05  5.08  5.10  5.13  5.13  5.13  5.13  5.14  5.15  5.18  5.18  5.19  5.22  5.23  5.25  5.27  5.31  5.33  5.34  5.34  5.39  5.39  5.39  5.44  5.45  5.45  5.47  5.53  5.55  5.65  5.66  5.69  5.71  5.80  5.83  5.84  5.86  5.87  5.91  5.94  5.94  5.95  5.96  5.97  6.01  6.02  6.04  6.05  6.08  6.17  6.19  6.23  6.29  6.34  6.37  6.42  6.46  6.47  6.47  6.49  6.53  6.55  6.56  6.56  6.56  6.66  6.66  6.70  6.70  6.80  6.89  6.90  6.91  6.95  6.99  7.00  7.00  7.10  7.12  7.16  7.17  7.20  7.20  7.21  7.26  7.27  7.28  7.44  7.54  7.54  7.56  7.59  7.61  7.63  7.67  7.69  7.71  7.85  7.86  7.91  8.01  8.12  8.13  8.19  8.19  8.20  8.24  8.26  8.27  8.28  8.28  8.33  8.38  8.53  8.60  8.69  8.87  8.91  9.04  9.04  9.06  9.12  9.13  9.22  9.23  9.28  9.31  9.32  9.33  9.35  9.36  9.39  9.45  9.54  9.56  9.58  9.62  9.68  9.71  9.75  9.82  9.92  10.01  10.12  10.14  10.19  10.20  10.23  10.26  10.26  10.31  10.31  10.59  10.72  10.75  10.89  10.95  11.13  11.17  11.18  11.19  11.46  11.50  11.52  11.54  11.65  11.90  12.42  12.47  12.49  12.55  12.60  12.72  12.72  12.72  12.75  12.83  12.92  12.96  13.03  13.05  13.07  13.17  13.39  13.49  13.49  13.68  13.76  13.79  13.87  13.92  14.19  14.39  14.47  14.62  14.81  14.87  14.88  15.09  15.11  15.28  15.40  15.48  15.51  15.59  15.96  16.05  16.05  16.09  16.27  16.38  16.43  16.60  16.67  16.79  16.81  16.90  17.06  17.17  17.23  17.27  17.29  17.45  17.66  18.09  18.11  18.25  18.28  18.63  18.91  19.05  19.39  19.48  19.61  20.27  20.89  20.91  21.14  21.43  22.28  22.35  22.59  22.63  22.74  23.46  23.83  24.09  24.33  24.35  24.41  24.52  24.59  24.74  24.89  25.73  25.77  25.84  25.91  26.26  26.35  26.40  26.53  26.60  27.31  27.98  28.06  28.59  28.64  29.68  29.75  29.93  30.00  31.51  31.90  33.17  33.74  34.02  34.89  35.90  36.17  36.70  38.12  40.00  40.78  40.92  40.97  41.22  43.02  43.40  43.58  46.22  46.50  46.60  46.92  49.95  50.43  57.62  61.03  62.81  64.82  66.23  66.47  68.10  68.69  71.10  74.10  75.99  79.49  81.49  82.63  85.98  88.56  94.67  164.1  178.7  184.9  192.8  199.3  294.6  349.4  438.4  491.7  878.9  1279 |
