# Supplementary material for: Comparative gene expression profiling between human cultured myotubes and skeletal muscle tissue
Source: BMC Genomics. 2010 Feb 22;11:125. doi: 10.1186/1471-2164-11-125 (PMC2838843; doi:10.1186/1471-2164-11-125)
Supplement: Additional file 4 — Table S2. Most significantly regulated gene ontologies. List of gene ontologies annotated from (A) the whole set or (B) the subset filtered by expression in SM, on the basis of the IPA knowledge-base, of downregulated and upregulated transcripts in cultured myotubes compared to the SM tissue. We used the GO database http://www.geneontology.org with the GeneSpring GX software. GeneSpring GX calculated enrichment scores for GO terms based on the list of regulated genes, and used enrichment scores and Benjamini-Yekutieli (False Discovery Rate) corrected p-values to filter the set of genes. GO terms that are enriched with a p-value cut-off of 0.1 are shown. Less specific nodes in the GO hierarchy that contained the same annotated genes as the stated most-specific nodes are not shown. The first column lists the GO symbol, the second the GO term, the third the corrected p-value and the fourth the count in the selection. [file 1471-2164-11-125-S4.DOC]

**Table S2A**

| **GO Accession** | **GO Term** | **Corrected p-value** | **Count** |
| --- | --- | --- | --- |
| **DOWNREGULATED**  **Cellular component**  GO:0005622  GO:0005737  GO:0005739  GO:0044429  GO:0031966  GO:0005746  GO:0005759  **Molecular function**  GO:0016491  GO:0016651  GO:0048037  **Biological process**  GO:0008152  GO:0006091  GO:0006120  GO:0045333  GO:0009060  GO:0006099  GO:0006119  GO:0051186  GO:0006084  GO:0042375  GO:0009056  GO:0055114  GO:0003012  GO:0006936  **UPREGULATED**  **Cellular component**  GO:0005737  GO:0044444  GO:0005783  GO:0044432  GO:0005789  GO:0008250  GO:0005793  GO:0005788|GO:0016022  GO:0012505  GO:0042175  GO:0005578  **Molecular function**  GO:0004576  GO:0004579  **Biological process**  GO:0018193  GO:0018196  GO:0018279 | intracellular  cytoplasm  mitochondrion  mitochondrial part  mitochondrial membrane  mitochondrial respiratory chain  mitochondrial matrix  oxidoreductase activity  oxidoreductase activity, acting on NADH or NADPH  cofactor binding  metabolic process  generation of precursor metabolites and energy  mitochondrial electron transport, NADH to ubiquinone  cellular respiration  aerobic respiration  tricarboxylic acid cycle  oxidative phosphorylation  cofactor metabolic process  acetyl-CoA metabolic process  quinone cofactor metabolic process  catabolic process  oxidation reduction  muscle system process  muscle contraction  cytoplasm  cytoplasmic part  endoplasmic reticulum  endoplasmic reticulum part  endoplasmic reticulum membrane  oligosaccharyl transferase complex  ER-Golgi intermediate compartment  endoplasmic reticulum lumen  endomembrane system  nuclear envelope-endoplasmic reticulum network  proteinaceous extracellular matrix  oligosaccharyl transferase activity  dolichyl-diphosphooligosaccharide-protein glycotransferase activity  peptidyl-amino acid modification  peptidyl-asparagine modification  protein amino acid N-linked glycosylation via asparagine | 6.49E-04  5.21E-12  5.53E-30  1.04E-10  2.46E-05  4.70E-04  2.30E-03  2.00E-04  3.51E-02  1.32E-02  5.72E-03  5.21E-12  2.31E-02  1.72E-09  1.71E-06  1.90E-04  8.66E-03  8.89E-06  3.87E-07  2.67E-02  2.36E-02  8.17E-04  9.74E-02  9.74E-02  1.64E-08  2.97E-08  2.04E-20  6.98E-08  4.67E-04  1.76E-03  1.08E-02  1.46E-03  4.82E-02  1.02E-03  9.38E-02  1.08E-02  1.76E-03  3.99E-02  1.76E-03  1.76E-03 | 180  144  94  18  12  5  6  32  11  2  68  28  7  15  12  9  9  16  11  5  9  7  12  12  142  86  83  19  12  6  9  8  12  12  15  6  6  7  6  6 |

**Table S2B**

| **GO Accession** | **GO Term** | **Corrected *p* Value** | **Count** |
| --- | --- | --- | --- |
| **DOWNREGULATED**  **Cellular component**  GO:0005622  GO:0005737  GO:0005739  GO:0044429  GO:0031966  GO:0005746  GO:0005759  **Molecular function**  GO:0016491  GO:0048037  GO:0008092  **Biological process**  GO:0006091  GO:0006120  GO:0045333  GO:0009060  GO:0006099  GO:0006119  GO:0051186  GO:0006084  GO:0042375  GO:0009056  GO:0006635  GO:0006082  GO:0019752  GO:0007517  GO:0003012  GO:0006936  **UPREGULATED**  **Cellular component**  GO:0005737  GO:0044444  GO:0005783  GO:0012505  GO:0005578 | intracellular  cytoplasm  mitochondrion  mitochondrial part  mitochondrial membrane  mitochondrial respiratory chain  mitochondrial matrix  oxidoreductase activity  cofactor binding  cytoskeletal protein binding  generation of precursor metabolites and energy  mitochondrial electron transport, NADH to ubiquinone  cellular respiration  aerobic respiration  tricarboxylic acid cycle  oxidative phosphorylation  cofactor metabolic process  acetyl-CoA metabolic process  quinone cofactor metabolic process  catabolic process  fatty acid beta-oxidation  organic acid metabolic process  carboxylic acid metabolic process  muscle development  muscle system process  muscle contraction  cytoplasm  cytoplasmic part  endoplasmic reticulum  endomembrane system  proteinaceous extracellular matrix | 6.49E-04  5.21E-12  5.53E-30  1.04E-10  2.46E-05  4.70E-04  2.30E-03  2.00E-04  1.32E-02  1.49E-02  5.21E-12  2.31E-02  1.72E-09  1.71E-06  1.90E-04  8.66E-03  8.89E-06  3.87E-07  2.67E-02  2.36E-02  8.76E-02  2.02E-02  1.90E-02  7.15E-02  9.74E-02  9.74E-02  1.64E-08  2.97E-08  2.04E-20  4.82E-02  9.38E-02 | 180  144  94  18  12  5  6  32  2  2  28  7  15  12  9  9  16  11  5  9  5  7  7  9  12  12  142  86  83  12  15 |
