# Supplementary material for: Comparative gene expression profiling between human cultured myotubes and skeletal muscle tissue
Source: BMC Genomics. 2010 Feb 22;11:125. doi: 10.1186/1471-2164-11-125 (PMC2838843; doi:10.1186/1471-2164-11-125)
Supplement: Additional file 5 — Table S3. Microarray data variability of the 10% most skeletal muscle-filtered differentially expressed transcripts. Log2 normalized intensities for each significantly regulated transcript (p < 0.01 and fold change > 2) in each sample. The standard deviation for each condition, in vitro and in vivo, is shown. A normalized intensity value of 0 means that the raw signal was below the background and an arbitrary value of 1 was assigned to enable the log2 transformation (log2(1) = 0). [file 1471-2164-11-125-S5.DOC]

| **Symbol EntrezGene** | | **B19 In vitro** | **B22 In vitro** | **B24 In vitro** | **B25 In vitro** | **B26 In vitro** | **SD**  **In vitro** | **B19 In vivo** | **B22 In vivo** | **B24 In vivo** | **B25 In vivo** | **B26 In vivo** | **SD**  **In vivo** |
| --- | --- | --- | --- | --- | --- | --- | --- | --- | --- | --- | --- | --- | --- |
| HBB | 3043 | 0.00 | 0.00 | 0.00 | 2.26 | 0.00 | 1.01 | 12.87 | 13.17 | 13.30 | 11.80 | 13.03 | 0.60 |
| MB | 4151 | 2.00 | 2.07 | 1.26 | 3.71 | 2.07 | 0.90 | 13.43 | 13.43 | 13.54 | 13.67 | 13.61 | 0.11 |
| CA3 | 761 | 0.00 | 0.00 | 0.00 | 0.00 | 1.81 | 0.81 | 10.45 | 12.25 | 11.61 | 11.38 | 12.23 | 0.74 |
| C20orf166 | 128826 | 0.00 | 1.26 | 0.00 | 0.00 | 0.00 | 0.56 | 11.32 | 11.10 | 10.57 | 10.78 | 11.02 | 0.29 |
| TMOD4 | 29765 | 0.00 | 3.17 | 0.00 | 1.58 | 0.00 | 1.42 | 11.75 | 11.61 | 11.49 | 11.32 | 11.64 | 0.17 |
| MYL3 | 4634 | 2.79 | 4.86 | 0.00 | 1.49 | 0.00 | 2.06 | 12.24 | 12.49 | 11.98 | 12.65 | 12.65 | 0.29 |
| CASQ1 | 844 | 0.00 | 2.46 | 0.38 | 0.00 | 0.00 | 1.07 | 11.63 | 11.13 | 10.88 | 11.31 | 10.62 | 0.39 |
| AMPD1 | 270 | 0.00 | 3.51 | 0.00 | 0.00 | 0.68 | 1.52 | 11.37 | 11.18 | 10.97 | 11.66 | 10.84 | 0.33 |
| MYBPC1 | 4604 | 2.00 | 0.00 | 1.32 | 1.38 | 1.07 | 0.73 | 10.86 | 11.37 | 11.27 | 11.14 | 11.83 | 0.36 |
| ASB10 | 136371 | 0.00 | 0.00 | 0.00 | 0.00 | 0.00 | 0.00 | 9.72 | 9.93 | 9.63 | 9.80 | 9.74 | 0.11 |
| MYOZ3 | 91977 | 1.20 | 0.58 | 0.00 | 1.89 | 0.85 | 0.70 | 10.41 | 10.66 | 10.37 | 10.90 | 10.78 | 0.23 |
| HBA2 | 3040 | 3.71 | 1.77 | 3.51 | 4.86 | 3.09 | 1.12 | 12.65 | 12.89 | 13.81 | 11.89 | 13.07 | 0.69 |
| LPL | 4023 | 0.00 | 0.77 | 0.00 | 0.00 | 1.14 | 0.54 | 9.38 | 9.35 | 9.72 | 9.42 | 10.28 | 0.39 |
| SLC7A2 | 6542 | 0.00 | 1.26 | 0.00 | 0.00 | 0.00 | 0.56 | 9.98 | 9.20 | 9.77 | 8.97 | 8.82 | 0.50 |
| MYOZ1 | 58529 | 3.77 | 4.99 | 2.61 | 2.43 | 4.34 | 1.10 | 13.03 | 12.80 | 12.56 | 12.80 | 12.42 | 0.24 |
| CLIC5 | 53405 | 0.00 | 0.00 | 0.00 | 0.00 | 0.00 | 0.00 | 8.87 | 8.69 | 9.30 | 9.33 | 8.72 | 0.31 |
| SRD5A2L2 | 253017 | 0.00 | 0.00 | 0.00 | 2.29 | 0.00 | 1.03 | 8.42 | 9.76 | 9.06 | 9.46 | 9.64 | 0.54 |
| IDI2 | 91734 | 1.58 | 0.68 | 0.00 | 2.38 | 1.26 | 0.90 | 9.80 | 10.29 | 9.83 | 9.84 | 9.72 | 0.23 |
| S100A1 | 6271 | 0.00 | 3.19 | 0.58 | 0.00 | 0.14 | 1.37 | 9.16 | 9.42 | 9.17 | 9.37 | 9.37 | 0.13 |
| SMTNL2 | 342527 | 0.00 | 0.68 | 0.38 | 1.77 | 0.00 | 0.73 | 9.41 | 8.88 | 9.41 | 9.13 | 8.53 | 0.37 |
| HHATL | 57467 | 0.00 | 1.00 | 0.00 | 1.43 | 0.00 | 0.68 | 8.92 | 9.30 | 9.30 | 8.66 | 8.81 | 0.29 |
| RTN4 | 57142 | 0.00 | 1.20 | 0.00 | 0.00 | 0.00 | 0.54 | 8.01 | 8.92 | 8.89 | 8.64 | 8.76 | 0.37 |
| RPL3L | 6123 | 2.32 | 3.42 | 1.93 | 1.49 | 2.32 | 0.72 | 10.97 | 10.84 | 10.64 | 10.44 | 10.13 | 0.33 |
| SLC36A2 | 153201 | 0.68 | 2.14 | 0.00 | 1.63 | 0.00 | 0.97 | 9.37 | 9.36 | 9.40 | 8.74 | 8.96 | 0.30 |
| ANKRD23 | 200539 | 0.00 | 0.00 | 2.54 | 0.26 | 0.00 | 1.11 | 8.69 | 8.67 | 9.10 | 8.74 | 8.70 | 0.18 |
| NRAP | 4892 | 2.26 | 4.00 | 1.20 | 2.20 | 2.49 | 1.01 | 10.71 | 9.99 | 10.58 | 10.93 | 10.96 | 0.39 |
| FSD2 | 123722 | 0.38 | 2.29 | 1.43 | 0.00 | 1.20 | 0.90 | 9.28 | 9.33 | 9.13 | 9.50 | 9.04 | 0.18 |
| CA2 | 760 | 0.00 | 0.49 | 0.00 | 0.00 | 0.00 | 0.22 | 7.95 | 8.37 | 8.29 | 8.45 | 8.33 | 0.19 |
| C8orf22 | 492307 | 0.00 | 0.00 | 0.00 | 0.00 | 0.00 | 0.00 | 7.36 | 7.98 | 8.31 | 8.50 | 8.45 | 0.47 |
| PYGM | 5837 | 3.67 | 3.79 | 2.85 | 3.14 | 3.05 | 0.41 | 12.05 | 11.22 | 11.45 | 11.43 | 10.92 | 0.42 |
| ABLIM2 | 84448 | 0.00 | 3.36 | 0.00 | 0.00 | 2.07 | 1.56 | 9.32 | 9.49 | 9.26 | 9.18 | 8.76 | 0.27 |
| MYF6 | 4618 | 0.00 | 3.78 | 0.00 | 0.00 | 0.00 | 1.69 | 8.84 | 8.63 | 8.95 | 8.95 | 8.67 | 0.15 |
| ASB12 | 142689 | 0.00 | 1.00 | 0.00 | 0.00 | 1.72 | 0.79 | 8.62 | 8.21 | 8.32 | 8.48 | 8.27 | 0.17 |
| TMEM52 | 339456 | 2.00 | 1.63 | 2.70 | 0.00 | 0.00 | 1.22 | 9.25 | 8.98 | 9.05 | 9.18 | 8.69 | 0.22 |
| XIRP2 | 129446 | 0.00 | 3.14 | 0.00 | 1.72 | 2.54 | 1.44 | 8.16 | 8.91 | 9.02 | 9.57 | 10.51 | 0.87 |
| ANKRD2 | 26287 | 0.00 | 4.05 | 1.54 | 0.00 | 0.38 | 1.72 | 7.84 | 9.73 | 8.62 | 8.09 | 9.92 | 0.95 |
| C1QA | 712 | 0.00 | 0.00 | 0.00 | 0.00 | 0.00 | 0.00 | 7.50 | 7.63 | 8.62 | 6.92 | 7.48 | 0.62 |
| ALDH1L1 | 10840 | 0.00 | 0.93 | 0.00 | 0.00 | 0.00 | 0.41 | 8.20 | 7.62 | 8.47 | 7.26 | 7.45 | 0.51 |
| KLHDC6 | 166348 | 0.00 | 2.58 | 0.00 | 1.26 | 0.00 | 1.15 | 8.47 | 8.21 | 7.84 | 8.64 | 8.57 | 0.32 |
| SLC16A10 | 117247 | 0.00 | 3.93 | 0.00 | 1.38 | 0.00 | 1.71 | 9.49 | 9.20 | 7.72 | 8.85 | 7.91 | 0.78 |
| ITM2A | 9452 | 2.49 | 0.00 | 0.00 | 0.00 | 4.07 | 1.88 | 9.03 | 8.48 | 8.90 | 8.36 | 9.18 | 0.35 |
| SERPINA5 | 5104 | 0.00 | 0.26 | 0.00 | 0.00 | 0.49 | 0.22 | 7.24 | 8.12 | 7.15 | 8.05 | 7.49 | 0.45 |
| CAPN3 | 825 | 1.26 | 4.12 | 4.07 | 1.32 | 3.46 | 1.44 | 10.72 | 10.43 | 9.76 | 10.12 | 10.01 | 0.37 |
| MYOC | 4653 | 0.00 | 0.00 | 0.00 | 2.07 | 1.68 | 1.04 | 7.72 | 7.74 | 8.24 | 7.32 | 9.47 | 0.83 |
| NIPSNAP3B | 55335 | 0.00 | 0.00 | 0.00 | 0.00 | 0.00 | 0.00 | 7.26 | 7.32 | 6.89 | 7.73 | 7.48 | 0.31 |
| AQP4 | 361 | 1.14 | 0.00 | 0.00 | 0.00 | 0.00 | 0.51 | 7.52 | 8.03 | 7.98 | 7.09 | 7.15 | 0.44 |
| MAOB | 4129 | 0.00 | 2.23 | 0.00 | 0.00 | 0.00 | 1.00 | 8.17 | 7.83 | 7.66 | 7.42 | 7.70 | 0.27 |
| GIMAP7 | 168537 | 0.00 | 0.00 | 0.00 | 0.00 | 0.00 | 0.00 | 6.45 | 6.89 | 8.38 | 7.36 | 7.32 | 0.72 |
| C10orf71 | 118461 | 0.58 | 1.93 | 1.43 | 1.07 | 0.00 | 0.75 | 8.00 | 7.69 | 9.05 | 8.21 | 8.34 | 0.51 |
| CPT1B | 1375 | 2.20 | 2.00 | 0.00 | 2.83 | 0.00 | 1.32 | 8.92 | 8.16 | 8.65 | 8.71 | 8.80 | 0.29 |
| DARC | 2532 | 0.00 | 0.00 | 2.07 | 0.00 | 0.00 | 0.93 | 7.54 | 7.00 | 8.74 | 7.67 | 7.18 | 0.68 |
| C9orf164 | 349236 | 1.58 | 2.70 | 1.07 | 0.00 | 1.49 | 0.97 | 9.14 | 8.37 | 8.42 | 8.47 | 8.44 | 0.32 |
| RASL12 | 51285 | 0.00 | 0.00 | 0.00 | 0.00 | 0.00 | 0.00 | 7.18 | 7.10 | 7.09 | 7.75 | 6.72 | 0.37 |
| KCNA7 | 3743 | 0.00 | 0.00 | 1.14 | 0.00 | 2.00 | 0.91 | 7.43 | 7.72 | 8.10 | 7.58 | 7.84 | 0.25 |
| RGS5 | 8490 | 0.00 | 0.93 | 1.32 | 1.85 | 0.00 | 0.82 | 7.92 | 6.74 | 7.81 | 8.41 | 8.33 | 0.67 |
| C1QB | 713 | 0.00 | 0.00 | 0.00 | 0.00 | 0.00 | 0.00 | 7.20 | 6.75 | 8.77 | 5.38 | 6.91 | 1.21 |
| AGTRL1 | 187 | 1.63 | 0.26 | 0.00 | 0.00 | 1.63 | 0.85 | 7.77 | 7.18 | 7.51 | 8.55 | 7.13 | 0.58 |
| TRIM7 | 81786 | 0.00 | 2.20 | 0.00 | 3.78 | 0.00 | 1.73 | 8.06 | 8.13 | 7.62 | 8.50 | 8.08 | 0.31 |
| PRKCQ | 5588 | 3.32 | 3.89 | 2.23 | 0.00 | 3.04 | 1.52 | 9.48 | 9.41 | 9.62 | 9.20 | 9.15 | 0.20 |
| ADSSL1 | 122622 | 3.92 | 4.91 | 4.92 | 3.14 | 4.90 | 0.80 | 11.22 | 11.06 | 11.45 | 11.37 | 11.04 | 0.18 |
| ZBTB16 | 7704 | 1.85 | 1.85 | 3.35 | 2.38 | 2.20 | 0.62 | 10.13 | 8.52 | 9.41 | 8.65 | 9.27 | 0.65 |
| HRASLS | 57110 | 2.58 | 2.70 | 1.00 | 2.85 | 3.15 | 0.84 | 9.46 | 9.54 | 9.24 | 9.54 | 8.85 | 0.29 |
| KLHL34 | 257240 | 0.00 | 0.00 | 0.00 | 0.00 | 0.00 | 0.00 | 5.33 | 6.82 | 7.38 | 6.78 | 7.93 | 0.97 |
| HMGCS2 | 3158 | 1.14 | 0.00 | 0.00 | 1.49 | 0.00 | 0.73 | 7.43 | 6.92 | 8.02 | 7.39 | 7.10 | 0.42 |
| CNTFR | 1271 | 1.49 | 2.04 | 0.00 | 2.54 | 0.00 | 1.17 | 8.64 | 8.06 | 8.81 | 7.35 | 7.38 | 0.68 |
| C7orf53 | 286006 | 0.00 | 1.26 | 0.38 | 1.72 | 2.58 | 1.04 | 8.22 | 7.82 | 7.97 | 8.32 | 7.76 | 0.25 |
| MYH7B | 57644 | 0.00 | 2.93 | 0.49 | 0.00 | 0.00 | 1.27 | 7.12 | 8.17 | 7.02 | 7.49 | 7.59 | 0.45 |
| UCP3 | 7352 | 0.00 | 0.00 | 0.00 | 0.00 | 0.00 | 0.00 | 7.63 | 5.19 | 7.46 | 7.89 | 5.50 | 1.28 |
| CD93 | 22918 | 2.63 | 1.68 | 1.68 | 0.00 | 1.32 | 0.95 | 8.61 | 7.35 | 8.21 | 8.55 | 8.19 | 0.50 |
| CA4 | 762 | 1.63 | 1.54 | 0.00 | 0.00 | 0.00 | 0.87 | 7.66 | 7.06 | 6.51 | 8.03 | 7.46 | 0.59 |
| CIDEA | 1149 | 0.00 | 0.38 | 0.00 | 0.00 | 1.07 | 0.47 | 7.72 | 6.62 | 8.49 | 6.32 | 5.79 | 1.10 |
| CMYA5 | 202333 | 4.67 | 6.82 | 5.30 | 5.72 | 5.97 | 0.80 | 12.58 | 12.17 | 12.34 | 12.43 | 12.45 | 0.15 |
| PTP4A3 | 11156 | 2.32 | 4.52 | 1.89 | 1.07 | 3.09 | 1.31 | 9.30 | 9.27 | 9.58 | 9.12 | 9.04 | 0.21 |
| C9orf61 | 9413 | 0.00 | 3.15 | 0.00 | 0.00 | 0.00 | 1.41 | 7.53 | 7.26 | 7.53 | 7.54 | 6.60 | 0.41 |
| ITGB1BP3 | 27231 | 0.00 | 1.32 | 0.00 | 0.00 | 0.00 | 0.59 | 5.61 | 6.56 | 7.78 | 6.62 | 7.85 | 0.94 |
| FABP4 | 2167 | 2.66 | 4.99 | 5.76 | 3.29 | 1.81 | 1.64 | 10.34 | 10.39 | 11.05 | 9.97 | 9.79 | 0.48 |
| LOC338328 | 338328 | 1.58 | 1.32 | 1.20 | 0.00 | 0.58 | 0.64 | 7.74 | 7.39 | 7.09 | 7.85 | 7.65 | 0.31 |
| CDH5 | 1003 | 2.14 | 0.00 | 0.00 | 0.00 | 1.32 | 0.99 | 7.53 | 6.63 | 7.14 | 7.78 | 7.39 | 0.44 |
| THRSP | 7069 | 0.00 | 0.26 | 0.00 | 0.00 | 0.00 | 0.12 | 6.99 | 7.52 | 7.43 | 6.66 | 4.61 | 1.19 |
| PFKFB1 | 5207 | 0.00 | 0.00 | 0.00 | 0.00 | 1.93 | 0.86 | 7.45 | 7.07 | 7.20 | 7.01 | 6.09 | 0.52 |
| TM6SF1 | 53346 | 0.00 | 0.00 | 0.00 | 0.00 | 0.00 | 0.00 | 6.36 | 6.59 | 6.50 | 6.47 | 6.84 | 0.18 |
| ADH1A | 124 | 3.49 | 1.20 | 3.29 | 1.38 | 2.17 | 1.06 | 9.13 | 9.02 | 9.52 | 7.78 | 8.76 | 0.65 |
| ABCG1 | 9619 | 0.00 | 1.85 | 0.00 | 0.00 | 0.00 | 0.83 | 7.80 | 6.96 | 6.93 | 6.74 | 6.00 | 0.64 |
| LDHD | 197257 | 1.58 | 2.23 | 0.00 | 0.00 | 1.81 | 1.05 | 7.21 | 7.88 | 7.48 | 7.83 | 7.76 | 0.28 |
| GIMAP6 | 474344 | 0.00 | 0.00 | 0.68 | 0.00 | 0.00 | 0.30 | 6.96 | 6.06 | 6.75 | 6.86 | 6.54 | 0.36 |
| PLVAP | 83483 | 1.93 | 0.00 | 2.72 | 3.25 | 0.93 | 1.32 | 8.44 | 7.31 | 8.93 | 8.19 | 8.25 | 0.59 |
| GPT | 2875 | 1.72 | 2.32 | 0.00 | 1.00 | 0.00 | 1.03 | 6.88 | 7.78 | 7.26 | 7.51 | 7.65 | 0.36 |
| LMO1 | 4004 | 0.00 | 0.00 | 0.00 | 0.58 | 0.00 | 0.26 | 6.74 | 6.56 | 6.06 | 7.34 | 5.88 | 0.58 |
| GPD1 | 2819 | 3.41 | 3.23 | 2.81 | 3.22 | 2.96 | 0.24 | 10.31 | 9.61 | 9.07 | 9.85 | 8.51 | 0.70 |
| CCL15 | 6359 | 3.15 | 1.85 | 1.14 | 2.41 | 0.14 | 1.16 | 8.50 | 7.84 | 8.24 | 7.75 | 8.07 | 0.30 |
| C2orf40 | 84417 | 0.00 | 2.20 | 0.00 | 1.58 | 2.68 | 1.24 | 7.43 | 7.94 | 7.50 | 7.32 | 7.89 | 0.28 |
| SLC29A2 | 3177 | 3.57 | 3.31 | 4.47 | 0.14 | 2.14 | 1.67 | 9.48 | 9.25 | 9.13 | 8.57 | 8.81 | 0.36 |
| ART3 | 419 | 3.67 | 3.26 | 2.94 | 4.24 | 3.57 | 0.48 | 10.10 | 9.95 | 10.36 | 9.49 | 9.01 | 0.54 |
| LOC161247 | 161247 | 3.64 | 6.11 | 4.68 | 3.79 | 4.78 | 0.99 | 10.93 | 11.19 | 10.60 | 10.81 | 10.70 | 0.23 |
| ESRRG | 2104 | 0.00 | 2.49 | 0.00 | 0.00 | 0.00 | 1.11 | 6.23 | 6.99 | 5.88 | 7.03 | 7.30 | 0.60 |
| MYH11 | 4629 | 3.26 | 3.29 | 3.51 | 2.79 | 3.17 | 0.27 | 9.27 | 8.31 | 10.43 | 9.59 | 9.27 | 0.76 |
| GIMAP4 | 55303 | 3.07 | 0.00 | 2.72 | 2.32 | 2.54 | 1.22 | 8.12 | 8.07 | 8.76 | 8.38 | 8.09 | 0.29 |
| DZIP3 | 9666 | 0.00 | 0.00 | 0.00 | 0.00 | 0.00 | 0.00 | 6.47 | 6.06 | 6.09 | 5.98 | 6.11 | 0.19 |
| KCNJ11 | 3767 | 2.10 | 3.04 | 0.00 | 0.00 | 1.38 | 1.33 | 8.14 | 7.26 | 7.60 | 7.15 | 6.89 | 0.48 |
| ABRA | 137735 | 0.00 | 0.00 | 1.14 | 0.00 | 0.00 | 0.51 | 7.16 | 5.83 | 5.94 | 6.30 | 6.37 | 0.52 |
| FAM107A | 11170 | 1.43 | 0.38 | 1.81 | 0.00 | 0.00 | 0.84 | 7.25 | 5.95 | 7.17 | 6.53 | 7.14 | 0.56 |
| RNASE1 | 6035 | 3.19 | 3.02 | 4.36 | 3.26 | 4.70 | 0.77 | 9.58 | 9.63 | 10.38 | 9.51 | 9.55 | 0.37 |
| TMEM88 | 92162 | 0.00 | 2.00 | 0.00 | 0.00 | 0.00 | 0.89 | 6.68 | 5.82 | 6.55 | 6.20 | 6.75 | 0.39 |
| SCN4B | 6330 | 0.00 | 0.00 | 0.00 | 0.00 | 0.00 | 0.00 | 6.93 | 5.05 | 5.52 | 6.90 | 5.58 | 0.86 |
| ADH1C | 126 | 2.14 | 0.00 | 2.29 | 1.72 | 0.00 | 1.14 | 7.34 | 6.97 | 7.53 | 6.40 | 7.60 | 0.49 |
| UCP3 | 7352 | 2.17 | 2.87 | 2.93 | 3.20 | 2.46 | 0.41 | 9.87 | 7.35 | 8.64 | 9.89 | 7.54 | 1.22 |
| C20orf160 | 140706 | 0.00 | 0.00 | 0.00 | 0.00 | 0.00 | 0.00 | 6.06 | 5.16 | 5.80 | 6.24 | 6.25 | 0.45 |
| CTSG | 1511 | 1.72 | 0.00 | 0.00 | 0.00 | 0.00 | 0.77 | 5.74 | 6.23 | 6.37 | 6.81 | 6.05 | 0.40 |
| SYNE2 | 23224 | 0.00 | 0.00 | 0.00 | 0.00 | 0.00 | 0.00 | 6.11 | 5.71 | 6.06 | 5.71 | 5.76 | 0.20 |
| MS4A6A | 64231 | 0.00 | 1.07 | 0.00 | 0.00 | 0.00 | 0.48 | 6.15 | 5.99 | 7.04 | 5.17 | 6.03 | 0.67 |
| LRP1B | 53353 | 0.00 | 0.00 | 0.00 | 0.00 | 0.00 | 0.00 | 5.01 | 5.99 | 4.72 | 6.86 | 6.64 | 0.96 |
| MEOX1 | 4222 | 1.49 | 0.00 | 0.00 | 1.49 | 0.00 | 0.81 | 6.37 | 5.70 | 5.94 | 6.79 | 7.11 | 0.58 |
| EMCN | 51705 | 0.00 | 0.00 | 0.00 | 0.00 | 0.00 | 0.00 | 5.56 | 5.07 | 6.17 | 6.44 | 5.65 | 0.54 |
| CXCL14 | 9547 | 1.77 | 2.23 | 1.49 | 2.61 | 0.85 | 0.68 | 8.19 | 8.06 | 7.23 | 8.13 | 6.13 | 0.89 |
| COX4I2 | 84701 | 0.00 | 0.00 | 0.00 | 0.00 | 0.00 | 0.00 | 5.19 | 5.50 | 6.71 | 5.72 | 5.34 | 0.60 |
| NRAP | 4892 | 0.00 | 0.00 | 0.00 | 0.00 | 0.00 | 0.00 | 4.03 | 5.10 | 6.24 | 6.50 | 6.49 | 1.08 |
| AIF1 | 199 | 0.49 | 0.00 | 0.00 | 1.72 | 0.00 | 0.75 | 5.71 | 5.93 | 6.94 | 5.79 | 6.17 | 0.50 |
| CSF1R | 1436 | 3.07 | 1.96 | 3.46 | 0.85 | 0.77 | 1.24 | 7.79 | 7.65 | 8.18 | 7.24 | 7.49 | 0.35 |
| PLN | 5350 | 3.82 | 2.51 | 3.62 | 2.91 | 2.98 | 0.54 | 8.23 | 9.02 | 8.70 | 8.63 | 9.45 | 0.46 |
| ATP2B2 | 491 | 0.93 | 0.00 | 1.93 | 0.00 | 0.00 | 0.86 | 7.63 | 6.02 | 6.41 | 6.43 | 4.34 | 1.18 |
| UGT3A1 | 133688 | 0.14 | 0.00 | 0.00 | 0.26 | 0.00 | 0.12 | 5.90 | 6.16 | 5.27 | 5.53 | 5.47 | 0.36 |
| TYRP1 | 7306 | 0.00 | 0.00 | 0.00 | 0.00 | 0.85 | 0.38 | 4.61 | 4.84 | 4.72 | 6.75 | 7.76 | 1.43 |
| SLC38A3 | 10991 | 1.68 | 0.00 | 0.00 | 0.00 | 0.00 | 0.75 | 6.05 | 5.89 | 5.71 | 5.94 | 5.83 | 0.13 |
| CABC1 | 56997 | 6.04 | 6.77 | 6.61 | 4.54 | 6.02 | 0.88 | 11.55 | 11.33 | 11.22 | 11.77 | 11.37 | 0.22 |
| PEBP4 | 157310 | 3.10 | 2.20 | 3.57 | 3.62 | 3.38 | 0.58 | 8.94 | 8.56 | 8.61 | 9.02 | 7.75 | 0.50 |
| GIMAP1 | 170575 | 1.43 | 0.00 | 0.00 | 2.63 | 0.00 | 1.19 | 6.32 | 5.71 | 6.31 | 6.27 | 6.38 | 0.27 |
| TXLNB | 167838 | 3.74 | 6.26 | 4.44 | 4.05 | 4.87 | 0.98 | 10.10 | 9.82 | 10.05 | 10.04 | 9.80 | 0.14 |
| SLC25A34 | 284723 | 2.63 | 4.08 | 3.58 | 4.02 | 3.45 | 0.58 | 9.38 | 8.63 | 9.02 | 9.60 | 7.48 | 0.83 |
| TMEM38A | 79041 | 3.25 | 4.31 | 1.81 | 2.54 | 3.81 | 1.00 | 8.59 | 8.27 | 8.38 | 8.58 | 8.20 | 0.18 |
| DACH1 | 1602 | 0.00 | 0.00 | 0.00 | 0.00 | 0.00 | 0.00 | 4.19 | 5.43 | 5.26 | 5.93 | 5.42 | 0.64 |
| MLXIPL | 51085 | 0.00 | 1.07 | 1.14 | 0.14 | 2.72 | 1.09 | 6.05 | 6.80 | 6.51 | 5.89 | 5.97 | 0.39 |
| WDR62 | 284403 | 2.46 | 2.83 | 1.96 | 1.26 | 2.14 | 0.59 | 6.41 | 7.48 | 6.95 | 7.90 | 8.02 | 0.67 |
| KCNA5 | 3741 | 0.00 | 0.00 | 0.00 | 0.00 | 0.00 | 0.00 | 5.33 | 3.50 | 5.56 | 5.83 | 5.79 | 0.97 |
| RGS9BP | 388531 | 1.14 | 0.00 | 0.00 | 1.49 | 0.00 | 0.73 | 5.17 | 5.63 | 5.98 | 5.96 | 5.84 | 0.33 |
| HYAL1 | 3373 | 2.32 | 2.35 | 3.47 | 1.85 | 4.06 | 0.92 | 7.48 | 8.18 | 7.93 | 7.98 | 8.37 | 0.33 |
| GPC3 | 2719 | 0.00 | 0.93 | 1.26 | 1.26 | 0.00 | 0.65 | 6.15 | 5.76 | 6.62 | 4.68 | 6.03 | 0.73 |
| PDLIM5 | 10611 | 4.58 | 6.70 | 3.98 | 5.42 | 5.42 | 1.02 | 10.32 | 10.43 | 10.26 | 10.26 | 10.59 | 0.14 |
| JPH1 | 56704 | 3.94 | 6.23 | 4.19 | 4.29 | 5.28 | 0.96 | 10.36 | 10.01 | 10.03 | 9.85 | 9.43 | 0.34 |
| RXRG | 6258 | 0.00 | 1.32 | 0.00 | 0.00 | 0.00 | 0.59 | 6.51 | 5.29 | 5.03 | 4.58 | 5.63 | 0.72 |
| MYBPC1 | 4604 | 1.14 | 0.00 | 1.20 | 1.00 | 0.00 | 0.61 | 5.09 | 5.76 | 6.65 | 5.39 | 6.11 | 0.61 |
| WNK2 | 65268 | 0.38 | 0.00 | 1.26 | 2.20 | 0.00 | 0.95 | 6.22 | 5.50 | 5.97 | 6.19 | 5.48 | 0.36 |
| MYO5C | 55930 | 0.00 | 0.38 | 0.00 | 0.00 | 0.00 | 0.17 | 5.48 | 5.35 | 5.03 | 5.20 | 4.73 | 0.29 |
| PLCD4 | 84812 | 3.49 | 3.20 | 4.52 | 2.81 | 2.72 | 0.72 | 8.42 | 8.70 | 8.27 | 8.51 | 8.15 | 0.21 |
| FXYD1 | 5348 | 0.00 | 1.14 | 0.00 | 0.00 | 0.00 | 0.51 | 5.30 | 5.62 | 5.36 | 4.72 | 5.45 | 0.34 |
| NMNAT3 | 349565 | 0.00 | 0.00 | 0.00 | 0.00 | 0.00 | 0.00 | 5.06 | 5.75 | 4.58 | 5.44 | 4.41 | 0.56 |
| TSPAN18 | 90139 | 0.00 | 0.68 | 0.00 | 0.00 | 0.38 | 0.31 | 4.72 | 4.24 | 5.12 | 5.87 | 6.11 | 0.78 |
| MUM1L1 | 139221 | 0.00 | 3.36 | 0.00 | 1.38 | 1.14 | 1.38 | 5.80 | 6.52 | 5.92 | 6.28 | 6.36 | 0.30 |
| SOX7 | 83595 | 1.14 | 0.00 | 0.00 | 0.58 | 0.00 | 0.51 | 5.33 | 4.69 | 5.53 | 6.05 | 5.05 | 0.51 |
| MOSC1 | 64757 | 0.00 | 1.93 | 3.87 | 0.00 | 0.00 | 1.73 | 5.83 | 6.55 | 7.30 | 5.46 | 5.57 | 0.77 |
| ADSSL1 | 122622 | 0.00 | 0.00 | 0.00 | 0.00 | 0.00 | 0.00 | 5.28 | 3.82 | 6.06 | 5.19 | 4.54 | 0.84 |
| C15orf27 | 123591 | 0.00 | 0.00 | 0.00 | 0.00 | 0.14 | 0.06 | 6.04 | 4.81 | 5.00 | 4.86 | 4.17 | 0.67 |
| FLJ32310 | 123624 | 0.38 | 0.00 | 0.49 | 0.00 | 0.00 | 0.24 | 4.41 | 6.48 | 4.56 | 4.94 | 5.17 | 0.82 |
| MYLK2 | 85366 | 4.06 | 3.15 | 4.29 | 3.29 | 3.96 | 0.50 | 9.07 | 8.09 | 9.62 | 8.42 | 8.25 | 0.64 |
| IL17D | 53342 | 6.00 | 4.96 | 5.78 | 5.64 | 5.12 | 0.44 | 9.91 | 10.53 | 10.32 | 10.54 | 10.86 | 0.35 |
| GIMAP5 | 55340 | 1.93 | 0.00 | 1.38 | 1.43 | 0.14 | 0.86 | 5.58 | 5.71 | 6.28 | 6.24 | 5.69 | 0.33 |
| SAMD4A | 23034 | 4.52 | 4.88 | 4.52 | 6.62 | 4.96 | 0.87 | 9.95 | 10.02 | 9.84 | 10.10 | 10.20 | 0.14 |
| MS4A6A | 64231 | 0.00 | 0.00 | 0.00 | 0.00 | 0.00 | 0.00 | 4.90 | 4.90 | 6.10 | 3.89 | 4.84 | 0.79 |
| CUL3 | 8452 | 0.00 | 1.77 | 0.00 | 0.00 | 0.38 | 0.77 | 5.59 | 5.69 | 4.82 | 5.53 | 5.07 | 0.37 |
| LOH3CR2A | 29931 | 1.63 | 3.58 | 1.96 | 3.96 | 4.02 | 1.15 | 7.47 | 8.37 | 7.56 | 8.18 | 8.06 | 0.39 |
| RASGRP3 | 25780 | 2.00 | 3.41 | 2.61 | 3.45 | 1.96 | 0.72 | 7.46 | 7.84 | 7.44 | 7.80 | 7.33 | 0.23 |
| ADPRHL1 | 113622 | 4.61 | 4.29 | 3.28 | 4.96 | 5.10 | 0.73 | 9.56 | 9.46 | 8.96 | 9.26 | 9.41 | 0.23 |
| GJA4 | 2701 | 2.98 | 0.00 | 2.89 | 2.91 | 1.07 | 1.36 | 7.03 | 6.21 | 7.07 | 7.12 | 6.78 | 0.38 |
| TUBA8 | 51807 | 0.00 | 0.00 | 0.00 | 0.00 | 0.00 | 0.00 | 5.55 | 5.54 | 3.55 | 4.83 | 4.86 | 0.81 |
| FLG2 | 388698 | 0.00 | 0.00 | 0.00 | 0.68 | 0.00 | 0.30 | 5.20 | 5.01 | 4.94 | 5.00 | 4.82 | 0.14 |
| HPN | 3249 | 0.00 | 0.58 | 0.00 | 0.00 | 0.00 | 0.26 | 5.03 | 5.83 | 5.89 | 3.83 | 4.26 | 0.92 |
| MB | 4151 | 0.00 | 0.00 | 0.00 | 0.00 | 0.00 | 0.00 | 5.40 | 4.29 | 5.10 | 5.39 | 4.01 | 0.65 |
| RCSD1 | 92241 | 2.58 | 4.32 | 1.43 | 1.77 | 1.43 | 1.22 | 7.40 | 7.53 | 7.23 | 6.98 | 6.53 | 0.39 |
| FAM46C | 54855 | 2.10 | 2.32 | 2.49 | 3.57 | 2.83 | 0.57 | 7.11 | 8.27 | 7.41 | 7.51 | 7.11 | 0.48 |
| FOLR2 | 2350 | 0.00 | 0.00 | 0.00 | 0.00 | 0.00 | 0.00 | 4.77 | 4.99 | 6.00 | 3.85 | 4.45 | 0.79 |
| G0S2 | 50486 | 5.23 | 4.03 | 4.43 | 4.33 | 4.50 | 0.44 | 10.22 | 9.34 | 9.26 | 9.35 | 8.35 | 0.66 |
| SCN1B | 6324 | 3.55 | 2.93 | 3.83 | 1.00 | 2.93 | 1.10 | 7.51 | 7.64 | 8.37 | 7.59 | 7.02 | 0.48 |
| AIF1 | 199 | 0.00 | 0.00 | 0.00 | 0.93 | 1.20 | 0.59 | 4.74 | 4.73 | 6.43 | 4.86 | 5.23 | 0.72 |
| FGD5 | 152273 | 0.00 | 0.00 | 0.00 | 1.89 | 1.58 | 0.96 | 5.74 | 4.49 | 5.86 | 5.91 | 5.32 | 0.59 |
| CCDC69 | 26112 | 3.70 | 2.63 | 2.66 | 3.07 | 2.23 | 0.56 | 7.47 | 7.22 | 7.41 | 8.37 | 7.51 | 0.45 |
| PER1 | 5187 | 2.26 | 2.29 | 0.00 | 0.00 | 0.00 | 1.25 | 5.99 | 6.02 | 5.86 | 5.08 | 5.30 | 0.43 |
| KIAA1161 | 57462 | 1.96 | 0.00 | 0.00 | 0.00 | 0.58 | 0.85 | 5.62 | 5.43 | 5.16 | 4.99 | 4.93 | 0.29 |
| MB | 4151 | 0.00 | 0.00 | 0.00 | 0.00 | 0.00 | 0.00 | 4.04 | 5.50 | 3.39 | 5.11 | 5.31 | 0.91 |
| CLYBL | 171425 | 3.75 | 4.73 | 4.60 | 2.94 | 4.03 | 0.72 | 8.10 | 8.92 | 8.30 | 9.30 | 8.75 | 0.48 |
| ADHFE1 | 137872 | 4.90 | 4.74 | 4.96 | 3.62 | 4.88 | 0.57 | 9.22 | 9.70 | 9.04 | 9.04 | 9.38 | 0.27 |
| DKFZp451A211 | 400169 | 3.31 | 3.51 | 2.26 | 3.19 | 3.17 | 0.48 | 7.22 | 7.67 | 7.60 | 7.39 | 8.47 | 0.48 |
| HIGD1B | 51751 | 0.00 | 1.20 | 0.85 | 2.49 | 0.85 | 0.90 | 5.56 | 5.42 | 5.41 | 5.93 | 5.95 | 0.27 |
| ACACB | 32 | 5.32 | 5.30 | 5.72 | 3.74 | 4.80 | 0.76 | 9.99 | 9.09 | 9.80 | 9.76 | 9.04 | 0.44 |
| LEP | 3952 | 0.93 | 0.00 | 1.72 | 0.00 | 1.32 | 0.78 | 5.34 | 5.19 | 7.44 | 3.80 | 4.89 | 1.33 |
| BHLHB3 | 79365 | 4.03 | 2.74 | 2.51 | 2.20 | 2.66 | 0.70 | 7.15 | 7.37 | 7.35 | 7.77 | 7.15 | 0.25 |
| ZNF541 | 84215 | 0.00 | 2.10 | 0.00 | 2.66 | 1.68 | 1.23 | 3.91 | 6.91 | 5.50 | 5.89 | 6.82 | 1.22 |
| RRAGD | 58528 | 5.20 | 6.77 | 5.18 | 5.24 | 6.35 | 0.75 | 10.29 | 9.96 | 10.30 | 10.45 | 10.17 | 0.18 |
| CPEB3 | 22849 | 4.45 | 4.94 | 3.74 | 4.04 | 3.70 | 0.52 | 8.81 | 8.72 | 8.58 | 8.88 | 8.29 | 0.23 |
| PPP1R16B | 26051 | 0.00 | 0.00 | 0.00 | 1.26 | 0.00 | 0.56 | 5.23 | 3.87 | 5.00 | 5.06 | 4.50 | 0.55 |
| TM4SF18 | 116441 | 0.00 | 0.00 | 0.00 | 0.00 | 0.00 | 0.00 | 4.68 | 3.42 | 4.54 | 5.41 | 4.17 | 0.73 |
| ATP2A2 | 488 | 6.66 | 8.65 | 7.34 | 6.36 | 7.58 | 0.89 | 11.92 | 11.59 | 11.73 | 11.62 | 11.81 | 0.13 |
| RXRG | 6258 | 0.00 | 1.72 | 0.00 | 0.00 | 0.00 | 0.77 | 5.52 | 5.19 | 3.58 | 5.13 | 4.15 | 0.81 |
| FHL3 | 2275 | 6.59 | 6.86 | 6.61 | 5.98 | 7.25 | 0.46 | 11.69 | 10.82 | 11.20 | 10.80 | 10.58 | 0.44 |
| CCDC21 | 64793 | 3.14 | 3.61 | 2.66 | 2.32 | 3.46 | 0.54 | 7.62 | 7.57 | 7.03 | 7.39 | 7.36 | 0.23 |
| BDH1 | 622 | 0.00 | 1.00 | 1.00 | 0.00 | 0.00 | 0.55 | 3.31 | 5.27 | 5.30 | 4.70 | 5.10 | 0.83 |
| USP13 | 8975 | 5.80 | 6.18 | 5.24 | 5.88 | 5.55 | 0.35 | 9.63 | 10.08 | 10.04 | 10.13 | 10.43 | 0.29 |
| PPM1J | 333926 | 2.46 | 2.63 | 0.68 | 1.49 | 2.00 | 0.79 | 6.28 | 6.08 | 5.99 | 6.10 | 6.41 | 0.17 |
| EPM2A | 7957 | 3.38 | 3.81 | 2.94 | 3.04 | 3.82 | 0.41 | 7.62 | 7.40 | 7.62 | 7.94 | 7.98 | 0.24 |
| CITED4 | 163732 | 3.73 | 4.50 | 4.97 | 4.68 | 3.87 | 0.53 | 8.88 | 8.77 | 8.86 | 8.26 | 8.34 | 0.30 |
| APCDD1 | 147495 | 1.14 | 1.63 | 2.23 | 0.00 | 1.32 | 0.82 | 5.97 | 4.71 | 6.28 | 5.52 | 5.06 | 0.64 |
| TFEB | 7942 | 0.00 | 0.00 | 0.93 | 0.00 | 0.00 | 0.41 | 5.64 | 3.63 | 4.60 | 4.36 | 3.88 | 0.78 |
| DYRK1B | 9149 | 3.78 | 3.49 | 4.87 | 4.02 | 3.00 | 0.70 | 7.96 | 7.59 | 8.83 | 7.95 | 7.86 | 0.47 |
| CACNG6 | 59285 | 0.68 | 0.38 | 0.00 | 2.07 | 0.00 | 0.86 | 5.16 | 4.90 | 5.03 | 4.69 | 4.33 | 0.32 |
| C1orf21 | 81563 | 3.23 | 3.45 | 3.97 | 3.67 | 3.00 | 0.38 | 8.14 | 7.12 | 7.31 | 8.28 | 7.40 | 0.52 |
| PDZD2 | 23037 | 2.17 | 0.00 | 1.63 | 0.00 | 1.54 | 1.00 | 5.21 | 4.58 | 5.26 | 4.90 | 6.30 | 0.65 |
| C3orf45 | 132228 | 0.00 | 0.93 | 0.00 | 2.32 | 0.00 | 1.02 | 4.49 | 5.82 | 3.80 | 5.10 | 4.87 | 0.75 |
| TCEA3 | 6920 | 7.97 | 7.65 | 8.44 | 7.27 | 7.25 | 0.50 | 11.82 | 11.94 | 12.00 | 12.14 | 11.43 | 0.27 |
| ZNF91 | 7644 | 4.82 | 4.96 | 3.71 | 5.55 | 3.93 | 0.76 | 8.44 | 9.49 | 7.72 | 8.74 | 9.24 | 0.70 |
| ABHD1 | 84696 | 0.00 | 0.00 | 0.00 | 0.00 | 0.00 | 0.00 | 4.63 | 4.53 | 3.50 | 4.43 | 3.57 | 0.55 |
| ACSS1 | 84532 | 1.89 | 2.51 | 1.93 | 2.54 | 2.23 | 0.31 | 5.29 | 6.55 | 6.49 | 5.73 | 7.65 | 0.90 |
| IGSF6 | 10261 | 0.00 | 0.77 | 0.00 | 1.00 | 0.00 | 0.49 | 3.92 | 5.38 | 4.80 | 3.83 | 4.45 | 0.64 |
| GPR44 | 11251 | 1.85 | 0.00 | 1.43 | 1.43 | 1.72 | 0.74 | 5.74 | 4.81 | 6.22 | 4.94 | 5.33 | 0.58 |
| IMPA2 | 3613 | 6.69 | 5.81 | 6.74 | 5.69 | 5.41 | 0.61 | 10.52 | 9.74 | 10.18 | 10.55 | 9.71 | 0.41 |
| FCER1A | 2205 | 0.00 | 0.00 | 0.00 | 0.00 | 0.00 | 0.00 | 4.56 | 4.63 | 3.28 | 3.22 | 4.64 | 0.75 |
| MLCK | 91807 | 0.00 | 0.00 | 0.00 | 0.00 | 0.00 | 0.00 | 3.51 | 3.68 | 4.45 | 4.24 | 4.44 | 0.44 |
| EFNA1 | 1942 | 0.49 | 2.58 | 1.32 | 1.26 | 0.93 | 0.78 | 5.10 | 5.32 | 5.48 | 5.62 | 5.38 | 0.19 |
| LMO2 | 4005 | 4.38 | 5.40 | 3.31 | 4.36 | 3.39 | 0.86 | 8.11 | 8.23 | 8.34 | 8.34 | 8.08 | 0.12 |
| BCL2 | 596 | 4.08 | 3.93 | 2.85 | 2.56 | 3.84 | 0.69 | 7.85 | 7.37 | 7.33 | 7.56 | 7.35 | 0.22 |
| PACSIN3 | 29763 | 5.22 | 5.60 | 6.97 | 5.17 | 6.18 | 0.75 | 9.88 | 9.77 | 9.97 | 9.60 | 10.10 | 0.19 |
| GMFG | 9535 | 1.93 | 2.72 | 1.72 | 2.46 | 1.68 | 0.47 | 5.90 | 5.74 | 6.48 | 6.26 | 6.12 | 0.29 |
| ESR1 | 2099 | 3.45 | 2.07 | 2.93 | 0.14 | 2.61 | 1.28 | 6.40 | 6.67 | 6.49 | 5.26 | 6.28 | 0.55 |
| COQ10A | 93058 | 6.17 | 6.87 | 6.00 | 4.90 | 6.53 | 0.74 | 9.94 | 9.84 | 10.09 | 10.23 | 10.14 | 0.16 |
| PNPLA7 | 375775 | 4.62 | 4.45 | 5.00 | 5.00 | 5.26 | 0.32 | 8.95 | 8.99 | 8.61 | 9.08 | 8.44 | 0.27 |
| SCN2B | 6327 | 1.43 | 1.93 | 2.56 | 0.00 | 2.14 | 0.99 | 5.59 | 5.52 | 5.94 | 4.98 | 5.68 | 0.35 |
| PFKM | 5213 | 5.53 | 6.19 | 6.11 | 5.74 | 5.69 | 0.29 | 10.35 | 9.55 | 9.86 | 9.94 | 9.17 | 0.44 |
| MAPK12 | 6300 | 5.42 | 4.65 | 4.28 | 4.68 | 5.40 | 0.50 | 8.58 | 8.79 | 8.88 | 8.78 | 8.94 | 0.14 |
| ANK1 | 286 | 3.67 | 3.99 | 2.66 | 4.71 | 3.88 | 0.74 | 7.66 | 7.68 | 6.93 | 8.14 | 7.99 | 0.47 |
| GUCY1A3 | 2982 | 2.04 | 2.14 | 2.79 | 3.02 | 2.46 | 0.42 | 5.87 | 5.59 | 6.87 | 7.05 | 6.55 | 0.63 |
| FMO2 | 2327 | 1.38 | 2.41 | 2.54 | 2.00 | 1.20 | 0.60 | 5.63 | 5.70 | 5.90 | 5.88 | 5.50 | 0.17 |
| PKIA | 5569 | 5.93 | 6.68 | 5.94 | 5.69 | 5.87 | 0.38 | 9.89 | 9.83 | 9.31 | 9.94 | 10.21 | 0.33 |
| GALNTL2 | 117248 | 0.00 | 0.00 | 2.10 | 1.14 | 0.00 | 0.95 | 4.71 | 3.15 | 6.41 | 4.45 | 3.56 | 1.26 |
| GPD1L | 23171 | 7.73 | 7.69 | 7.92 | 5.97 | 7.23 | 0.79 | 10.94 | 11.43 | 11.08 | 10.86 | 11.20 | 0.23 |
| C1orf127 | 148345 | 0.00 | 0.38 | 0.38 | 0.00 | 0.00 | 0.21 | 4.07 | 4.79 | 3.72 | 4.12 | 3.00 | 0.65 |
| COLQ | 8292 | 2.07 | 4.71 | 4.08 | 2.17 | 3.70 | 1.18 | 6.27 | 7.42 | 7.44 | 6.64 | 7.91 | 0.67 |
| LHX6 | 26468 | 0.00 | 0.00 | 1.32 | 0.00 | 0.00 | 0.59 | 3.98 | 3.04 | 4.82 | 5.07 | 3.29 | 0.90 |
| GAMT | 2593 | 5.97 | 6.93 | 6.14 | 5.10 | 6.63 | 0.70 | 10.09 | 10.20 | 10.14 | 9.74 | 9.41 | 0.34 |
| SPOCK2 | 9806 | 2.14 | 2.43 | 2.81 | 2.23 | 2.26 | 0.26 | 5.25 | 5.97 | 6.17 | 6.84 | 6.40 | 0.59 |
| SLC6A1 | 6529 | 0.00 | 0.00 | 1.58 | 0.68 | 0.00 | 0.70 | 4.49 | 3.57 | 3.93 | 4.82 | 4.16 | 0.48 |
| FAM78A | 286336 | 1.07 | 0.00 | 0.00 | 0.00 | 0.00 | 0.48 | 4.45 | 4.42 | 4.01 | 3.67 | 3.14 | 0.55 |
| SNTA1 | 6640 | 5.35 | 5.06 | 6.58 | 4.91 | 4.76 | 0.73 | 8.95 | 8.74 | 9.22 | 9.02 | 9.22 | 0.20 |
| GAMT | 2593 | 7.77 | 8.26 | 8.13 | 6.66 | 8.25 | 0.68 | 11.59 | 11.84 | 11.26 | 11.53 | 11.34 | 0.23 |
| IQWD1 | 55827 | 2.23 | 3.98 | 2.77 | 2.41 | 3.19 | 0.70 | 6.07 | 6.32 | 7.19 | 6.69 | 6.76 | 0.43 |
| GHR | 2690 | 5.83 | 5.78 | 5.91 | 5.26 | 5.59 | 0.26 | 9.65 | 9.34 | 9.25 | 9.28 | 9.11 | 0.20 |
| FEM1A | 55527 | 7.58 | 8.34 | 7.58 | 8.54 | 7.79 | 0.45 | 11.79 | 11.39 | 11.38 | 11.84 | 11.65 | 0.22 |
| MACROD1 | 28992 | 5.71 | 6.26 | 5.96 | 4.60 | 5.98 | 0.65 | 9.51 | 9.42 | 9.43 | 9.40 | 8.95 | 0.22 |
| FKBP5 | 2289 | 4.50 | 3.88 | 5.67 | 3.88 | 4.08 | 0.75 | 9.08 | 7.59 | 8.80 | 6.77 | 7.88 | 0.93 |
| MAPT | 4137 | 1.14 | 0.00 | 0.49 | 0.00 | 0.00 | 0.50 | 5.27 | 3.15 | 3.89 | 4.14 | 3.25 | 0.85 |
| CD38 | 952 | 3.14 | 1.85 | 1.58 | 3.23 | 2.54 | 0.74 | 6.26 | 6.04 | 5.93 | 6.28 | 5.81 | 0.21 |
| ACADM | 34 | 5.90 | 6.15 | 5.63 | 5.50 | 5.97 | 0.26 | 9.20 | 9.23 | 9.37 | 9.63 | 9.67 | 0.22 |
| KIAA1737 | 85457 | 5.85 | 6.82 | 6.17 | 5.49 | 6.09 | 0.49 | 9.79 | 9.64 | 9.87 | 9.48 | 9.36 | 0.21 |
| AMICA1 | 120425 | 0.00 | 0.00 | 0.00 | 0.00 | 0.00 | 0.00 | 3.72 | 3.80 | 3.41 | 3.75 | 2.96 | 0.35 |
| MKNK2 | 2872 | 5.29 | 4.99 | 4.59 | 4.03 | 4.79 | 0.47 | 8.09 | 8.04 | 8.90 | 8.29 | 7.93 | 0.38 |
| ROBO4 | 54538 | 2.35 | 2.46 | 1.32 | 1.96 | 1.58 | 0.49 | 5.82 | 5.15 | 5.52 | 5.62 | 5.09 | 0.31 |
| TACC2 | 10579 | 6.16 | 7.07 | 7.09 | 7.07 | 6.86 | 0.40 | 10.50 | 10.23 | 10.36 | 10.37 | 10.16 | 0.13 |
| DCUN1D2 | 55208 | 2.41 | 3.35 | 2.68 | 2.89 | 2.87 | 0.35 | 6.32 | 6.13 | 6.39 | 6.61 | 6.11 | 0.20 |
| AR | 367 | 3.67 | 3.98 | 4.64 | 2.85 | 3.34 | 0.68 | 6.96 | 6.87 | 7.63 | 7.37 | 7.01 | 0.32 |
| L2HGDH | 79944 | 3.34 | 4.23 | 3.61 | 3.56 | 4.23 | 0.41 | 7.19 | 7.29 | 7.01 | 7.64 | 7.17 | 0.24 |
| ATP1B1 | 481 | 6.16 | 6.27 | 5.33 | 6.86 | 6.78 | 0.61 | 10.11 | 9.86 | 9.59 | 9.39 | 9.77 | 0.27 |
| RHOU | 58480 | 4.19 | 5.41 | 4.12 | 5.15 | 4.79 | 0.57 | 8.78 | 8.51 | 8.03 | 7.85 | 7.79 | 0.43 |
| C22orf32 | 91689 | 3.04 | 4.02 | 3.92 | 3.56 | 3.46 | 0.39 | 6.90 | 7.44 | 6.84 | 7.05 | 6.96 | 0.24 |
| NXN | 64359 | 6.00 | 4.72 | 4.14 | 3.98 | 4.10 | 0.84 | 8.41 | 7.93 | 7.96 | 7.81 | 7.96 | 0.23 |
| CBX7 | 23492 | 5.03 | 4.60 | 5.76 | 3.49 | 5.07 | 0.84 | 8.05 | 8.16 | 8.59 | 8.11 | 8.14 | 0.22 |
| LOC205251 | | 7.54 | 7.76 | 6.76 | 6.72 | 7.44 | 0.47 | 10.88 | 10.62 | 10.30 | 10.72 | 10.75 | 0.22 |
| C10orf92 | 54777 | 0.85 | 2.56 | 3.07 | 3.14 | 2.10 | 0.94 | 4.94 | 5.61 | 5.83 | 6.46 | 5.89 | 0.55 |
| EPB49 | 2039 | 2.00 | 2.46 | 1.93 | 2.26 | 1.49 | 0.37 | 5.18 | 5.55 | 4.88 | 5.73 | 5.78 | 0.39 |
| SHMT1 | 6470 | 3.87 | 3.85 | 4.45 | 3.54 | 3.43 | 0.40 | 7.62 | 7.64 | 7.22 | 7.13 | 6.41 | 0.50 |
| PPP1R13B | 23368 | 2.87 | 3.38 | 3.22 | 2.79 | 4.13 | 0.54 | 6.67 | 6.54 | 6.32 | 7.03 | 6.68 | 0.26 |
| ATP5G1 | 516 | 4.72 | 3.71 | 3.36 | 4.58 | 4.59 | 0.61 | 7.82 | 7.14 | 7.34 | 7.96 | 7.56 | 0.33 |
| ACO2 | 50 | 6.14 | 6.12 | 5.53 | 5.05 | 6.06 | 0.48 | 9.21 | 9.00 | 9.19 | 9.23 | 9.01 | 0.11 |
| APOB | 338 | 0.00 | 0.00 | 1.07 | 0.00 | 0.00 | 0.48 | 3.58 | 2.70 | 4.07 | 3.41 | 3.95 | 0.54 |
| CUTC | 51076 | 7.31 | 7.44 | 6.84 | 7.07 | 6.94 | 0.25 | 10.23 | 10.59 | 10.23 | 10.46 | 10.67 | 0.20 |
| ARMETL1 | 441549 | 4.08 | 3.64 | 4.02 | 2.94 | 3.86 | 0.46 | 7.02 | 7.27 | 6.96 | 6.95 | 6.87 | 0.15 |
| GBAS | 2631 | 7.34 | 7.40 | 7.60 | 6.65 | 7.46 | 0.37 | 10.66 | 10.47 | 10.56 | 10.65 | 10.61 | 0.08 |
| OGDH | 4967 | 4.91 | 5.63 | 5.30 | 5.31 | 4.95 | 0.30 | 8.56 | 8.28 | 8.93 | 8.43 | 8.30 | 0.26 |
| XPO4 | 64328 | 6.17 | 6.86 | 6.17 | 6.46 | 6.28 | 0.29 | 8.73 | 10.37 | 9.53 | 9.65 | 10.03 | 0.61 |
| SSH2 | 85464 | 5.15 | 5.50 | 5.50 | 5.36 | 5.41 | 0.14 | 8.48 | 8.74 | 8.75 | 8.71 | 8.57 | 0.12 |
| LOC128977 | 128977 | 3.73 | 4.31 | 4.33 | 3.68 | 4.22 | 0.32 | 7.37 | 7.62 | 7.13 | 7.00 | 7.48 | 0.25 |
| LCP2 | 3937 | 0.00 | 0.00 | 1.26 | 2.29 | 1.32 | 0.98 | 3.61 | 4.04 | 4.72 | 5.00 | 3.80 | 0.60 |
| SESN1 | 27244 | 6.24 | 6.51 | 5.92 | 4.11 | 5.47 | 0.94 | 9.16 | 9.13 | 8.99 | 8.51 | 8.64 | 0.29 |
| TBC1D4 | 9882 | 4.87 | 4.77 | 5.59 | 5.10 | 4.72 | 0.36 | 8.67 | 8.04 | 8.33 | 8.23 | 7.92 | 0.29 |
| GPT2 | 84706 | 7.32 | 7.44 | 6.51 | 6.32 | 7.54 | 0.56 | 10.27 | 10.25 | 10.11 | 10.14 | 10.48 | 0.15 |
| FGL2 | 10875 | 4.83 | 3.41 | 3.90 | 3.91 | 3.54 | 0.56 | 7.60 | 7.14 | 6.20 | 7.33 | 7.40 | 0.55 |
| STBD1 | 8987 | 3.52 | 4.09 | 3.92 | 4.36 | 3.56 | 0.36 | 7.77 | 6.89 | 7.22 | 6.93 | 6.46 | 0.48 |
| ABCA5 | 23461 | 3.07 | 3.43 | 2.26 | 4.03 | 2.32 | 0.75 | 6.39 | 6.81 | 5.23 | 6.26 | 6.22 | 0.58 |
| PPAPDC3 | 84814 | 7.07 | 6.95 | 7.08 | 6.83 | 7.18 | 0.13 | 10.54 | 10.10 | 9.91 | 10.21 | 9.97 | 0.25 |
| FHL1 | 2273 | 9.43 | 9.82 | 9.48 | 8.05 | 9.61 | 0.70 | 12.22 | 12.42 | 12.42 | 12.36 | 12.58 | 0.13 |
| PPP2R3A | 5523 | 5.13 | 5.45 | 5.69 | 6.54 | 5.39 | 0.54 | 8.40 | 8.89 | 8.97 | 8.83 | 8.67 | 0.23 |
| CLIP1 | 6249 | 2.68 | 2.87 | 2.54 | 2.91 | 2.70 | 0.15 | 4.97 | 6.14 | 6.15 | 5.88 | 5.97 | 0.49 |
| DKFZP564O0823 | 25849 | 4.64 | 5.54 | 4.81 | 4.75 | 3.94 | 0.57 | 7.59 | 8.00 | 8.11 | 7.47 | 7.92 | 0.27 |
| NUDT8 | 254552 | 6.11 | 5.44 | 5.98 | 4.58 | 5.40 | 0.60 | 8.47 | 8.65 | 8.61 | 8.54 | 8.62 | 0.08 |
| FBXO32 | 114907 | 7.85 | 7.78 | 8.01 | 7.15 | 7.75 | 0.33 | 11.25 | 10.78 | 10.91 | 10.81 | 10.13 | 0.41 |
| ASPH | 444 | 4.16 | 4.13 | 3.39 | 5.42 | 3.87 | 0.75 | 7.25 | 7.15 | 6.75 | 7.79 | 7.29 | 0.37 |
| HCN1 | 348980 | 0.00 | 0.00 | 0.00 | 0.00 | 0.00 | 0.00 | 3.36 | 2.94 | 3.66 | 3.23 | 2.04 | 0.62 |
| KIAA0828 | 23382 | 4.18 | 4.13 | 4.10 | 2.91 | 3.45 | 0.56 | 7.29 | 7.05 | 6.68 | 6.38 | 6.54 | 0.37 |
| MLLT6 | 4302 | 5.55 | 5.56 | 6.41 | 5.08 | 5.19 | 0.52 | 9.07 | 8.72 | 8.49 | 8.57 | 8.12 | 0.35 |
| CRY2 | 1408 | 6.68 | 6.15 | 6.67 | 7.42 | 6.32 | 0.49 | 9.63 | 9.95 | 9.51 | 9.74 | 9.53 | 0.18 |
| PECAM1 | 5175 | 3.91 | 3.17 | 3.98 | 4.19 | 3.09 | 0.50 | 6.54 | 6.30 | 7.18 | 6.69 | 6.67 | 0.32 |
| GOT2 | 2806 | 7.59 | 7.37 | 7.71 | 7.49 | 7.65 | 0.13 | 10.78 | 10.34 | 10.48 | 10.72 | 10.51 | 0.18 |
| PLA2G4C | 8605 | 6.50 | 6.34 | 6.99 | 6.11 | 6.04 | 0.38 | 9.34 | 9.76 | 8.95 | 9.64 | 9.25 | 0.32 |
| CD163 | 9332 | 0.26 | 0.00 | 1.81 | 0.00 | 0.00 | 0.79 | 4.07 | 3.43 | 4.08 | 2.66 | 2.72 | 0.69 |
| SPINK2 | 6691 | 0.00 | 0.00 | 0.00 | 0.00 | 0.14 | 0.06 | 2.20 | 3.20 | 3.22 | 3.57 | 2.79 | 0.52 |
| MYLIP | 29116 | 5.87 | 6.51 | 6.56 | 5.97 | 4.99 | 0.64 | 8.97 | 8.99 | 9.28 | 8.95 | 8.45 | 0.30 |
| KIF1C | 10749 | 5.29 | 5.15 | 5.55 | 5.04 | 5.40 | 0.20 | 8.41 | 7.71 | 8.29 | 8.26 | 8.40 | 0.29 |
| GSTM4 | 2948 | 4.48 | 3.85 | 4.55 | 3.39 | 4.22 | 0.48 | 6.73 | 7.07 | 7.46 | 6.74 | 7.02 | 0.30 |
| DNAJB5 | 25822 | 3.88 | 4.94 | 4.72 | 4.58 | 4.43 | 0.40 | 7.78 | 7.51 | 7.55 | 6.99 | 7.24 | 0.30 |
| NDUFS7 | 374291 | 8.09 | 7.52 | 7.87 | 7.20 | 8.16 | 0.41 | 10.64 | 10.71 | 10.49 | 10.79 | 10.64 | 0.11 |
| CX3CL1 | 6376 | 2.29 | 0.00 | 3.31 | 3.15 | 2.41 | 1.32 | 5.42 | 3.35 | 5.18 | 6.10 | 5.49 | 1.04 |
| EIF4E3 | 317649 | 3.36 | 2.93 | 2.81 | 3.55 | 3.12 | 0.31 | 6.07 | 5.78 | 6.06 | 6.18 | 5.98 | 0.15 |
| ECH1 | 1891 | 8.84 | 8.86 | 8.71 | 7.86 | 8.82 | 0.43 | 11.74 | 11.27 | 11.33 | 11.71 | 11.33 | 0.23 |
| HADH | 3033 | 7.78 | 8.45 | 8.24 | 7.32 | 7.77 | 0.44 | 10.46 | 10.98 | 10.57 | 10.78 | 10.99 | 0.24 |
| C20orf26 | 26074 | 2.54 | 3.46 | 3.23 | 2.93 | 3.42 | 0.39 | 4.93 | 6.38 | 6.71 | 5.22 | 6.54 | 0.82 |
| NANOS1 | 340719 | 2.17 | 1.49 | 3.26 | 3.00 | 1.81 | 0.76 | 5.76 | 4.45 | 5.58 | 5.63 | 4.49 | 0.65 |
| TBC1D8 | 11138 | 3.45 | 3.86 | 3.51 | 1.89 | 3.54 | 0.78 | 6.42 | 6.17 | 6.15 | 5.45 | 6.20 | 0.37 |
| ANKRD46 | 157567 | 5.66 | 6.00 | 5.52 | 6.53 | 5.50 | 0.43 | 8.90 | 8.88 | 8.58 | 8.44 | 8.52 | 0.21 |
| DPEP2 | 64174 | 0.38 | 1.63 | 0.00 | 0.00 | 0.00 | 0.71 | 3.41 | 3.64 | 3.12 | 2.72 | 3.07 | 0.35 |
| ST6GALNAC2 | 10610 | 2.63 | 2.61 | 3.66 | 3.50 | 4.67 | 0.85 | 5.73 | 5.55 | 6.20 | 6.46 | 7.07 | 0.61 |
| GFOD1 | 54438 | 5.80 | 5.03 | 4.54 | 5.81 | 5.71 | 0.57 | 8.32 | 7.77 | 8.19 | 8.15 | 8.40 | 0.24 |
| LMOD3 | 56203 | 5.66 | 6.44 | 6.09 | 6.04 | 5.99 | 0.28 | 9.20 | 8.57 | 8.94 | 8.94 | 8.38 | 0.33 |
| FKBP3 | 2287 | 4.55 | 5.83 | 4.65 | 4.43 | 4.96 | 0.56 | 7.58 | 7.78 | 7.26 | 7.62 | 8.02 | 0.28 |
| PCNT | 5116 | 6.51 | 6.24 | 7.11 | 6.17 | 6.43 | 0.37 | 9.46 | 9.23 | 9.32 | 9.15 | 9.02 | 0.17 |
| BTG2 | 7832 | 4.82 | 5.05 | 5.14 | 4.04 | 4.78 | 0.43 | 7.88 | 6.78 | 8.30 | 6.63 | 7.78 | 0.73 |
| RNF123 | 63891 | 5.80 | 6.11 | 5.51 | 5.46 | 5.84 | 0.26 | 8.71 | 8.53 | 8.39 | 8.64 | 7.99 | 0.28 |
| PPP1R3F | 89801 | 4.96 | 6.23 | 4.57 | 4.97 | 5.52 | 0.64 | 7.92 | 8.10 | 7.72 | 7.96 | 8.07 | 0.15 |
| ADRB2 | 154 | 3.79 | 5.73 | 3.87 | 4.78 | 4.23 | 0.80 | 6.90 | 7.65 | 7.25 | 7.03 | 7.04 | 0.29 |
| PPP1R16A | 84988 | 6.22 | 6.05 | 6.24 | 6.03 | 6.09 | 0.10 | 8.14 | 9.31 | 8.77 | 8.61 | 9.22 | 0.48 |
| C11orf67 | 28971 | 7.71 | 8.08 | 7.96 | 7.11 | 8.16 | 0.42 | 10.62 | 10.58 | 10.21 | 10.73 | 10.26 | 0.23 |
| PHYH | 5264 | 8.81 | 9.29 | 9.14 | 8.09 | 8.70 | 0.47 | 11.52 | 11.55 | 11.36 | 11.63 | 11.33 | 0.13 |
| WWP1 | 11059 | 7.72 | 7.38 | 7.21 | 8.27 | 7.28 | 0.44 | 9.84 | 10.22 | 10.10 | 10.57 | 10.45 | 0.29 |
| HSDL2 | 84263 | 6.82 | 7.29 | 7.10 | 6.58 | 6.40 | 0.37 | 9.49 | 9.61 | 9.31 | 9.47 | 9.61 | 0.12 |
| FLJ90650 | 206338 | 2.70 | 2.04 | 2.51 | 2.61 | 2.29 | 0.27 | 5.45 | 5.46 | 4.63 | 5.32 | 4.57 | 0.45 |
| COQ3 | 51805 | 5.97 | 6.02 | 6.04 | 5.75 | 5.96 | 0.11 | 8.34 | 8.81 | 8.32 | 8.79 | 8.75 | 0.25 |
| TRIP10 | 9322 | 6.43 | 5.53 | 6.49 | 6.00 | 6.16 | 0.39 | 8.92 | 8.57 | 9.14 | 8.64 | 8.54 | 0.26 |
| ECHDC2 | 55268 | 7.13 | 6.97 | 7.60 | 6.96 | 7.84 | 0.40 | 9.71 | 10.24 | 9.82 | 10.13 | 9.79 | 0.23 |
| AKAP7 | 9465 | 5.18 | 5.06 | 4.25 | 4.59 | 5.03 | 0.39 | 7.59 | 7.21 | 7.11 | 7.84 | 7.50 | 0.29 |
| USP47 | 55031 | 5.05 | 5.85 | 5.60 | 5.90 | 5.48 | 0.34 | 8.52 | 8.26 | 7.88 | 8.17 | 8.17 | 0.23 |
| ESRRA | 2101 | 4.86 | 4.68 | 4.50 | 5.26 | 5.24 | 0.34 | 7.41 | 7.28 | 7.75 | 7.57 | 7.61 | 0.18 |
| HSD17B7P2 | | 5.49 | 6.28 | 6.43 | 6.35 | 5.82 | 0.41 | 8.70 | 8.31 | 8.96 | 9.15 | 8.27 | 0.39 |
| LIAS | 11019 | 3.99 | 5.23 | 5.10 | 5.31 | 4.37 | 0.59 | 7.21 | 7.70 | 7.49 | 7.49 | 7.11 | 0.24 |
| TAPT1 | 202018 | 4.16 | 5.04 | 4.49 | 3.80 | 4.59 | 0.47 | 7.04 | 7.00 | 6.68 | 7.18 | 7.18 | 0.20 |
| ASB2 | 51676 | 4.22 | 3.15 | 3.83 | 3.91 | 3.71 | 0.39 | 7.27 | 6.15 | 6.25 | 6.38 | 5.66 | 0.58 |
| IPO13 | 9670 | 6.61 | 6.86 | 6.52 | 6.48 | 6.50 | 0.16 | 9.41 | 9.16 | 8.91 | 9.04 | 9.31 | 0.20 |
| BCKDHA | 593 | 7.21 | 6.99 | 7.60 | 6.43 | 7.09 | 0.42 | 9.37 | 9.96 | 9.65 | 9.55 | 9.65 | 0.22 |
| UBE2G1 | 7326 | 5.42 | 5.62 | 5.11 | 6.28 | 5.20 | 0.47 | 8.30 | 8.14 | 7.84 | 8.22 | 7.98 | 0.19 |
| COQ7 | 10229 | 2.81 | 3.25 | 2.79 | 2.26 | 3.25 | 0.41 | 5.59 | 5.58 | 5.11 | 5.64 | 5.22 | 0.24 |
| SLC25A11 | 8402 | 6.29 | 6.12 | 6.23 | 6.04 | 6.53 | 0.19 | 8.91 | 8.61 | 8.71 | 8.96 | 8.80 | 0.14 |
| NEURL | 9148 | 3.56 | 3.02 | 2.70 | 3.86 | 2.94 | 0.48 | 6.48 | 5.69 | 5.32 | 5.91 | 5.44 | 0.46 |
| VIT | 5212 | 2.32 | 0.00 | 1.43 | 0.00 | 0.00 | 1.08 | 4.31 | 2.61 | 4.28 | 1.96 | 3.32 | 1.03 |
| DLAT | 1737 | 5.41 | 6.11 | 5.13 | 5.88 | 5.57 | 0.39 | 8.46 | 8.19 | 7.67 | 8.12 | 8.28 | 0.29 |
| ACSS2 | 55902 | 8.25 | 7.92 | 8.20 | 7.41 | 7.97 | 0.33 | 10.57 | 10.50 | 10.52 | 10.32 | 10.44 | 0.10 |
| DUSP28 | 285193 | 5.75 | 6.85 | 5.77 | 5.52 | 6.33 | 0.54 | 8.54 | 9.00 | 8.00 | 8.56 | 8.61 | 0.35 |
| SIX1 | 6495 | 4.27 | 4.59 | 3.70 | 4.41 | 4.40 | 0.34 | 6.54 | 7.11 | 7.09 | 6.71 | 6.33 | 0.34 |
| SLCO2B1 | 11309 | 2.46 | 0.00 | 2.10 | 0.00 | 1.54 | 1.16 | 4.72 | 2.29 | 4.96 | 2.51 | 4.02 | 1.24 |
| LIAS | 11019 | 3.45 | 4.10 | 3.83 | 4.51 | 2.32 | 0.83 | 6.13 | 6.31 | 6.43 | 6.31 | 5.40 | 0.42 |
| ACAT1 | 38 | 8.80 | 8.76 | 8.82 | 8.28 | 8.61 | 0.23 | 10.80 | 11.29 | 10.96 | 11.23 | 11.31 | 0.23 |
| SDHA | 6389 | 5.99 | 5.68 | 5.90 | 5.59 | 5.68 | 0.17 | 8.07 | 7.96 | 8.71 | 8.46 | 7.96 | 0.34 |
| HBD | 3045 | 1.26 | 2.35 | 2.29 | 3.23 | 2.10 | 0.70 | 4.28 | 4.17 | 4.39 | 6.08 | 4.62 | 0.78 |
| C16orf14 | 84331 | 4.39 | 4.38 | 4.34 | 4.23 | 4.65 | 0.15 | 6.61 | 6.85 | 7.03 | 6.78 | 6.99 | 0.17 |
| PPP1CB | 5500 | 6.94 | 7.40 | 7.48 | 7.05 | 7.04 | 0.24 | 9.61 | 9.63 | 9.46 | 9.77 | 9.69 | 0.11 |
| LCP1 | 3936 | 3.77 | 2.51 | 4.18 | 3.50 | 3.79 | 0.63 | 6.05 | 5.50 | 6.72 | 6.15 | 5.51 | 0.51 |
| MFN2 | 9927 | 6.11 | 6.45 | 6.05 | 5.94 | 5.94 | 0.21 | 8.81 | 8.23 | 8.31 | 8.75 | 8.51 | 0.26 |
| C1orf162 | 128346 | 3.07 | 3.47 | 3.54 | 4.17 | 3.66 | 0.40 | 6.11 | 6.35 | 5.62 | 6.28 | 5.65 | 0.34 |
| TOB1 | 10140 | 6.73 | 5.76 | 6.21 | 6.47 | 6.24 | 0.36 | 8.55 | 8.34 | 9.15 | 8.86 | 8.59 | 0.32 |
| USP15 | 9958 | 4.36 | 5.26 | 4.57 | 5.06 | 4.74 | 0.36 | 7.48 | 7.45 | 6.66 | 7.30 | 7.17 | 0.33 |
| MT1X | 4501 | 8.65 | 8.59 | 9.27 | 8.39 | 8.92 | 0.34 | 11.54 | 10.96 | 11.52 | 10.82 | 11.05 | 0.33 |
| COX10 | 1352 | 4.74 | 4.32 | 4.15 | 5.26 | 4.41 | 0.44 | 7.09 | 6.87 | 6.90 | 7.15 | 6.90 | 0.13 |
| RPUSD4 | 84881 | 7.86 | 8.44 | 8.41 | 8.41 | 7.45 | 0.44 | 10.54 | 10.53 | 10.36 | 10.70 | 10.44 | 0.13 |
| PIB5PA | 27124 | 0.68 | 2.20 | 1.54 | 1.00 | 1.77 | 0.61 | 3.05 | 4.56 | 4.15 | 3.29 | 4.10 | 0.63 |
| ACSL1 | 2180 | 6.92 | 7.56 | 6.78 | 6.67 | 6.79 | 0.35 | 9.75 | 9.01 | 9.29 | 9.32 | 9.19 | 0.27 |
| MARCH6 | 10299 | 6.29 | 5.74 | 6.32 | 6.75 | 5.72 | 0.43 | 8.70 | 8.11 | 8.40 | 8.89 | 8.46 | 0.30 |
| SLC6A8 | 6535 | 3.77 | 4.22 | 4.66 | 4.25 | 4.45 | 0.33 | 6.93 | 6.46 | 6.77 | 6.67 | 6.23 | 0.27 |
| DCI | 1632 | 7.68 | 7.60 | 7.19 | 6.89 | 8.07 | 0.46 | 10.05 | 9.95 | 9.63 | 9.88 | 9.61 | 0.19 |
| COQ9 | 57017 | 7.48 | 7.68 | 7.62 | 7.20 | 7.58 | 0.19 | 9.83 | 9.88 | 9.92 | 9.93 | 9.68 | 0.10 |
| SUCLA2 | 8803 | 5.79 | 6.79 | 5.68 | 5.86 | 5.92 | 0.44 | 8.39 | 8.24 | 8.03 | 8.66 | 8.37 | 0.23 |
| DUSP3 | 1845 | 8.51 | 8.96 | 8.27 | 9.20 | 8.14 | 0.45 | 11.00 | 10.76 | 10.95 | 11.15 | 10.73 | 0.17 |
| KLHL23 | 151230 | 1.14 | 2.58 | 1.63 | 2.41 | 1.14 | 0.69 | 3.71 | 4.45 | 4.04 | 4.77 | 3.39 | 0.55 |
| ZYG11B | 79699 | 8.58 | 8.33 | 8.45 | 7.96 | 8.13 | 0.25 | 10.75 | 10.49 | 10.41 | 10.85 | 10.39 | 0.21 |
| CYCS | 54205 | 4.94 | 5.77 | 5.60 | 5.44 | 5.03 | 0.36 | 7.65 | 7.55 | 7.55 | 7.91 | 7.57 | 0.15 |
| UQCRC1 | 7384 | 8.32 | 8.56 | 8.45 | 7.77 | 8.64 | 0.35 | 10.73 | 10.51 | 10.45 | 10.83 | 10.63 | 0.16 |
| RXRA | 6256 | 7.20 | 6.53 | 7.75 | 7.16 | 6.99 | 0.44 | 9.55 | 9.18 | 9.79 | 9.37 | 9.15 | 0.27 |
| ARHGAP15 | 55843 | 2.43 | 2.23 | 2.54 | 2.04 | 3.47 | 0.55 | 4.67 | 4.26 | 5.06 | 4.73 | 5.37 | 0.42 |
| FHL5 | 9457 | 3.92 | 3.09 | 3.94 | 2.89 | 3.58 | 0.48 | 5.43 | 5.88 | 6.10 | 5.57 | 5.71 | 0.26 |
| SLC16A14 | 151473 | 1.32 | 0.00 | 1.26 | 0.68 | 1.00 | 0.54 | 3.80 | 2.32 | 3.09 | 3.10 | 3.23 | 0.53 |
| H3F3B | 3021 | 6.66 | 6.22 | 7.48 | 7.01 | 5.78 | 0.66 | 8.85 | 8.96 | 9.16 | 8.99 | 8.45 | 0.26 |
| KIF1B | 23095 | 2.66 | 3.07 | 2.93 | 3.49 | 3.28 | 0.32 | 4.77 | 4.61 | 5.54 | 5.91 | 5.83 | 0.60 |
| UBAC1 | 10422 | 8.37 | 8.46 | 8.46 | 7.64 | 8.36 | 0.35 | 10.46 | 10.63 | 10.50 | 10.56 | 10.38 | 0.10 |
| MITF | 4286 | 4.19 | 4.19 | 5.13 | 4.22 | 4.10 | 0.43 | 6.67 | 6.47 | 6.64 | 6.74 | 6.49 | 0.12 |
| OSBPL1A | 114876 | 6.96 | 6.41 | 6.78 | 6.17 | 6.70 | 0.31 | 9.00 | 8.76 | 8.73 | 9.01 | 8.61 | 0.18 |
| HERC1 | 8925 | 5.95 | 5.64 | 6.35 | 5.69 | 5.72 | 0.29 | 7.92 | 7.95 | 8.01 | 8.11 | 8.44 | 0.21 |
| FAM125B | 89853 | 5.68 | 4.85 | 5.03 | 5.03 | 5.34 | 0.33 | 7.77 | 7.56 | 7.24 | 7.20 | 7.13 | 0.27 |
| NDUFS2 | 4720 | 4.63 | 5.26 | 4.07 | 4.42 | 4.41 | 0.44 | 7.22 | 6.69 | 6.29 | 6.96 | 6.56 | 0.36 |
| ALDH2 | 217 | 7.57 | 7.05 | 7.83 | 6.33 | 6.98 | 0.58 | 9.40 | 9.34 | 9.79 | 8.94 | 9.19 | 0.31 |
| CSDE1 | 7812 | 5.07 | 5.35 | 4.63 | 4.69 | 5.04 | 0.30 | 7.12 | 6.97 | 6.88 | 7.50 | 7.20 | 0.24 |
| FLJ39378 | 353116 | 4.84 | 5.16 | 4.83 | 5.65 | 4.83 | 0.36 | 7.25 | 7.08 | 6.99 | 7.29 | 7.57 | 0.22 |
| ZNF76 | 7629 | 4.41 | 3.70 | 4.09 | 3.64 | 4.31 | 0.35 | 6.51 | 6.02 | 6.39 | 6.33 | 5.75 | 0.31 |
| DDX46 | 9879 | 5.33 | 5.69 | 5.46 | 5.45 | 5.26 | 0.16 | 7.57 | 7.69 | 7.40 | 7.84 | 7.54 | 0.17 |
| GSTM2 | 2946 | 4.72 | 5.13 | 5.57 | 5.31 | 5.31 | 0.32 | 7.21 | 7.48 | 8.00 | 6.92 | 7.26 | 0.40 |
| ATP5D | 513 | 9.31 | 9.04 | 9.49 | 8.87 | 9.40 | 0.26 | 11.35 | 11.38 | 11.42 | 11.49 | 11.26 | 0.09 |
| PARVB | 29780 | 6.34 | 6.22 | 6.36 | 5.93 | 6.48 | 0.21 | 8.13 | 8.29 | 8.99 | 8.26 | 8.47 | 0.34 |
| AFG3L2 | 10939 | 5.64 | 5.46 | 5.25 | 6.52 | 5.64 | 0.48 | 7.92 | 7.73 | 7.72 | 8.08 | 7.78 | 0.15 |
| TTLL2 | 83887 | 0.49 | 0.00 | 0.14 | 1.00 | 0.00 | 0.43 | 2.32 | 2.72 | 2.54 | 3.07 | 1.63 | 0.54 |
| C6orf106 | 64771 | 5.79 | 5.30 | 5.60 | 5.84 | 5.43 | 0.23 | 8.02 | 7.43 | 7.91 | 7.80 | 7.40 | 0.28 |
| UQCRB | 7381 | 7.36 | 7.42 | 7.28 | 6.92 | 7.49 | 0.22 | 9.48 | 9.22 | 9.22 | 9.74 | 9.39 | 0.21 |
| RBM9 | 23543 | 5.44 | 5.88 | 4.99 | 5.16 | 5.20 | 0.34 | 7.70 | 7.88 | 7.26 | 7.38 | 7.03 | 0.34 |
| RAD23A | 5886 | 9.41 | 9.43 | 9.24 | 8.93 | 9.20 | 0.20 | 11.45 | 11.17 | 11.66 | 11.31 | 11.19 | 0.20 |
| NDUFB9 | 4715 | 8.59 | 9.28 | 8.68 | 8.55 | 8.92 | 0.30 | 11.18 | 10.95 | 10.74 | 10.89 | 10.79 | 0.17 |
| C9orf23 | 138716 | 6.87 | 6.97 | 7.20 | 6.40 | 7.06 | 0.30 | 8.95 | 9.15 | 9.14 | 9.03 | 8.71 | 0.18 |
| PTGES2 | 80142 | 5.40 | 5.17 | 5.10 | 5.16 | 5.49 | 0.17 | 7.63 | 7.28 | 7.32 | 7.61 | 6.94 | 0.28 |
| TTC32 | 130502 | 3.41 | 4.26 | 4.93 | 4.23 | 5.03 | 0.65 | 6.12 | 6.24 | 6.60 | 6.60 | 6.65 | 0.24 |
| PDHA1 | 5160 | 8.47 | 8.88 | 8.32 | 8.08 | 8.55 | 0.29 | 10.58 | 10.46 | 10.38 | 10.74 | 10.43 | 0.15 |
| HSD17B7 | 51478 | 7.35 | 8.37 | 8.49 | 8.32 | 7.74 | 0.49 | 9.96 | 9.88 | 10.86 | 10.20 | 9.63 | 0.47 |
| SIRT5 | 23408 | 6.53 | 6.49 | 6.42 | 5.64 | 6.66 | 0.40 | 8.01 | 8.52 | 8.31 | 8.37 | 8.74 | 0.27 |
| TRAP1 | 10131 | 6.55 | 6.65 | 6.54 | 6.04 | 6.62 | 0.25 | 8.62 | 8.71 | 8.41 | 8.42 | 8.37 | 0.15 |
| ST3GAL3 | 6487 | 5.47 | 5.87 | 5.43 | 5.47 | 5.64 | 0.18 | 7.75 | 7.48 | 7.23 | 7.97 | 7.58 | 0.28 |
| MRPL2 | 51069 | 6.35 | 6.15 | 6.20 | 6.56 | 6.33 | 0.16 | 8.00 | 8.47 | 8.75 | 8.27 | 8.19 | 0.29 |
| PSCD1 | 9267 | 3.19 | 4.63 | 4.05 | 4.11 | 3.67 | 0.54 | 5.81 | 6.30 | 5.91 | 5.72 | 5.99 | 0.22 |
| MID1IP1 | 58526 | 6.53 | 6.23 | 6.74 | 6.58 | 6.64 | 0.19 | 9.08 | 8.48 | 8.49 | 8.67 | 8.04 | 0.38 |
| PDHX | 8050 | 6.58 | 7.39 | 6.70 | 7.13 | 6.92 | 0.33 | 8.90 | 8.89 | 8.51 | 9.36 | 9.09 | 0.31 |
| BLCAP | 10904 | 7.71 | 8.41 | 7.42 | 7.83 | 7.98 | 0.37 | 10.19 | 10.03 | 9.82 | 9.67 | 9.63 | 0.24 |
| AHDC1 | 27245 | 5.64 | 5.69 | 5.91 | 4.99 | 4.95 | 0.44 | 7.27 | 7.50 | 7.73 | 7.20 | 7.38 | 0.21 |
| PER2 | 8864 | 6.38 | 6.24 | 6.46 | 5.77 | 6.03 | 0.28 | 8.24 | 8.30 | 8.28 | 8.06 | 7.89 | 0.18 |
| CUL5 | 8065 | 6.22 | 6.71 | 5.78 | 6.47 | 6.41 | 0.35 | 8.23 | 8.59 | 8.45 | 8.09 | 8.12 | 0.22 |
| PPP3CB | 5532 | 8.34 | 8.81 | 8.86 | 7.83 | 8.57 | 0.42 | 10.67 | 10.52 | 10.33 | 10.31 | 10.38 | 0.15 |
| ATPAF2 | 91647 | 4.70 | 5.22 | 3.85 | 3.95 | 4.47 | 0.56 | 6.69 | 6.41 | 6.03 | 6.22 | 6.40 | 0.24 |
| C6orf136 | 221545 | 6.41 | 6.02 | 6.25 | 6.28 | 6.45 | 0.17 | 8.48 | 8.28 | 7.81 | 8.25 | 8.11 | 0.25 |
| FXR2 | 9513 | 4.79 | 4.66 | 4.71 | 4.56 | 4.85 | 0.11 | 6.45 | 6.23 | 7.07 | 6.75 | 6.61 | 0.32 |
| GFM1 | 85476 | 7.63 | 7.44 | 7.57 | 8.56 | 7.37 | 0.48 | 9.62 | 9.75 | 9.35 | 9.79 | 9.57 | 0.17 |
| TIE1 | 7075 | 3.29 | 2.94 | 3.07 | 3.74 | 3.10 | 0.31 | 5.01 | 4.83 | 5.05 | 5.55 | 5.16 | 0.27 |
| INPP5A | 3632 | 7.80 | 7.69 | 7.58 | 8.22 | 7.75 | 0.25 | 9.63 | 9.76 | 9.46 | 9.66 | 9.96 | 0.18 |
| POPDC3 | 64208 | 6.23 | 6.55 | 5.94 | 6.59 | 6.68 | 0.31 | 8.57 | 8.01 | 8.00 | 8.54 | 8.27 | 0.28 |
| DGCR6 | 8214 | 8.50 | 8.63 | 8.47 | 7.91 | 8.66 | 0.30 | 10.09 | 10.47 | 10.14 | 10.19 | 10.66 | 0.24 |
| NDUFV2 | 4729 | 9.61 | 9.48 | 9.75 | 9.43 | 9.56 | 0.13 | 11.22 | 11.30 | 11.30 | 11.61 | 11.75 | 0.23 |
| KLF9 | 687 | 8.66 | 8.30 | 8.42 | 7.49 | 8.13 | 0.44 | 10.26 | 10.26 | 10.23 | 9.72 | 9.87 | 0.26 |
| COQ6 | 51004 | 6.31 | 6.36 | 6.05 | 5.96 | 6.47 | 0.21 | 7.86 | 8.30 | 7.96 | 8.35 | 8.02 | 0.22 |
| DDX59 | 83479 | 5.67 | 5.30 | 5.31 | 5.96 | 5.40 | 0.28 | 7.36 | 7.67 | 7.33 | 7.35 | 7.19 | 0.18 |
| UGP2 | 7360 | 8.37 | 8.91 | 8.29 | 7.91 | 8.77 | 0.40 | 10.33 | 10.19 | 10.15 | 10.05 | 10.72 | 0.26 |
| UCKL1 | 54963 | 6.52 | 6.52 | 6.38 | 6.01 | 6.47 | 0.22 | 8.15 | 8.35 | 8.20 | 8.32 | 8.07 | 0.12 |
| TSPYL1 | 7259 | 7.44 | 6.69 | 7.59 | 7.71 | 7.74 | 0.43 | 9.28 | 8.97 | 9.39 | 9.20 | 9.50 | 0.20 |
| SLMAP | 7871 | 7.46 | 7.55 | 7.36 | 7.71 | 7.32 | 0.16 | 9.30 | 9.47 | 9.02 | 9.18 | 9.54 | 0.21 |
| RBJ | 51277 | 4.33 | 4.63 | 4.54 | 3.51 | 4.39 | 0.45 | 5.98 | 6.27 | 6.04 | 5.98 | 6.22 | 0.14 |
| BTBD6 | 90135 | 5.95 | 5.57 | 5.99 | 5.77 | 6.06 | 0.20 | 7.83 | 7.57 | 7.54 | 7.99 | 7.48 | 0.22 |
| AES | 166 | 8.86 | 8.72 | 8.76 | 8.10 | 9.22 | 0.41 | 10.45 | 10.27 | 10.81 | 10.44 | 10.74 | 0.23 |
| STAT5B | 6777 | 5.45 | 5.10 | 5.73 | 5.27 | 5.24 | 0.24 | 7.46 | 7.14 | 7.21 | 7.08 | 6.90 | 0.20 |
| NSFL1C | 55968 | 6.34 | 6.66 | 6.33 | 7.15 | 6.55 | 0.33 | 8.60 | 8.52 | 8.18 | 8.49 | 8.22 | 0.19 |
| ZNF291 | 49855 | 4.25 | 4.47 | 4.49 | 3.94 | 4.48 | 0.24 | 6.05 | 6.05 | 6.16 | 6.21 | 6.14 | 0.07 |
| ACYP1 | 97 | 6.20 | 6.76 | 7.15 | 5.82 | 6.52 | 0.51 | 8.13 | 8.49 | 8.37 | 8.08 | 8.25 | 0.17 |
| CS | 1431 | 9.44 | 9.45 | 9.54 | 9.47 | 9.33 | 0.07 | 11.27 | 11.19 | 11.11 | 11.26 | 11.25 | 0.07 |
| CYC1 | 1537 | 9.18 | 9.28 | 8.81 | 9.51 | 9.15 | 0.25 | 10.74 | 11.14 | 10.83 | 11.10 | 10.97 | 0.17 |
| CHCHD3 | 54927 | 6.74 | 6.00 | 6.82 | 7.31 | 6.96 | 0.48 | 8.43 | 8.39 | 8.43 | 8.76 | 8.64 | 0.16 |
| MRPL33 | 9553 | 9.14 | 9.27 | 9.01 | 8.87 | 9.42 | 0.22 | 10.60 | 11.06 | 10.65 | 11.05 | 11.16 | 0.26 |
| KIAA1787 | 84461 | 3.85 | 3.23 | 4.14 | 3.25 | 4.19 | 0.47 | 5.33 | 5.39 | 5.72 | 5.46 | 5.55 | 0.15 |
| ZNF768 | 79724 | 4.49 | 4.04 | 4.56 | 4.78 | 4.41 | 0.27 | 6.16 | 5.76 | 6.47 | 6.47 | 6.17 | 0.29 |
| CAPN6 | 827 | 5.67 | 4.85 | 5.39 | 5.38 | 5.88 | 0.39 | 6.99 | 6.95 | 7.39 | 7.37 | 7.20 | 0.20 |
| CCDC43 | 124808 | 6.12 | 6.53 | 5.73 | 6.23 | 6.33 | 0.30 | 7.97 | 7.98 | 7.73 | 7.97 | 8.04 | 0.12 |
| JPH2 | 57158 | 4.45 | 3.79 | 4.22 | 4.21 | 4.26 | 0.24 | 5.77 | 5.82 | 6.24 | 5.89 | 5.90 | 0.19 |
| NDUFA9 | 4704 | 8.00 | 7.96 | 7.70 | 7.58 | 8.01 | 0.20 | 9.51 | 9.48 | 9.63 | 9.75 | 9.54 | 0.11 |
| SDHD | 6392 | 7.96 | 8.25 | 7.84 | 7.78 | 7.88 | 0.19 | 9.69 | 9.73 | 9.55 | 9.69 | 9.70 | 0.07 |
| VDAC3 | 7419 | 9.77 | 10.27 | 10.09 | 10.11 | 9.88 | 0.20 | 11.66 | 11.80 | 11.75 | 11.71 | 11.84 | 0.07 |
| WDR23 | 80344 | 4.58 | 4.74 | 4.74 | 3.81 | 4.71 | 0.40 | 5.93 | 6.46 | 6.37 | 6.17 | 6.27 | 0.20 |
| C9orf5 | 23731 | 6.90 | 7.90 | 7.26 | 7.13 | 7.20 | 0.37 | 8.93 | 9.25 | 9.15 | 8.79 | 8.88 | 0.19 |
| DGCR6 | 8214 | 8.79 | 9.09 | 8.79 | 8.05 | 8.79 | 0.39 | 10.20 | 10.70 | 10.29 | 10.24 | 10.69 | 0.25 |
| ATP5G1 | 516 | 8.92 | 8.95 | 8.59 | 8.43 | 9.00 | 0.25 | 10.50 | 10.47 | 10.25 | 10.68 | 10.58 | 0.16 |
| NDUFB10 | 4716 | 9.55 | 9.70 | 9.67 | 9.48 | 9.64 | 0.09 | 11.35 | 11.35 | 11.09 | 11.55 | 11.27 | 0.17 |
| DULLARD | 23399 | 7.00 | 6.70 | 7.34 | 6.48 | 7.03 | 0.33 | 8.78 | 8.23 | 8.97 | 8.49 | 8.63 | 0.28 |
| THRAP4 | 9862 | 5.26 | 5.83 | 5.26 | 5.34 | 5.30 | 0.24 | 7.42 | 7.13 | 6.66 | 7.17 | 7.17 | 0.28 |
| ZNF124 | 7678 | 0.00 | 0.77 | 0.00 | 0.00 | 0.00 | 0.34 | 2.07 | 2.68 | 1.63 | 1.38 | 1.54 | 0.52 |
| ATP5S | 27109 | 4.49 | 5.60 | 4.99 | 4.52 | 5.27 | 0.48 | 6.49 | 7.12 | 6.34 | 6.37 | 7.08 | 0.39 |
| MRPL38 | 64978 | 6.86 | 6.81 | 7.00 | 7.22 | 7.07 | 0.16 | 8.80 | 8.80 | 8.63 | 8.74 | 8.49 | 0.13 |
| ATG4A | 115201 | 5.47 | 5.61 | 5.11 | 5.30 | 5.44 | 0.19 | 6.95 | 7.38 | 6.68 | 7.29 | 7.13 | 0.28 |
| PPP1R2 | 5504 | 7.01 | 7.53 | 6.70 | 7.55 | 7.22 | 0.36 | 9.13 | 9.22 | 8.68 | 9.00 | 8.43 | 0.33 |
| FLII | 2314 | 6.13 | 5.93 | 6.19 | 5.94 | 5.85 | 0.15 | 7.73 | 7.63 | 7.72 | 7.81 | 7.59 | 0.08 |
| KIF21A | 55605 | 4.74 | 5.42 | 5.17 | 5.85 | 5.22 | 0.41 | 6.81 | 6.91 | 6.62 | 7.16 | 7.35 | 0.29 |
| NDUFC1 | 4717 | 7.85 | 7.83 | 7.79 | 7.33 | 7.83 | 0.22 | 9.33 | 9.16 | 9.58 | 9.53 | 9.44 | 0.17 |
| HSF2 | 3298 | 6.87 | 7.08 | 6.82 | 7.01 | 6.61 | 0.18 | 8.97 | 8.50 | 8.48 | 8.49 | 8.30 | 0.25 |
| APOO | 79135 | 6.89 | 6.91 | 6.37 | 6.61 | 6.92 | 0.24 | 8.34 | 8.34 | 8.42 | 8.56 | 8.33 | 0.10 |
| HDHD1A | 8226 | 4.73 | 4.98 | 4.88 | 5.22 | 5.10 | 0.19 | 6.87 | 6.95 | 6.24 | 6.60 | 6.48 | 0.29 |
| REV1 | 51455 | 6.31 | 6.33 | 6.09 | 5.83 | 6.22 | 0.21 | 7.72 | 8.10 | 7.66 | 7.67 | 7.86 | 0.18 |
| C20orf7 | 79133 | 6.67 | 7.28 | 6.88 | 7.48 | 7.12 | 0.32 | 8.73 | 8.90 | 7.94 | 9.25 | 8.84 | 0.48 |
| CALCOCO2 | 10241 | 4.39 | 4.36 | 4.77 | 4.26 | 4.21 | 0.22 | 6.34 | 5.89 | 6.21 | 6.03 | 5.71 | 0.25 |
| PDE4A | 5141 | 3.71 | 3.74 | 4.26 | 4.20 | 4.30 | 0.29 | 5.76 | 5.25 | 5.84 | 5.95 | 5.62 | 0.27 |
| NDUFA10 | 4705 | 7.72 | 7.29 | 7.87 | 7.68 | 7.53 | 0.22 | 9.39 | 9.20 | 9.01 | 9.53 | 9.15 | 0.21 |
| DENND2C | 163259 | 5.81 | 6.38 | 5.80 | 7.26 | 5.93 | 0.62 | 8.04 | 7.92 | 7.26 | 8.51 | 7.63 | 0.47 |
| ECSIT | 51295 | 5.39 | 4.83 | 5.46 | 4.95 | 5.25 | 0.27 | 7.00 | 6.77 | 6.64 | 6.91 | 6.74 | 0.14 |
| CCDC101 | 112869 | 3.74 | 3.22 | 3.29 | 3.92 | 3.50 | 0.30 | 5.22 | 4.94 | 5.44 | 5.41 | 4.81 | 0.28 |
| NDUFV1 | 4723 | 8.44 | 8.25 | 8.68 | 7.87 | 8.58 | 0.32 | 10.24 | 10.08 | 10.01 | 9.84 | 9.80 | 0.18 |
| DDX59 | 83479 | 5.12 | 4.80 | 4.64 | 4.71 | 5.00 | 0.20 | 6.46 | 6.50 | 6.73 | 6.43 | 6.29 | 0.16 |
| FBXO3 | 26273 | 4.74 | 5.07 | 4.91 | 4.41 | 4.69 | 0.25 | 6.33 | 6.54 | 6.12 | 6.53 | 6.46 | 0.17 |
| DIP2C | 22982 | 6.19 | 5.52 | 5.57 | 5.85 | 5.59 | 0.28 | 7.65 | 7.03 | 7.24 | 7.75 | 7.19 | 0.31 |
| SFXN4 | 119559 | 7.24 | 6.89 | 6.68 | 7.09 | 7.29 | 0.25 | 8.54 | 8.66 | 8.78 | 8.78 | 8.56 | 0.12 |
| KALRN | 8997 | 4.04 | 4.30 | 4.32 | 4.32 | 4.24 | 0.12 | 5.68 | 5.56 | 5.82 | 5.96 | 6.31 | 0.29 |
| NR1D1 | 9572 | 0.00 | 1.07 | 1.81 | 2.74 | 0.58 | 1.07 | 2.04 | 2.63 | 3.67 | 3.95 | 2.00 | 0.91 |
| COX5A | 9377 | 10.36 | 10.86 | 10.19 | 10.34 | 10.47 | 0.25 | 12.10 | 11.90 | 11.94 | 12.06 | 12.27 | 0.15 |
| C5orf32 | 84418 | 7.77 | 7.35 | 7.87 | 7.89 | 8.04 | 0.26 | 9.40 | 9.40 | 9.57 | 9.21 | 9.39 | 0.13 |
| APIP | 51074 | 6.10 | 6.04 | 6.20 | 5.61 | 6.38 | 0.29 | 7.61 | 7.76 | 7.82 | 7.69 | 7.48 | 0.13 |
| RBM17 | 84991 | 7.17 | 7.36 | 7.25 | 7.16 | 7.11 | 0.10 | 8.89 | 8.88 | 8.65 | 8.82 | 8.83 | 0.10 |
| DIAPH2 | 1730 | 5.24 | 4.94 | 5.15 | 5.91 | 5.15 | 0.37 | 7.33 | 6.77 | 6.49 | 7.13 | 6.69 | 0.34 |
| ARAF | 369 | 6.26 | 5.82 | 6.34 | 5.67 | 6.11 | 0.29 | 7.78 | 7.60 | 7.71 | 7.64 | 7.49 | 0.11 |
| XPA | 7507 | 4.41 | 4.72 | 4.69 | 3.84 | 4.47 | 0.35 | 5.85 | 5.98 | 6.38 | 5.87 | 6.04 | 0.21 |
| HIF1AN | 55662 | 5.89 | 6.20 | 5.95 | 5.89 | 5.97 | 0.13 | 7.74 | 7.62 | 7.52 | 7.64 | 7.37 | 0.14 |
| MDH2 | 4191 | 9.61 | 9.26 | 9.38 | 9.49 | 9.66 | 0.16 | 11.12 | 11.04 | 11.12 | 11.30 | 10.79 | 0.18 |
| RAF1 | 5894 | 6.35 | 6.88 | 6.11 | 7.07 | 6.42 | 0.40 | 8.27 | 8.24 | 8.10 | 8.29 | 7.87 | 0.18 |
| FRAT2 | 23401 | 5.27 | 5.00 | 4.89 | 5.30 | 4.98 | 0.19 | 6.86 | 6.90 | 6.37 | 6.64 | 6.58 | 0.22 |
| C8orf38 | 137682 | 5.58 | 6.21 | 5.26 | 5.53 | 5.78 | 0.36 | 7.31 | 7.42 | 6.97 | 7.34 | 7.20 | 0.17 |
| MTRF1 | 9617 | 5.91 | 5.54 | 5.96 | 5.88 | 6.10 | 0.21 | 7.28 | 7.41 | 7.47 | 7.71 | 7.39 | 0.16 |
| SETD3 | 84193 | 8.46 | 8.79 | 8.09 | 8.48 | 8.63 | 0.26 | 10.03 | 10.14 | 10.01 | 10.26 | 9.90 | 0.14 |
| CHMP6 | 79643 | 1.68 | 2.77 | 3.57 | 3.12 | 3.02 | 0.71 | 3.67 | 4.66 | 4.96 | 4.54 | 4.20 | 0.50 |
| SDHB | 6390 | 9.40 | 9.67 | 9.14 | 9.22 | 9.55 | 0.22 | 10.87 | 10.97 | 10.93 | 11.08 | 11.00 | 0.08 |
| KEAP1 | 9817 | 6.00 | 5.82 | 6.23 | 6.31 | 5.74 | 0.25 | 7.59 | 7.48 | 7.63 | 7.69 | 7.56 | 0.08 |
| PRKAR2A | 5576 | 3.26 | 3.77 | 3.86 | 4.27 | 2.61 | 0.64 | 4.94 | 4.92 | 5.54 | 5.75 | 4.41 | 0.54 |
| SAMM50 | 25813 | 7.56 | 7.24 | 7.82 | 7.76 | 7.56 | 0.23 | 9.26 | 9.17 | 9.20 | 9.38 | 8.69 | 0.27 |
| C9orf86 | 55684 | 6.57 | 5.83 | 6.33 | 6.38 | 6.47 | 0.29 | 7.78 | 7.89 | 7.95 | 7.81 | 7.89 | 0.07 |
| CSDA | 8531 | 11.22 | 11.26 | 11.17 | 11.33 | 11.12 | 0.08 | 12.61 | 12.78 | 12.85 | 12.78 | 12.81 | 0.09 |
| TRIM43 | 129868 | 4.75 | 4.14 | 4.31 | 4.59 | 4.75 | 0.28 | 6.04 | 5.62 | 6.08 | 6.53 | 6.01 | 0.32 |
| LOC88523 | 88523 | 7.98 | 8.46 | 8.22 | 8.11 | 8.19 | 0.18 | 9.74 | 9.85 | 9.50 | 9.92 | 9.69 | 0.16 |
| COQ4 | 51117 | 5.21 | 5.06 | 5.65 | 5.15 | 5.75 | 0.31 | 6.68 | 6.97 | 7.17 | 6.69 | 7.02 | 0.22 |
| MRPS28 | 28957 | 7.10 | 7.54 | 7.05 | 7.39 | 7.16 | 0.21 | 8.72 | 9.03 | 8.36 | 8.83 | 8.98 | 0.27 |
| ZCD1 | 55847 | 7.50 | 7.48 | 7.42 | 7.16 | 7.55 | 0.16 | 9.13 | 8.80 | 8.52 | 9.05 | 9.26 | 0.29 |
| MRPL46 | 26589 | 7.35 | 7.38 | 7.21 | 7.79 | 7.21 | 0.24 | 8.89 | 9.01 | 8.83 | 8.93 | 8.86 | 0.07 |
| MRPS18A | 55168 | 6.43 | 6.10 | 6.42 | 6.29 | 6.57 | 0.18 | 7.94 | 8.05 | 7.65 | 7.84 | 7.84 | 0.15 |
| TPD52L3 | 89882 | 4.31 | 3.77 | 4.26 | 4.19 | 4.37 | 0.24 | 5.67 | 5.18 | 6.07 | 5.84 | 5.65 | 0.33 |
| ANKRD40 | 91369 | 5.35 | 5.26 | 5.11 | 5.46 | 5.38 | 0.13 | 6.46 | 6.73 | 6.98 | 6.87 | 6.94 | 0.21 |
| NUCKS1 | 64710 | 5.88 | 4.89 | 5.38 | 5.72 | 5.80 | 0.41 | 7.66 | 6.61 | 6.48 | 7.11 | 7.24 | 0.48 |
| KIAA0460 | 23248 | 4.30 | 3.68 | 4.74 | 4.41 | 3.93 | 0.42 | 5.91 | 5.26 | 6.09 | 5.47 | 5.74 | 0.33 |
| KCNJ10 | 3766 | 5.85 | 5.07 | 5.62 | 5.59 | 5.71 | 0.30 | 6.84 | 6.83 | 7.16 | 7.34 | 7.05 | 0.22 |
| KLHDC3 | 116138 | 9.44 | 9.60 | 9.28 | 9.07 | 9.49 | 0.21 | 11.05 | 10.88 | 10.69 | 10.67 | 10.88 | 0.16 |
| ABCA12 | 26154 | 5.19 | 4.94 | 5.22 | 5.10 | 5.49 | 0.20 | 6.53 | 6.26 | 6.97 | 6.76 | 6.69 | 0.27 |
| CRNKL1 | 51340 | 3.34 | 4.12 | 3.80 | 3.95 | 2.94 | 0.48 | 4.95 | 5.51 | 5.14 | 5.11 | 4.71 | 0.29 |
| NLRX1 | 79671 | 6.30 | 5.95 | 5.90 | 5.54 | 6.35 | 0.33 | 7.63 | 7.56 | 7.21 | 7.30 | 7.58 | 0.19 |
| ZNF438 | 220929 | 5.36 | 4.83 | 5.07 | 5.71 | 4.84 | 0.38 | 6.84 | 6.61 | 6.17 | 6.86 | 6.55 | 0.28 |
| MRPS9 | 64965 | 6.55 | 6.16 | 6.50 | 6.68 | 6.51 | 0.20 | 7.78 | 7.76 | 8.24 | 8.09 | 7.72 | 0.23 |
| ELSPBP1 | 64100 | 5.42 | 4.39 | 5.56 | 5.28 | 5.59 | 0.49 | 6.46 | 6.20 | 7.04 | 6.91 | 6.80 | 0.35 |
| UQCRFS1 | 7386 | 9.87 | 10.32 | 9.51 | 10.30 | 9.96 | 0.34 | 11.42 | 11.48 | 11.37 | 11.59 | 11.27 | 0.12 |
| FOXD4L2 | 100036519 | 6.44 | 5.94 | 6.33 | 6.20 | 6.50 | 0.22 | 7.65 | 7.38 | 8.02 | 7.95 | 7.56 | 0.27 |
| CCDC44 | 51204 | 6.84 | 6.94 | 6.85 | 6.74 | 6.98 | 0.09 | 8.22 | 8.39 | 7.93 | 8.51 | 8.34 | 0.22 |
| POLDIP2 | 26073 | 6.13 | 6.34 | 6.13 | 5.98 | 6.17 | 0.13 | 7.71 | 7.60 | 7.40 | 7.56 | 7.49 | 0.11 |
| APPL1 | 26060 | 6.36 | 6.68 | 6.54 | 6.28 | 6.50 | 0.16 | 7.95 | 7.84 | 7.84 | 7.86 | 7.88 | 0.05 |
| KTN1 | 3895 | 4.26 | 4.16 | 3.74 | 4.96 | 3.77 | 0.49 | 5.58 | 5.36 | 5.37 | 6.29 | 5.29 | 0.41 |
| PDCD2L | 84306 | 5.58 | 5.53 | 5.75 | 5.22 | 5.61 | 0.20 | 6.79 | 7.13 | 7.10 | 6.90 | 6.75 | 0.18 |
| DEXI | 28955 | 8.42 | 8.42 | 7.95 | 8.31 | 8.56 | 0.23 | 9.85 | 9.72 | 9.50 | 9.82 | 9.74 | 0.14 |
| NCOA1 | 8648 | 7.29 | 7.46 | 7.41 | 7.22 | 7.50 | 0.12 | 8.81 | 8.80 | 8.65 | 8.70 | 8.86 | 0.09 |
| FH | 2271 | 8.55 | 8.58 | 8.60 | 8.69 | 8.58 | 0.05 | 10.08 | 9.91 | 9.72 | 10.03 | 10.19 | 0.18 |
| TRIM24 | 8805 | 6.46 | 6.36 | 6.35 | 6.49 | 6.33 | 0.07 | 7.76 | 7.85 | 7.58 | 7.96 | 7.73 | 0.14 |
| SLC19A2 | 10560 | 5.05 | 4.47 | 5.13 | 5.02 | 5.10 | 0.28 | 6.28 | 6.17 | 6.70 | 6.21 | 6.25 | 0.21 |
| GIN1 | 54826 | 4.26 | 4.94 | 4.15 | 4.05 | 4.24 | 0.35 | 5.61 | 6.08 | 5.21 | 5.58 | 5.97 | 0.34 |
| SAP18 | 10284 | 6.22 | 5.88 | 6.21 | 6.12 | 6.07 | 0.14 | 7.30 | 7.20 | 7.40 | 7.65 | 7.71 | 0.22 |
| KBTBD2 | 25948 | 7.82 | 7.93 | 7.55 | 8.60 | 7.78 | 0.40 | 9.07 | 9.00 | 9.24 | 9.91 | 9.20 | 0.36 |
| MEF2D | 4209 | 5.71 | 6.14 | 5.91 | 5.90 | 6.13 | 0.18 | 7.18 | 7.17 | 7.29 | 7.57 | 7.31 | 0.16 |
| IMMT | 10989 | 8.83 | 8.86 | 8.74 | 8.97 | 8.75 | 0.09 | 10.21 | 10.16 | 9.96 | 10.30 | 10.25 | 0.14 |
| NDUFAF1 | 51103 | 6.60 | 6.84 | 6.51 | 6.54 | 6.70 | 0.13 | 8.00 | 8.17 | 8.14 | 7.66 | 7.95 | 0.20 |
| ZNF511 | 118472 | 7.25 | 7.39 | 7.47 | 7.28 | 7.20 | 0.11 | 8.42 | 8.90 | 8.47 | 8.66 | 8.88 | 0.22 |
| PREB | 10113 | 6.39 | 6.01 | 6.12 | 6.74 | 6.55 | 0.30 | 8.13 | 7.25 | 7.67 | 7.98 | 7.51 | 0.35 |
| MATN4 | 8785 | 0.58 | 1.93 | 1.20 | 0.00 | 1.26 | 0.73 | 1.49 | 3.54 | 2.77 | 1.32 | 2.56 | 0.93 |
| MRPS15 | 64960 | 8.46 | 8.42 | 8.32 | 8.00 | 8.47 | 0.19 | 9.65 | 9.86 | 9.61 | 9.67 | 9.55 | 0.12 |
| KIAA0182 | 23199 | 6.27 | 6.42 | 6.32 | 6.07 | 5.95 | 0.19 | 7.74 | 7.59 | 7.49 | 7.45 | 7.39 | 0.13 |
| PSEN1 | 5663 | 2.38 | 2.61 | 2.72 | 2.93 | 1.68 | 0.48 | 3.74 | 3.58 | 4.39 | 4.04 | 3.19 | 0.46 |
| NFYC | 4802 | 6.97 | 7.18 | 7.12 | 6.98 | 6.88 | 0.12 | 8.28 | 8.59 | 8.53 | 8.20 | 8.06 | 0.23 |
| IDH3B | 3420 | 8.79 | 8.99 | 8.87 | 9.23 | 8.70 | 0.21 | 10.33 | 10.23 | 10.13 | 10.36 | 10.07 | 0.13 |
| LAIR1 | 3903 | 3.36 | 3.09 | 3.71 | 3.29 | 3.07 | 0.26 | 4.58 | 4.55 | 4.74 | 4.56 | 4.61 | 0.08 |
| ACOX1 | 51 | 4.86 | 5.18 | 4.55 | 4.82 | 5.02 | 0.23 | 6.27 | 6.49 | 6.03 | 6.13 | 6.04 | 0.19 |
| HNRPL | 3191 | 6.06 | 5.45 | 6.13 | 6.19 | 6.19 | 0.32 | 7.25 | 7.20 | 7.41 | 7.31 | 7.31 | 0.08 |
| GARNL1 | 253959 | 4.43 | 4.28 | 4.69 | 4.54 | 4.54 | 0.15 | 5.92 | 5.81 | 5.71 | 5.84 | 5.59 | 0.13 |
| NDUFA4 | 4697 | 11.15 | 11.27 | 11.32 | 10.90 | 11.31 | 0.18 | 12.39 | 12.56 | 12.22 | 12.56 | 12.61 | 0.16 |
| CAPZA2 | 830 | 8.93 | 9.22 | 9.16 | 9.29 | 9.13 | 0.13 | 10.44 | 10.64 | 10.16 | 10.60 | 10.24 | 0.21 |
| LNX2 | 222484 | 4.55 | 5.01 | 4.87 | 4.33 | 4.26 | 0.33 | 5.94 | 6.39 | 5.94 | 5.19 | 5.78 | 0.43 |
| MRPS7 | 51081 | 7.43 | 7.60 | 7.16 | 7.66 | 7.59 | 0.20 | 8.66 | 8.83 | 8.72 | 8.72 | 8.72 | 0.06 |
| OXR1 | 55074 | 7.95 | 7.97 | 8.18 | 7.82 | 8.00 | 0.13 | 9.17 | 9.39 | 9.24 | 9.26 | 9.07 | 0.12 |
| UBAP2 | 55833 | 7.54 | 7.41 | 7.51 | 7.83 | 7.44 | 0.17 | 8.79 | 8.83 | 8.88 | 8.80 | 8.62 | 0.10 |
| PAIP2 | 51247 | 7.84 | 8.44 | 8.13 | 8.10 | 8.25 | 0.22 | 9.37 | 9.36 | 9.37 | 9.51 | 9.33 | 0.07 |
| LAPTM4B | 55353 | 8.05 | 8.22 | 8.44 | 7.98 | 8.38 | 0.20 | 9.36 | 9.55 | 9.86 | 9.10 | 9.37 | 0.28 |
| ZNF323 | 64288 | 4.55 | 4.46 | 4.18 | 3.96 | 4.05 | 0.26 | 5.62 | 5.91 | 5.17 | 5.23 | 5.43 | 0.30 |
| PSMA2 | 5683 | 6.42 | 6.60 | 6.18 | 6.48 | 6.20 | 0.18 | 7.46 | 7.94 | 7.16 | 7.78 | 7.70 | 0.30 |
| OXA1L | 5018 | 7.26 | 7.44 | 7.56 | 7.15 | 7.30 | 0.16 | 8.61 | 8.47 | 8.69 | 8.55 | 8.49 | 0.09 |
| PHB | 5245 | 7.88 | 7.87 | 7.70 | 8.16 | 7.91 | 0.16 | 9.35 | 9.33 | 8.74 | 9.13 | 9.07 | 0.25 |
| PCM1 | 5108 | 7.58 | 7.91 | 7.71 | 7.32 | 7.47 | 0.22 | 8.76 | 8.66 | 8.93 | 8.73 | 8.90 | 0.11 |
| TPRKB | 51002 | 5.32 | 5.46 | 5.34 | 5.19 | 5.48 | 0.12 | 6.79 | 6.44 | 6.51 | 6.47 | 6.52 | 0.14 |
| UBE3B | 89910 | 6.67 | 6.84 | 6.98 | 6.68 | 6.74 | 0.13 | 8.23 | 7.70 | 8.22 | 7.82 | 7.87 | 0.24 |
| MRPL1 | 65008 | 7.68 | 7.78 | 7.68 | 7.62 | 7.65 | 0.06 | 8.72 | 9.17 | 8.70 | 9.00 | 8.73 | 0.21 |
| NARS2 | 79731 | 5.60 | 6.11 | 5.40 | 5.27 | 5.99 | 0.37 | 6.74 | 7.13 | 6.50 | 6.85 | 7.04 | 0.25 |
| NDUFAB1 | 4706 | 10.28 | 10.87 | 10.26 | 10.46 | 10.37 | 0.25 | 11.61 | 11.62 | 11.40 | 11.81 | 11.69 | 0.15 |
| SENP2 | 59343 | 4.93 | 5.39 | 4.91 | 5.50 | 4.94 | 0.29 | 6.40 | 6.31 | 6.01 | 6.44 | 6.25 | 0.17 |
| QDPR | 5860 | 8.05 | 7.89 | 8.33 | 7.98 | 7.80 | 0.20 | 9.25 | 9.12 | 9.28 | 9.17 | 8.91 | 0.15 |
| CWC15 | 51503 | 7.91 | 7.80 | 7.73 | 7.50 | 7.89 | 0.17 | 8.81 | 9.08 | 8.94 | 8.82 | 8.84 | 0.11 |
| PKD1 | 5310 | 5.89 | 5.96 | 6.42 | 5.42 | 6.32 | 0.40 | 7.27 | 7.19 | 7.20 | 6.72 | 7.26 | 0.23 |
| VPS4A | 27183 | 6.68 | 6.50 | 6.56 | 7.01 | 6.79 | 0.20 | 7.97 | 7.87 | 7.76 | 8.01 | 7.56 | 0.18 |
| MRPL16 | 54948 | 7.48 | 7.37 | 7.64 | 7.14 | 7.48 | 0.18 | 8.44 | 8.47 | 8.53 | 8.60 | 8.66 | 0.09 |
| MBNL1 | 4154 | 8.36 | 8.29 | 8.47 | 8.21 | 8.13 | 0.13 | 9.42 | 9.53 | 9.25 | 9.43 | 9.40 | 0.10 |
| UNC84A | 23353 | 6.77 | 6.83 | 7.13 | 6.56 | 6.49 | 0.25 | 8.05 | 7.79 | 8.03 | 7.67 | 7.82 | 0.16 |
| PSMF1 | 9491 | 8.04 | 8.16 | 8.06 | 8.17 | 7.69 | 0.20 | 9.13 | 9.21 | 8.96 | 9.14 | 9.22 | 0.10 |
| TBC1D14 | 57533 | 7.05 | 6.89 | 7.07 | 6.42 | 6.77 | 0.27 | 8.17 | 7.83 | 8.17 | 7.81 | 7.76 | 0.20 |
| PDPR | 55066 | 6.60 | 6.07 | 5.59 | 6.54 | 5.07 | 0.65 | 7.53 | 7.15 | 7.03 | 7.53 | 6.12 | 0.57 |
| VBP1 | 7411 | 7.01 | 7.62 | 6.83 | 6.90 | 7.24 | 0.32 | 8.05 | 8.36 | 8.20 | 8.16 | 8.31 | 0.12 |
| LOC91661 | 91661 | 2.43 | 1.00 | 1.72 | 2.23 | 1.81 | 0.55 | 3.28 | 2.23 | 2.89 | 3.09 | 3.19 | 0.42 |
| TMEM93 | 83460 | 8.80 | 8.93 | 8.46 | 9.31 | 8.86 | 0.30 | 9.99 | 10.03 | 9.76 | 10.06 | 9.99 | 0.12 |
| THRA | 7067 | 4.04 | 3.71 | 4.45 | 3.81 | 3.77 | 0.31 | 5.25 | 5.02 | 5.63 | 4.60 | 4.69 | 0.42 |
| PECI | 10455 | 8.65 | 9.09 | 9.12 | 8.98 | 8.75 | 0.21 | 9.89 | 10.02 | 9.98 | 9.96 | 10.12 | 0.08 |
| TMEM111 | 55831 | 8.75 | 8.66 | 8.66 | 8.95 | 8.78 | 0.12 | 9.86 | 9.50 | 10.08 | 9.85 | 9.79 | 0.21 |
| CHMP6 | 79643 | 6.49 | 6.36 | 6.10 | 6.05 | 6.31 | 0.18 | 7.55 | 7.54 | 6.93 | 7.21 | 7.28 | 0.26 |
| MRPS24 | 64951 | 8.96 | 8.86 | 9.02 | 9.35 | 8.96 | 0.19 | 10.15 | 10.08 | 10.08 | 10.14 | 9.88 | 0.11 |
| MRPS31 | 10240 | 6.94 | 7.27 | 7.10 | 7.24 | 7.01 | 0.14 | 8.19 | 8.22 | 8.15 | 8.07 | 8.09 | 0.06 |
| COPS4 | 51138 | 7.55 | 8.10 | 7.63 | 7.69 | 7.76 | 0.21 | 8.54 | 9.07 | 8.55 | 8.77 | 8.96 | 0.24 |
| KIAA1199 | 57214 | 11.57 | 10.72 | 10.02 | 11.25 | 11.26 | 0.61 | 0.00 | 0.00 | 1.63 | 1.58 | 0.00 | 0.88 |
| LOX | 4015 | 10.37 | 9.91 | 8.22 | 9.93 | 10.47 | 0.91 | 0.00 | 0.00 | 0.00 | 0.00 | 0.00 | 0.00 |
| PTX3 | 5806 | 10.66 | 9.56 | 6.85 | 9.02 | 10.66 | 1.57 | 2.04 | 0.00 | 0.00 | 0.00 | 0.00 | 0.91 |
| STC2 | 8614 | 9.85 | 10.45 | 7.96 | 11.73 | 10.04 | 1.36 | 3.19 | 0.00 | 0.85 | 0.58 | 1.54 | 1.22 |
| TUBB3 | 10381 | 9.31 | 8.18 | 9.65 | 8.85 | 8.46 | 0.60 | 0.00 | 0.00 | 0.00 | 0.49 | 1.72 | 0.75 |
| SCRG1 | 11341 | 10.63 | 10.14 | 9.66 | 10.24 | 10.89 | 0.48 | 0.26 | 1.89 | 2.70 | 2.00 | 3.70 | 1.26 |
| RGS4 | 5999 | 9.29 | 8.84 | 8.80 | 9.61 | 8.71 | 0.39 | 0.00 | 0.00 | 2.00 | 2.04 | 3.04 | 1.36 |
| CDKN1A | 1026 | 12.19 | 12.25 | 11.42 | 12.03 | 12.43 | 0.39 | 4.33 | 5.24 | 4.59 | 4.45 | 3.75 | 0.54 |
| KIAA1913 | 114801 | 9.73 | 9.18 | 8.52 | 8.82 | 8.91 | 0.45 | 1.20 | 1.00 | 0.14 | 1.32 | 3.86 | 1.40 |
| SCG2 | 7857 | 7.20 | 7.95 | 7.71 | 7.65 | 6.90 | 0.43 | 0.00 | 0.00 | 0.00 | 0.00 | 0.00 | 0.00 |
| TMEM166 | 84141 | 8.62 | 8.11 | 6.47 | 9.60 | 8.35 | 1.14 | 2.43 | 0.00 | 0.00 | 0.00 | 1.93 | 1.21 |
| MOXD1 | 26002 | 9.28 | 8.11 | 9.99 | 9.03 | 8.70 | 0.70 | 1.43 | 1.54 | 2.32 | 0.14 | 4.04 | 1.43 |
| ANPEP | 290 | 7.86 | 6.26 | 9.25 | 8.51 | 7.48 | 1.12 | 2.77 | 0.00 | 1.63 | 2.14 | 0.00 | 1.26 |
| CPA4 | 51200 | 6.40 | 8.29 | 5.60 | 6.39 | 6.92 | 1.00 | 0.00 | 1.26 | 0.00 | 0.00 | 0.00 | 0.56 |
| PYCR1 | 5831 | 7.80 | 6.65 | 6.30 | 6.04 | 7.99 | 0.88 | 0.00 | 0.00 | 1.72 | 0.00 | 0.93 | 0.78 |
| FN1 | 2335 | 6.46 | 5.87 | 6.66 | 6.16 | 6.72 | 0.36 | 0.00 | 0.00 | 0.00 | 0.00 | 0.00 | 0.00 |
| PSD3 | 23362 | 6.29 | 6.28 | 7.54 | 6.30 | 5.57 | 0.71 | 0.14 | 0.00 | 0.00 | 0.00 | 0.00 | 0.06 |
| TYMS | 7298 | 7.21 | 6.46 | 7.10 | 5.07 | 5.90 | 0.89 | 0.00 | 0.00 | 0.00 | 0.00 | 0.00 | 0.00 |
| C6orf65 | 221336 | 7.08 | 6.21 | 7.65 | 5.73 | 5.82 | 0.84 | 0.00 | 0.00 | 0.00 | 0.00 | 0.93 | 0.41 |
| FJX1 | 24147 | 7.34 | 7.31 | 7.16 | 9.02 | 7.29 | 0.78 | 0.00 | 3.02 | 0.00 | 2.10 | 1.77 | 1.34 |
| ALPL | 249 | 10.38 | 7.64 | 10.17 | 10.03 | 9.31 | 1.12 | 2.87 | 3.42 | 3.85 | 3.10 | 3.25 | 0.37 |
| SPOCD1 | 90853 | 6.54 | 5.99 | 6.06 | 6.05 | 6.60 | 0.29 | 0.00 | 0.49 | 0.00 | 0.00 | 0.00 | 0.22 |
| MGC15523 | 124565 | 7.01 | 7.41 | 5.82 | 6.14 | 7.77 | 0.83 | 2.32 | 1.32 | 0.00 | 0.00 | 0.00 | 1.06 |
| TIMP2 | 7077 | 6.33 | 5.82 | 6.77 | 5.81 | 6.49 | 0.42 | 0.00 | 0.00 | 0.77 | 0.00 | 0.00 | 0.34 |
| NUDT11 | 55190 | 5.83 | 6.43 | 6.00 | 6.26 | 5.75 | 0.29 | 0.00 | 0.00 | 0.00 | 0.00 | 0.00 | 0.00 |
| PNMA2 | 10687 | 5.79 | 5.90 | 6.10 | 5.90 | 6.55 | 0.30 | 0.00 | 0.00 | 0.00 | 0.00 | 0.00 | 0.00 |
| KDELR3 | 11015 | 6.36 | 5.83 | 5.79 | 5.11 | 7.13 | 0.75 | 0.00 | 0.00 | 0.00 | 0.00 | 0.00 | 0.00 |
| PLAUR | 5329 | 7.18 | 5.82 | 7.11 | 8.14 | 7.27 | 0.83 | 2.41 | 0.00 | 0.00 | 3.02 | 0.00 | 1.50 |
| ESM1 | 11082 | 6.53 | 6.51 | 7.21 | 6.35 | 6.82 | 0.34 | 0.00 | 0.00 | 0.00 | 1.54 | 2.00 | 0.98 |
| PTK7 | 5754 | 6.51 | 6.40 | 6.01 | 5.51 | 6.30 | 0.40 | 0.58 | 0.00 | 0.00 | 0.00 | 0.49 | 0.30 |
| CH25H | 9023 | 8.28 | 6.52 | 6.47 | 4.12 | 7.11 | 1.52 | 0.93 | 1.14 | 0.00 | 0.00 | 1.20 | 0.60 |
| TPBG | 7162 | 7.08 | 7.05 | 6.72 | 7.53 | 6.67 | 0.35 | 2.74 | 0.00 | 0.85 | 3.19 | 0.00 | 1.52 |
| MFAP2 | 4237 | 7.74 | 7.61 | 7.47 | 7.02 | 7.83 | 0.32 | 0.68 | 2.14 | 2.61 | 2.23 | 1.81 | 0.74 |
| LAMA1 | 284217 | 7.04 | 5.68 | 5.41 | 5.40 | 6.43 | 0.72 | 0.00 | 1.43 | 0.00 | 0.00 | 0.77 | 0.65 |
| KDELC1 | 79070 | 6.19 | 5.49 | 5.96 | 4.60 | 5.48 | 0.61 | 0.00 | 0.00 | 0.00 | 0.00 | 0.00 | 0.00 |
| C11orf70 | 85016 | 6.21 | 6.57 | 6.01 | 5.24 | 6.21 | 0.49 | 0.00 | 0.00 | 0.00 | 0.00 | 2.54 | 1.13 |
| MOXD1 | 26002 | 6.94 | 6.12 | 7.79 | 6.56 | 6.37 | 0.65 | 2.63 | 0.00 | 1.72 | 1.77 | 0.00 | 1.17 |
| TOP2A | 7153 | 6.38 | 5.84 | 6.68 | 3.84 | 4.49 | 1.23 | 0.00 | 0.00 | 0.00 | 0.00 | 0.00 | 0.00 |
| FN1 | 2335 | 5.30 | 5.87 | 5.61 | 4.50 | 5.91 | 0.58 | 0.00 | 0.00 | 0.00 | 0.00 | 0.00 | 0.00 |
| PLOD3 | 8985 | 6.41 | 5.96 | 5.57 | 5.71 | 6.44 | 0.40 | 2.96 | 0.00 | 0.00 | 0.00 | 0.00 | 1.33 |
| HIST1H2BK | 85236 | 6.37 | 5.39 | 6.10 | 6.41 | 6.26 | 0.42 | 0.00 | 0.00 | 1.96 | 0.00 | 1.63 | 0.99 |
| IGF2BP3 | 10643 | 5.20 | 5.15 | 6.17 | 6.70 | 4.54 | 0.87 | 0.00 | 0.00 | 0.93 | 0.00 | 0.00 | 0.41 |
| SLC1A1 | 6505 | 5.52 | 5.45 | 5.04 | 5.64 | 5.12 | 0.26 | 0.00 | 0.00 | 0.00 | 0.00 | 0.00 | 0.00 |
| LPIN2 | 9663 | 5.47 | 5.24 | 5.85 | 6.22 | 5.00 | 0.48 | 0.00 | 0.00 | 1.00 | 0.00 | 0.00 | 0.45 |
| GALNS | 2588 | 5.42 | 5.05 | 6.29 | 5.12 | 5.64 | 0.50 | 0.77 | 0.00 | 0.00 | 0.00 | 0.00 | 0.34 |
| CSGlcA-T | 54480 | 5.41 | 5.15 | 5.54 | 5.34 | 5.16 | 0.17 | 0.00 | 0.00 | 0.00 | 0.00 | 0.00 | 0.00 |
| TMEM119 | 338773 | 10.26 | 9.75 | 9.42 | 9.60 | 9.72 | 0.31 | 5.18 | 4.50 | 3.23 | 3.90 | 5.67 | 0.98 |
| BDKRB1 | 623 | 5.82 | 4.20 | 5.71 | 5.77 | 4.49 | 0.79 | 0.00 | 0.00 | 0.00 | 0.00 | 0.00 | 0.00 |
| ARMC9 | 80210 | 6.36 | 5.61 | 6.71 | 4.22 | 5.97 | 0.96 | 0.93 | 0.00 | 0.85 | 0.00 | 1.20 | 0.56 |
| PLOD2 | 5352 | 9.94 | 9.73 | 9.31 | 9.31 | 10.44 | 0.47 | 4.15 | 5.34 | 4.86 | 3.57 | 4.93 | 0.70 |
| LOC493869 | 493869 | 9.23 | 9.17 | 9.18 | 8.78 | 9.41 | 0.23 | 3.34 | 4.34 | 4.19 | 3.26 | 4.81 | 0.67 |
| HIF1A | 3091 | 5.38 | 6.21 | 5.80 | 5.56 | 5.20 | 0.39 | 1.89 | 0.26 | 0.00 | 0.00 | 0.38 | 0.79 |
| GALNT12 | 79695 | 5.92 | 4.89 | 4.41 | 4.83 | 5.87 | 0.68 | 0.00 | 0.00 | 0.00 | 0.49 | 0.00 | 0.22 |
| CTHRC1 | 115908 | 11.51 | 10.50 | 11.03 | 11.18 | 11.09 | 0.36 | 6.18 | 5.53 | 5.99 | 6.44 | 5.78 | 0.35 |
| NPAS1 | 4861 | 6.05 | 5.78 | 3.54 | 4.69 | 5.78 | 1.05 | 0.00 | 0.58 | 0.00 | 0.00 | 0.00 | 0.26 |
| PDLIM4 | 8572 | 5.26 | 6.59 | 4.67 | 7.34 | 5.83 | 1.06 | 0.14 | 0.38 | 0.00 | 1.96 | 2.23 | 1.07 |
| C18orf56 | 494514 | 5.88 | 5.36 | 6.06 | 3.56 | 4.03 | 1.12 | 0.00 | 0.00 | 0.00 | 0.00 | 0.00 | 0.00 |
| CCDC80 | 151887 | 5.83 | 4.80 | 5.75 | 5.53 | 5.64 | 0.41 | 1.63 | 1.14 | 0.00 | 0.00 | 0.00 | 0.78 |
| TIMP1 | 7076 | 14.03 | 13.75 | 13.75 | 13.81 | 14.03 | 0.14 | 8.82 | 8.74 | 9.04 | 8.79 | 9.42 | 0.28 |
| LOC400406 | 400406 | 4.60 | 5.61 | 4.85 | 4.64 | 4.82 | 0.41 | 0.00 | 0.00 | 0.00 | 0.00 | 0.00 | 0.00 |
| MMP14 | 4323 | 4.94 | 4.57 | 5.33 | 4.79 | 4.85 | 0.28 | 0.00 | 0.00 | 0.00 | 0.00 | 0.00 | 0.00 |
| FBXO22 | 26263 | 5.73 | 6.18 | 5.27 | 5.46 | 6.31 | 0.45 | 2.00 | 0.00 | 0.00 | 0.00 | 2.49 | 1.24 |
| BCAS4 | 55653 | 5.25 | 5.40 | 5.45 | 4.81 | 5.74 | 0.34 | 0.00 | 1.38 | 1.07 | 0.00 | 0.00 | 0.68 |
| CA12 | 771 | 8.72 | 7.19 | 9.91 | 8.71 | 7.51 | 1.09 | 4.06 | 3.23 | 3.47 | 3.66 | 3.43 | 0.31 |
| RGS17 | 26575 | 5.19 | 3.92 | 5.27 | 6.11 | 3.83 | 0.98 | 0.00 | 0.26 | 0.00 | 0.00 | 0.00 | 0.12 |
| MTHFD1L | 25902 | 7.69 | 7.65 | 6.77 | 6.77 | 7.85 | 0.53 | 2.10 | 2.94 | 3.02 | 1.93 | 2.70 | 0.50 |
| ABHD2 | 11057 | 4.83 | 4.46 | 4.97 | 5.46 | 4.94 | 0.36 | 0.00 | 0.00 | 0.68 | 0.00 | 0.14 | 0.29 |
| PCNXL2 | 80003 | 5.69 | 5.64 | 4.58 | 4.35 | 5.52 | 0.64 | 2.10 | 0.00 | 0.00 | 0.00 | 0.00 | 0.94 |
| NFASC | 23114 | 4.55 | 4.73 | 4.66 | 4.94 | 4.77 | 0.15 | 0.00 | 0.00 | 0.00 | 0.00 | 0.00 | 0.00 |
| SPCS3 | 60559 | 5.79 | 5.77 | 5.18 | 5.43 | 5.92 | 0.30 | 0.93 | 0.00 | 0.00 | 1.38 | 2.17 | 0.93 |
| LOXL3 | 84695 | 7.41 | 6.78 | 6.33 | 7.70 | 8.08 | 0.71 | 3.19 | 1.96 | 2.10 | 2.29 | 3.15 | 0.59 |
| SERPINB8 | 5271 | 4.71 | 4.69 | 3.91 | 5.64 | 4.63 | 0.61 | 0.00 | 0.00 | 0.00 | 0.00 | 0.00 | 0.00 |
| COL1A1 | 1277 | 13.54 | 13.30 | 12.17 | 12.13 | 13.67 | 0.75 | 8.67 | 7.92 | 7.35 | 8.32 | 9.07 | 0.66 |
| SCN9A | 6335 | 5.69 | 4.92 | 4.03 | 3.62 | 5.20 | 0.85 | 0.00 | 0.00 | 0.00 | 0.00 | 0.00 | 0.00 |
| PLOD2 | 5352 | 5.03 | 4.91 | 4.13 | 3.47 | 6.04 | 0.97 | 0.00 | 0.00 | 0.14 | 0.00 | 0.00 | 0.06 |
| CEP55 | 55165 | 5.55 | 5.20 | 5.49 | 3.09 | 4.10 | 1.07 | 0.00 | 0.00 | 0.00 | 0.00 | 0.00 | 0.00 |
| TMED3 | 23423 | 11.04 | 10.46 | 10.77 | 6.28 | 10.85 | 2.02 | 6.16 | 6.66 | 6.82 | 0.00 | 6.57 | 2.94 |
| ASPHD2 | 57168 | 5.48 | 5.11 | 4.04 | 5.33 | 5.39 | 0.59 | 0.00 | 0.00 | 0.77 | 0.00 | 1.43 | 0.65 |
| SERPINE2 | 5270 | 13.12 | 12.80 | 11.51 | 12.56 | 13.43 | 0.73 | 8.68 | 7.51 | 7.63 | 8.08 | 8.42 | 0.50 |
| DNAJC10 | 54431 | 5.35 | 5.61 | 4.34 | 4.64 | 5.01 | 0.52 | 0.38 | 1.49 | 0.00 | 0.00 | 0.00 | 0.64 |
| TMEM136 | 219902 | 7.26 | 6.93 | 7.84 | 6.44 | 7.57 | 0.55 | 1.72 | 2.04 | 3.20 | 2.20 | 3.83 | 0.88 |
| SPOCK1 | 6695 | 10.23 | 9.80 | 11.29 | 9.40 | 10.53 | 0.72 | 4.87 | 5.73 | 5.75 | 5.58 | 6.30 | 0.51 |
| GBA | 2629 | 7.65 | 6.36 | 7.25 | 6.64 | 7.40 | 0.54 | 1.58 | 1.81 | 3.14 | 2.46 | 3.29 | 0.77 |
| ALCAM | 214 | 8.87 | 8.32 | 9.08 | 9.84 | 8.31 | 0.63 | 4.09 | 3.86 | 4.15 | 4.49 | 4.88 | 0.40 |
| VDR | 7421 | 4.39 | 4.89 | 4.45 | 3.95 | 5.19 | 0.48 | 0.00 | 0.00 | 0.00 | 0.00 | 0.00 | 0.00 |
| MYO1E | 4643 | 5.34 | 4.27 | 5.19 | 6.20 | 5.13 | 0.69 | 0.00 | 0.00 | 0.93 | 2.43 | 0.00 | 1.06 |
| CEECAM1 | 51148 | 8.98 | 7.93 | 8.67 | 7.84 | 9.06 | 0.58 | 4.19 | 3.88 | 4.49 | 3.04 | 4.36 | 0.58 |
| SGMS2 | 166929 | 4.47 | 4.37 | 3.49 | 5.41 | 5.04 | 0.73 | 0.00 | 0.00 | 0.00 | 0.26 | 0.00 | 0.12 |
| THBS2 | 7058 | 11.08 | 10.48 | 10.70 | 8.52 | 10.95 | 1.05 | 5.28 | 6.02 | 6.19 | 5.24 | 6.51 | 0.57 |
| FEZ1 | 9638 | 5.16 | 5.30 | 5.34 | 4.32 | 5.55 | 0.48 | 1.07 | 0.00 | 2.14 | 0.00 | 0.00 | 0.96 |
| HM13 | 81502 | 4.67 | 4.29 | 3.69 | 5.07 | 4.69 | 0.52 | 0.00 | 0.00 | 0.00 | 0.00 | 0.00 | 0.00 |
| SKIV2L | 6499 | 7.27 | 6.47 | 7.54 | 6.84 | 7.27 | 0.42 | 3.71 | 1.26 | 3.17 | 2.83 | 2.04 | 0.96 |
| FKBP11 | 51303 | 9.69 | 8.65 | 8.90 | 9.19 | 9.84 | 0.51 | 4.94 | 3.84 | 4.99 | 5.26 | 5.13 | 0.57 |
| NME1 | 4830 | 3.96 | 4.69 | 3.02 | 5.29 | 5.09 | 0.93 | 0.00 | 0.00 | 0.00 | 0.00 | 0.00 | 0.00 |
| VEGFC | 7424 | 7.68 | 7.75 | 8.00 | 7.75 | 7.48 | 0.19 | 3.04 | 4.17 | 3.50 | 2.72 | 3.22 | 0.55 |
| CDKN2A | 1029 | 5.46 | 5.73 | 5.11 | 5.93 | 6.00 | 0.37 | 0.00 | 0.93 | 2.04 | 1.00 | 2.35 | 0.94 |
| CPZ | 8532 | 4.43 | 4.66 | 3.60 | 6.09 | 4.53 | 0.90 | 0.00 | 0.00 | 0.00 | 1.38 | 0.00 | 0.62 |
| UAP1L1 | 91373 | 4.53 | 5.20 | 4.92 | 5.16 | 4.42 | 0.36 | 0.49 | 0.00 | 2.04 | 0.00 | 0.00 | 0.88 |
| NME5 | 8382 | 4.49 | 4.33 | 5.53 | 2.87 | 4.25 | 0.95 | 0.00 | 0.00 | 0.00 | 0.00 | 0.00 | 0.00 |
| PI4K2A | 55361 | 4.60 | 4.60 | 4.57 | 4.87 | 4.36 | 0.18 | 0.00 | 0.58 | 0.00 | 1.00 | 0.00 | 0.46 |
| CDH11 | 1009 | 10.16 | 9.59 | 10.45 | 9.72 | 9.82 | 0.35 | 5.33 | 5.63 | 5.21 | 5.44 | 6.75 | 0.63 |
| TRIP13 | 9319 | 5.16 | 3.83 | 4.69 | 4.00 | 3.58 | 0.65 | 0.00 | 0.00 | 0.00 | 0.00 | 0.00 | 0.00 |
| RAB33A | 9363 | 5.52 | 5.64 | 3.80 | 5.81 | 5.30 | 0.81 | 0.00 | 1.54 | 0.00 | 2.56 | 0.77 | 1.09 |
| STAT1 | 6772 | 7.82 | 7.72 | 6.48 | 6.96 | 7.94 | 0.63 | 3.80 | 2.96 | 2.23 | 3.61 | 3.22 | 0.61 |
| C2orf37 | 80067 | 4.45 | 4.23 | 3.50 | 4.36 | 4.43 | 0.40 | 0.00 | 0.00 | 0.00 | 0.00 | 0.00 | 0.00 |
| SHKBP1 | 92799 | 5.11 | 4.49 | 5.48 | 4.36 | 5.01 | 0.46 | 1.54 | 0.00 | 1.58 | 0.00 | 0.38 | 0.80 |
| PGM2 | 55276 | 5.69 | 5.61 | 5.27 | 5.71 | 5.62 | 0.18 | 2.14 | 1.68 | 1.00 | 0.93 | 1.26 | 0.51 |
| P4HA2 | 8974 | 10.57 | 10.39 | 9.83 | 9.51 | 10.58 | 0.48 | 6.45 | 5.57 | 5.99 | 5.89 | 6.09 | 0.32 |
| TBC1D2 | 55357 | 7.90 | 6.69 | 7.60 | 7.53 | 7.26 | 0.46 | 2.87 | 3.25 | 3.23 | 3.22 | 3.70 | 0.30 |
| CDC7 | 8317 | 4.75 | 4.58 | 5.13 | 5.15 | 4.64 | 0.27 | 0.00 | 0.49 | 2.56 | 0.58 | 0.00 | 1.06 |
| ARL13B | 200894 | 4.19 | 4.74 | 3.98 | 4.92 | 4.26 | 0.40 | 0.00 | 0.00 | 0.00 | 0.00 | 1.54 | 0.69 |
| C11orf41 | 25758 | 6.23 | 4.98 | 4.73 | 7.61 | 5.76 | 1.15 | 2.70 | 0.00 | 1.32 | 3.15 | 1.58 | 1.24 |
| C20orf42 | 55612 | 4.53 | 3.50 | 4.39 | 5.14 | 2.98 | 0.86 | 0.00 | 0.00 | 0.00 | 0.00 | 0.00 | 0.00 |
| TRAM2 | 9697 | 9.20 | 9.36 | 10.30 | 10.40 | 8.97 | 0.66 | 5.28 | 5.19 | 5.90 | 5.46 | 5.90 | 0.34 |
| CCDC80 | 151887 | 7.88 | 7.53 | 7.66 | 7.02 | 8.34 | 0.48 | 4.87 | 2.68 | 3.41 | 3.42 | 3.58 | 0.80 |
| TGFBI | 7045 | 13.03 | 12.81 | 12.84 | 12.99 | 13.03 | 0.10 | 8.38 | 8.41 | 9.30 | 8.86 | 9.34 | 0.46 |
| DNAJC3 | 5611 | 4.31 | 3.51 | 3.45 | 4.84 | 4.26 | 0.59 | 0.00 | 0.00 | 0.00 | 0.00 | 0.00 | 0.00 |
| KDELR3 | 11015 | 9.79 | 9.16 | 9.34 | 8.65 | 9.69 | 0.46 | 4.74 | 5.22 | 5.07 | 5.23 | 6.02 | 0.47 |
| THY1 | 7070 | 11.20 | 10.12 | 10.73 | 10.29 | 11.17 | 0.50 | 6.54 | 5.58 | 6.80 | 6.91 | 7.39 | 0.67 |
| UBXD6 | 7993 | 4.83 | 4.74 | 4.11 | 4.90 | 4.77 | 0.32 | 0.00 | 1.96 | 0.00 | 0.26 | 0.85 | 0.83 |
| LEPRE1 | 64175 | 8.93 | 8.05 | 8.11 | 7.78 | 8.80 | 0.50 | 4.02 | 4.13 | 4.69 | 4.40 | 4.26 | 0.26 |
| FAP | 2191 | 10.19 | 9.92 | 9.88 | 8.84 | 9.89 | 0.52 | 5.70 | 5.52 | 5.00 | 5.64 | 6.68 | 0.61 |
| CNN2 | 1265 | 9.08 | 8.50 | 9.12 | 7.88 | 8.99 | 0.53 | 4.81 | 4.80 | 5.17 | 3.55 | 5.13 | 0.66 |
| SERPINH1 | 871 | 10.92 | 10.51 | 10.18 | 9.73 | 11.06 | 0.54 | 6.26 | 6.26 | 6.62 | 6.47 | 6.68 | 0.20 |
| SLC7A1 | 6541 | 9.17 | 8.71 | 8.19 | 8.78 | 9.04 | 0.38 | 4.66 | 4.43 | 5.46 | 3.90 | 5.41 | 0.67 |
| SLC44A3 | 126969 | 4.15 | 5.49 | 4.12 | 4.17 | 4.83 | 0.60 | 0.00 | 2.74 | 0.00 | 0.00 | 0.00 | 1.23 |
| PLAU | 5328 | 9.29 | 9.93 | 10.14 | 9.61 | 8.72 | 0.56 | 5.10 | 5.54 | 5.33 | 6.06 | 5.65 | 0.36 |
| CCND1 | 595 | 11.02 | 10.93 | 10.88 | 12.40 | 10.90 | 0.66 | 7.46 | 6.06 | 7.54 | 7.55 | 7.54 | 0.66 |
| DKFZP564J0863 | 25923 | 6.84 | 5.91 | 7.00 | 6.77 | 6.35 | 0.44 | 3.12 | 2.83 | 2.93 | 2.00 | 2.17 | 0.49 |
| ELL2 | 22936 | 6.69 | 5.80 | 6.12 | 7.52 | 6.74 | 0.66 | 3.20 | 2.17 | 2.17 | 3.55 | 2.00 | 0.71 |
| ADM2 | 79924 | 7.51 | 7.20 | 5.35 | 5.53 | 7.65 | 1.12 | 3.72 | 3.12 | 1.14 | 2.83 | 2.68 | 0.96 |
| RCN3 | 57333 | 10.02 | 8.72 | 9.34 | 8.91 | 10.06 | 0.62 | 5.51 | 5.72 | 4.91 | 5.40 | 5.77 | 0.35 |
| TAF1A | 9015 | 3.63 | 4.23 | 4.00 | 5.64 | 3.60 | 0.84 | 0.00 | 0.00 | 0.00 | 1.43 | 0.00 | 0.64 |
| C14orf79 | 122616 | 5.48 | 5.07 | 5.45 | 5.79 | 5.64 | 0.27 | 1.38 | 1.07 | 1.14 | 1.32 | 2.93 | 0.77 |
| CDR2 | 1039 | 8.21 | 7.98 | 7.56 | 8.31 | 8.21 | 0.30 | 4.58 | 2.72 | 4.07 | 4.43 | 4.89 | 0.85 |
| TNFRSF10B | 8795 | 8.27 | 8.58 | 7.72 | 9.32 | 8.39 | 0.58 | 4.68 | 4.53 | 4.65 | 4.15 | 4.78 | 0.24 |
| SNAI2 | 6591 | 7.03 | 7.48 | 6.85 | 6.63 | 6.97 | 0.31 | 3.67 | 2.63 | 3.07 | 3.00 | 3.12 | 0.37 |
| HYAL3 | 8372 | 4.30 | 4.16 | 3.41 | 4.47 | 3.10 | 0.60 | 0.00 | 0.00 | 0.00 | 0.00 | 0.00 | 0.00 |
| QPCT | 25797 | 7.65 | 6.07 | 6.65 | 7.66 | 7.60 | 0.73 | 3.45 | 3.10 | 2.96 | 2.87 | 3.90 | 0.42 |
| G6PD | 2539 | 8.04 | 7.21 | 7.80 | 7.56 | 7.68 | 0.31 | 3.82 | 3.09 | 4.70 | 3.41 | 4.01 | 0.62 |
| SEC14L2 | 23541 | 4.12 | 3.72 | 3.91 | 4.19 | 4.37 | 0.25 | 0.00 | 0.00 | 0.00 | 0.00 | 1.07 | 0.48 |
| TBC1D8B | 54885 | 4.86 | 4.35 | 4.68 | 4.35 | 4.38 | 0.23 | 1.38 | 0.00 | 1.96 | 0.14 | 0.00 | 0.92 |
| CD248 | 57124 | 11.73 | 10.54 | 12.16 | 11.40 | 11.27 | 0.60 | 7.37 | 7.25 | 8.54 | 7.40 | 7.55 | 0.52 |
| GLIPR1 | 11010 | 8.53 | 6.91 | 8.98 | 7.91 | 8.39 | 0.79 | 4.20 | 4.25 | 4.97 | 3.50 | 4.83 | 0.59 |
| VCAN | 1462 | 10.61 | 10.15 | 10.02 | 9.25 | 10.74 | 0.59 | 5.77 | 5.68 | 6.87 | 6.41 | 7.11 | 0.64 |
| TTLL1 | 25809 | 5.34 | 4.61 | 5.25 | 3.58 | 5.10 | 0.72 | 1.58 | 0.00 | 2.14 | 0.00 | 1.26 | 0.96 |
| LOXL1 | 4016 | 7.39 | 7.87 | 7.60 | 6.41 | 7.70 | 0.58 | 4.07 | 3.00 | 4.50 | 2.66 | 3.87 | 0.77 |
| CHSY-2 | 337876 | 6.94 | 6.97 | 5.94 | 6.68 | 6.42 | 0.42 | 3.83 | 2.61 | 3.02 | 1.96 | 2.77 | 0.68 |
| CENPM | 79019 | 5.17 | 4.15 | 4.60 | 2.56 | 3.71 | 0.99 | 1.43 | 0.00 | 0.00 | 0.00 | 0.00 | 0.64 |
| CLIP3 | 25999 | 9.81 | 9.51 | 10.02 | 8.82 | 9.45 | 0.46 | 6.30 | 5.21 | 5.55 | 5.38 | 6.47 | 0.57 |
| ULBP1 | 80329 | 3.69 | 4.47 | 3.04 | 3.50 | 3.91 | 0.53 | 0.00 | 0.00 | 0.00 | 0.00 | 0.00 | 0.00 |
| FKBP10 | 60681 | 6.70 | 6.00 | 6.24 | 5.59 | 6.77 | 0.49 | 2.93 | 1.93 | 2.87 | 2.14 | 2.91 | 0.48 |
| PHGDH | 26227 | 10.81 | 10.49 | 10.38 | 10.33 | 10.76 | 0.22 | 6.46 | 7.07 | 7.64 | 6.28 | 6.81 | 0.54 |
| SUMF2 | 25870 | 7.50 | 7.14 | 7.02 | 7.04 | 7.48 | 0.24 | 3.20 | 3.19 | 4.02 | 4.29 | 2.96 | 0.58 |
| HTRA1 | 5654 | 12.45 | 11.71 | 12.89 | 12.06 | 11.62 | 0.53 | 8.35 | 8.20 | 8.72 | 8.17 | 8.82 | 0.30 |
| COL5A1 | 1289 | 11.56 | 11.63 | 10.48 | 10.62 | 11.86 | 0.63 | 7.71 | 7.26 | 7.21 | 7.77 | 7.74 | 0.27 |
| IKIP | 121457 | 7.69 | 7.34 | 6.90 | 7.63 | 7.57 | 0.32 | 3.47 | 3.85 | 4.51 | 3.66 | 3.23 | 0.49 |
| PDIA5 | 10954 | 7.62 | 6.58 | 9.28 | 6.41 | 7.24 | 1.15 | 4.43 | 3.14 | 5.07 | 3.22 | 2.91 | 0.94 |
| GPR172A | 79581 | 7.47 | 6.42 | 6.60 | 7.70 | 7.44 | 0.58 | 3.94 | 1.93 | 3.78 | 3.54 | 4.10 | 0.88 |
| C19orf10 | 56005 | 10.05 | 8.89 | 9.15 | 9.44 | 10.11 | 0.54 | 5.58 | 5.53 | 6.17 | 6.01 | 5.98 | 0.28 |
| PI4K2B | 55300 | 6.06 | 5.22 | 6.36 | 5.91 | 5.54 | 0.45 | 3.46 | 2.17 | 2.10 | 1.58 | 1.43 | 0.80 |
| TECT1 | 79600 | 8.40 | 7.75 | 8.80 | 7.68 | 8.02 | 0.47 | 4.74 | 3.84 | 4.52 | 4.56 | 4.72 | 0.37 |
| MASTL | 84930 | 4.00 | 3.96 | 4.15 | 3.74 | 3.32 | 0.32 | 0.00 | 0.93 | 0.00 | 0.00 | 0.00 | 0.41 |
| SRPX2 | 27286 | 7.65 | 7.55 | 9.09 | 7.45 | 8.15 | 0.68 | 3.93 | 4.54 | 5.19 | 2.96 | 5.06 | 0.91 |
| S100A11 | 6282 | 10.49 | 10.69 | 9.94 | 10.35 | 10.67 | 0.31 | 6.74 | 6.62 | 7.47 | 6.38 | 6.73 | 0.41 |
| ZNF365 | 22891 | 6.83 | 5.48 | 5.95 | 4.78 | 6.20 | 0.77 | 3.14 | 1.14 | 2.32 | 1.43 | 3.04 | 0.91 |
| LARP6 | 55323 | 8.99 | 9.23 | 8.81 | 8.34 | 9.10 | 0.35 | 4.46 | 5.53 | 6.03 | 5.04 | 5.26 | 0.58 |
| SLC25A17 | 10478 | 4.00 | 4.21 | 3.95 | 4.88 | 4.24 | 0.37 | 0.00 | 0.00 | 0.00 | 1.38 | 2.04 | 0.96 |
| CUTA | 51596 | 3.09 | 4.35 | 3.38 | 3.02 | 3.88 | 0.56 | 0.00 | 0.00 | 0.00 | 0.00 | 0.00 | 0.00 |
| MYO5A | 4644 | 7.22 | 7.47 | 6.67 | 7.11 | 7.17 | 0.29 | 4.04 | 3.85 | 3.38 | 2.94 | 3.79 | 0.44 |
| HAGHL | 84264 | 4.10 | 3.74 | 3.36 | 3.05 | 3.36 | 0.41 | 0.00 | 0.00 | 0.00 | 0.00 | 0.00 | 0.00 |
| ZMAT3 | 64393 | 9.42 | 9.67 | 9.47 | 8.35 | 9.56 | 0.54 | 5.81 | 5.67 | 5.83 | 5.86 | 5.67 | 0.09 |
| SLC22A18 | 5002 | 5.80 | 5.45 | 5.42 | 4.75 | 6.37 | 0.59 | 2.74 | 2.07 | 2.23 | 0.00 | 3.15 | 1.22 |
| DPP4 | 1803 | 8.14 | 6.38 | 6.99 | 7.97 | 6.75 | 0.77 | 4.41 | 2.56 | 4.06 | 3.87 | 3.92 | 0.70 |
| ARSB | 411 | 3.20 | 2.83 | 3.43 | 3.87 | 4.09 | 0.51 | 0.00 | 0.00 | 0.00 | 0.00 | 0.00 | 0.00 |
| C1orf102 | 127700 | 3.09 | 3.79 | 4.12 | 3.14 | 4.12 | 0.51 | 0.00 | 0.00 | 0.00 | 0.00 | 0.85 | 0.38 |
| SH3PXD2B | 285590 | 6.78 | 5.55 | 6.42 | 6.50 | 5.56 | 0.57 | 2.79 | 1.00 | 3.79 | 3.45 | 2.41 | 1.09 |
| VKORC1 | 79001 | 8.84 | 8.79 | 9.25 | 8.47 | 8.98 | 0.28 | 6.04 | 4.94 | 5.61 | 4.93 | 5.56 | 0.48 |
| CDR2L | 30850 | 6.75 | 5.26 | 5.66 | 6.33 | 6.33 | 0.60 | 2.87 | 1.26 | 2.00 | 3.78 | 3.15 | 0.99 |
| LOC150223 | 150223 | 3.31 | 3.72 | 3.42 | 3.74 | 3.66 | 0.20 | 0.00 | 0.49 | 0.00 | 0.00 | 0.14 | 0.21 |
| MAP1B | 4131 | 7.80 | 5.87 | 7.07 | 8.19 | 6.91 | 0.89 | 4.32 | 2.46 | 3.92 | 4.75 | 3.26 | 0.90 |
| FEZ1 | 9638 | 3.32 | 4.01 | 2.68 | 3.31 | 3.81 | 0.52 | 0.00 | 0.00 | 0.00 | 0.00 | 0.00 | 0.00 |
| ABCC3 | 8714 | 5.04 | 4.55 | 5.63 | 5.73 | 4.41 | 0.60 | 0.93 | 2.26 | 2.58 | 1.89 | 0.58 | 0.86 |
| GMIP | 51291 | 4.50 | 5.52 | 4.63 | 5.39 | 5.31 | 0.47 | 1.20 | 1.93 | 0.68 | 1.85 | 2.68 | 0.76 |
| FAM129B | 64855 | 10.44 | 10.22 | 10.98 | 10.52 | 10.21 | 0.32 | 7.02 | 6.61 | 7.20 | 7.28 | 7.43 | 0.31 |
| NACA | 4666 | 5.97 | 5.59 | 5.85 | 5.62 | 6.19 | 0.25 | 2.23 | 1.43 | 3.67 | 2.26 | 2.79 | 0.82 |
| DCBLD2 | 131566 | 9.09 | 8.23 | 9.07 | 9.72 | 8.84 | 0.54 | 5.79 | 5.63 | 5.76 | 5.33 | 5.64 | 0.18 |
| HHLA3 | 11147 | 3.22 | 3.69 | 3.10 | 2.83 | 3.95 | 0.46 | 0.00 | 0.00 | 0.00 | 0.00 | 0.00 | 0.00 |
| LRRC49 | 54839 | 5.45 | 5.19 | 5.54 | 5.86 | 5.22 | 0.27 | 2.61 | 2.26 | 1.54 | 2.10 | 1.96 | 0.39 |
| ECM1 | 1893 | 7.14 | 6.26 | 7.30 | 6.36 | 6.90 | 0.46 | 3.63 | 3.82 | 3.97 | 2.20 | 3.60 | 0.71 |
| NHEDC2 | 133308 | 3.22 | 4.11 | 2.93 | 2.85 | 3.64 | 0.53 | 0.00 | 0.00 | 0.00 | 0.00 | 0.00 | 0.00 |
| PPAPDC1B | 84513 | 6.09 | 5.55 | 5.54 | 5.52 | 6.20 | 0.33 | 2.29 | 2.14 | 2.70 | 1.81 | 3.25 | 0.56 |
| ANXA2 | 302 | 7.12 | 7.17 | 6.89 | 7.36 | 7.68 | 0.29 | 3.45 | 3.64 | 3.91 | 3.94 | 4.58 | 0.43 |
| ZC3HAV1 | 56829 | 4.13 | 3.64 | 2.35 | 3.83 | 3.20 | 0.69 | 0.38 | 0.00 | 0.14 | 0.00 | 0.00 | 0.17 |
| C20orf102 | 128434 | 2.89 | 3.95 | 3.26 | 2.41 | 4.10 | 0.71 | 0.00 | 0.00 | 0.00 | 0.00 | 0.00 | 0.00 |
| PIGK | 10026 | 7.61 | 7.02 | 7.38 | 6.70 | 7.60 | 0.39 | 3.34 | 3.64 | 4.00 | 3.98 | 4.79 | 0.54 |
| CHPF | 79586 | 8.83 | 7.73 | 9.59 | 7.99 | 8.34 | 0.74 | 5.03 | 4.79 | 5.40 | 5.08 | 5.71 | 0.36 |
| PPIB | 5479 | 11.62 | 10.86 | 11.35 | 11.12 | 11.55 | 0.31 | 7.95 | 7.97 | 8.31 | 7.88 | 7.96 | 0.17 |
| FNDC4 | 64838 | 6.05 | 5.82 | 6.70 | 5.48 | 5.89 | 0.45 | 3.31 | 2.00 | 2.94 | 2.00 | 3.29 | 0.66 |
| PDIA6 | 10130 | 7.64 | 6.85 | 7.43 | 6.81 | 7.67 | 0.42 | 4.36 | 3.95 | 4.39 | 2.91 | 4.43 | 0.64 |
| ACOT7 | 11332 | 7.85 | 8.97 | 8.26 | 7.74 | 8.39 | 0.49 | 4.49 | 5.15 | 4.86 | 4.89 | 5.49 | 0.37 |
| DC2 | 58505 | 9.96 | 9.68 | 9.58 | 9.16 | 10.17 | 0.39 | 6.06 | 6.25 | 6.83 | 6.35 | 6.77 | 0.34 |
| SH3BGRL3 | 83442 | 10.34 | 9.91 | 9.97 | 9.80 | 10.28 | 0.24 | 6.52 | 6.57 | 7.79 | 6.51 | 6.64 | 0.55 |
| SC4MOL | 6307 | 9.70 | 9.95 | 8.58 | 8.08 | 9.76 | 0.83 | 5.96 | 6.26 | 5.53 | 5.92 | 6.13 | 0.28 |
| RAB32 | 10981 | 8.29 | 8.29 | 7.75 | 7.87 | 8.97 | 0.48 | 4.61 | 4.89 | 5.29 | 5.00 | 5.19 | 0.26 |
| TP53I3 | 9540 | 8.07 | 7.46 | 7.13 | 6.29 | 8.32 | 0.80 | 4.47 | 4.12 | 4.87 | 3.15 | 4.50 | 0.65 |
| TKT | 7086 | 10.41 | 9.83 | 10.54 | 10.75 | 9.85 | 0.41 | 6.78 | 6.84 | 8.15 | 6.55 | 6.94 | 0.63 |
| FOXD1 | 2297 | 8.25 | 8.01 | 7.64 | 8.69 | 8.32 | 0.39 | 4.77 | 4.06 | 4.83 | 5.07 | 6.06 | 0.72 |
| SLC6A9 | 6536 | 7.26 | 5.96 | 5.97 | 6.29 | 7.05 | 0.61 | 3.79 | 2.04 | 3.29 | 3.86 | 3.45 | 0.74 |
| STIL | 6491 | 5.50 | 5.71 | 5.43 | 5.09 | 4.99 | 0.30 | 2.72 | 1.96 | 1.32 | 2.54 | 2.07 | 0.55 |
| ITGA5 | 3678 | 9.15 | 9.16 | 8.78 | 10.08 | 9.04 | 0.49 | 6.08 | 5.68 | 6.08 | 6.21 | 6.09 | 0.20 |
| C5orf34 | 375444 | 3.07 | 3.25 | 3.61 | 4.04 | 2.10 | 0.72 | 0.00 | 0.00 | 0.00 | 0.00 | 0.00 | 0.00 |
| DUSP14 | 11072 | 9.26 | 8.53 | 8.60 | 10.37 | 8.46 | 0.81 | 6.10 | 5.94 | 5.82 | 5.97 | 5.37 | 0.28 |
| IMPDH1 | 3614 | 7.67 | 6.93 | 7.34 | 8.05 | 7.58 | 0.41 | 4.19 | 3.64 | 5.18 | 4.04 | 4.49 | 0.57 |
| NUDT5 | 11164 | 9.12 | 8.53 | 8.75 | 8.60 | 9.05 | 0.26 | 5.65 | 5.43 | 5.87 | 5.29 | 5.85 | 0.25 |
| HMMR | 3161 | 4.14 | 3.79 | 4.92 | 2.49 | 2.72 | 1.01 | 0.26 | 0.00 | 1.85 | 0.00 | 0.00 | 0.81 |
| ADM | 133 | 11.07 | 10.41 | 10.67 | 10.99 | 10.39 | 0.32 | 7.99 | 7.47 | 7.99 | 6.88 | 7.30 | 0.47 |
| RIPK2 | 8767 | 6.96 | 6.97 | 6.44 | 6.32 | 7.02 | 0.33 | 3.34 | 4.17 | 4.22 | 2.56 | 3.55 | 0.68 |
| INA | 9118 | 2.51 | 3.52 | 3.02 | 4.29 | 2.81 | 0.70 | 0.00 | 0.26 | 0.00 | 0.00 | 0.00 | 0.12 |
| EXT1 | 2131 | 8.54 | 8.15 | 8.09 | 9.42 | 8.38 | 0.53 | 5.45 | 4.45 | 5.95 | 5.76 | 5.21 | 0.58 |
| MDK | 4192 | 3.62 | 2.77 | 3.38 | 3.02 | 3.64 | 0.38 | 0.00 | 0.00 | 0.68 | 0.00 | 0.00 | 0.30 |
| RAB34 | 83871 | 8.04 | 8.05 | 8.48 | 7.65 | 8.21 | 0.30 | 5.15 | 4.57 | 5.52 | 3.86 | 5.73 | 0.76 |
| EZH2 | 2146 | 3.39 | 3.63 | 3.09 | 2.41 | 3.00 | 0.46 | 0.00 | 0.00 | 0.00 | 0.00 | 0.00 | 0.00 |
| COL6A1 | 1291 | 11.90 | 11.05 | 11.97 | 11.43 | 11.37 | 0.39 | 8.45 | 8.11 | 8.29 | 8.51 | 8.89 | 0.29 |
| LAMP2 | 3920 | 8.69 | 8.28 | 8.81 | 7.48 | 8.60 | 0.53 | 5.83 | 5.51 | 5.61 | 3.73 | 5.77 | 0.88 |
| DAB2 | 1601 | 10.77 | 9.61 | 10.05 | 10.26 | 10.22 | 0.42 | 6.98 | 6.95 | 7.51 | 6.90 | 7.24 | 0.26 |
| COMT | 1312 | 4.96 | 4.71 | 4.00 | 4.08 | 5.36 | 0.58 | 2.17 | 2.32 | 0.00 | 1.14 | 2.17 | 0.99 |
| ITGB5 | 3693 | 8.35 | 8.39 | 8.71 | 7.84 | 8.24 | 0.31 | 5.31 | 4.97 | 5.73 | 5.21 | 5.02 | 0.30 |
| FTL | 2512 | 13.89 | 13.67 | 13.54 | 13.89 | 13.75 | 0.15 | 10.62 | 10.39 | 11.29 | 10.50 | 10.68 | 0.35 |
| BMP1 | 649 | 7.27 | 7.65 | 7.15 | 7.04 | 7.62 | 0.28 | 4.07 | 4.27 | 4.89 | 4.40 | 3.86 | 0.39 |
| OLFML3 | 56944 | 9.20 | 8.78 | 9.53 | 7.96 | 8.67 | 0.60 | 5.92 | 5.35 | 6.16 | 5.35 | 6.13 | 0.40 |
| ITPR3 | 3710 | 8.51 | 7.93 | 8.31 | 8.99 | 8.26 | 0.39 | 5.65 | 4.45 | 5.47 | 5.81 | 5.39 | 0.53 |
| CDK6 | 1021 | 7.17 | 8.23 | 6.78 | 9.16 | 7.83 | 0.93 | 4.60 | 4.63 | 4.49 | 5.24 | 5.00 | 0.32 |
| LY96 | 23643 | 9.41 | 8.58 | 8.93 | 9.46 | 9.28 | 0.37 | 6.11 | 6.49 | 6.20 | 5.87 | 5.81 | 0.27 |
| ALG2 | 85365 | 3.12 | 3.29 | 2.04 | 3.47 | 3.25 | 0.57 | 0.00 | 0.00 | 0.00 | 0.00 | 0.00 | 0.00 |
| COL1A2 | 1278 | 12.16 | 12.30 | 12.13 | 11.43 | 12.13 | 0.34 | 9.20 | 8.45 | 8.84 | 8.91 | 9.57 | 0.42 |
| EVC | 2121 | 3.20 | 2.94 | 3.20 | 2.83 | 2.98 | 0.17 | 0.00 | 0.00 | 0.00 | 0.00 | 0.00 | 0.00 |
| PRDX4 | 10549 | 11.05 | 10.78 | 11.23 | 10.50 | 10.95 | 0.28 | 7.68 | 8.11 | 7.95 | 7.71 | 7.94 | 0.18 |
| AMMECR1 | 9949 | 7.30 | 7.77 | 6.54 | 8.04 | 7.50 | 0.57 | 4.61 | 4.93 | 3.87 | 4.41 | 4.22 | 0.40 |
| FBXO4 | 26272 | 2.91 | 4.18 | 3.72 | 2.58 | 3.15 | 0.64 | 0.00 | 1.54 | 0.00 | 0.00 | 0.00 | 0.69 |
| GPR137B | 7107 | 7.39 | 7.53 | 7.82 | 7.58 | 7.51 | 0.16 | 4.82 | 4.33 | 4.55 | 4.26 | 4.96 | 0.30 |
| SLC10A7 | 84068 | 3.35 | 4.07 | 3.36 | 2.94 | 3.68 | 0.42 | 1.00 | 1.54 | 0.00 | 0.00 | 0.00 | 0.72 |
| TMEM44 | 93109 | 6.98 | 6.39 | 5.92 | 6.78 | 6.75 | 0.42 | 3.50 | 3.09 | 3.92 | 4.14 | 3.32 | 0.43 |
| TMEM97 | 27346 | 8.37 | 8.09 | 8.08 | 7.75 | 8.36 | 0.26 | 5.35 | 5.32 | 4.91 | 5.33 | 5.00 | 0.21 |
| AKAP10 | 11216 | 3.22 | 2.96 | 1.89 | 3.17 | 3.47 | 0.62 | 0.00 | 0.00 | 0.00 | 0.00 | 0.00 | 0.00 |
| NQO1 | 1728 | 9.99 | 9.99 | 9.46 | 10.63 | 9.23 | 0.55 | 6.38 | 7.59 | 6.95 | 7.02 | 6.66 | 0.45 |
| TUBA1C | 84790 | 11.38 | 11.25 | 11.26 | 12.37 | 11.38 | 0.48 | 8.10 | 8.49 | 8.51 | 8.93 | 8.97 | 0.36 |
| ST7L | 54879 | 2.20 | 3.43 | 3.75 | 3.49 | 3.46 | 0.61 | 0.00 | 0.00 | 0.85 | 0.00 | 0.85 | 0.46 |
| FAM14A | 83982 | 7.79 | 8.56 | 7.77 | 6.99 | 8.05 | 0.57 | 4.66 | 5.14 | 4.36 | 4.93 | 5.45 | 0.42 |
| ITPKA | 3706 | 3.39 | 2.94 | 3.14 | 2.29 | 2.83 | 0.41 | 0.00 | 0.00 | 0.00 | 0.00 | 0.00 | 0.00 |
| VKORC1 | 79001 | 12.07 | 11.86 | 12.27 | 12.14 | 12.05 | 0.15 | 9.28 | 9.25 | 9.17 | 8.88 | 9.23 | 0.16 |
| XYLB | 9942 | 3.17 | 3.07 | 2.81 | 3.20 | 3.17 | 0.16 | 0.00 | 0.85 | 0.00 | 0.00 | 0.00 | 0.38 |
| TRIM5 | 85363 | 3.19 | 3.28 | 3.04 | 2.66 | 2.32 | 0.40 | 0.00 | 0.00 | 0.00 | 0.00 | 0.00 | 0.00 |
| TXNDC5 | 81567 | 10.03 | 9.65 | 9.78 | 9.66 | 9.73 | 0.15 | 6.82 | 6.77 | 6.99 | 6.81 | 7.14 | 0.16 |
| TAGLN | 6876 | 12.92 | 12.87 | 13.12 | 11.97 | 13.07 | 0.47 | 9.85 | 9.50 | 11.04 | 9.63 | 9.63 | 0.63 |
| FEZ1 | 9638 | 8.60 | 8.04 | 8.73 | 8.23 | 8.47 | 0.28 | 5.04 | 5.62 | 5.97 | 5.15 | 6.00 | 0.45 |
| PRMT6 | 55170 | 6.81 | 6.11 | 6.92 | 6.85 | 6.37 | 0.35 | 3.69 | 3.51 | 3.72 | 3.94 | 3.94 | 0.18 |
| CKAP4 | 10970 | 9.80 | 9.02 | 8.69 | 9.08 | 9.40 | 0.42 | 5.91 | 6.44 | 6.49 | 6.31 | 6.61 | 0.27 |
| RPN2 | 6185 | 9.95 | 9.54 | 9.53 | 9.28 | 9.73 | 0.25 | 6.65 | 6.37 | 6.99 | 6.63 | 7.16 | 0.31 |
| NGFRAP1 | 27018 | 8.82 | 8.48 | 9.16 | 8.04 | 8.85 | 0.43 | 5.80 | 5.60 | 6.19 | 5.76 | 5.78 | 0.22 |
| FAM38A | 9780 | 9.18 | 8.23 | 8.70 | 9.36 | 8.74 | 0.44 | 6.26 | 5.67 | 6.44 | 5.77 | 5.87 | 0.33 |
| DAP | 1611 | 10.00 | 9.38 | 9.98 | 9.10 | 10.07 | 0.44 | 6.43 | 6.75 | 7.21 | 6.47 | 7.51 | 0.47 |
| CLIC3 | 9022 | 6.40 | 4.01 | 5.32 | 5.21 | 6.70 | 1.07 | 3.04 | 1.07 | 2.61 | 2.81 | 3.97 | 1.05 |
| LAMP2 | 3920 | 9.85 | 9.50 | 9.96 | 8.90 | 9.69 | 0.42 | 6.76 | 6.96 | 7.10 | 6.22 | 6.82 | 0.33 |
| CTSA | 5476 | 8.94 | 7.99 | 9.00 | 8.24 | 8.51 | 0.44 | 5.97 | 5.20 | 6.22 | 5.75 | 5.50 | 0.40 |
| PARVA | 55742 | 7.12 | 6.24 | 6.26 | 6.83 | 7.00 | 0.41 | 4.39 | 3.10 | 4.04 | 3.71 | 4.19 | 0.50 |
| PDIA4 | 9601 | 8.18 | 7.36 | 8.23 | 7.34 | 7.96 | 0.44 | 5.03 | 4.34 | 5.42 | 5.07 | 5.22 | 0.41 |
| WIPF2 | 147179 | 2.70 | 3.20 | 2.56 | 2.83 | 2.66 | 0.25 | 0.00 | 0.00 | 0.00 | 0.00 | 0.00 | 0.00 |
| FGF1 | 2246 | 2.41 | 2.17 | 2.77 | 4.56 | 3.10 | 0.94 | 0.00 | 0.00 | 0.00 | 1.07 | 0.00 | 0.48 |
| TMPO | 7112 | 3.38 | 3.79 | 2.49 | 3.25 | 3.69 | 0.51 | 0.68 | 0.85 | 0.00 | 0.00 | 1.14 | 0.51 |
| COL6A2 | 1292 | 11.02 | 10.51 | 11.51 | 10.54 | 10.16 | 0.52 | 8.14 | 7.31 | 8.04 | 8.40 | 7.93 | 0.40 |
| KDELR2 | 11014 | 9.59 | 9.43 | 9.32 | 9.13 | 9.96 | 0.31 | 6.65 | 6.72 | 7.39 | 5.90 | 6.95 | 0.54 |
| EFEMP2 | 30008 | 10.75 | 10.32 | 11.15 | 10.37 | 10.68 | 0.34 | 7.73 | 8.11 | 7.48 | 8.07 | 8.17 | 0.30 |
| MYO9B | 4650 | 6.80 | 6.05 | 7.31 | 8.49 | 6.67 | 0.92 | 4.19 | 3.71 | 4.80 | 4.72 | 4.18 | 0.45 |
| UGCGL2 | 55757 | 7.56 | 7.17 | 6.64 | 6.21 | 7.36 | 0.55 | 4.25 | 3.95 | 4.14 | 4.41 | 4.52 | 0.22 |
| SOCS1 | 8651 | 2.29 | 3.28 | 2.87 | 3.05 | 3.88 | 0.58 | 0.00 | 0.00 | 0.85 | 0.00 | 0.85 | 0.46 |
| CALU | 813 | 10.54 | 10.27 | 9.79 | 10.48 | 10.35 | 0.30 | 7.35 | 7.52 | 7.53 | 7.39 | 8.06 | 0.28 |
| HIST1H2BK | 85236 | 10.66 | 9.89 | 10.47 | 11.03 | 10.12 | 0.45 | 7.58 | 7.51 | 7.71 | 7.79 | 8.00 | 0.19 |
| PLK4 | 10733 | 4.01 | 3.69 | 3.45 | 2.41 | 2.66 | 0.68 | 1.38 | 0.00 | 1.26 | 0.00 | 0.00 | 0.72 |
| RAB31 | 11031 | 10.18 | 9.27 | 9.93 | 10.15 | 9.51 | 0.40 | 6.87 | 6.89 | 7.40 | 6.92 | 7.41 | 0.28 |
| SNAPC2 | 6618 | 7.19 | 5.99 | 7.14 | 7.51 | 6.95 | 0.58 | 4.52 | 3.88 | 4.50 | 4.57 | 3.78 | 0.39 |
| C10orf61 | 26123 | 6.00 | 5.59 | 5.49 | 5.69 | 6.14 | 0.28 | 3.15 | 2.93 | 3.45 | 2.96 | 2.94 | 0.22 |
| COL6A3 | 1293 | 12.25 | 11.23 | 12.19 | 12.00 | 11.80 | 0.41 | 9.27 | 8.64 | 9.55 | 9.10 | 9.45 | 0.36 |
| ANTXR2 | 118429 | 9.25 | 8.99 | 9.34 | 9.34 | 8.81 | 0.24 | 6.34 | 6.23 | 6.65 | 6.24 | 6.80 | 0.26 |
| ATOX1 | 475 | 10.26 | 9.36 | 10.08 | 9.97 | 10.48 | 0.42 | 7.05 | 7.43 | 7.42 | 7.45 | 7.34 | 0.17 |
| ITGB1 | 3688 | 8.59 | 8.84 | 9.24 | 7.60 | 9.01 | 0.64 | 5.38 | 5.87 | 6.19 | 5.84 | 6.58 | 0.45 |
| CALD1 | 800 | 11.48 | 11.31 | 11.24 | 10.22 | 11.22 | 0.50 | 8.26 | 7.90 | 8.79 | 8.27 | 8.82 | 0.39 |
| SKIL | 6498 | 2.96 | 3.94 | 3.12 | 4.67 | 3.93 | 0.69 | 0.00 | 1.63 | 0.00 | 2.63 | 1.00 | 1.12 |
| PGLS | 25796 | 9.50 | 9.00 | 9.31 | 8.83 | 9.35 | 0.27 | 6.40 | 6.40 | 7.19 | 6.38 | 6.32 | 0.37 |
| RCN1 | 5954 | 11.52 | 11.35 | 11.01 | 11.22 | 11.60 | 0.24 | 8.51 | 8.44 | 8.52 | 8.85 | 9.12 | 0.29 |
| FSCN1 | 6624 | 10.96 | 10.13 | 10.27 | 10.91 | 10.61 | 0.37 | 7.61 | 7.64 | 8.04 | 8.04 | 8.35 | 0.31 |
| TMEM14A | 28978 | 6.99 | 6.90 | 7.09 | 6.15 | 7.16 | 0.41 | 3.71 | 3.94 | 5.04 | 3.77 | 4.67 | 0.60 |
| LENG4 | 79143 | 7.71 | 7.44 | 7.01 | 7.54 | 7.79 | 0.31 | 5.14 | 4.49 | 5.19 | 4.93 | 4.63 | 0.31 |
| SLFN12 | 55106 | 5.91 | 6.06 | 5.37 | 6.10 | 5.77 | 0.29 | 2.56 | 3.75 | 3.36 | 3.19 | 3.32 | 0.43 |
| MVP | 9961 | 9.57 | 9.32 | 9.55 | 8.90 | 9.30 | 0.27 | 6.68 | 6.90 | 6.70 | 6.95 | 6.43 | 0.21 |
| CCNE2 | 9134 | 3.36 | 3.31 | 2.51 | 3.22 | 3.05 | 0.34 | 1.20 | 0.14 | 0.00 | 0.00 | 1.14 | 0.62 |
| IFI16 | 3428 | 8.61 | 8.30 | 9.35 | 8.19 | 8.07 | 0.51 | 6.05 | 5.34 | 6.10 | 5.73 | 6.36 | 0.39 |
| C4orf34 | 201895 | 9.07 | 8.70 | 9.37 | 8.62 | 9.00 | 0.30 | 5.96 | 6.20 | 7.08 | 6.26 | 6.32 | 0.42 |
| SLC30A5 | 64924 | 6.04 | 6.35 | 5.81 | 5.84 | 6.26 | 0.24 | 2.79 | 3.60 | 2.98 | 3.52 | 4.52 | 0.68 |
| GRN | 2896 | 8.75 | 8.23 | 9.01 | 7.79 | 8.39 | 0.47 | 5.88 | 5.45 | 6.53 | 5.82 | 5.60 | 0.41 |
| PGAM4 | 441531 | 8.71 | 9.00 | 8.50 | 8.61 | 9.04 | 0.24 | 6.16 | 5.83 | 6.58 | 6.04 | 6.38 | 0.29 |
| ACLY | 47 | 8.63 | 8.88 | 7.87 | 8.67 | 8.64 | 0.39 | 5.90 | 6.30 | 6.14 | 5.72 | 5.76 | 0.25 |
| EVC | 2121 | 6.68 | 6.77 | 6.92 | 6.61 | 6.37 | 0.21 | 3.83 | 3.88 | 4.45 | 3.69 | 4.65 | 0.42 |
| NRAS | 4893 | 5.92 | 6.59 | 5.94 | 6.42 | 6.23 | 0.30 | 3.80 | 4.03 | 3.04 | 3.51 | 3.88 | 0.39 |
| SMG6 | 23293 | 2.68 | 3.17 | 2.94 | 2.72 | 2.77 | 0.20 | 0.00 | 1.32 | 0.00 | 0.00 | 0.14 | 0.58 |
| THRA | 7067 | 8.84 | 8.82 | 8.69 | 8.02 | 9.57 | 0.55 | 6.41 | 6.46 | 6.00 | 5.80 | 6.48 | 0.31 |
| COPB2 | 9276 | 8.80 | 8.46 | 8.26 | 8.68 | 8.52 | 0.21 | 6.06 | 5.69 | 6.06 | 6.08 | 6.06 | 0.17 |
| MID1 | 4281 | 5.50 | 5.11 | 4.46 | 5.69 | 5.43 | 0.48 | 3.25 | 2.63 | 1.96 | 2.63 | 2.96 | 0.48 |
| TAX1BP3 | 30851 | 9.93 | 8.96 | 9.46 | 8.75 | 9.81 | 0.52 | 6.84 | 6.42 | 7.02 | 6.82 | 7.08 | 0.26 |
| ENO1 | 2023 | 11.66 | 11.15 | 11.56 | 11.67 | 11.75 | 0.24 | 9.05 | 9.11 | 9.12 | 9.04 | 8.76 | 0.15 |
| C20orf59 | 63910 | 2.14 | 2.79 | 2.32 | 3.26 | 2.17 | 0.48 | 0.00 | 0.00 | 0.00 | 0.00 | 0.00 | 0.00 |
| CASP4 | 837 | 8.85 | 8.86 | 8.62 | 8.25 | 8.90 | 0.27 | 6.16 | 5.69 | 6.63 | 6.10 | 6.34 | 0.34 |
| CD151 | 977 | 8.10 | 8.52 | 8.90 | 7.85 | 8.36 | 0.40 | 5.83 | 5.36 | 6.30 | 5.62 | 6.06 | 0.37 |
| NME1 | 4830 | 10.54 | 10.40 | 10.13 | 10.58 | 10.84 | 0.26 | 7.55 | 8.19 | 8.23 | 7.79 | 8.17 | 0.30 |
| GGH | 8836 | 7.31 | 7.69 | 8.18 | 6.98 | 7.63 | 0.45 | 4.94 | 5.29 | 5.30 | 4.36 | 5.41 | 0.43 |
| CAND1 | 55832 | 6.67 | 6.56 | 6.80 | 7.03 | 6.61 | 0.19 | 4.45 | 3.25 | 4.54 | 4.77 | 4.17 | 0.59 |
| EEF1A1 | 1915 | 12.80 | 13.17 | 12.85 | 12.60 | 12.99 | 0.21 | 10.20 | 10.20 | 10.97 | 10.06 | 10.49 | 0.37 |
| ATP2A2 | 488 | 7.49 | 6.45 | 7.30 | 7.94 | 7.32 | 0.54 | 5.14 | 4.62 | 4.93 | 4.63 | 4.72 | 0.22 |
| TRIOBP | 11078 | 9.95 | 9.04 | 9.86 | 8.75 | 9.21 | 0.52 | 6.95 | 6.73 | 7.43 | 6.62 | 6.70 | 0.32 |
| SLC20A1 | 6574 | 8.91 | 8.48 | 8.51 | 8.82 | 8.68 | 0.19 | 6.18 | 6.38 | 6.49 | 6.08 | 5.93 | 0.22 |
| TMSB10 | 9168 | 13.35 | 13.61 | 13.43 | 13.35 | 13.30 | 0.12 | 11.02 | 10.52 | 11.19 | 10.83 | 11.23 | 0.29 |
| SPATA2L | 124044 | 4.79 | 5.09 | 5.67 | 6.29 | 4.95 | 0.62 | 2.77 | 2.46 | 2.72 | 3.57 | 3.02 | 0.42 |
| PIGO | 84720 | 5.07 | 4.80 | 4.89 | 4.67 | 4.55 | 0.20 | 2.56 | 2.00 | 2.81 | 1.63 | 2.74 | 0.51 |
| ARMET | 7873 | 10.31 | 10.52 | 9.98 | 10.18 | 10.41 | 0.21 | 7.48 | 7.83 | 7.71 | 7.99 | 8.16 | 0.26 |
| OPRS1 | 10280 | 5.62 | 5.04 | 5.03 | 5.12 | 5.88 | 0.39 | 3.85 | 2.46 | 2.77 | 2.26 | 3.19 | 0.63 |
| ZYX | 7791 | 9.84 | 9.73 | 9.89 | 10.12 | 9.85 | 0.14 | 7.53 | 7.21 | 7.61 | 7.51 | 7.39 | 0.16 |
| NAT14 | 57106 | 6.38 | 7.07 | 7.89 | 5.15 | 6.83 | 1.01 | 3.78 | 4.18 | 5.08 | 3.32 | 4.81 | 0.72 |
| TRIM22 | 10346 | 7.13 | 6.65 | 6.93 | 6.45 | 6.77 | 0.26 | 4.82 | 3.85 | 4.90 | 3.39 | 4.89 | 0.70 |
| TMED9 | 54732 | 9.58 | 9.82 | 9.38 | 9.55 | 9.79 | 0.18 | 7.14 | 7.08 | 7.23 | 7.30 | 7.28 | 0.09 |
| RHOG | 391 | 8.86 | 7.95 | 8.72 | 9.30 | 8.79 | 0.49 | 6.60 | 6.05 | 6.36 | 6.36 | 6.18 | 0.21 |
| C18orf10 | 25941 | 7.06 | 7.02 | 6.84 | 5.90 | 7.22 | 0.53 | 4.44 | 4.50 | 4.83 | 3.57 | 4.65 | 0.49 |
| MMP23A | 8511 | 5.94 | 3.88 | 4.24 | 4.05 | 6.24 | 1.13 | 2.98 | 1.14 | 2.00 | 2.35 | 3.90 | 1.04 |
| GPNMB | 10457 | 10.98 | 10.42 | 11.31 | 10.72 | 9.81 | 0.57 | 8.34 | 7.68 | 9.07 | 7.92 | 8.28 | 0.53 |
| ARMCX2 | 9823 | 9.14 | 9.55 | 9.32 | 8.73 | 8.97 | 0.32 | 6.47 | 7.07 | 6.42 | 6.68 | 7.14 | 0.34 |
| NRP1 | 8829 | 6.59 | 5.59 | 7.01 | 5.63 | 6.25 | 0.61 | 4.26 | 4.01 | 4.15 | 2.77 | 3.95 | 0.61 |
| GRN | 2896 | 9.02 | 8.97 | 9.46 | 8.33 | 8.78 | 0.41 | 6.52 | 6.28 | 6.59 | 6.67 | 6.61 | 0.15 |
| FYN | 2534 | 8.02 | 7.50 | 7.89 | 8.15 | 7.51 | 0.30 | 5.30 | 4.74 | 5.91 | 5.71 | 5.56 | 0.45 |
| RPL23A | 6147 | 8.51 | 8.69 | 8.53 | 8.02 | 8.37 | 0.25 | 6.25 | 6.27 | 6.11 | 5.55 | 6.07 | 0.29 |
| HYOU1 | 10525 | 6.20 | 6.07 | 4.95 | 5.33 | 6.31 | 0.60 | 4.14 | 2.96 | 2.81 | 2.63 | 4.50 | 0.85 |
| COL6A2 | 1292 | 8.80 | 7.71 | 9.21 | 8.26 | 8.14 | 0.59 | 6.11 | 5.46 | 6.86 | 5.91 | 5.98 | 0.51 |
| TMEM98 | 26022 | 9.08 | 8.57 | 9.19 | 8.76 | 9.09 | 0.26 | 6.63 | 6.24 | 6.48 | 6.44 | 7.10 | 0.32 |
| NME7 | 29922 | 7.06 | 7.15 | 7.31 | 6.90 | 7.23 | 0.16 | 4.80 | 4.52 | 4.32 | 5.17 | 5.05 | 0.35 |
| COL3A1 | 1281 | 11.88 | 11.22 | 11.40 | 10.46 | 11.42 | 0.52 | 9.56 | 8.72 | 8.40 | 8.91 | 8.99 | 0.43 |
| CDC42EP1 | 11135 | 6.23 | 6.75 | 6.36 | 6.57 | 6.81 | 0.25 | 4.15 | 3.77 | 3.74 | 4.94 | 4.33 | 0.49 |
| FSTL1 | 11167 | 12.31 | 12.16 | 11.87 | 11.98 | 12.37 | 0.21 | 9.61 | 9.54 | 10.44 | 9.36 | 10.01 | 0.43 |
| SLC30A7 | 148867 | 8.74 | 8.57 | 8.64 | 9.07 | 8.80 | 0.19 | 6.32 | 6.30 | 6.33 | 6.62 | 6.53 | 0.14 |
| SHC1 | 6464 | 10.33 | 9.72 | 10.09 | 9.82 | 10.01 | 0.24 | 7.61 | 7.63 | 7.75 | 7.64 | 7.66 | 0.05 |
| C6orf48 | 50854 | 7.07 | 7.28 | 6.68 | 6.83 | 7.64 | 0.38 | 4.54 | 4.52 | 4.74 | 5.11 | 4.93 | 0.25 |
| CCDC109B | 55013 | 8.91 | 8.71 | 9.08 | 8.77 | 8.80 | 0.15 | 6.07 | 6.20 | 6.94 | 6.38 | 7.04 | 0.44 |
| MGC33212 | 255758 | 8.05 | 8.11 | 8.28 | 7.59 | 8.09 | 0.26 | 5.89 | 5.94 | 5.92 | 5.13 | 5.60 | 0.35 |
| SUMF1 | 285362 | 8.23 | 7.47 | 8.53 | 7.40 | 7.80 | 0.49 | 5.59 | 5.69 | 5.57 | 5.22 | 5.74 | 0.20 |
| ARF4 | 378 | 11.01 | 10.39 | 10.39 | 10.95 | 11.05 | 0.34 | 8.25 | 8.39 | 8.64 | 8.33 | 8.60 | 0.17 |
| SPIN4 | 139886 | 5.24 | 5.76 | 5.74 | 4.89 | 5.14 | 0.38 | 2.91 | 3.99 | 2.77 | 2.70 | 2.83 | 0.54 |
| STX3 | 6809 | 5.47 | 5.43 | 5.66 | 6.92 | 5.46 | 0.64 | 2.91 | 3.83 | 3.02 | 4.35 | 3.28 | 0.60 |
| TRIP6 | 7205 | 9.24 | 8.21 | 9.43 | 9.10 | 8.80 | 0.47 | 6.68 | 6.67 | 6.85 | 6.44 | 6.61 | 0.15 |
| EEF1A1 | 1915 | 12.71 | 12.43 | 12.61 | 12.45 | 12.84 | 0.17 | 10.00 | 10.12 | 10.84 | 10.05 | 10.52 | 0.36 |
| HSPA5 | 3309 | 8.46 | 8.54 | 8.03 | 8.15 | 8.57 | 0.25 | 5.80 | 5.87 | 5.85 | 6.36 | 6.40 | 0.30 |
| EML4 | 27436 | 7.69 | 7.72 | 7.66 | 7.38 | 7.37 | 0.18 | 5.12 | 4.94 | 5.31 | 5.82 | 5.18 | 0.33 |
| SSR4 | 6748 | 11.21 | 10.98 | 10.97 | 10.84 | 11.27 | 0.18 | 8.59 | 8.91 | 8.77 | 8.66 | 8.89 | 0.14 |
| POFUT2 | 23275 | 8.25 | 8.46 | 8.42 | 8.88 | 8.50 | 0.23 | 6.39 | 6.36 | 6.06 | 5.97 | 6.34 | 0.19 |
| PYGB | 5834 | 8.26 | 7.79 | 8.26 | 7.30 | 7.93 | 0.40 | 5.82 | 4.97 | 6.26 | 5.54 | 5.57 | 0.47 |
| PEA15 | 8682 | 10.91 | 11.34 | 10.31 | 11.26 | 11.21 | 0.42 | 8.31 | 8.88 | 8.61 | 8.61 | 9.26 | 0.36 |
| SPA17 | 53340 | 6.88 | 6.16 | 6.98 | 6.16 | 7.04 | 0.45 | 4.52 | 3.95 | 5.33 | 3.20 | 4.87 | 0.83 |
| C1R | 715 | 8.13 | 7.25 | 8.81 | 7.57 | 7.20 | 0.68 | 5.40 | 5.10 | 6.54 | 4.96 | 5.64 | 0.63 |
| PRELID1 | 27166 | 7.93 | 7.93 | 7.45 | 8.16 | 8.15 | 0.29 | 5.58 | 5.39 | 6.09 | 5.55 | 5.73 | 0.27 |
| XPR1 | 9213 | 7.15 | 7.23 | 7.46 | 7.37 | 7.28 | 0.12 | 4.98 | 4.74 | 5.60 | 4.60 | 5.30 | 0.41 |
| TGIF1 | 7050 | 7.38 | 7.30 | 7.10 | 7.80 | 7.29 | 0.26 | 4.72 | 5.18 | 5.30 | 5.47 | 4.93 | 0.30 |
| ABHD12 | 26090 | 7.23 | 5.93 | 6.80 | 6.48 | 5.55 | 0.67 | 5.55 | 3.36 | 4.77 | 3.54 | 3.54 | 0.96 |
| LAMB1 | 3912 | 8.89 | 8.46 | 9.27 | 7.99 | 8.49 | 0.48 | 6.68 | 5.88 | 6.43 | 6.03 | 6.87 | 0.42 |
| RRAS | 6237 | 10.14 | 9.64 | 10.44 | 9.37 | 10.44 | 0.48 | 7.74 | 7.78 | 8.06 | 7.49 | 7.75 | 0.21 |
| LTBR | 4055 | 8.97 | 8.10 | 8.84 | 8.64 | 8.28 | 0.37 | 6.37 | 5.55 | 7.20 | 6.12 | 6.39 | 0.59 |
| CD63 | 967 | 10.53 | 9.70 | 10.75 | 9.91 | 10.33 | 0.43 | 8.27 | 7.99 | 8.45 | 7.89 | 7.46 | 0.38 |
| TMEM138 | 51524 | 8.18 | 7.48 | 7.84 | 8.37 | 8.05 | 0.34 | 5.82 | 5.68 | 5.87 | 5.56 | 5.84 | 0.13 |
| ARHGEF2 | 9181 | 10.81 | 10.76 | 10.33 | 10.54 | 10.94 | 0.24 | 7.98 | 8.29 | 8.65 | 8.72 | 8.59 | 0.31 |
| CAPG | 822 | 6.07 | 5.14 | 6.27 | 6.02 | 5.83 | 0.44 | 3.82 | 3.60 | 3.97 | 3.32 | 3.47 | 0.26 |
| LOC57228 | 57228 | 6.57 | 5.80 | 7.00 | 6.84 | 6.18 | 0.49 | 4.60 | 3.41 | 4.74 | 4.04 | 4.45 | 0.54 |
| HEXB | 3074 | 11.14 | 10.16 | 10.91 | 10.87 | 10.61 | 0.37 | 8.49 | 8.18 | 8.98 | 8.41 | 8.50 | 0.29 |
| DKFZp762E1312 | 55355 | 4.79 | 3.83 | 4.46 | 3.38 | 4.04 | 0.55 | 2.77 | 1.68 | 2.10 | 1.07 | 1.81 | 0.62 |
| NPC2 | 10577 | 11.27 | 10.96 | 11.61 | 11.26 | 11.06 | 0.25 | 8.81 | 9.07 | 9.42 | 8.74 | 9.06 | 0.27 |
| TSHZ3 | 57616 | 7.47 | 7.42 | 7.48 | 7.92 | 7.06 | 0.31 | 5.24 | 5.13 | 5.12 | 5.41 | 5.50 | 0.17 |
| CHIC2 | 26511 | 8.70 | 8.77 | 8.15 | 9.47 | 9.01 | 0.48 | 6.71 | 6.76 | 6.35 | 6.44 | 6.90 | 0.23 |
| TPST1 | 8460 | 8.06 | 7.16 | 7.36 | 7.08 | 7.89 | 0.44 | 5.86 | 5.06 | 5.29 | 4.85 | 5.58 | 0.40 |
| SEC61A1 | 29927 | 8.32 | 7.60 | 7.90 | 7.64 | 8.10 | 0.30 | 5.57 | 5.51 | 5.80 | 5.83 | 5.95 | 0.19 |
| PGM3 | 5238 | 7.08 | 6.82 | 6.11 | 7.43 | 7.41 | 0.54 | 4.77 | 4.72 | 4.75 | 4.86 | 4.91 | 0.08 |
| MIF | 4282 | 10.51 | 11.12 | 10.64 | 10.23 | 11.01 | 0.37 | 8.50 | 8.49 | 8.56 | 8.62 | 8.50 | 0.06 |
| LMNA | 4000 | 9.32 | 9.02 | 9.63 | 9.04 | 8.84 | 0.31 | 7.03 | 6.48 | 7.50 | 7.12 | 6.89 | 0.37 |
| LARP6 | 55323 | 8.80 | 8.83 | 8.46 | 8.25 | 8.72 | 0.25 | 6.38 | 6.34 | 6.51 | 6.21 | 6.78 | 0.22 |
| SLC35B3 | 51000 | 7.35 | 7.00 | 6.78 | 6.75 | 7.15 | 0.25 | 4.99 | 4.87 | 4.37 | 4.85 | 5.13 | 0.28 |
| ZDHHC24 | 254359 | 5.37 | 4.89 | 4.93 | 4.85 | 5.39 | 0.27 | 3.19 | 3.43 | 2.70 | 2.70 | 2.58 | 0.37 |
| ATP6AP2 | 10159 | 10.70 | 10.23 | 10.44 | 10.38 | 10.38 | 0.17 | 8.04 | 8.38 | 8.46 | 8.02 | 8.40 | 0.21 |
| P4HB | 5034 | 11.47 | 11.10 | 11.27 | 10.94 | 11.38 | 0.21 | 8.97 | 9.11 | 9.06 | 9.16 | 9.06 | 0.07 |
| ZNF668 | 79759 | 6.91 | 7.52 | 6.45 | 7.72 | 7.07 | 0.50 | 4.97 | 5.13 | 4.77 | 4.84 | 5.17 | 0.17 |
| EIF4A1 | 1973 | 11.27 | 11.04 | 11.07 | 11.71 | 11.12 | 0.28 | 9.06 | 8.81 | 9.54 | 8.95 | 9.10 | 0.28 |
| SCPEP1 | 59342 | 9.25 | 8.14 | 9.10 | 8.54 | 8.59 | 0.45 | 6.48 | 6.36 | 6.76 | 6.68 | 6.58 | 0.16 |
| STT3A | 3703 | 8.88 | 8.72 | 8.83 | 8.10 | 9.02 | 0.36 | 6.41 | 6.26 | 7.01 | 6.41 | 6.72 | 0.30 |
| RCC2 | 55920 | 9.44 | 8.21 | 8.54 | 8.83 | 8.87 | 0.46 | 6.80 | 6.22 | 6.73 | 6.78 | 6.63 | 0.24 |
| GTF2E2 | 2961 | 8.88 | 8.64 | 8.61 | 9.19 | 9.05 | 0.26 | 6.55 | 6.73 | 6.80 | 6.66 | 6.95 | 0.15 |
| NAV1 | 89796 | 6.86 | 7.13 | 7.22 | 7.37 | 7.22 | 0.19 | 4.88 | 4.39 | 5.47 | 5.19 | 5.19 | 0.41 |
| ITGAV | 3685 | 9.39 | 9.43 | 8.86 | 8.51 | 9.33 | 0.40 | 6.73 | 6.99 | 6.97 | 6.82 | 7.35 | 0.24 |
| HSP90B1 | 7184 | 10.96 | 11.13 | 10.61 | 10.73 | 10.93 | 0.20 | 8.93 | 8.51 | 8.90 | 8.72 | 8.74 | 0.17 |
| XPO6 | 23214 | 7.71 | 7.42 | 7.52 | 8.29 | 7.59 | 0.34 | 5.52 | 5.42 | 5.79 | 5.71 | 5.55 | 0.15 |
| CMTM6 | 54918 | 7.72 | 7.34 | 7.55 | 7.27 | 7.33 | 0.19 | 4.95 | 5.57 | 5.74 | 5.29 | 5.13 | 0.32 |
| UNC119 | 9094 | 5.61 | 6.11 | 5.97 | 5.40 | 6.10 | 0.32 | 3.28 | 3.99 | 3.96 | 3.32 | 4.12 | 0.40 |
| CD99 | 4267 | 11.33 | 11.77 | 11.73 | 10.95 | 11.24 | 0.35 | 9.27 | 9.31 | 9.46 | 8.76 | 9.73 | 0.36 |
| FAM46A | 55603 | 10.00 | 9.56 | 10.29 | 9.77 | 9.82 | 0.27 | 7.71 | 7.98 | 7.67 | 7.70 | 7.91 | 0.14 |
| LOC731049 | 731049 | 8.13 | 7.94 | 7.92 | 8.44 | 8.30 | 0.23 | 5.97 | 5.41 | 6.24 | 6.65 | 5.99 | 0.45 |
| CFL1 | 1072 | 11.38 | 11.19 | 11.12 | 11.14 | 11.33 | 0.12 | 9.03 | 9.07 | 9.27 | 9.12 | 9.25 | 0.11 |
| BNIP3L | 665 | 8.05 | 8.04 | 8.23 | 7.81 | 8.11 | 0.15 | 5.81 | 6.05 | 6.57 | 5.56 | 5.83 | 0.38 |
| RHOC | 389 | 9.45 | 9.25 | 9.26 | 9.75 | 9.71 | 0.24 | 7.22 | 6.89 | 7.27 | 7.69 | 7.92 | 0.41 |
| RPL3 | 6122 | 12.39 | 12.06 | 12.14 | 11.94 | 12.23 | 0.17 | 10.14 | 10.16 | 10.63 | 9.95 | 9.48 | 0.41 |
| SSR1 | 6745 | 9.43 | 8.66 | 8.94 | 9.07 | 9.15 | 0.28 | 6.92 | 7.10 | 7.25 | 6.51 | 7.09 | 0.28 |
| ATP1B3 | 483 | 6.28 | 6.51 | 5.81 | 6.22 | 6.59 | 0.31 | 3.55 | 4.74 | 4.16 | 3.93 | 4.66 | 0.50 |
| SYDE1 | 85360 | 7.36 | 6.77 | 7.28 | 7.67 | 7.35 | 0.32 | 5.36 | 4.85 | 4.90 | 5.34 | 5.60 | 0.32 |
| HMGCR | 3156 | 7.91 | 7.51 | 6.46 | 6.98 | 7.37 | 0.55 | 5.42 | 5.63 | 4.76 | 5.09 | 4.99 | 0.35 |
| GBP1 | 2633 | 5.05 | 5.25 | 4.93 | 4.32 | 5.02 | 0.35 | 2.41 | 3.22 | 3.12 | 2.58 | 2.93 | 0.35 |
| MAGED1 | 9500 | 11.51 | 11.69 | 11.46 | 10.64 | 11.45 | 0.41 | 9.23 | 9.71 | 9.13 | 9.07 | 9.31 | 0.26 |
| SGCE | 8910 | 9.40 | 8.04 | 9.74 | 9.04 | 9.07 | 0.63 | 7.10 | 6.54 | 7.07 | 6.90 | 7.36 | 0.30 |
| SDF4 | 51150 | 9.93 | 9.09 | 9.77 | 9.33 | 9.48 | 0.34 | 7.44 | 7.36 | 7.74 | 7.38 | 7.40 | 0.16 |
| DSE | 29940 | 8.97 | 8.78 | 8.63 | 8.22 | 8.83 | 0.28 | 6.91 | 6.49 | 7.01 | 6.07 | 6.68 | 0.37 |
| RAB23 | 51715 | 6.94 | 7.14 | 6.37 | 5.98 | 6.99 | 0.49 | 4.78 | 4.38 | 4.70 | 4.37 | 4.94 | 0.25 |
| AXL | 558 | 8.62 | 8.73 | 9.28 | 8.91 | 8.67 | 0.27 | 6.67 | 6.51 | 7.11 | 6.63 | 7.05 | 0.27 |
| CASP3 | 836 | 8.23 | 9.27 | 7.94 | 8.93 | 8.51 | 0.53 | 6.32 | 6.93 | 6.28 | 6.61 | 6.54 | 0.26 |
| LDOC1 | 23641 | 8.13 | 7.35 | 8.91 | 7.38 | 7.98 | 0.64 | 5.61 | 6.06 | 6.74 | 5.21 | 5.92 | 0.57 |
| CNN2 | 1265 | 3.19 | 2.32 | 2.91 | 2.00 | 3.55 | 0.63 | 0.58 | 0.00 | 1.14 | 0.00 | 2.04 | 0.86 |
| ARPC5 | 10092 | 10.33 | 9.97 | 9.91 | 10.35 | 9.98 | 0.21 | 7.85 | 7.96 | 8.29 | 8.01 | 8.22 | 0.18 |
| LASS5 | 91012 | 7.67 | 7.45 | 7.67 | 7.67 | 7.87 | 0.15 | 5.34 | 5.72 | 5.73 | 5.82 | 5.56 | 0.19 |
| CD151 | 977 | 6.19 | 5.90 | 6.97 | 6.11 | 6.61 | 0.43 | 4.22 | 3.29 | 5.27 | 4.36 | 4.49 | 0.71 |
| LASP1 | 3927 | 10.24 | 9.52 | 10.04 | 10.07 | 9.93 | 0.27 | 8.03 | 7.57 | 8.11 | 7.95 | 8.01 | 0.21 |
| NUCB2 | 4925 | 9.82 | 9.39 | 9.68 | 9.15 | 9.63 | 0.26 | 7.62 | 7.50 | 7.47 | 7.19 | 7.76 | 0.21 |
| IFITM3 | 10410 | 11.78 | 11.62 | 12.45 | 11.82 | 11.76 | 0.32 | 9.77 | 9.44 | 10.50 | 9.81 | 9.81 | 0.39 |
| RAB8A | 4218 | 8.33 | 7.76 | 8.05 | 7.96 | 8.01 | 0.20 | 6.19 | 5.78 | 6.29 | 5.90 | 5.85 | 0.22 |
| SLC25A24 | 29957 | 2.26 | 3.26 | 1.81 | 1.43 | 2.79 | 0.73 | 0.00 | 0.77 | 0.00 | 0.00 | 0.77 | 0.42 |
| C14orf45 | 80127 | 5.52 | 5.28 | 5.62 | 5.22 | 5.77 | 0.23 | 3.80 | 3.23 | 3.57 | 2.58 | 4.21 | 0.61 |
| LAMC1 | 3915 | 11.62 | 11.20 | 11.09 | 11.42 | 11.00 | 0.25 | 9.26 | 8.93 | 9.33 | 9.36 | 9.42 | 0.19 |
| SHMT2 | 6472 | 10.43 | 10.06 | 9.77 | 9.50 | 10.29 | 0.38 | 8.10 | 8.19 | 7.79 | 8.02 | 7.95 | 0.15 |
| CLPTM1L | 81037 | 8.64 | 7.97 | 8.29 | 8.22 | 8.51 | 0.26 | 6.47 | 5.79 | 6.59 | 6.43 | 6.36 | 0.31 |
| TMEM4 | 10330 | 9.81 | 9.32 | 9.50 | 9.16 | 9.67 | 0.26 | 7.52 | 7.39 | 7.58 | 7.40 | 7.63 | 0.11 |
| DEGS1 | 8560 | 7.64 | 7.84 | 7.45 | 8.09 | 7.71 | 0.24 | 5.84 | 5.34 | 5.97 | 5.69 | 5.94 | 0.26 |
| C12orf23 | 90488 | 7.39 | 7.81 | 7.00 | 7.58 | 7.43 | 0.30 | 5.93 | 5.21 | 5.06 | 5.53 | 5.55 | 0.34 |
| JARID1B | 10765 | 7.72 | 8.10 | 7.92 | 8.46 | 7.96 | 0.27 | 6.26 | 6.22 | 5.66 | 5.90 | 6.19 | 0.26 |
| C19orf48 | 84798 | 7.99 | 7.28 | 7.41 | 7.78 | 7.62 | 0.28 | 5.67 | 5.89 | 5.42 | 5.59 | 5.74 | 0.17 |
| SDF2L1 | 23753 | 8.45 | 8.50 | 8.16 | 8.01 | 8.69 | 0.27 | 6.26 | 6.19 | 6.34 | 6.54 | 6.72 | 0.22 |
| COX6A1 | 1337 | 9.46 | 9.91 | 9.37 | 9.25 | 9.80 | 0.29 | 7.45 | 7.91 | 7.96 | 7.24 | 7.49 | 0.31 |
| LRP10 | 26020 | 10.08 | 9.50 | 10.17 | 9.50 | 9.77 | 0.31 | 7.64 | 7.58 | 7.98 | 7.93 | 8.15 | 0.24 |
| SGPP1 | 81537 | 8.43 | 8.86 | 8.36 | 9.08 | 8.46 | 0.32 | 6.59 | 6.84 | 6.61 | 6.55 | 6.86 | 0.15 |
| MGC40499 | 245812 | 8.24 | 7.36 | 8.55 | 7.64 | 7.85 | 0.47 | 5.89 | 5.62 | 6.23 | 5.85 | 6.34 | 0.30 |
| LMNA | 4000 | 9.96 | 9.36 | 10.58 | 9.68 | 9.53 | 0.48 | 7.57 | 7.72 | 8.32 | 7.93 | 7.90 | 0.28 |
| CEP290 | 80184 | 4.49 | 4.57 | 4.72 | 3.80 | 4.66 | 0.37 | 2.58 | 1.89 | 2.91 | 2.07 | 3.10 | 0.52 |
| ARMCX1 | 51309 | 6.46 | 6.83 | 7.13 | 6.75 | 6.75 | 0.24 | 4.69 | 4.20 | 5.49 | 4.99 | 4.88 | 0.47 |
| FBXO5 | 26271 | 5.77 | 5.10 | 4.80 | 5.14 | 4.72 | 0.41 | 3.49 | 3.42 | 2.43 | 3.25 | 3.29 | 0.43 |
| ALG8 | 79053 | 7.57 | 7.39 | 7.25 | 6.96 | 7.56 | 0.25 | 5.42 | 5.19 | 5.25 | 5.39 | 5.82 | 0.25 |
| SLC38A6 | 145389 | 7.23 | 6.90 | 6.96 | 6.53 | 7.02 | 0.25 | 4.71 | 5.30 | 5.44 | 4.61 | 4.96 | 0.36 |
| RNF135 | 84282 | 6.76 | 6.36 | 6.61 | 6.62 | 6.71 | 0.15 | 5.31 | 4.50 | 4.67 | 4.70 | 4.28 | 0.39 |
| KLHDC5 | 57542 | 7.64 | 7.36 | 7.20 | 7.83 | 7.44 | 0.24 | 5.42 | 5.18 | 5.53 | 5.91 | 5.83 | 0.30 |
| SLC39A11 | 201266 | 6.19 | 5.98 | 6.47 | 5.90 | 5.80 | 0.27 | 4.52 | 3.35 | 4.64 | 4.39 | 3.86 | 0.54 |
| GMFB | 2764 | 8.83 | 8.96 | 8.75 | 9.02 | 8.74 | 0.13 | 6.97 | 7.21 | 6.54 | 6.74 | 7.26 | 0.31 |
| MAN2B2 | 23324 | 9.71 | 9.04 | 10.13 | 9.22 | 9.24 | 0.45 | 7.34 | 7.17 | 7.94 | 7.53 | 7.84 | 0.33 |
| TETRAN | 10227 | 9.25 | 9.07 | 9.57 | 8.82 | 9.27 | 0.28 | 7.26 | 7.02 | 7.47 | 7.21 | 7.48 | 0.19 |
| RABAC1 | 10567 | 10.55 | 9.89 | 10.61 | 9.86 | 10.78 | 0.43 | 8.18 | 8.22 | 8.80 | 8.44 | 8.56 | 0.26 |
| PRKCSH | 5589 | 8.92 | 8.74 | 8.84 | 8.13 | 8.86 | 0.32 | 6.84 | 6.65 | 7.01 | 6.70 | 6.81 | 0.14 |
| ZC3HAV1 | 56829 | 8.24 | 7.65 | 8.20 | 7.28 | 8.06 | 0.41 | 5.95 | 6.07 | 6.29 | 5.49 | 6.18 | 0.31 |
| DDOST | 1650 | 10.11 | 9.49 | 10.37 | 9.51 | 10.01 | 0.38 | 7.93 | 7.98 | 8.20 | 7.99 | 7.99 | 0.11 |
| MED19 | 219541 | 7.37 | 6.81 | 6.78 | 7.49 | 7.25 | 0.32 | 5.42 | 5.42 | 4.74 | 5.40 | 5.31 | 0.29 |
| PLXNA1 | 5361 | 7.77 | 7.78 | 7.40 | 7.89 | 7.82 | 0.19 | 6.13 | 6.28 | 5.21 | 5.70 | 5.94 | 0.42 |
| TULP3 | 7289 | 6.20 | 5.35 | 6.19 | 5.75 | 6.11 | 0.37 | 4.43 | 3.36 | 4.43 | 4.22 | 3.78 | 0.46 |
| ELOVL1 | 64834 | 6.35 | 5.68 | 5.82 | 6.43 | 6.20 | 0.33 | 3.85 | 4.23 | 4.23 | 4.46 | 4.32 | 0.23 |
| REXO2 | 25996 | 11.24 | 11.08 | 10.86 | 10.83 | 11.15 | 0.18 | 8.92 | 9.14 | 9.00 | 9.28 | 9.44 | 0.21 |
| SFRS9 | 8683 | 10.25 | 10.16 | 10.05 | 10.61 | 10.23 | 0.21 | 8.29 | 8.39 | 8.65 | 8.29 | 8.33 | 0.15 |
| TCEAL3 | 85012 | 7.80 | 7.31 | 8.01 | 7.05 | 7.86 | 0.41 | 5.76 | 5.56 | 5.78 | 5.49 | 6.10 | 0.24 |
| YARS | 8565 | 9.97 | 9.80 | 9.56 | 9.56 | 9.86 | 0.19 | 7.92 | 7.87 | 7.69 | 8.04 | 7.98 | 0.13 |
| LOC727761 | 727761 | 6.22 | 5.96 | 6.20 | 5.53 | 6.24 | 0.30 | 4.24 | 3.94 | 4.82 | 3.70 | 4.23 | 0.42 |
| EXT2 | 2132 | 8.37 | 7.83 | 7.67 | 8.19 | 8.18 | 0.29 | 6.14 | 6.07 | 6.11 | 6.13 | 6.59 | 0.22 |
| EEF1A1 | 1915 | 13.17 | 13.35 | 13.35 | 13.17 | 13.22 | 0.09 | 11.34 | 11.24 | 11.82 | 11.14 | 11.57 | 0.28 |
| SERF2 | 10169 | 11.42 | 11.45 | 11.77 | 10.90 | 11.70 | 0.34 | 9.85 | 9.36 | 9.59 | 9.62 | 9.68 | 0.18 |
| SLC17A5 | 26503 | 8.70 | 8.26 | 8.78 | 8.16 | 8.35 | 0.27 | 6.34 | 6.54 | 6.88 | 6.33 | 7.01 | 0.31 |
| IL4R | 3566 | 7.51 | 7.27 | 8.12 | 8.25 | 7.12 | 0.51 | 5.87 | 5.39 | 6.10 | 6.07 | 5.73 | 0.29 |
| MVD | 4597 | 5.31 | 5.33 | 4.90 | 4.14 | 5.54 | 0.56 | 3.62 | 4.04 | 2.91 | 1.77 | 3.79 | 0.92 |
| PDPN | 10630 | 8.55 | 7.74 | 8.03 | 8.34 | 8.41 | 0.33 | 6.93 | 6.25 | 6.51 | 5.95 | 6.39 | 0.36 |
| NAGK | 55577 | 9.07 | 9.04 | 8.87 | 9.33 | 8.98 | 0.17 | 7.33 | 7.31 | 7.28 | 7.35 | 6.99 | 0.15 |
| MFSD1 | 64747 | 9.33 | 8.78 | 9.40 | 8.90 | 9.23 | 0.27 | 7.10 | 7.11 | 7.28 | 7.47 | 7.67 | 0.24 |
| SURF4 | 6836 | 9.33 | 9.04 | 9.25 | 9.59 | 9.31 | 0.20 | 7.79 | 7.49 | 7.51 | 7.39 | 7.35 | 0.17 |
| MPZL1 | 9019 | 8.68 | 7.93 | 8.83 | 8.13 | 8.55 | 0.38 | 6.50 | 6.21 | 7.29 | 6.31 | 6.84 | 0.44 |
| OSBPL8 | 114882 | 9.00 | 8.12 | 7.95 | 8.40 | 8.42 | 0.40 | 6.65 | 6.82 | 6.25 | 6.41 | 6.80 | 0.25 |
| DEGS1 | 8560 | 10.29 | 10.12 | 10.14 | 10.67 | 10.02 | 0.26 | 8.41 | 8.21 | 8.57 | 8.36 | 8.78 | 0.22 |
| RPS27L | 51065 | 11.82 | 11.97 | 11.94 | 11.46 | 12.03 | 0.23 | 10.06 | 10.43 | 10.01 | 9.89 | 9.95 | 0.21 |
| UBE2E2 | 7325 | 8.99 | 9.02 | 9.00 | 8.57 | 9.21 | 0.24 | 6.97 | 7.36 | 7.33 | 7.01 | 7.27 | 0.18 |
| ITGB1 | 3688 | 12.32 | 12.23 | 12.40 | 12.15 | 12.19 | 0.10 | 10.52 | 10.44 | 10.42 | 10.50 | 10.58 | 0.06 |
| TRAM1 | 23471 | 10.89 | 10.55 | 10.38 | 10.59 | 10.86 | 0.22 | 8.80 | 8.94 | 8.75 | 8.81 | 9.16 | 0.17 |
| IFITM2 | 10581 | 11.96 | 11.51 | 12.53 | 11.83 | 11.73 | 0.38 | 10.11 | 9.85 | 10.69 | 10.12 | 10.01 | 0.32 |
| POLR3H | 171568 | 7.19 | 7.07 | 6.74 | 7.01 | 7.25 | 0.20 | 5.51 | 5.59 | 4.56 | 5.31 | 5.53 | 0.43 |
| CLTA | 1211 | 8.39 | 8.27 | 8.36 | 8.70 | 8.49 | 0.17 | 6.96 | 6.58 | 6.95 | 6.38 | 6.58 | 0.26 |
| C12orf24 | 29902 | 7.62 | 8.19 | 7.44 | 7.82 | 7.71 | 0.28 | 5.53 | 6.07 | 5.63 | 6.25 | 6.53 | 0.42 |
| CNIH | 10175 | 7.21 | 7.34 | 7.13 | 7.16 | 7.57 | 0.18 | 5.41 | 5.50 | 5.49 | 5.58 | 5.74 | 0.13 |
| M6PRBP1 | 10226 | 10.94 | 11.55 | 11.28 | 10.63 | 10.82 | 0.37 | 9.38 | 9.69 | 9.36 | 9.16 | 8.98 | 0.27 |
| DBN1 | 1627 | 9.03 | 9.29 | 8.82 | 8.98 | 9.22 | 0.19 | 6.95 | 7.13 | 7.54 | 7.31 | 7.76 | 0.32 |
| ACTB | 60 | 13.81 | 13.54 | 13.89 | 14.03 | 13.89 | 0.18 | 12.08 | 11.83 | 12.31 | 12.09 | 12.21 | 0.18 |
| ERLIN1 | 10613 | 5.64 | 5.27 | 5.32 | 5.41 | 5.36 | 0.15 | 4.26 | 3.31 | 3.22 | 3.51 | 4.10 | 0.47 |
| PRTFDC1 | 56952 | 5.95 | 4.71 | 6.43 | 5.04 | 4.64 | 0.80 | 4.26 | 2.85 | 4.41 | 3.79 | 2.87 | 0.75 |
| DNCL1 | 8655 | 11.39 | 11.66 | 11.52 | 11.41 | 11.61 | 0.12 | 9.75 | 9.65 | 10.00 | 9.67 | 9.93 | 0.16 |
| MAP4K5 | 11183 | 7.83 | 7.75 | 7.63 | 8.43 | 7.78 | 0.31 | 6.47 | 5.82 | 5.86 | 6.33 | 6.36 | 0.31 |
| TBPL1 | 9519 | 8.13 | 8.27 | 8.00 | 8.63 | 8.18 | 0.24 | 6.48 | 6.82 | 6.50 | 6.31 | 6.53 | 0.18 |
| NLGN2 | 57555 | 6.59 | 6.31 | 7.28 | 6.23 | 6.73 | 0.41 | 4.72 | 4.78 | 5.07 | 4.79 | 5.24 | 0.23 |
| LAMP1 | 3916 | 12.05 | 11.82 | 11.84 | 11.81 | 11.77 | 0.11 | 10.30 | 9.92 | 10.24 | 10.11 | 10.22 | 0.15 |
| TNFRSF1A | 7132 | 9.57 | 9.12 | 9.82 | 9.48 | 9.02 | 0.33 | 7.93 | 7.51 | 8.16 | 7.80 | 7.13 | 0.40 |
| LRRC32 | 2615 | 7.82 | 7.26 | 8.53 | 8.38 | 7.63 | 0.53 | 6.20 | 5.79 | 6.53 | 6.22 | 6.37 | 0.28 |
| SLC25A6 | 293 | 10.00 | 9.63 | 9.98 | 9.13 | 9.80 | 0.35 | 7.84 | 7.90 | 8.55 | 7.85 | 7.94 | 0.30 |
| SLC25A5 | 292 | 10.74 | 10.85 | 10.58 | 11.08 | 10.64 | 0.20 | 9.12 | 8.83 | 9.32 | 8.98 | 9.18 | 0.19 |
| DCTD | 1635 | 9.00 | 8.71 | 8.72 | 8.48 | 8.72 | 0.18 | 6.93 | 6.73 | 7.26 | 7.10 | 7.18 | 0.21 |
| ZNF329 | 79673 | 6.89 | 7.08 | 7.36 | 7.36 | 7.03 | 0.21 | 5.22 | 5.34 | 5.41 | 5.49 | 5.84 | 0.23 |
| OCIAD2 | 132299 | 6.56 | 7.74 | 7.83 | 7.81 | 7.69 | 0.54 | 5.30 | 6.09 | 6.07 | 6.22 | 5.54 | 0.40 |
| S100PBP | 64766 | 4.22 | 4.45 | 4.74 | 4.22 | 4.43 | 0.21 | 2.89 | 2.70 | 3.19 | 2.00 | 2.89 | 0.44 |
| FADD | 8772 | 7.70 | 7.71 | 7.49 | 8.01 | 7.91 | 0.20 | 6.24 | 6.17 | 5.89 | 6.23 | 5.91 | 0.18 |
| NSDHL | 50814 | 7.31 | 7.10 | 6.87 | 7.43 | 7.25 | 0.21 | 5.50 | 5.64 | 5.42 | 5.51 | 5.54 | 0.08 |
| MFGE8 | 4240 | 11.73 | 11.43 | 11.65 | 11.35 | 11.59 | 0.16 | 9.67 | 9.63 | 9.92 | 10.01 | 10.20 | 0.24 |
| SND1 | 27044 | 9.90 | 9.33 | 9.91 | 9.23 | 9.63 | 0.32 | 8.05 | 8.07 | 7.82 | 7.71 | 8.00 | 0.16 |
| PPP1R14B | 26472 | 11.26 | 11.84 | 11.30 | 11.27 | 11.40 | 0.25 | 9.82 | 9.57 | 9.59 | 9.77 | 10.02 | 0.18 |
| CD81 | 975 | 12.65 | 12.28 | 13.17 | 12.81 | 12.56 | 0.33 | 11.01 | 10.90 | 11.19 | 10.90 | 11.19 | 0.15 |
| LRP11 | 84918 | 7.29 | 7.28 | 8.24 | 6.45 | 7.20 | 0.64 | 5.48 | 5.73 | 6.15 | 5.03 | 5.78 | 0.42 |
| UBE2Z | 65264 | 8.21 | 7.66 | 7.86 | 8.33 | 8.13 | 0.27 | 6.53 | 6.20 | 6.63 | 6.30 | 6.25 | 0.19 |
| MYL6 | 4637 | 12.13 | 11.80 | 12.21 | 11.85 | 12.19 | 0.19 | 10.07 | 10.44 | 10.64 | 10.28 | 10.46 | 0.22 |
| GNS | 2799 | 10.89 | 10.61 | 10.76 | 10.48 | 10.38 | 0.21 | 9.02 | 8.78 | 9.06 | 8.97 | 9.03 | 0.11 |
| ARF3 | 377 | 7.37 | 6.42 | 7.70 | 6.97 | 7.18 | 0.48 | 5.52 | 5.30 | 5.71 | 5.20 | 5.65 | 0.22 |
| RHBDD2 | 57414 | 8.81 | 8.15 | 8.27 | 8.24 | 8.47 | 0.26 | 6.75 | 6.64 | 6.48 | 6.96 | 6.87 | 0.19 |
| NME1-NME2 | 654364 | 11.24 | 10.94 | 10.89 | 10.96 | 11.24 | 0.17 | 9.20 | 9.59 | 9.38 | 9.57 | 9.33 | 0.17 |
| FNDC3B | 64778 | 9.25 | 9.20 | 8.86 | 9.13 | 9.32 | 0.18 | 7.44 | 7.29 | 7.56 | 7.40 | 7.90 | 0.24 |
| TM9SF1 | 10548 | 9.19 | 8.64 | 9.15 | 8.55 | 9.07 | 0.30 | 7.24 | 7.29 | 7.47 | 7.20 | 7.27 | 0.11 |
| NEU1 | 4758 | 7.74 | 7.79 | 7.69 | 7.69 | 7.55 | 0.09 | 6.25 | 6.24 | 6.26 | 5.88 | 5.73 | 0.25 |
| FLJ35220 | 284131 | 1.63 | 2.70 | 3.43 | 3.47 | 3.20 | 0.77 | 0.00 | 1.00 | 2.20 | 1.81 | 1.32 | 0.84 |
| LOC650152 | 650152 | 11.58 | 11.58 | 11.31 | 12.09 | 11.62 | 0.28 | 9.76 | 9.94 | 10.14 | 10.05 | 10.21 | 0.18 |
| ALG3 | 10195 | 8.00 | 7.67 | 7.86 | 8.33 | 8.20 | 0.26 | 6.60 | 6.41 | 6.23 | 6.28 | 6.46 | 0.15 |
| GLG1 | 2734 | 10.84 | 10.44 | 10.66 | 10.11 | 10.81 | 0.30 | 8.85 | 8.86 | 8.93 | 8.98 | 9.16 | 0.12 |
| TAF2 | 6873 | 5.53 | 5.50 | 5.02 | 5.87 | 5.40 | 0.30 | 4.08 | 3.60 | 3.39 | 4.21 | 3.99 | 0.34 |
| sep-09 | 10801 | 10.87 | 11.30 | 11.63 | 11.55 | 11.14 | 0.31 | 9.67 | 9.62 | 9.80 | 9.50 | 9.86 | 0.14 |
| CDK4 | 1019 | 9.68 | 9.40 | 9.71 | 9.11 | 9.58 | 0.25 | 7.77 | 8.09 | 7.85 | 7.79 | 7.96 | 0.13 |
| ZMYM6 | 9204 | 8.51 | 7.94 | 8.31 | 8.13 | 8.33 | 0.22 | 6.61 | 6.45 | 6.99 | 6.28 | 6.88 | 0.30 |
| RPN1 | 6184 | 9.84 | 9.53 | 9.74 | 9.54 | 9.87 | 0.16 | 8.18 | 8.13 | 8.22 | 8.03 | 7.96 | 0.11 |
| TGFB1I1 | 7041 | 7.46 | 7.17 | 7.67 | 6.93 | 7.40 | 0.28 | 5.49 | 5.37 | 6.28 | 5.62 | 5.91 | 0.36 |
| AP3B1 | 8546 | 8.58 | 8.32 | 8.46 | 7.96 | 8.56 | 0.26 | 6.96 | 6.81 | 6.88 | 6.73 | 6.56 | 0.16 |
| DTWD1 | 56986 | 7.18 | 7.12 | 7.58 | 7.25 | 7.00 | 0.22 | 5.70 | 5.55 | 5.55 | 5.48 | 5.92 | 0.18 |
| C17orf45 | 125144 | 12.19 | 12.21 | 11.97 | 11.97 | 12.27 | 0.15 | 10.57 | 10.93 | 10.46 | 10.41 | 10.30 | 0.24 |
| TMEM183A | 92703 | 9.23 | 9.27 | 9.19 | 8.83 | 9.26 | 0.18 | 7.70 | 7.48 | 7.56 | 7.23 | 7.94 | 0.26 |
| CLN5 | 1203 | 7.23 | 6.89 | 7.28 | 6.91 | 7.02 | 0.18 | 5.55 | 5.93 | 5.58 | 5.01 | 5.44 | 0.33 |
| KTELC1 | 56983 | 8.29 | 7.93 | 7.78 | 8.00 | 7.77 | 0.21 | 6.32 | 6.24 | 6.60 | 6.63 | 6.17 | 0.21 |
| EHBP1 | 23301 | 8.66 | 8.63 | 8.91 | 8.70 | 8.68 | 0.11 | 7.08 | 7.04 | 7.15 | 7.16 | 7.35 | 0.12 |
| LOC348262 | 348262 | 7.38 | 7.32 | 7.95 | 6.99 | 7.64 | 0.36 | 6.03 | 5.89 | 6.14 | 5.79 | 5.63 | 0.20 |
| YIPF5 | 81555 | 8.09 | 8.03 | 7.84 | 7.84 | 7.98 | 0.11 | 6.47 | 6.80 | 6.16 | 5.83 | 6.73 | 0.41 |
| ABL1 | 25 | 7.91 | 7.50 | 8.19 | 7.71 | 7.75 | 0.26 | 6.39 | 6.37 | 6.31 | 6.13 | 6.11 | 0.13 |
| MEA1 | 4201 | 8.16 | 8.22 | 8.29 | 7.88 | 8.46 | 0.21 | 6.58 | 6.65 | 7.16 | 6.11 | 6.76 | 0.38 |
| LIMK1 | 3984 | 9.36 | 8.74 | 9.50 | 9.88 | 9.25 | 0.41 | 8.16 | 7.08 | 8.11 | 8.23 | 7.41 | 0.52 |
| CTTN | 2017 | 9.14 | 8.35 | 9.16 | 8.92 | 8.94 | 0.33 | 7.16 | 7.28 | 7.79 | 7.15 | 7.39 | 0.26 |
| PRMT2 | 3275 | 7.25 | 7.24 | 7.33 | 6.26 | 7.07 | 0.44 | 5.45 | 5.73 | 5.58 | 5.09 | 5.61 | 0.24 |
| COMT | 1312 | 9.98 | 9.63 | 9.64 | 9.40 | 10.14 | 0.30 | 8.34 | 8.19 | 8.22 | 8.04 | 8.34 | 0.13 |
| DSCR2 | 8624 | 8.61 | 8.12 | 7.96 | 8.53 | 8.55 | 0.29 | 6.64 | 6.58 | 6.96 | 7.01 | 6.92 | 0.20 |
| PRNP | 5621 | 10.70 | 10.62 | 10.25 | 10.86 | 10.74 | 0.23 | 8.96 | 9.11 | 9.02 | 9.11 | 9.35 | 0.15 |
| TSPO | 706 | 11.37 | 11.17 | 11.37 | 11.04 | 11.45 | 0.17 | 9.55 | 9.81 | 9.87 | 9.71 | 9.86 | 0.13 |
| COPG | 22820 | 8.52 | 8.50 | 8.22 | 8.03 | 8.56 | 0.23 | 7.01 | 6.80 | 6.84 | 6.77 | 6.82 | 0.10 |
| LRCH2 | 57631 | 5.81 | 6.00 | 6.11 | 5.50 | 5.74 | 0.24 | 4.81 | 4.14 | 4.41 | 3.94 | 4.29 | 0.33 |
| ANKRD13A | 88455 | 8.17 | 8.19 | 8.19 | 8.69 | 8.28 | 0.22 | 6.85 | 6.68 | 6.57 | 6.89 | 6.96 | 0.16 |
| ZNF226 | 7769 | 5.50 | 5.89 | 5.52 | 5.71 | 5.57 | 0.16 | 4.30 | 4.58 | 3.56 | 4.06 | 4.11 | 0.38 |
| ATP6V0E1 | 8992 | 9.14 | 9.31 | 9.03 | 9.49 | 9.42 | 0.19 | 7.77 | 7.54 | 7.83 | 7.63 | 8.10 | 0.21 |
| PPIL5 | 122769 | 6.24 | 6.41 | 6.16 | 6.53 | 6.15 | 0.17 | 4.95 | 5.13 | 4.35 | 4.64 | 4.88 | 0.30 |
| MSTO1 | 55154 | 6.10 | 6.08 | 5.87 | 6.15 | 5.22 | 0.39 | 4.85 | 4.80 | 4.28 | 4.67 | 3.28 | 0.65 |
| TXNDC1 | 81542 | 8.59 | 8.48 | 8.48 | 8.26 | 8.43 | 0.12 | 7.06 | 7.21 | 6.96 | 6.78 | 6.73 | 0.20 |
| RALA | 5898 | 9.83 | 9.89 | 9.29 | 10.31 | 9.66 | 0.37 | 8.28 | 8.34 | 8.09 | 8.35 | 8.43 | 0.13 |
| SAMD9L | 219285 | 6.76 | 6.61 | 6.93 | 5.81 | 6.16 | 0.46 | 5.27 | 5.17 | 5.22 | 4.50 | 4.62 | 0.37 |
| NME4 | 4833 | 9.50 | 9.73 | 9.73 | 9.40 | 9.86 | 0.19 | 8.13 | 8.41 | 8.04 | 7.77 | 8.42 | 0.27 |
| CAPRIN2 | 65981 | 5.95 | 6.11 | 5.62 | 6.70 | 5.92 | 0.40 | 4.50 | 4.54 | 4.14 | 5.00 | 4.68 | 0.31 |
| ZMPSTE24 | 10269 | 9.76 | 9.71 | 9.67 | 9.77 | 9.49 | 0.11 | 7.96 | 8.08 | 8.29 | 8.10 | 8.53 | 0.22 |
| TXNL5 | 84817 | 10.50 | 10.99 | 10.63 | 10.31 | 10.75 | 0.26 | 9.02 | 9.10 | 9.17 | 9.20 | 9.31 | 0.11 |
| C9orf30 | 91283 | 7.89 | 8.13 | 8.23 | 8.55 | 7.90 | 0.27 | 6.68 | 6.56 | 6.62 | 6.80 | 6.73 | 0.10 |
| GNL1 | 2794 | 7.14 | 6.72 | 7.86 | 6.99 | 7.24 | 0.42 | 5.62 | 5.27 | 5.96 | 5.88 | 5.92 | 0.29 |
| APRT | 353 | 7.08 | 6.80 | 6.95 | 6.80 | 7.17 | 0.17 | 5.68 | 5.82 | 5.52 | 5.03 | 5.52 | 0.30 |
| NIPA2 | 81614 | 8.70 | 8.61 | 8.07 | 8.75 | 8.65 | 0.28 | 7.18 | 7.12 | 7.04 | 7.11 | 7.09 | 0.05 |
| DBNL | 28988 | 8.67 | 8.38 | 8.77 | 8.41 | 8.76 | 0.19 | 7.03 | 7.14 | 7.30 | 6.87 | 7.43 | 0.22 |
| CETN2 | 1069 | 10.09 | 9.97 | 10.06 | 9.53 | 9.91 | 0.23 | 8.30 | 8.54 | 8.58 | 8.38 | 8.58 | 0.13 |
| SH3PX3 | 257364 | 6.55 | 6.17 | 6.36 | 6.21 | 6.14 | 0.17 | 4.99 | 4.89 | 4.94 | 4.78 | 4.67 | 0.13 |
| PTRF | 284119 | 12.28 | 11.62 | 12.28 | 12.68 | 12.24 | 0.38 | 10.59 | 10.51 | 11.15 | 10.88 | 10.85 | 0.25 |
| NOLA3 | 55505 | 10.90 | 10.97 | 10.48 | 11.27 | 11.07 | 0.29 | 9.45 | 9.63 | 9.31 | 9.58 | 9.60 | 0.14 |
| C14orf32 | 93487 | 7.09 | 7.32 | 7.31 | 8.00 | 7.28 | 0.35 | 5.74 | 6.06 | 6.20 | 6.20 | 5.74 | 0.23 |
| ACTL6A | 86 | 7.59 | 7.64 | 7.34 | 7.79 | 7.39 | 0.18 | 6.20 | 6.52 | 6.14 | 5.85 | 6.00 | 0.25 |
| WDR1 | 9948 | 10.21 | 9.92 | 10.25 | 10.39 | 10.12 | 0.17 | 8.80 | 8.70 | 8.88 | 8.69 | 8.82 | 0.08 |
| IQGAP1 | 8826 | 7.96 | 8.24 | 8.20 | 8.04 | 7.59 | 0.26 | 6.62 | 6.48 | 6.84 | 6.45 | 6.65 | 0.15 |
| SEC31A | 22872 | 8.34 | 8.23 | 8.12 | 8.15 | 8.33 | 0.10 | 6.72 | 6.62 | 7.06 | 6.58 | 7.21 | 0.28 |
| POFUT1 | 23509 | 7.42 | 6.74 | 7.13 | 7.01 | 7.12 | 0.24 | 5.73 | 5.72 | 5.62 | 5.70 | 5.73 | 0.04 |
| SARS | 6301 | 11.25 | 11.00 | 10.45 | 10.67 | 11.08 | 0.32 | 9.47 | 9.63 | 9.33 | 9.61 | 9.51 | 0.12 |
| GNB1 | 2782 | 10.51 | 10.53 | 10.42 | 10.52 | 10.86 | 0.17 | 9.25 | 8.81 | 9.38 | 9.08 | 9.51 | 0.27 |
| TAP1 | 6890 | 8.39 | 8.40 | 8.56 | 8.56 | 8.47 | 0.08 | 6.83 | 6.88 | 7.24 | 7.54 | 7.09 | 0.29 |
| APRT | 353 | 7.90 | 7.14 | 7.78 | 7.84 | 7.78 | 0.31 | 6.58 | 6.21 | 6.43 | 6.24 | 6.19 | 0.17 |
| NAT9 | 26151 | 6.27 | 6.06 | 6.29 | 6.42 | 6.47 | 0.16 | 5.00 | 4.95 | 4.70 | 5.13 | 4.94 | 0.16 |
| DAD1 | 1603 | 10.93 | 11.08 | 10.78 | 10.78 | 11.26 | 0.21 | 9.55 | 9.72 | 9.69 | 9.45 | 9.63 | 0.11 |
| RPLP0 | 6175 | 12.01 | 12.27 | 11.78 | 11.55 | 12.04 | 0.27 | 10.34 | 10.72 | 10.81 | 10.47 | 10.57 | 0.19 |
| COMMD2 | 51122 | 7.26 | 7.50 | 6.77 | 7.24 | 7.31 | 0.27 | 5.48 | 6.31 | 5.75 | 5.82 | 5.98 | 0.30 |
| TMEM184B | 25829 | 7.36 | 6.65 | 7.25 | 7.26 | 7.17 | 0.28 | 5.87 | 5.10 | 6.25 | 5.81 | 5.96 | 0.42 |
| YIF1A | 10897 | 9.71 | 9.60 | 9.23 | 9.22 | 9.85 | 0.28 | 8.10 | 8.18 | 8.19 | 8.21 | 8.30 | 0.07 |
| PAICS | 10606 | 8.74 | 8.62 | 8.68 | 8.56 | 8.79 | 0.09 | 7.39 | 7.65 | 7.00 | 7.17 | 7.57 | 0.27 |
| AP2S1 | 1175 | 12.23 | 12.01 | 11.92 | 12.07 | 12.15 | 0.12 | 10.77 | 10.90 | 10.49 | 10.67 | 10.95 | 0.19 |
| PRRC1 | 133619 | 7.67 | 7.85 | 7.53 | 7.75 | 7.56 | 0.13 | 6.22 | 6.31 | 6.41 | 6.37 | 6.45 | 0.09 |
| RPS19 | 6223 | 13.67 | 13.81 | 13.81 | 13.75 | 13.61 | 0.09 | 12.36 | 12.63 | 12.47 | 12.30 | 12.32 | 0.14 |
| SLC35B2 | 347734 | 8.64 | 7.97 | 8.42 | 7.97 | 8.53 | 0.32 | 7.09 | 6.74 | 7.31 | 6.91 | 6.96 | 0.22 |
| TRAPPC3 | 27095 | 8.77 | 7.90 | 8.52 | 8.50 | 8.61 | 0.33 | 7.47 | 6.51 | 7.10 | 7.44 | 7.28 | 0.39 |
| PHLDB1 | 23187 | 7.48 | 7.68 | 7.92 | 7.71 | 8.05 | 0.22 | 6.12 | 6.63 | 6.48 | 6.65 | 6.49 | 0.21 |
| BUD31 | 8896 | 10.08 | 9.94 | 9.50 | 9.94 | 10.28 | 0.29 | 8.70 | 8.73 | 8.67 | 8.41 | 8.74 | 0.14 |
| B2M | 567 | 11.91 | 11.67 | 12.47 | 11.96 | 11.85 | 0.30 | 10.46 | 10.55 | 10.89 | 10.75 | 10.75 | 0.17 |
| AP2S1 | 1175 | 9.94 | 10.00 | 9.55 | 9.55 | 10.04 | 0.25 | 8.61 | 8.65 | 8.57 | 8.25 | 8.54 | 0.16 |
| BET1L | 51272 | 9.11 | 8.94 | 9.21 | 8.88 | 9.00 | 0.13 | 7.72 | 7.95 | 7.59 | 7.55 | 7.87 | 0.17 |
| TM9SF3 | 56889 | 7.91 | 8.26 | 7.94 | 7.82 | 8.02 | 0.17 | 6.87 | 6.70 | 6.41 | 6.62 | 6.89 | 0.20 |
| TSEN34 | 79042 | 8.52 | 8.32 | 8.48 | 7.81 | 8.62 | 0.32 | 7.05 | 7.01 | 7.13 | 6.95 | 7.17 | 0.09 |
| GPAA1 | 8733 | 7.49 | 6.77 | 7.41 | 6.94 | 7.26 | 0.31 | 6.22 | 5.50 | 6.47 | 5.50 | 5.75 | 0.44 |
| STK4 | 6789 | 8.35 | 7.60 | 8.24 | 8.45 | 8.20 | 0.33 | 6.91 | 6.75 | 7.10 | 6.94 | 6.72 | 0.16 |
| TMCO1 | 54499 | 9.52 | 9.73 | 9.41 | 9.67 | 9.63 | 0.13 | 8.30 | 8.46 | 8.44 | 8.12 | 8.26 | 0.14 |
| SAP30L | 79685 | 7.51 | 7.39 | 7.25 | 7.55 | 7.46 | 0.12 | 6.28 | 6.06 | 5.95 | 6.12 | 6.36 | 0.16 |
| PEX16 | 9409 | 6.01 | 5.54 | 5.59 | 6.42 | 5.89 | 0.36 | 4.88 | 4.20 | 4.19 | 5.04 | 4.80 | 0.40 |
| UBE2I | 7329 | 8.95 | 8.58 | 8.78 | 9.32 | 9.02 | 0.28 | 7.73 | 7.53 | 7.85 | 7.76 | 7.47 | 0.16 |
| C20orf117 | 140710 | 6.57 | 6.90 | 7.38 | 7.37 | 6.59 | 0.40 | 5.54 | 5.33 | 6.08 | 5.89 | 5.66 | 0.30 |
| PDCD6 | 10016 | 8.95 | 8.92 | 9.07 | 9.22 | 8.90 | 0.13 | 7.78 | 7.94 | 7.60 | 7.68 | 7.75 | 0.13 |
| GLTP | 51228 | 9.74 | 9.77 | 9.64 | 9.67 | 9.65 | 0.06 | 8.15 | 8.32 | 8.60 | 8.41 | 8.70 | 0.22 |
| SH3BGRL | 6451 | 8.82 | 8.68 | 9.55 | 8.28 | 8.72 | 0.46 | 7.38 | 7.17 | 8.26 | 7.18 | 7.78 | 0.46 |
| PHF11 | 51131 | 7.94 | 7.84 | 8.46 | 7.98 | 7.97 | 0.24 | 6.58 | 6.77 | 6.87 | 7.03 | 6.71 | 0.17 |
| ARPC1A | 10552 | 9.68 | 9.12 | 9.68 | 9.38 | 9.60 | 0.24 | 8.14 | 8.25 | 8.55 | 8.03 | 8.25 | 0.19 |
| C20orf3 | 57136 | 7.44 | 6.87 | 7.38 | 7.06 | 7.21 | 0.24 | 6.15 | 5.82 | 6.09 | 6.10 | 5.57 | 0.25 |
| sep-15 | 9403 | 9.83 | 9.86 | 9.93 | 10.09 | 9.91 | 0.10 | 8.60 | 8.75 | 8.68 | 8.53 | 8.83 | 0.12 |
| ADAR | 103 | 9.07 | 8.63 | 9.34 | 8.86 | 8.93 | 0.26 | 7.58 | 7.64 | 7.84 | 7.87 | 7.73 | 0.13 |
| TICAM1 | 148022 | 7.47 | 7.32 | 7.21 | 8.23 | 7.25 | 0.42 | 6.35 | 6.11 | 5.97 | 6.63 | 6.28 | 0.25 |
| B3GALT6 | 126792 | 6.66 | 6.46 | 6.84 | 6.98 | 6.80 | 0.20 | 5.68 | 5.15 | 5.81 | 5.40 | 5.55 | 0.26 |
| CYP2U1 | 113612 | 6.01 | 5.85 | 6.44 | 5.72 | 5.73 | 0.30 | 4.60 | 4.49 | 5.13 | 4.69 | 4.72 | 0.24 |
| C9orf19 | 152007 | 8.37 | 8.37 | 8.88 | 8.21 | 8.54 | 0.25 | 6.70 | 7.17 | 7.65 | 7.26 | 7.47 | 0.36 |
| PLEKHO1 | 51177 | 6.59 | 6.58 | 6.61 | 7.32 | 6.71 | 0.32 | 5.38 | 5.59 | 5.01 | 6.22 | 5.50 | 0.44 |
| TMEM117 | 84216 | 5.56 | 6.05 | 5.46 | 4.91 | 5.83 | 0.43 | 4.47 | 4.55 | 4.08 | 3.98 | 4.63 | 0.29 |
| C14orf135 | 64430 | 7.32 | 7.37 | 7.53 | 7.13 | 7.33 | 0.14 | 6.00 | 6.33 | 6.00 | 6.02 | 6.28 | 0.16 |
| GEMIN6 | 79833 | 6.95 | 6.89 | 6.91 | 7.04 | 6.82 | 0.08 | 5.69 | 6.13 | 5.73 | 5.53 | 5.56 | 0.24 |
| ZZZ3 | 26009 | 6.95 | 7.11 | 6.56 | 7.51 | 6.63 | 0.38 | 5.80 | 5.96 | 5.50 | 6.04 | 5.52 | 0.25 |
| PPP4C | 5531 | 8.60 | 8.23 | 8.22 | 8.64 | 8.76 | 0.25 | 7.59 | 7.29 | 6.98 | 7.33 | 7.32 | 0.22 |
| UNC45A | 55898 | 6.91 | 6.56 | 7.09 | 6.72 | 6.81 | 0.20 | 5.64 | 5.53 | 6.11 | 5.67 | 5.21 | 0.32 |
| PANK2 | 80025 | 7.75 | 7.33 | 7.38 | 7.89 | 7.51 | 0.24 | 6.49 | 6.24 | 6.18 | 6.48 | 6.59 | 0.18 |
| CDK2AP1 | 8099 | 11.67 | 11.79 | 11.62 | 11.57 | 11.88 | 0.13 | 10.54 | 10.39 | 10.32 | 10.58 | 10.86 | 0.21 |
| BCAP31 | 10134 | 9.40 | 9.58 | 9.43 | 9.55 | 9.70 | 0.12 | 8.33 | 8.35 | 8.42 | 8.34 | 8.39 | 0.04 |
| LACTB | 114294 | 4.87 | 4.49 | 4.71 | 4.55 | 4.89 | 0.18 | 3.77 | 3.29 | 3.66 | 3.62 | 3.38 | 0.20 |
| CTNNA1 | 1495 | 9.97 | 10.23 | 9.85 | 9.88 | 10.08 | 0.16 | 8.82 | 8.76 | 8.98 | 8.78 | 8.94 | 0.10 |
| WASF3 | 10810 | 6.76 | 6.29 | 6.31 | 6.40 | 6.73 | 0.23 | 5.47 | 5.32 | 5.44 | 5.02 | 5.56 | 0.21 |
| CASC4 | 113201 | 8.89 | 8.70 | 8.78 | 8.36 | 8.71 | 0.20 | 7.56 | 7.60 | 7.54 | 7.27 | 7.82 | 0.20 |
| GOLGA5 | 9950 | 8.50 | 8.52 | 7.97 | 8.40 | 8.30 | 0.22 | 7.22 | 7.22 | 7.13 | 7.35 | 7.10 | 0.10 |
| RBM4 | 5936 | 6.34 | 6.85 | 6.87 | 6.73 | 6.65 | 0.21 | 5.46 | 5.40 | 5.94 | 5.40 | 5.58 | 0.23 |
| GTF2H3 | 2967 | 5.08 | 5.11 | 4.49 | 5.04 | 4.98 | 0.26 | 3.92 | 4.11 | 3.57 | 3.61 | 3.85 | 0.22 |
| NCLN | 56926 | 7.58 | 7.35 | 7.39 | 8.30 | 7.67 | 0.38 | 6.69 | 6.45 | 6.14 | 6.82 | 6.54 | 0.26 |
| HPS5 | 11234 | 5.82 | 6.27 | 5.32 | 5.54 | 6.11 | 0.39 | 4.69 | 4.95 | 4.55 | 4.38 | 4.85 | 0.23 |
| RPS12 | 6206 | 12.87 | 12.85 | 12.73 | 12.84 | 12.92 | 0.07 | 11.70 | 11.75 | 11.91 | 11.67 | 11.56 | 0.13 |
| MTPN | 136319 | 8.61 | 9.07 | 8.61 | 8.38 | 8.79 | 0.25 | 7.78 | 7.59 | 7.50 | 7.23 | 7.76 | 0.22 |
| XPNPEP1 | 7511 | 9.49 | 9.40 | 8.93 | 9.17 | 9.34 | 0.22 | 8.24 | 7.96 | 8.06 | 8.31 | 8.16 | 0.14 |
| LOC440589 | 440589 | 12.43 | 12.20 | 12.25 | 12.04 | 12.25 | 0.14 | 11.31 | 11.11 | 11.34 | 11.13 | 10.83 | 0.20 |
| DNAL4 | 10126 | 7.78 | 7.49 | 7.94 | 7.38 | 7.71 | 0.23 | 6.35 | 6.46 | 6.98 | 6.50 | 6.57 | 0.24 |
| RARS2 | 57038 | 7.50 | 7.10 | 7.09 | 7.32 | 7.56 | 0.22 | 6.19 | 6.34 | 6.09 | 6.26 | 6.26 | 0.09 |
| MRLC2 | 103910 | 11.42 | 11.39 | 11.19 | 11.31 | 11.57 | 0.14 | 10.17 | 10.47 | 10.41 | 10.14 | 10.29 | 0.15 |
| POLR2H | 5437 | 9.00 | 9.07 | 9.37 | 9.22 | 8.86 | 0.20 | 7.95 | 8.25 | 8.06 | 8.07 | 7.87 | 0.14 |
| CLCN7 | 1186 | 9.57 | 9.11 | 9.56 | 9.54 | 9.13 | 0.24 | 8.61 | 8.03 | 8.16 | 8.50 | 8.36 | 0.24 |
| SDCCAG3 | 10807 | 6.86 | 6.77 | 6.83 | 7.35 | 6.61 | 0.28 | 6.14 | 5.87 | 5.66 | 6.16 | 5.35 | 0.34 |
| ARFIP1 | 27236 | 7.35 | 7.23 | 7.06 | 7.14 | 7.07 | 0.12 | 6.06 | 6.41 | 6.06 | 6.05 | 6.04 | 0.16 |
| HSPC152 | 51504 | 10.26 | 10.19 | 10.29 | 9.55 | 10.37 | 0.33 | 9.09 | 9.15 | 9.31 | 8.80 | 9.10 | 0.18 |
| TIPRL | 261726 | 6.61 | 6.68 | 6.55 | 6.21 | 7.19 | 0.35 | 5.31 | 5.51 | 5.72 | 5.40 | 6.11 | 0.32 |
| SRP72 | 6731 | 7.91 | 7.87 | 7.55 | 7.91 | 7.75 | 0.15 | 6.68 | 6.97 | 6.68 | 6.90 | 6.61 | 0.16 |
| SAE1 | 10055 | 9.91 | 10.02 | 9.88 | 9.70 | 9.85 | 0.12 | 8.87 | 8.84 | 8.84 | 8.97 | 8.77 | 0.07 |
| KATNB1 | 10300 | 5.94 | 6.08 | 5.45 | 6.14 | 5.94 | 0.27 | 5.12 | 4.74 | 4.60 | 5.00 | 5.00 | 0.21 |
| TPCN2 | 219931 | 4.84 | 4.03 | 5.31 | 5.52 | 4.52 | 0.60 | 3.61 | 3.28 | 4.47 | 4.31 | 3.49 | 0.53 |
